# Insights into translocation mechanism and ribosome evolution from cryo-EM structures of translocation intermediates of *Giardia intestinalis*

**DOI:** 10.1093/nar/gkad176

**Published:** 2023-03-13

**Authors:** Soneya Majumdar, Andrew Emmerich, Sascha Krakovka, Chandra Sekhar Mandava, Staffan G Svärd, Suparna Sanyal

**Affiliations:** Department of Cell and Molecular Biology, Uppsala University, Box 596, 75124 Uppsala, Sweden; Department of Cell and Molecular Biology, Uppsala University, Box 596, 75124 Uppsala, Sweden; Department of Cell and Molecular Biology, Uppsala University, Box 596, 75124 Uppsala, Sweden; Department of Cell and Molecular Biology, Uppsala University, Box 596, 75124 Uppsala, Sweden; Department of Cell and Molecular Biology, Uppsala University, Box 596, 75124 Uppsala, Sweden; Department of Cell and Molecular Biology, Uppsala University, Box 596, 75124 Uppsala, Sweden

## Abstract

*Giardia intestinalis* is a protozoan parasite that causes diarrhea in humans. Using single-particle cryo-electron microscopy, we have determined high-resolution structures of six naturally populated translocation intermediates, from ribosomes isolated directly from actively growing *Giardia* cells. The highly compact and uniquely GC-rich *Giardia* ribosomes possess eukaryotic rRNAs and ribosomal proteins, but retain some bacterial features. The translocation intermediates, with naturally bound tRNAs and eukaryotic elongation factor 2 (eEF2), display characteristic ribosomal intersubunit rotation and small subunit’s head swiveling—universal for translocation. In addition, we observe the eukaryote-specific ‘subunit rolling’ dynamics, albeit with limited features. Finally, the eEF2·GDP state features a uniquely positioned ‘leaving phosphate (Pi)’ that proposes hitherto unknown molecular events of Pi and eEF2 release from the ribosome at the final stage of translocation. In summary, our study elucidates the mechanism of translocation in the protists and illustrates evolution of the translation machinery from bacteria to eukaryotes from both the structural and mechanistic perspectives.

## INTRODUCTION

Translation elongation involves three sequential reactions: (i) decoding and accommodation of aminoacyl tRNA in the A (aminoacyl tRNA) site; (ii) peptide bond formation followed by generation of pre-translocation (PRE-T) complex with deacylated tRNA in the P (peptidyl tRNA) site and peptidyl tRNA in the A site; and (iii) translocation of A- and P-site tRNAs to the P and E (exit) sites through multiple substeps resulting in the post-translocation (POST-T) state ready for next cycle of elongation [reviewed in (1–3)]. Translocation of tRNAs requires rotation of the small ribosomal subunit (SSU) relative to the large subunit (LSU) as well as a swiveling motion of the SSU head relative to the body (1,4). The ribosome in the PRE-T state spontaneously samples between the nonrotated and rotated states of the SSU relative to the LSU (5,6). Concomitantly, the acceptor arms of the tRNAs shift from the A and P sites toward the P and E sites in the rotated state, adopting hybrid conformations (5–10). However, the movement of the anticodon stem loops (ASLs) of tRNAs and mRNA along the SSU is the most crucial step of translocation, which is catalyzed by a conserved GTPase factor, elongation factor G (EF-G) in bacteria or eukaryotic elongation factor 2 (EF2) in archaea and eukaryotes (eEF2) (3,6,11–15).

The role of EF-G in tRNA translocation has been extensively investigated in bacteria through biochemical (12,16–18), structural (19–26) and single-molecule fluorescence energy transfer studies (27–31). Two schools of thought have emerged from these studies. The first one views EF-G as a propelling motor and proposes that GTP hydrolysis in EF-G leads to large conformational changes in EF-G and induces ribosomal rearrangements, which in turn facilitates forward movement of the tRNAs (3,25,32–35). The second model, based on the observations that (i) tRNA translocation on the ribosome can occur slowly in the absence of EF-G and (ii) EF-G does not undergo major conformational changes during translocation once bound to the Ap/P state, proposes that translocation is an inherent property of the ribosome and not exclusively dependent on GTP hydrolysis on EF-G (12,36–40). Here, EF-G acts as a pawl that hinders the intersubunit rotational motion of the ribosome and uncouples the entire tRNA–mRNA module from SSU (19). Thereafter, back-rotation and back-swiveling of the SSU body and head lead to completion of translocation. Recently, there are multiple time-resolved cryo-electron microscopy (cryo-EM) studies on EF-G-mediated translocation (25,26,41). In spite of that, the debate for establishment of the role of GTP hydrolysis in translocation is still unsettled. While there has been a lot of focus on pre-GTP hydrolysis steps, the sequence of post-GTP hydrolysis events, particularly the exact mechanism of phosphate (Pi) release and dissociation of EF-G/eEF2 from the ribosome, which lead to completion of one round of elongation, has not been fully clarified in the existing literature.

Despite similarity in the main mechanism of tRNA translocation along all three kingdoms of life, differences exist between eukaryotes and bacteria. This is partially due to different architecture of the tRNA binding sites that lead to different interactions of the tRNAs with the ribosome (7,42,43). Furthermore, the eukaryotic SSU exhibits an additional rotational motion, termed as ‘subunit rolling’, that is nearly orthogonal to the canonical SSU rotation observed in bacterial translocation (42,43). Subunit rolling occurs after codon recognition at the A site and makes eukaryotic ribosome conformation in the classical PRE-T and POST-T states distinct, which are otherwise identical in bacterial translation (7,42–45).

Although our knowledge about eukaryotic translation has developed a lot in recent years, studies investigating mechanisms of translation in unicellular parasitic eukaryotes are scarce. Structures of cytoplasmic ribosomes are available for *Giardia lamblia* (46,47), *Trichomonas vaginalis* (48), *Trypanosoma brucei* (49,50), *Vairimorpha necatrix* (51), *Toxoplasma gondii* (48) and *Plasmodium falciparum* (52,53), which present distinctive features compared to higher eukaryotic ribosomes. For example, *G. lamblia*, *T. vaginalis* and *V. necatrix* all have a highly reduced rRNA devoid of most eukaryote-specific expansion segments (ESs) (48,51), while *T. brucei* 28S rRNA has six fragments stabilized by species-specific ESs or ribosomal protein (r-protein) extensions (49,50). Alongside these ESs, the process of translation initiation, fidelity and termination has also developed special features during the course of evolution (54–57). However, there are no studies available investigating translocation steps in protozoan parasites so far. The question remains whether translocation in these ribosomes resembles more closely bacterial or eukaryotic translocation—including the characteristic ‘subunit rolling’.

*Giardia intestinalis* (syn. *G. lamblia* and *G. duodenalis*) is a flagellated, amitochondrial, binucleated, protozoan parasite of the order Diplomonadida, which causes diarrhea in humans and other mammals (58). Besides being a pathogen, *Giardia* has been of interest from the perspective of evolution of eukaryotic cells. It belongs to the Metamonada supergroup, a divergent eukaryotic lineage that has been used in many comparative studies of basic cellular processes. This feature together with the ease to culture in the laboratory environment makes *Giardia* a very interesting model organism to study the mechanistic steps of translation in lower eukaryotes.

In this study, we determined the structure of the native cytosolic ribosomes isolated in bulk from actively growing *Giardia* cells at an overall resolution of 2.95 Å, using single-particle cryo-EM. Further, through extensive *in silico* sorting, we visualized six individual translational states with tRNAs and eEF2 trapped on the ribosome. These states, trapped without any inhibitor or nonhydrolyzable GTP analogs, represent authentic translocation-intermediate states in which rotation of the SSU relative to the LSU, eEF2 binding, the swiveling motion of the SSU head relative to the body and SSU rolling/back-rolling coupled to translocation of tRNA from the A to P site and from the P to E site could be visualized in near-atomic detail. The eEF2·GDP-bound ‘late’ translocation-intermediate state portrays a ‘leaving Pi’ group in a unique location. This state, in comparison with similar states reported before, elucidates the sequence of molecular events that guide Pi release at the final step of tRNA translocation. We have also quantified the rotational movements of the SSU body and head, which allowed us to characterize and compare the ‘subunit rotation’ and ‘subunit rolling’ dynamics, also between ribosomes of different origin. Altogether, our study with high-resolution structures of the translocation intermediates of the *Giardia* ribosome presents for the first time the mechanism of translocation in a protist. Moreover, of general interest, it elucidates gradual evolution of the translation machinery from bacteria to higher eukaryotes from both structural and mechanistic perspectives.

## MATERIALS AND METHODS

### Culturing *Giardia* cells

*Giardia* trophozoites of strain WB-C6 (ATC 50803) were maintained in TYI-S-33 medium. For the experiment, the cells were grown in 50 ml centrifugation tubes. At a cell density of around 1 × 10^6^ cells/ml, the cells were harvested by cooling the tubes on ice for 15 min, agitating the tubes repeatedly to detach the cells from the tube walls and centrifuging the tubes at 2000 × *g* and 4°C for 5 min. The supernatant was decanted; the pellets were washed twice in PBS and pooled into microcentrifugation tubes with ∼0.7 ml of pellet each. These pellets were stored at −80°C until ribosome extraction.

### Ribosome preparation

Ribosomes from *G. intestinalis* were prepared according to (59) with minor modification. Approximately 300 mg of (wet weight) *Giardia* trophozoites were resuspended in a low-salt buffer [100 mM NH_4_OAc, 15 mM Mg(OAc)_2_, 20 mM Tris–HCl (pH 7.6) and 6 mM β-ME). Lysis was carried out by vortexing the resuspended trophozoites with 300 mg of silica beads (0.5 mm diameter) for 10 min with intermediate incubation on ice. Lysate was clarified by centrifugation at 20 000 × *g* for 30 min at 4°C. To separate the ribosomes from other cellular contents, the cleared lysate was layered on 30% sucrose cushion in a low-salt buffer with 1:1 ratio and centrifuged for 2 h at 250 000 × *g* in a Sorvall RC M150 GX ultracentrifuge. The ribosome pellets were washed and resuspended in a high-salt buffer (same as a low-salt buffer but with 1 M NH_4_OAc). For further clarification, resuspended ribosomes were layered on a 30% sucrose cushion prepared in a high-salt buffer and centrifuged as above. The resulted ribosome pellets were washed and resuspended in HEPES–polymix buffer [5 mM HEPES (pH 7.5), 95 mM KCl, 5 mM NH_4_Cl, 5 mM Mg(OAc)_2_, 8 mM putrescine, 0.5 mM CaCl_2_, 1 mM spermidine and 1 mM 1,4-dithioerythritol buffer] (60) and further stored at −80°C after shock freezing in liquid nitrogen.

### Grid preparation and imaging

Grids used for imaging were R2/2 300 mesh copper grids (Quantifoil) with a 2 nm amorphous carbon layer. The grids were glow discharged (PELCO easiGlow, 20 mA, 30 s) immediately before use. Vitrification of the sample (3 μl) was done using a Vitrobot Mark IV (FEI Thermo Fisher, 4°C, 100% humidity, blot force 0, blot time 3 s). Grids were screened on a Talos Arctica (FEI Thermo Fisher) and imaged on a Titan Krios (FEI Thermo Fisher) at 300 keV using a K2 direct electron detector (Gatan) at a resolution of 0.82 Å/pixel and nominal defocus of 1.2 μm. Image stacks of 20 frames with electron dose of 1.5 e/Å^2^ per frame (4 frames/s) were captured with 7654 image stacks in total.

### Cryo-EM image analysis

The cryo-EM image reconstruction pipeline is summarized in Supplementary Figure S1A. Using MotionCor2 (61), image stacks were gain corrected, dose weighted and aligned. CTFFIND-4.1 (62) was used to estimate the contrast transfer function of each aligned micrograph. All further image processing was done with RELION 3.1.2 (63). From a random subset of micrographs, particles were manually picked, followed by 2D classification. With suitable 2D classes, reference-based particle picking was performed on all micrographs yielding 682.2k particles (extracted with 5× binning, 500 to 100 pixels). These particles were 2D classified (383.2k particles) and suitable class averages chosen and 3D refined with a global mask followed by 3D classification (205.2k particles) (Supplementary Table S1). 3D refinement of unbinned particles (500 pixels at 0.82 Å/pixel) yielded an initial 3.15 Å resolution electron density map. CTF refinement and Bayesian polishing improved this resolution to 2.96 Å. Masked refinements of the large subunit (2.75 Å) and small subunit (2.91 Å) were used to build the *de novoGiardia* ribosome structure, though the poor density of the unmasked regions of the ribosome indicated significant residual structural heterogeneity. Structural heterogeneity was addressed first with 3D refinement using a large subunit mask (using 2.5× binned particles, with the 383.2k post-2D classification particle set), followed by two rounds of fixed orientation 3D classification. The first round of fixed orientation 3D classification using SSU mask dealt with the conformational heterogeneity attributable to the SSU (12 classes, yielding 9 suitable subclasses, representing 4 major SSU conformations). The second round of fixed orientation 3D classification was employed on each distinct SSU conformation using a mask encompassing the P stalk, APE sites and the L1 stalk (Supplementary Figure S1A), which was generated with custom code. This second round of fixed orientation 3D classification addressed any remaining conformational and (in particular) compositional heterogeneity. Unbinned (500 pixels at 0.82 Å/pixel) polished particles were then 3D refined to give electron density maps for each of these final classes. Final map resolutions together with cryo-EM data are shown in Supplementary Table S2. Fourier shell correlation curves for half maps (left panel) and map to model are presented in Supplementary Figure S1B. The local resolution as well as angular distribution of the ribosomal particles is presented in Supplementary Figure S2A. The surface resolution of eEF2 bound to state B with magnified view of the nucleotide binding pocket is shown in Supplementary Figure S2B.

### Structure building

The core of the *de novo* structure of the *Giardia* ribosome was built in Coot (64) using the human ribosome as reference (PDB ID: 6w6l, 4v6x). ClustalW in BioPython was then used to align the conserved core to the *G. intestinalis* rRNA (ATCC 50803/WB clone C6) followed by assignment of mutations and insertions/deletions as peripheral regions of the ribosomal rRNA were added. Initial *Giardia* r-protein structures and eEF2 were predicted using Phyre2 (65) through homology modeling. Subsequent structures were real-space refined in Coot using a custom script. As tRNA and mRNA represent an ensemble of molecules, nucleotide assignments for these molecules were made according to the available electron density. Representative cryo-EM densities are presented in Supplementary Figures S3 and S4.

### Determination of SSU rRNA residue displacement and rotational characteristics

Superimposition of all atomic structures showed no significant changes in LSU conformation. From the four major states with distinct SSU conformations, the classical PRE-T state C SSU was chosen as reference to determine the direction and magnitude of movement (the Euclidean norm) between it and the remaining three states (D, B and A) (Supplementary Figure S5). Two steps were used to characterize intersubunit rotation. First, singular value decomposition was used to align the SSU body of state C to the origin, with all other structures then transformed similarly. This ensures that there is minimal movement along the axis of rotation confining the majority of movement to a single plane for most state transitions. Second, the transformation matrix that minimized the RMSD of the SSU body and/or head from one state to another was calculated. Using a custom script, this transformation matrix was then decomposed using the axis–angle representation to obtain the rotation axis and angle of rotation about this axis (Supplementary Table S3A, B and Supplementary Figure S6). The rotational axes and vectors for SSU head/body movements were determined in ChimeraX (Supplementary Figure S6 and Supplementary Movie A). SSU head (residues 895–1315) movement was further characterized by aligning the rRNA residues of the SSU body (1–894, 1316–1454) of all states to state C with similar analysis performed to determine residue displacement and isolated SSU head rotational characteristics (Supplementary Table S3C and Supplementary Figure S5). L1-stalk movement in states A-I to A-VI was characterized and visualized in a similar manner (Figure 3C and Supplementary Table S3D).

## RESULTS

### *Giardia* ribosomes are highly compact and uniquely rich in GC content

**Figure 1.**
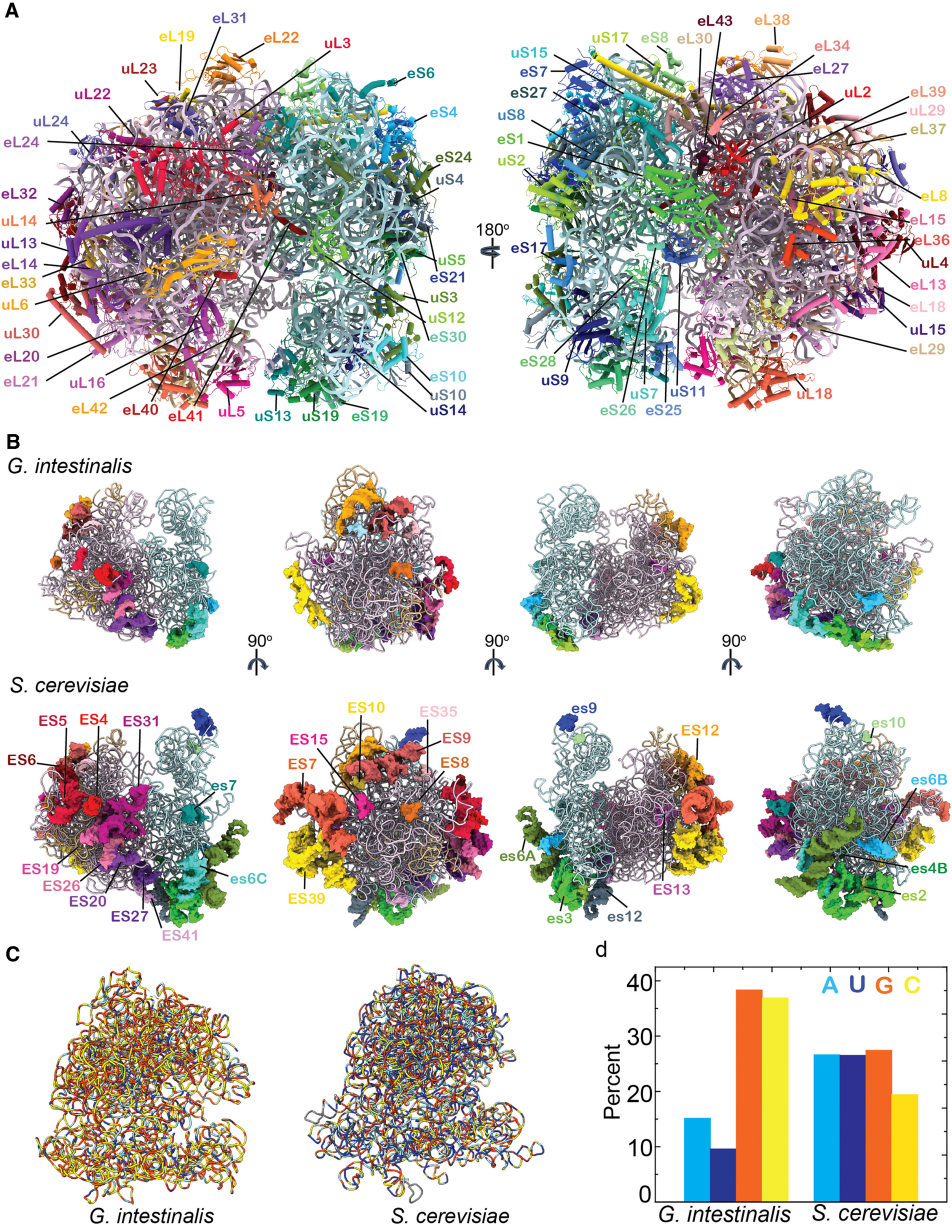
Cryo-EM structure of the *Giardia* ribosome reveals a highly compact, GC-rich rRNA with eukaryotic r-proteins. (**A**) Structure of the *Giardia* ribosome with all the r-proteins colored (SSU, shades of blue and green; LSU, shades of red and purple) and labeled. (**B**) Four views (90° related) of the *Giardia* (top) and *Saccharomyces* (bottom) ribosomes showing rRNA ESs (ES for LSU, colored in shades of red and purple; es for SSU, colored in shades of blue and green). ESs are similarly colored in both ribosomes for size comparison. (**C**) Distribution of A (blue), U (dark blue), G (orange) and C (yellow) nucleotides across the rRNA in *Giardia* and *Saccharomyces* ribosomes. (**D**) Percent A, U, G and C in *Giardia* and *Saccharomyces* ribosomes compared in a histogram.

We have determined the cryo-EM structure of the *G. intestinalis* ribosome at an overall resolution of 2.95 Å (Figure 1A and Supplementary Figure S1A). Clear densities could be observed for all universal and eukaryote-specific r-proteins, except eL6, eL28, the P-stalk proteins, eS12, eS31 and RACK1 (Figure 1A). It is known that the orthologs for eL6 and eL28 are missing in *G. intestinalis* (46,47), which we confirm by performing mass spectroscopic analysis (Supplementary Material, PDF Spreadsheet). Further, we have built the entire rRNA except the P stalk and some small parts of LSU in the vicinity of eL13 and uL29. Although the four eukaryote-specific rRNAs are present in *Giardia*, overall rRNA is highly reduced in size compared to yeast and human ribosomes. The rRNA in *Giardia* consists of 4496 nt, which is much shorter than human (7217 nt) or yeast (*Saccharomyces cerevisiae*, 5366 nt) rRNA, but comparable with the 4556 nt long *Escherichia coli* rRNA. Several rRNA ESs are shortened, which can probably be attributed to its parasitic lifestyle (51). *Saccharomyces cerevisiae* has 28 rRNA ESs, while *Giardia* has only 18 ESs, of which 13 are highly reduced (Figure 1B). Although the functional significance of all ribosomal ESs is not known, some of these ESs are associated with distinct functions. For example, ES27 at the bottom of LSU mediates interaction with nascent polypeptide processing factors (48). Also, es6C contacts ribosomal components constituting the mRNA exit and entry sites (66) and acts as a docking surface for factors such as eIF4G involved in translation initiation (66). Both of these ESs are highly reduced in *Giardia* (Figure 1B).

*Giardia* ribosome is significantly depleted of adenines and uracils in the rRNA compared to ribosomes from other species, even the closely related ones from the Metamonada group, such as *T. vaginalis*. As reported previously (47,67), GC content of *Giardia* rRNA is nearly 75% and the GCs are distributed throughout the rRNA (Figure 1C and D). In contrast, human rRNAs have 65% GC, which are localized mostly at the ESs (Supplementary Figure S7). Thus, high GC% of the *Giardia* rRNA could be attributed to stabilization of the overall rRNA architecture compensating for the loss of ESs. However, rRNA of the microsporidium *V. necatrix*—the smallest known eukaryotic ribosome—has a much lower GC content (35%) despite almost no ESs (Supplementary Figure S7). Moreover, not only the matured rRNA, but the spacer regions between the rRNAs in the unprocessed rRNA precursor in *Giardia* are also GC rich (47). Thus, the unique GC-rich property of *Giardia* rRNA may be not only for imparting structural stability to the reduced rRNA, but also for evolutionary pressure due to its parasitic life cycle (68). Alternatively, the high GC content of the *Giardia* rRNA can also be attributed to the location of rRNA genes near the telomere region of *Giardia* chromosomes, which are also known as recombination hotspots leading to GC-biased gene conversion and high GC content (69).

### Functional centers of the *Giardia* ribosome share both bacterial and eukaryotic features

The decoding center (DC) and the peptidyl transferase center (PTC) constitute the main functional centers on the ribosome and are therefore potential targets for small-molecule inhibitors. We analyzed the *Giardia* ribosome structure in the light of high-resolution crystal structures of yeast 80S ribosome (70), *E. coli* 70S ribosome (71) and the archaebacterial 70S ribosome from *Pyrococcus furiosus*. In addition to DC and PTC, differences in the E site and peptide exit tunnel are also discussed.

**Figure 2.**
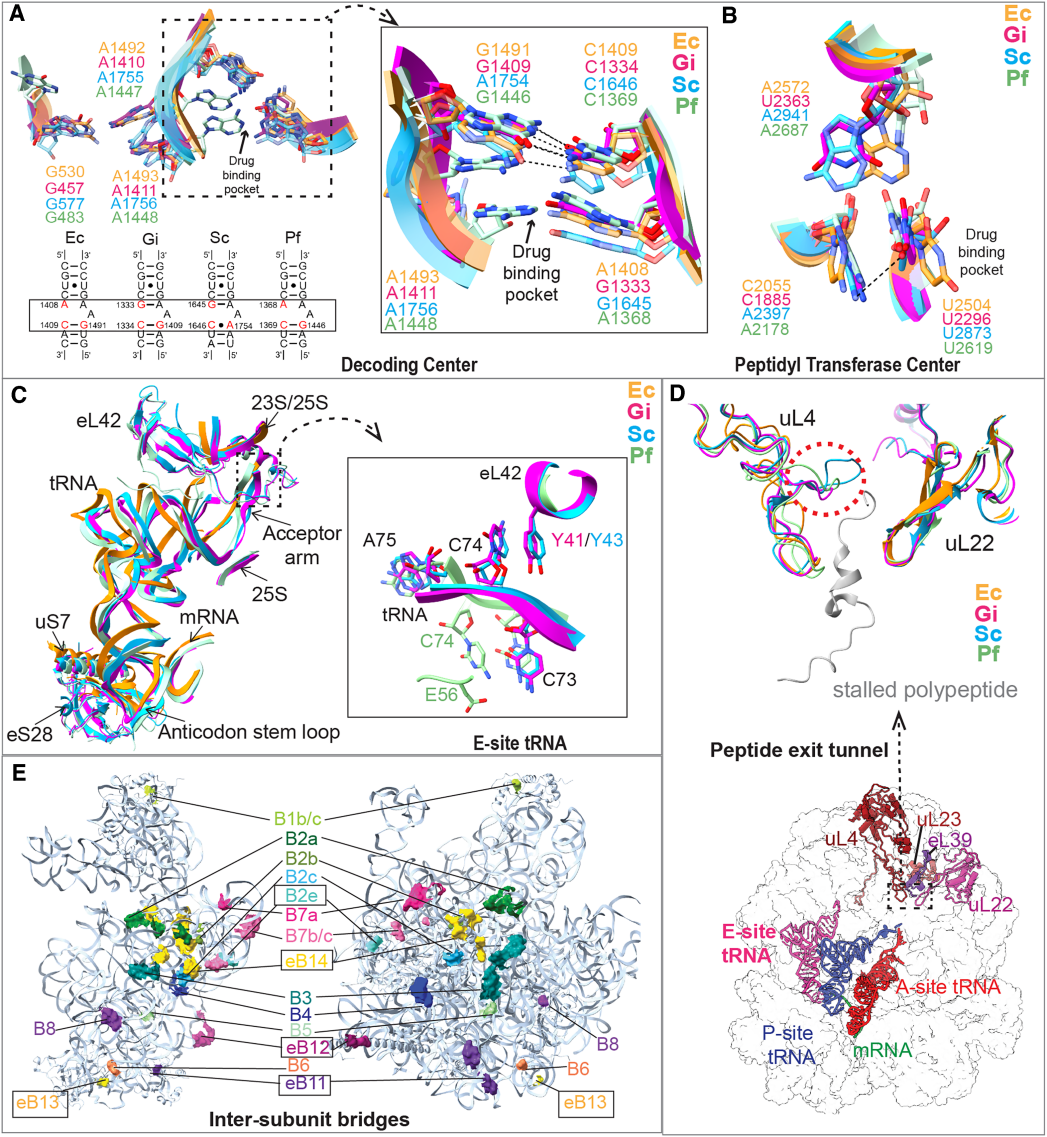
Functional centers of the *Giardia* ribosome display feature characteristic to both bacterial and eukaryotic ribosomes. In panels (A)–(D), the color codes used are orange for *E. coli* (Ec, PDB: 7K00), pink for *G. intestinalis* (Gi), cyan for *S. cerevisiae* (Sc, PDB: 5NDG) and sea green for *P. furiosus* (Pf, PDB:4V6U). (**A**) Comparison of helix 44 secondary structure (bottom panel) between Ec, Gi, Sc and Pf showing the sequence variation at specific positions (in red). These nucleotide changes affect the architecture of the aminoglycoside drug binding pocket at the DC (top panel and zoom), while the three monitoring bases show similar orientation. (**B**) Comparison of the conformation and interactions of the conserved U2504/U2296/U2873/U2619 between Ec, Gi, Sc and Pf ribosomes at PTC. This uracil in Sc and Pf interacts with A2397 and A2178, respectively, which opens up the drug binding pocket at the A site. This interaction is absent in Ec and Gi due to the presence of C2055 in Ec and C1885 in Gi instead of adenine. This changes the potential of binding of the eukaryote-specific A-site inhibitors to the Ec and Gi PTC. (**C**) E-site tRNA interactions in Gi are similar to Sc. The eukaryote-specific protein eL42 stabilizes the CCA end of the E-site tRNA (zoom) in Sc, Pf and Gi, which is absent in Ec. (**D**) Gi uL4 has a shorter loop (similar to Pf, red dotted circle) at the peptide exit tunnel compared to Sc. This loop characteristic to eukaryotic ribosomes creates an extra constriction in the peptide exit tunnel. (**E**) Intersubunit bridges in the Gi ribosome. Regions of SSU and LSU contributing to specific intersubunit bridges are colored identically and labeled accordingly. The eukaryote-specific bridges are indicated (box).

The DC selects the appropriate aminoacyl tRNA based on the mRNA codon placed at the A site (72). The SSU rRNA bases responsible for correct codon–anticodon recognition following binding of the aminoacyl tRNA to the A site are universally conserved (72). These bases, commonly known as ‘monitoring bases’, are A1410, A1411 and G457 in *Giardia*, A1492, A1493 and G530 in *E. coli*, A1755, A1756 and G577 in *S. cerevisiae*, and A1447, A1448 and G483 in *P. furiosus* (Figure 2A). However, differences exist in two important nucleotides in the vicinity of these bases, which vary between the bacteria and eukaryotes and are known for regulating aminoglycoside specificity (70). These are A1408 and G1491 in *E. coli*, and G1645 and A1754 in *S. cerevisiae*, respectively. *Pyrococcus furiosus* 70S ribosome has A1368 and G1446, similar to *E. coli*. Interestingly, both of these bases in *Giardia* SSU rRNA are guanines: G1333 (similar to *S. cerevisiae* G1645) and G1409 (similar to *E. coli* G1491) (Figure 2A).

The PTC of the ribosome is highly conserved and is responsible for peptide bond formation and peptide release. A number of inhibitors such as tiamulin, chloramphenicol, clindamycin, etc. are known to bind to PTC of the bacterial ribosome, at the A-site cleft (73,74). The species specificity of A-site inhibitors is determined by a single nucleotide—U2504 in *E. coli*, U2873 in *S. cerevisiae* and U2619 in *P. furiosus*. The conformation of this nucleotide is regulated by its interaction with two additional nucleotides, which are C2055 and A2572 in *E. coli* (73,74), and A2397 and A2941 in *S. cerevisiae*, respectively. *Pyrococcus furiosus* 70S ribosome has A2178 and A2687 similar to *S. cerevisiae*. On the other hand, in the *Giardia* ribosome, these three nucleotides are U2296, C1885 and U2363, respectively (Figure 2B); C1885 is similar to C2055 in *E. coli*.

In contrast to the DC and PTC, the E site of the *Giardia* ribosome has a prototypical eukaryotic architecture. The E-tRNA binding site is formed by the 25S rRNA and eL42. The penultimate cytosine of CCA end of the tRNA is positioned by stacking and H-bond interactions with Y41 (Y43 in *S. cerevisiae*) of eL42 (Figure 2C). Further, the ASL of the tRNA in *S. cerevisiae* and *Giardia* is stabilized by eS28. Interestingly, in the *P. furiosus* 70S ribosome the role of eL42 Y41-*Giardia* is taken over by an Asp residue (D56), which interacts with the CCA end of the E-site tRNA. This reiterates that the E site of the *Giardia* ribosome has a specific eukaryotic architecture.

The peptide exit tunnel of the ribosome is usually lined by uL4, uL22, uL23 and the 25/23S rRNA of LSU. In eukaryotes, an additional protein eL39 lies adjacent to uL23, which is also present in *Giardia* ribosome (Figure 2D). The peptide exit tunnel is constricted where uL22 and uL4 meet; interestingly, in eukaryotes an additional constriction is formed by an extension of the uL4 loop. A comparison of *Giardia* uL4 with those of *S. cerevisiae* and humans (as well as other protozoan parasites; Supplementary Figure S8) reveals that the eukaryote-specific uL4 loop extension is uniquely missing in *Giardia* ribosome. Thus, although the *Giardia* peptide exit tunnel contains eukaryotic ribosome-specific protein eL39, its tunnel constriction pattern resembles bacterial ribosomes.

Intersubunit bridges in the ribosome allow communication between the LSU and SSU during protein synthesis. Eukaryotic cytoplasmic ribosomes have five additional intersubunit bridges located mostly peripherally (except eB14), which are attributed to the preferential rotated state of the eukaryotic ribosomes (42,66,75). In *Giardia* ribosomes, one of these intersubunit bridges eB8 is missing due to absence of LSU rRNA ES31, while two others eB11 and eB12 are highly reduced compared to *S. cerevisiae* and human ribosomes (Figure 2E). Likewise, we could see the unrotated state of these ribosomes as the predominant class. These comparisons suggest that *Giardia* ribosomes (i) present an amalgam of bacterial and eukaryotic features and (ii) can be sensitive to some bacteria-specific translation inhibitors.

### *In silico* sorting of ribosomal conformations reveals six classes of translocation intermediates of *Giardia* ribosome

**Figure 3.**
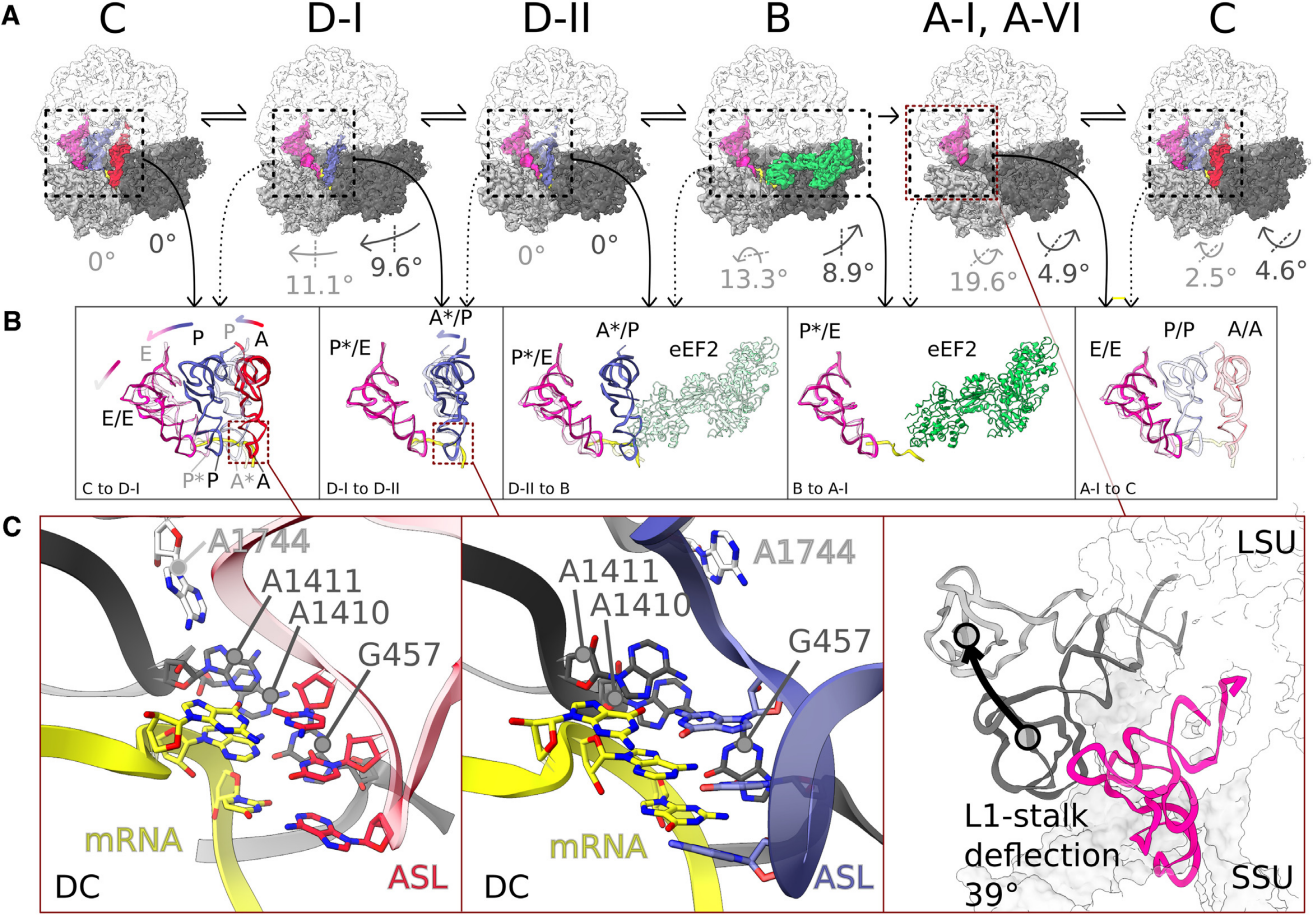
Translocation-intermediate states of the *Giardia* ribosome with naturally trapped tRNAs and eEF2 captured by cryo-EM. (**A**) Top row: Structures of distinct translocational states (LSU, white; SSU body, dark gray; SSU head, light gray; A-site tRNA, red; P-site tRNA, blue; E-site tRNA, pink; mRNA, yellow; eEF2, green) obtained through cryo-EM single-particle reconstruction and arranged according to the sequence in translocation (C, D-I, D-II, A-I, A-IV, B). Degree of rotation in the SSU body (dark gray) and head (light gray) with respect to the previous state is indicated along with the rotation axis (dotted line). (**B**) Middle row: Comparison of the relative positions of A-, P- and E-site tRNAs and eEF2 in different translocation states (marked with solid arrows) with those from the subsequent states (marked with dotted arrows) superimposed with transparent colors. The colored arrows indicate tRNA movement. (**C**) Bottom panels, left/middle: Zoomed image of the DC of the PRE-T states showing monitoring bases sampling the codon–anticodon minor groove and (right) movement of L1 stalk in the POST-T state from class A-I to A-VI. (Note the absence of E/E tRNA for class A-VI.)

Cryo-EM single-particle reconstruction was used to create a hierarchy from the parent class with differing SSU conformations and four subclasses with compositional heterogeneity (Supplementary Figure S1). The resolved ribosomal subclasses (referred to hereafter as states) clearly feature translocation intermediates (Figure 3A). Unrotated state C (structure C-III) and rotated/ratcheted state D (structures D-I and D-II) represent PRE-T substrates differing in their SSU and tRNA conformations (Figure 3A). State C contains tRNAs in the classical A (A/A), P (P/P) and E (E/E) states (Figure 3A and B) similar to previous bacterial or eukaryotic classical PRE-T complexes (43,76,77). The tRNAs are stabilized through canonical interactions from the A, P or E sites. The acceptor arm of the A-site tRNA is at the PTC (Figure 3A and B). Clear density is seen for mRNA with the monitoring bases A1410 and A1411 sampling the minor groove between the codon and anticodon of the A-site tRNA (Supplementary Figure S4). The third monitoring base G457 and A1744 of helix 69 (A1913 in *E. coli* and A2256 in *S. cerevisiae*) were seen interacting with the codon–anticodon pairs (Figure 3C).

The D states show relative SSU body rotation of 9.6° and SSU head rotation of 11.1° with respect to state C (Figure 3A and Supplementary Table S3). This is also evident from rRNA residue displacement analysis focusing on SSU head and body segments (Supplementary Figure S5). States D-I and D-II feature rotated ribosomes, each with two tRNAs in hybrid states (Figure 3A and B). Interestingly, these structures contain a tRNA in the A*/P state, such that its ASL moves toward the SSU P site by ∼10 Å compared to the tRNA in the A state in C (Figure 3B). This movement breaks the anticodon stem interaction with H69 nucleotide A1744, but the interactions with the monitoring bases A1410 and A1411 as well as G457 are retained (Figure 3C). Helix 69 is pulled away during transition from D-I to D-II through interaction with SSU h44 (C1334) (Figure 3C, middle panel, and Supplementary Figure S9). Further, the elbow of the A*/P tRNA also shows deflection in transition from the D-I to D-II states (Supplementary Figure S10). The A*/P tRNA elbow in D-I interacts with 28S rRNA nucleotides 731–33 (H38) and uL16 D28 residue, which are not seen in D-II. Contrarily, the acceptor arm in D-II is stabilized by several interactions from 28S rRNA and uL16. Structures C, D-I and D-II appear to trace the path of the A-site tRNA toward the P site (Figure 3B). The second tRNA in D-I and D-II states is in the P*/E state such that its ASL is intermediate between the P and E sites of the SSU (∼14 Å from the SSU P site), the elbow is yet to approach E site (8.8 Å away from E-site tRNA elbow of state C), while the acceptor arm and CCA end are stabilized by E-site-specific interactions (see the ‘Functional centers of the *Giardia* ribosome share both bacterial and eukaryotic features’ section and Figure 2C).

State B represents an eEF2-bound transient translocation intermediate in which eEF2 is bound to GDP and the leaving Pi (Figures 3A and B, and 5A and B). The SSU body in B is slightly rotated: 1.05° compared to state C, but corresponding to a back-rotation of 8.9° from the D states. However, the SSU head is still highly swiveled: 17.5° compared to state C, and 13.3° on an orthogonal rotation axis compared to the D states (Figure 3A, Supplementary Table S3 and Supplementary Figure S5). eEF2 domain IV is inserted between the SSU A and P sites (∼10 Å from the P site) (Figures 3B and 5C). We observe clear density for the P*/E tRNA. However, there is no density corresponding to the A/P tRNA similar to previous studies (20,78,79), which indicates lower stability of the A/P tRNA during the dynamic state of translocation by eEF2. Moreover, we find the mRNA somewhat dislodged from its usual position at the A site. This can potentially be due to the loss of the A/P tRNA, which would otherwise stabilize the mRNA by codon–anticodon interaction while interacting with A1133 and C1016 on h34 (A1427 and C1274 in *S. cerevisiae*) with the 5′ end, as seen in the C and D states.

State A represents an ensemble of ribosomal POST-T states with a strong density for the E-site tRNA with occupancy of ∼91%. The most populated state (A-I) shows a completely closed L1-stalk and E-site tRNA, while the other substates (II through VI) show snapshots of a continuum of movement of the L1 stalk with the final state (A-VI) having no bound tRNAs and an L1-stalk angle of 39° (Figure 3C, right panel). The stable binding of the E-site tRNA is ensured through interactions of the CCA end of the tRNA to 28S and eL42, similar to the POST-T state structures of *S. cerevisiae* ribosome (80,81). Compared to state B, the SSU head in state A occupies a conformation having undergone a 19.6° reverse swivel (Figure 3A). With this, the SSU head achieves a conformation quite similar to state C (with a minor 2.5° deflection). Interestingly, the A state(s) exhibit an additional rotation of the SSU with a different rotational axis, which is similar to previously reported eukaryote-specific ‘subunit rolling’ (Supplementary Movie A).

### *Giardia* ribosome demonstrates eukaryote-specific subunit rolling but with insignificant widening of the E site

**Figure 4.**
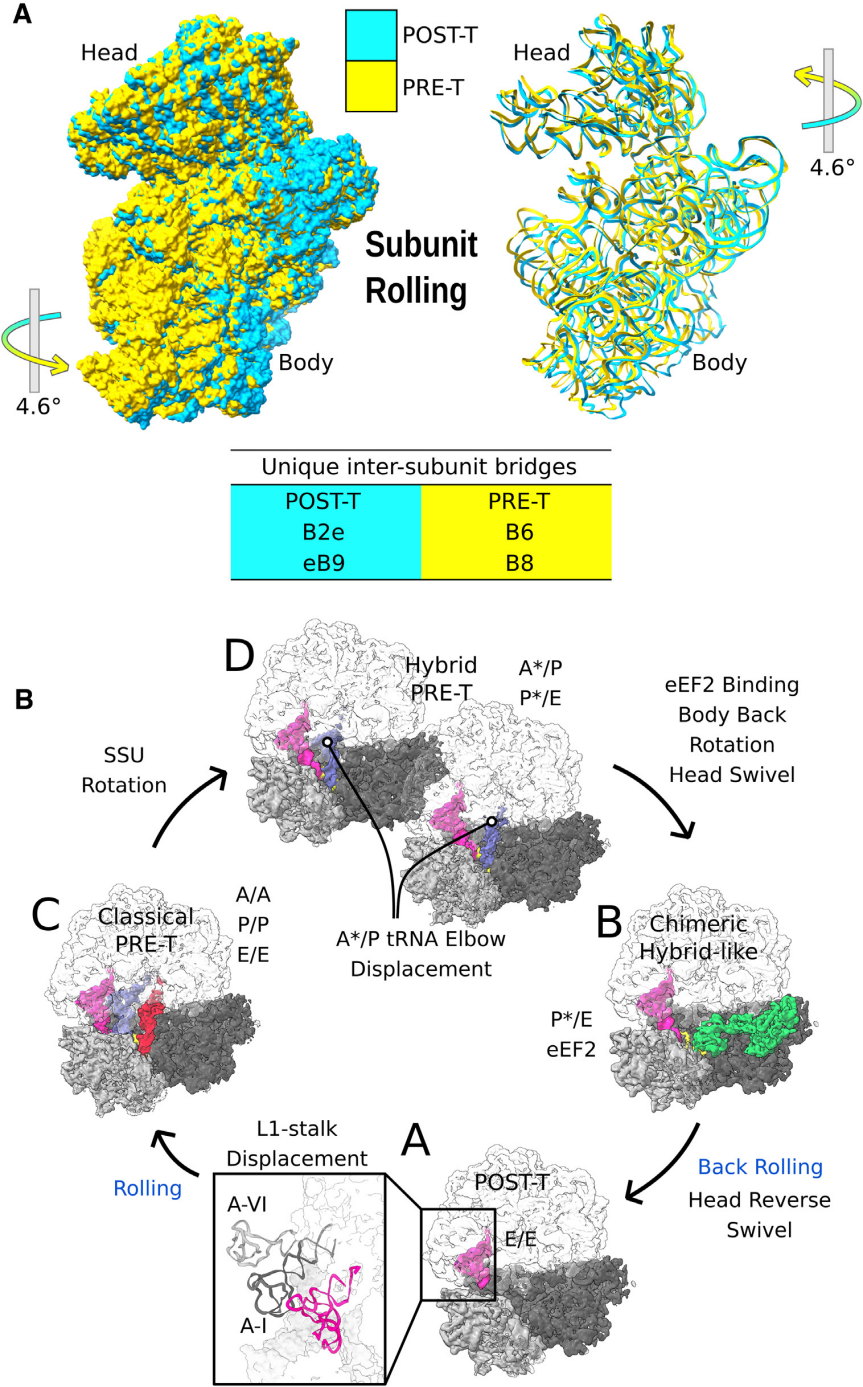
*Giardia* ribosomes exhibit eukaryotic ribosome-specific SSU rolling. (**A**) Comparison of SSU between the POST-T state (yellow) and classical PRE-T state (cyan) in RNA ribbon structures (left) and surface view (right). Ribosomes in these two states are aligned based on LSU. The arrows indicate the direction of movement with the angle specified. The intersubunit bridges unique to the two states are listed below. (**B**) The SSU motions during the *Giardia* elongation cycle. The conventional SSU rotation is seen when the classical PRE-T state (state C) changes to the hybrid PRE-T state (states D, D-I and D-II, respectively). eEF2 binds to the hybrid PRE-T state to trigger a chimeric hybrid state (state B) in which the SSU body back-rotates and simultaneously SSU head swivels. The SSU head back-swivels after/during eEF2 dissociation and thereby the ribosome reaches the POST-T state (state A). The SSU rolling (marked in blue) takes place during the transition from the POST-T state to the PRE-T state, whereas back-rolling occurs in the last step of translocation (state B → state A) together with reverse swivel of the SSU head (Supplementary Movies A and B).

In eukaryotes, accommodation of aminoacyl tRNA involves a unique ‘rolling’ motion, where the SSU rotates by ∼5^o^ toward the A site with a rotational axis described as ‘orthogonal’ to the axis of rotation for subunit ratcheting (43). The rolling movement narrows down the A site by ∼5 Å (7,44). This is associated with concomitant widening of the E site, which potentially facilitates tRNA dissociation from the E site (7,42–45). This motion also leads to remodeling of intersubunit bridges; B6 and B8 appear in the classical PRE-T state (Figure 4 and Supplementary Figure S11), which are retained in the hybrid PRE-T state. During translocation, the ribosomal subunits undergo ‘back-rolling’ leading to loss of B6 and B8 bridges and appearance of new bridges eB8, eB8b, B2e and eB9 in the POST-T state. These bridges being in the vicinity constrict the E site (44). Subunit rolling is absent in bacterial elongation, where the POST-T and the classical PRE-T states show essentially the same ribosomal conformation. To the best of our knowledge, subunit rolling has not yet been reported in lower eukaryotes.

To understand whether *Giardia* ribosomes exhibit subunit rolling, we compared the classical PRE-T state ‘C’ with the POST-T state ‘A-I’. A clear 4.6° rotation of the SSU toward the A site could be observed in transition from the POST-T to the PRE-T state, along with formation of the B6 and B8 bridges (Figure 4, Supplementary Figures S4 and S10, and Supplementary Table S3). This matched well with the previous reports of subunit rolling in eukaryotic ribosomes (7,43,44). We further identified the axis for this rolling movement by decomposition of the transformation matrix into the axis–angle form (Supplementary Figure S5). Our analysis shows that the axis for subunit ‘rolling’ (state A → state C) in *Giardia* ribosome is not orthogonal (90°) to the axis of subunit rotation (state C → state D), but inclined at an angle of 32° with respect to each other (Supplementary Figure S5 and Supplementary Movie A).

Next, we looked for changes in the size of the A site (calculated by the distance between G2440 of 25S and A330 of 18S) and the E site (distance between G2217 of 25S and U667 of 18S). We observed ‘subunit rolling’-specific narrowing of the A site by ∼6 Å in the PRE-T state, similar to the earlier report in human ribosomes (∼5 Å) (44). However, the widening of the E site was negligible (∼1 Å), which is clearly different from the subunit rolling in the human ribosome where ∼5 Å widening of the E site in the PRE-T state was reported (44). Moreover, *Giardia* ribosomes lack bridges eB8 and eB8b due to absence of corresponding rRNA ESs. These bridges are seen in the human POST-T state, in addition to bridges B2e and eB9 that are also present in *Giardia* (Figure 4 and Supplementary Figure S11). Since the eB8 and eB8b bridges (absent in *Giardia*) are close to the E site, the absence of these two bridges in the POST-T state is likely to be the main reason for the negligible size change in the E site during subunit rolling. Thus, *Giardia* ribosome demonstrates an intermediate ‘subunit rolling’ in its elongation cycle with a distinct difference of insignificant widening of the E site compared to higher eukaryotes.

### The eEF2·GDP-bound state elucidates the mechanism of Pi release

**Figure 5.**
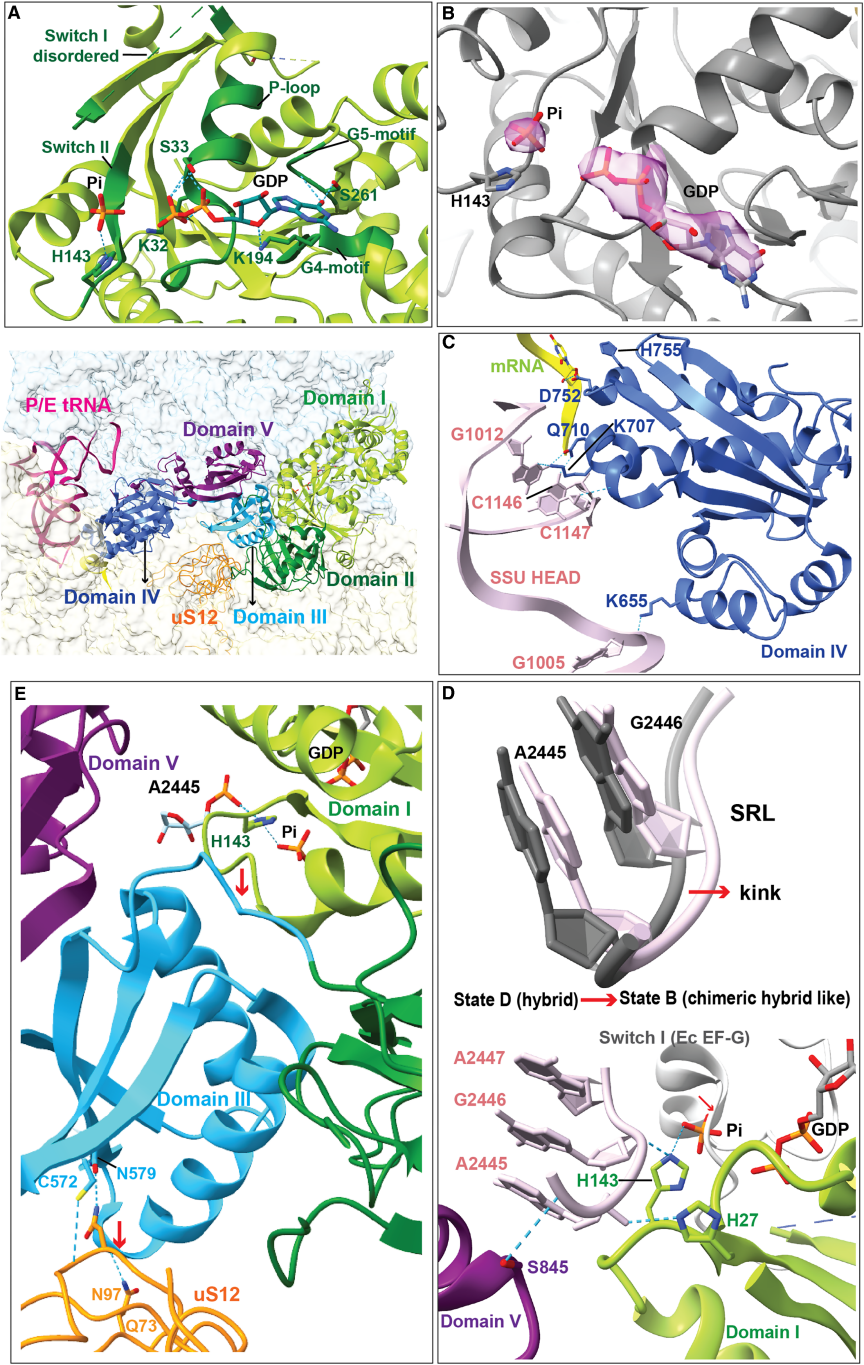
Overview of *Giardia* eEF2 interaction with the ribosome in the chimeric hybrid state. (**A**) The G domain of eEF2 with GDP and a leaving Pi stabilized through interactions from P-loop, Sw-II, G4 and G5 motifs (dark green). (**B**) Cryo-EM density of GDP and Pi (threshold: RMSD 4.55) showing that the Pi is a distinct noncovalently coordinated entity. (**C**) Interactions of eEF2 domain IV (blue) with SSU head (light pink) and mRNA (yellow). (**D**) A kinked conformation of SRL induced between the hybrid (gray) and chimeric hybrid (light pink) states of *Giardia* ribosomes (top). The kinked conformation is induced through interactions with domain I (green) and domain V (purple) of eEF2 (bottom). A compact Sw-I (gray) from *E. coli* (Ec) EF-G bound to GDP + Pi on the ribosome (PDB: 7PJV) is displayed. The structures are aligned on the LSU for comparison, which indicates that Sw-I needs to be disordered for Pi release. Interactions of His143 in Sw-II with the leaving Pi and SRL, and the P-loop His27 with the SRL are shown. (**E**) Interactions of eEF2 domain III with uS12 on one hand and the Sw-II loop of domain I (G domain) on the other hand are illustrated.

State B of the *Giardia* ribosome presents eEF2·GDP and a leaving Pi. *Giardia* eEF2 resembles eEF2/EF-G orthologues in both overall domain architecture and binding to the ribosome. It consists of five distinct domains (I–V) wherein domain I is the GTPase domain or G domain (Figure 5A and B) and domain IV is crucial for translocation function (Figure 5C). We found a clear density of GDP in the G domain of eEF2, which is coordinated by interactions from P-loop (G1), G4 and G5 motifs, similar to other reported EF-G/eEF2 structures (25,26,41,45) (Figure 5A and B). Switch-I (Sw-I) is disordered, which is usual to the off-state (Figure 5A). The catalytic histidine (H143) in switch-II (Sw-II) interacts with the phosphate backbone of A2447 (A2662 in *E. coli*) in SRL (Figure 5D). The SRL conformation, induced by interaction of the domain V residue S845 with A2445, closely resembles the twisted conformation of the SRL observed in the EF-G·GDP + Pi structure on *E. coli* ribosomes (Figure 5D) (25). A distinct density of the ‘leaving Pi’ group can be seen in the path of the expected trajectory for Pi release (82) held by the interaction with H143 (Figure 5B). Superimposition of state B with *E. coli* 70S in the EF-G·GDP + Pi state suggests that Pi release requires Sw-I to be disordered to avoid a steric clash (Figure 5D). One interesting observation is that the Pi moiety in this structure is placed 4.7 Å away from the phosphate of the recently reported GDP + Pi cryo-EM structure of *E. coli* ribosome (25) (Supplementary Figure S12). This indicates that we have trapped a mimic of the post-GTP hydrolysis eEF2 intermediate on the ribosome closer to the completion of Pi release.

In our structure, domain IV of eEF2 is extensively stabilized through interactions with the SSU head and H69 of 25S rRNA, in the absence of A/P tRNA (Figure 5C). Further, domain III of eEF2 interacts extensively with uS12 via three interactions between C572, K549 and N579 of eEF2 with N96, N97 and S78 of uS12. In an early translocation-intermediate structure of *S. cerevisiae*, eEF2 domain III-N499 interacts with K82 of uS12. In this structure, the SSU body is in the rotated state. However, in three late translocation-intermediate states of mammalian 80S (Tl-POST-1, Tl-POST-2) (45) where the SSU body has back-ratcheted, eEF2 domain III does not interact with uS12. This indicates that interaction of domain III of eEF2 and uS12 is lost during SSU back-rotation. Most probably, SSU body back-rotation after GTP hydrolysis pulls down domain III of EF-2 via uS12, which in turn pulls back Sw-II guiding the Pi release (Figure 5B and C).

## DISCUSSION

In recent years, our knowledge about the steps of translation has progressed significantly due to the technical advances in cryo-EM. Our observations of translocation intermediates of the native ribosomes of *G. intestinalis* add insightful details regarding ribosomal translocation. We discuss below the main observations in the light of translocation mechanism, drug sensitivity and evolution of translational machinery.

### Implications of *Giardia*-specific ribosomal features in antibiotic binding/resistance

The ribosomes of *G. intestinalis* are highly compact with significantly reduced ES and with uniquely high GC content. While it contains all characteristic eukaryotic rRNAs and most of the eukaryotic r-proteins, a closer look at the functional centers lifts up the bacterial ribosome-specific features, which enable us to reflect on the prospects of drug binding.

The DC of the ribosome is usually targeted by aminoglycoside antibiotics, which bind to h44 of 18S rRNA at the DC and flip the monitoring bases, allowing near- or noncognate tRNAs to be accepted, causing errors in translation (60). Aminoglycoside specificity in *E. coli* is regulated by the nucleotides A1408 and G1491, which are replaced with G1645 and A1754 in *S. cerevisiae*. G1645 occludes aminoglycosides with 6′-substitution in ring I (e.g. neomycin with amino group substitution) except those with hydroxyl group substitution (e.g. geneticin and paromomycin). Similarly, A1754 prohibits aminoglycosides with 5″-hydroxyl group in ring III, e.g. neomycin and paromomycin from binding (70). Since the *Giardia* ribosome has G1409 similar to G1491 in *E. coli* instead of A1754 of *S. cerevisiae*, we speculate that paromomycin could bind to the DC (Supplementary Figure S13A). This agrees with reports from earlier clinical studies (83,84). Paromomycin was also used to treat giardiasis in pregnant women (67). To validate our hypothesis, we have tested percent survival of *Giardia* in the presence of paromomycin. Our data show that the effectivity of paromomycin to limit *Giardia* survival is in similar range as the predominantly used *Giardia* drug metronidazole (Supplementary Figure S14). Another aminoglycoside antibiotic geneticin exhibits high affinity for *S. cerevisiae* ribosomes due to its interaction with the eukaryote-specific bases (G1645 and A1754) (70) (Supplementary Figure S13A). A previous biochemical study demonstrates that *S. cerevisiae* ribosomes with A1754G mutation are ∼60-fold more sensitive to paromomycin and 10-fold more sensitive to G418 (geneticin) (85). Since *Giardia* ribosomes resemble the above *S. cerevisiae* mutant scenario, it is possible that geneticin may also inhibit protein synthesis in *Giardia*. Interestingly, ribosomes of some other protozoan parasites such as *P. falciparum*, *T. gondii* and *T. vaginalis* possess two guanine nucleotides corresponding to G1333 and G1409 of *Giardia*. Thus, it is tempting to speculate that aminoglycosides with a ring-I 6′-hydroxyl group might inhibit these ribosomes as well.

In *S. cerevisiae*, A2397 interacts with U2873 to accommodate the eukaryote-specific PTC inhibitors (e.g. T2 toxin; Supplementary Figure S13B) (70). In many protozoan parasites, including *T. vaginalis*, closely related to *G*. intestinalis, there is an adenine in the position corresponding to A2397 in *S. cerevisiae*. However, *Giardia* ribosomes possess C1885 (instead of A) similar to C2055 in *E. coli*. Hence, we suspect that the bacteria-specific A-site inhibitors, e.g. clindamycin and chloramphenicol, might inhibit *Giardia* ribosomes (Figure 2B and Supplementary Figure S13B). However, it is known that A2572U mutation leads to tiamulin resistance in *Brachyspira hyodysenteriae* (86). *Giardia* and *T. vaginalis* ribosomes have uracil (U2363 in *Giardia*) in place of A2572 in *E. coli*, which suggests that *Giardia* and *T. vaginalis* ribosomes might be resistant to tiamulin.

Selectivity of E-site inhibitors toward eukaryotes is regulated by two bacteria-specific rRNA residues, A2432 and U2431, in *E. coli* (70). These residues occlude inhibitor binding. In *Giardia* and *S. cerevisiae*, there are two adenines in these positions. Thus, cycloheximide and lactimidomycin, which are E-site inhibitors of the eukaryotic ribosome, should potentially bind and inhibit *Giardia* ribosomes. We have tested percent survival of *Giardia* trophozoites in the presence of cycloheximide and lactimidomycin. Cycloheximide efficiently kills the trophozoites at a drug concentration significantly lower than metronidazole, while lactimidomycin roughly matches metronidazole (Supplementary Figure S14) supporting our structural analysis.

### Translocation intermediates of the *Giardia* ribosomes display both subunit rotation and rolling, albeit to a limited extent

Translocation intermediates for structural studies are often formed through *in vitro* assembled complexes, either with nonhydrolyzable guanine nucleotide analogs (GMPPCP, GDPCP, etc.) or with antibiotics trapping eEF2 on the ribosome (45,87). These states are therefore not completely natural. We have isolated translating ribosomes directly from the log-phase culture of *G. intestinalis*, containing an ensemble of various translation intermediate states. Further through *in silico* sorting, we could resolve the heterogeneity in the ribosome sample and obtain six distinct translational states at near-atomic resolution. These states represent authentic, native elongation cycle intermediates in which rotation of the SSU relative to the LSU, eEF2 binding, swiveling motion of the SSU head relative to the body and subunit rolling motion could be observed in conjunction with displacement of tRNA from the A to P site and from the P to E site. The strength of our approach is portrayed by the fact that we could assign 94.5% of the particles (Supplementary Table S1) to distinct ribosomal states, which are named A–D (Supplementary Figure S1).

These distinct ribosomal states represent intermediates of the *Giardia* tRNA translocation cycle. State C represents the nonrotated, PRE-T ribosome in which tRNAs are in the A/A, P/P and A/A, P/P, E/E classical states, respectively. Ribosomes are reported to undergo spontaneous intersubunit rotation (also called ratcheting) even in the absence of EF-G/eEF2, oscillating between classical and hybrid states (88). Structures D-I and D-II represent these hybrid conformations in which SSU rotation causes tRNAs to acquire the A*/P and P*/E states. These states constitute proper substrate for eEF2·GTP binding, shifting the equilibrium toward the hybrid conformation. Structure B is a hitherto unknown eEF2-bound chimeric hybrid-like intermediate, where eEF2 is in the GDP state with a distinct density for the leaving Pi held by the catalytic H143 from Sw-II. Comparison of the D states with state B shows that the A*/P tRNA ASL must translocate to the P site to accommodate domain IV of eEF2 in the A site (Supplementary Movie B). Further analysis reveals that state B represents an ‘early’ late intermediate state, with the SSU body showing a lesser degree of back-rotation than the previously reported late intermediate states (45). Reverse swivel of the SSU head marks completion of translocation, leading to the final POST-T state, represented by state A of *Giardia* ribosomes.

As mentioned earlier, eukaryotic tRNA translocation is associated with an additional rotational motion of the SSU termed ‘subunit rolling’ (42–45). This motion leads to A-site narrowing and E-site widening during transition from the POST-T to the PRE-T state. These reciprocal changes lead to aminoacyl tRNA accommodation at the A site and deacylated tRNA release from the E site. In *Giardia*, subunit rolling is observed, but the extent of E-site widening is insignificant. Overall, these structures are the first snapshots of actively translating ribosomes from a parasitic protozoan elucidating tRNA translocation (Supplementary Movies A and B).

### Mechanism of Pi and eEF2 release marks the completion of elongation cycle

**Figure 6.**
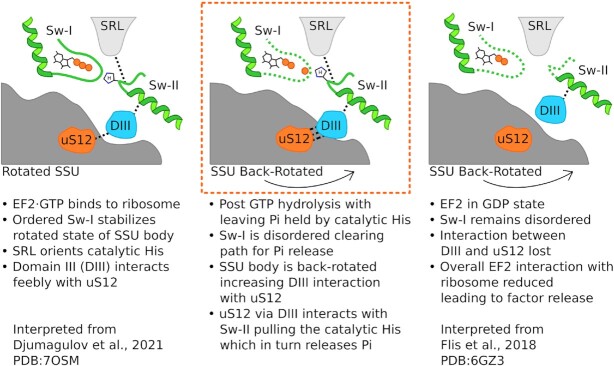
Mechanism of eEF2 and Pi release from the ribosome. Left: When eEF2–GTP binds to the ribosome, its ordered Sw-I stabilizes the rotated SSU body. SRL orients the catalytic His on Sw-II toward GTP and domain III interacts weakly with uS12 (interpreted from PDB: 7OSM). Middle: Our state B structure shows that post-GTP hydrolysis, where Sw-I becomes disordered clearing the path for Pi release. SSU body back-rotates, thereby resulting in increased interaction between domain III and uS12. This enhanced interaction pulls Sw-II linked to domain III and the catalytic His loses interaction with Pi, which releases Pi (right) (interpreted from PDB: 6GZ3).

State B resembles a translocation intermediate with eEF2 bound to GDP and the leaving Pi in a unique position. Comparison of our structure with the EF-G/eEF2-bound early translocation intermediates from *E. coli* (GDP + Pi) (25) and *S. cerevisiae* (GDPCP) (41), and late translocation intermediates from mammals (GMPPNP) (45) reveals an interesting aspect (summarized in Figure 6).

*Escherichia coli* EF-G in the GDP + Pi state has been seen as bound to the ribosome in a fully rotated state (PDB: 7PJV) (25). Sw-I of EF-G is ordered, which by interaction with SSU body stabilizes the rotated state of the ribosome. In comparison, the SSU body appears slightly back-rotated in the eEF-2·GDPCP-bound early translocation-intermediate structure of *S. cerevisiae* ribosome (41), which causes loss of interaction of the SSU body with Sw-I. The back-rotation of the SSU body brings eEF2 domain III at interacting distance to uS12. Domain III interacts with Sw-II in all available EF-G/eEF2 structures. As the SSU body back-rotates further (our structure), interaction of domain III with uS12 enhances, which pulls Sw-II. In the late translocation intermediates (PDB: 6GZ4), the domain III–uS12 interaction is lost. From these comparisons, the order of events appears as follows: (i) EF-G/eEF2 binds to a fully rotated ribosome in the GTP state stabilized by an ordered Sw-I; (ii) GTP hydrolysis disorders Sw-I, initiates SSU body back-rotation and strengthens domain III interaction with uS12; and (iii) domain III interacts with Sw-II, which pulls the catalytic His to a catalytically incompetent state. In this process, the Pi from GTP hydrolysis that is held by the catalytic His is released (Figure 6).

We conclude that post-GTP hydrolysis, SSU body back-rotation pulls down domain III of EF-2 via uS12, which in turn pulls back Sw-II with the catalytic His, thereby guiding the Pi release. It is plausible that we managed to trap this naturally populated state due to the interactions of EF-2 domain IV with SSU head, which acts as a ‘door-stop’ and prevents further SSU back-ratcheting and loss of interactions with uS12.

### Evolutionary perspective of the *Giardia* ribosome

The proteins and RNAs that constitute a bacterial ribosome form the conserved core of the eukaryotic ribosome. Additional eukaryote-specific r-proteins and rRNA ESs add to the complexity of this core. *Giardia* is a protozoan parasite that diverged early from the eukaryotic branch (89). Although its rRNA is highly compact as in bacteria, and the ESs are highly reduced, it contains almost all of the eukaryote-specific r-proteins, except eL6 and eL28. In the DC and PTC, *Giardia* ribosomes possess nucleotides similar to either bacteria or eukaryotes (Supplementary Table S4). When compared for E-site interactions with the tRNA, *Giardia* ribosome shows intermediate number of interactions compared to bacteria and eukaryotes. Similar trend is also observed for intersubunit bridges, while in the peptide exit tunnel, it possesses bacteria-like single constriction instead of two constrictions in eukaryotic ribosomes. Not only the structural features, but also the mechanism of translocation shows intermediate conformational dynamics in *Giardia* ribosomes. While subunit rolling is absent in bacteria, *Giardia* ribosomes undergo rolling, albeit to a lesser extent than other eukaryotes. Thus, the *Giardia* ribosome shares both bacterial and eukaryotic features (Supplementary Table S4), which justifies its intermediate position in the evolutionary tree (89).

The length of the rRNA ESs has increased significantly in the course of evolution from the protozoan to the metazoan eukaryotes. These ESs have been proposed to be associated with ribosome biogenesis (90), translation initiation (78) and fidelity (57). ES3 and ES6 in the SSU interact with the eukaryote-specific translation initiation factors eIF3 and eIF4G. Interestingly, compared to higher eukaryotes, the *Giardia* ribosome lacks both ES3 and ES6, and likewise the eIF4G ortholog and several eIF3 subunits. Thus, the lack of ESs and other typical eukaryotic features in the *Giardia* ribosome may reflect its evolutionary lineage as an early branching member of the eukaryotic domain. Alternatively, the loss of the ESs could have been attributed to an adaptive evolution for its parasitic life cycle.

Comparison of the *Giardia* ribosome with other eukaryotic ribosomes provides insights about evolution of the eukaryotic ribosomes. As mentioned above, *Giardia* ribosomes lack multiple ESs and the eukaryotic r-proteins eL6 and eL28. Interestingly, the *V. necatrix* ribosomes, the smallest eukaryotic ribosome with minimal ESs, also lack three r-proteins, eL41, eL38 and eL28. These observations suggest that most of the eukaryote-specific r-proteins might have evolved prior to rRNA ESs; hence, the conserved bacterial rRNA core is sufficient to support these additional proteins through enhanced protein–protein interactions (51). However, some eukaryotic r-proteins might also have coevolved with the rRNA ESs. The r-proteins such as eL6, eL28 and eL14 bind to the region ES7–ES15–ES39 and likely stabilize these ESs. In *V. necatrix*, where all the three ESs are missing, eL14 is devoid of most of its secondary structure. Thus, it is plausible that these r-proteins have coevolved with ESs 7 and 15, as also suggested previously (48). Interestingly, ES7 is missing in *G. intestinalis*, *V. necatrix* and *T. vaginalis* (Supplementary Figure S15). In contrast, *T. brucei* has two helical branches in ES7 and the higher eukaryotes, *T. gondii* and *P. falciparum* have three helices in ES7 wherein two branches are much longer than in *S. cerevisiae*. Metazoans such as *Homo sapiens* and *Drosophila melanogaster* have ES7 with multiple helical branches (Supplementary Figure S15). Thus, it is fair to comment that ES complexity in the ribosomes has increased progressively during the course of evolution, which correlates well with the separation times of the species (Supplementary Figure S15). Combining our observations with earlier reports, it can be suggested that evolution of eukaryotic ribosome from the conserved bacterial ribosomal core has occurred in multiple stages through increase in (i) protein–protein interaction as seen in both the *V. necatrix* and *G. intestinalis* ribosomes, (ii) protein–RNA interaction, e.g. *S. cerevisiae*, and (iii) RNA–RNA interaction, e.g. *H. sapiens*.

## DATA AVAILABILITY

The cryo-EM maps and atomic coordinates generated in this study have been deposited in the Electron Microscopy Data Bank and Protein Data Bank (see Supplementary Table S2 for accession codes). The code used in this study is available at https://github.com/andy-emmo/ribosome-building-analysis and https://doi.org/10.5281/zenodo.7670234.

## Supplementary Material

gkad176_Supplemental_Files
