# Supplementary material for: Insights into translocation mechanism and ribosome evolution from cryo-EM structures of translocation intermediates of Giardia intestinalis
Source: Nucleic Acids Res. 2023 Mar 13;51(7):3436–51. doi: 10.1093/nar/gkad176 (PMC10123126; doi:10.1093/nar/gkad176)
Supplement: gkad176_Supplemental_Files [file gkad176_supplemental_files.zip › Giardia ribosome Mass Spec Spreadsheet.pdf]

Accession	Description	Score	Coverage	# Proteins	# Unique Peptides	# Peptides
A8BRZ3	Ribosomal protein L3 OS=Giardia intestinalis (strain ATCC 50803 / WB clo	1778.58	70.45	4	28	28
V6TNC8	Heat shock protein 70 OS=Giardia intestinalis OX=5741 GN=DHA2_88765	1353.03	59.79	5	33	33
A0A644F983	Axoneme-associated protein GASP-180 OS=Giardia intestinalis (strain ATC	1051.52	70.87	9	9	106
Q962Q0	Axoneme-associated protein GASP-180 OS=Giardia intestinalis OX=5741 P	1048.84	70.87	4	8	106
A8B7H8	Ribosomal protein L4 OS=Giardia intestinalis (strain ATCC 50803 / WB clo	917.95	73.42	4	23	23
A8BCP0	Ribosomal protein P2 OS=Giardia intestinalis (strain ATCC 50803 / WB clo	824.40	67.21	4	8	8
E2RU04	Dynamin OS=Giardia intestinalis (strain ATCC 50803 / WB clone C6) OX=:	803.35	59.29	3	38	38
A8BFE3	40S ribosomal protein S4 OS=Giardia intestinalis (strain ATCC 50803 / WB	766.90	68.28	3	9	18
V6TAM8	60S acidic ribosomal protein P0 OS=Giardia intestinalis OX=5741 GN=DHA	705.11	52.15	1	9	19
V6TSQ3	40S ribosomal protein SA OS=Giardia intestinalis OX=5741 GN=GSB_7766	692.61	51.64	4	11	11
A0A644F9A2	Ribosomal protein S3a OS=Giardia intestinalis (strain ATCC 50803 / WB cl	666.38	59.27	3	3	15
V6TJ44	SSU ribosomal protein S4P OS=Giardia intestinalis OX=5741 GN=DHA2_4	649.92	57.67	1	2	20
A8BZ58	Ribosomal protein S18 OS=Giardia intestinalis (strain ATCC 50803 / WB cl	621.81	67.53	5	12	12
E1EWX6	Ribosomal protein S9 OS=Giardia intestinalis (strain P15) OX=658858 GN=	613.46	57.67	2	1	19
V6TI59	40S ribosomal protein S8 OS=Giardia intestinalis OX=5741 GN=DHA2_584	611.94	60.92	3	10	10
A8BMZ1	Ribosomal protein S2 OS=Giardia intestinalis (strain ATCC 50803 / WB clo	523.39	70.66	6	19	19
V6TEG8	LSU ribosomal protein L23P OS=Giardia intestinalis OX=5741 GN=DHA2_	466.75	75.18	3	16	16
A0A644FB74	Ribosomal protein L10a OS=Giardia intestinalis (strain ATCC 50803 / WB c	461.70	61.75	4	12	12
A8BEW2	Uncharacterized protein OS=Giardia intestinalis (strain ATCC 50803 / WB	438.52	42.28	2	11	66
V6T7M2	SSU ribosomal protein S3P OS=Giardia intestinalis OX=5741 GN=DHA2_7	427.51	60.37	2	20	20
A8BRN9	Uncharacterized protein OS=Giardia intestinalis (strain ATCC 50803 / WB	421.65	64.24	4	11	11
E1EX75	Ribosomal protein L19 OS=Giardia intestinalis (strain P15) OX=658858 GN	416.48	40.82	3	11	11
A8B8F8	Ribosomal protein L12 OS=Giardia intestinalis (strain ATCC 50803 / WB cl	411.36	75.27	5	12	12
V6TBX0	Protein Translation Elongation factor 2 (EF-2) OS=Giardia intestinalis OX=	410.43	45.03	5	37	37
A8BTP1	C4 group specific protein OS=Giardia intestinalis (strain ATCC 50803 / WB	394.94	48.99	10	9	9
V6TFW6	40S ribosomal protein S21 OS=Giardia intestinalis OX=5741 GN=DHA2_70	380.49	70.79	2	8	8
E1EVJ1	Axoneme-associated protein GASP-180 OS=Giardia intestinalis (strain P15	377.10	28.70	3	1	43
A8BZD3	Ribosomal protein S5 OS=Giardia intestinalis (strain ATCC 50803 / WB clo	375.36	58.42	5	13	13
A8BS96	Ribosomal protein S23 OS=Giardia intestinalis (strain ATCC 50803 / WB cl	366.84	47.55	5	8	8
A8B4F8	Ribosomal protein L14 OS=Giardia intestinalis (strain ATCC 50803 / WB cl	363.02	76.34	4	11	11
E1EZE4	60S ribosomal protein L7a OS=Giardia intestinalis (strain P15) OX=65885	347.48	64.13	19	15	15
V6TFA0	LSU ribosomal protein L7 OS=Giardia intestinalis OX=5741 GN=DHA2_19	344.40	57.87	2	17	17
A8B2Q4	Ribosomal protein L21 OS=Giardia intestinalis (strain ATCC 50803 / WB cl	341.68	48.43	4	9	9
V6TGV0	Uncharacterized protein OS=Giardia intestinalis OX=5741 GN=DHA2_150	341.37	33.51	2	1	54
A0A644F964	Ribosomal protein S19e OS=Giardia intestinalis (strain ATCC 50803 / WB	324.07	71.22	3	6	11
E1F7W3	40S ribosomal protein S7 OS=Giardia intestinalis (strain P15) OX=658858	316.42	61.58	4	15	15
A8BPE3	4-alpha-glucanotransferase, amylo-alpha-1,6-glucosidase OS=Giardia inte	312.28	40.44	3	21	55
E1EWT0	Ribosomal protein L35a OS=Giardia intestinalis (strain P15) OX=658858 G	308.64	65.04	3	11	11

V6TF82	SSU ribosomal protein S17P OS=Giardia intestinalis OX=5741 GN=DHA2_	302.18	51.29	5	14	14
A0A644FBU1	60S ribosomal protein L18a OS=Giardia intestinalis (strain ATCC 50803 /	300.00	62.79	6	13	13
E1F2W2	Ribosomal protein L31B OS=Giardia intestinalis (strain P15) OX=658858	298.71	34.91	4	5	5
A8B9C0	Importin beta-3 subunit OS=Giardia intestinalis (strain ATCC 50803 / WB	294.52	39.01	4	34	34
V6TJT0	40S ribosomal protein S6 OS=Giardia intestinalis OX=5741 GN=DHA2_14	291.17	54.44	3	13	13
V6T7I9	Ribosomal protein L15 OS=Giardia intestinalis OX=5741 GN=DHA2_8001	289.45	45.10	2	11	11
E1F4K8	Ribosomal protein L23 OS=Giardia intestinalis (strain P15) OX=658858 G	277.26	71.83	2	10	10
E2RU47	Ribosomal protein L5 OS=Giardia intestinalis (strain ATCC 50803 / WB clo	276.05	47.81	3	17	17
V6TCR2	SSU ribosomal protein S17E OS=Giardia intestinalis OX=5741 GN=DHA2_	275.93	54.74	3	9	9
V6TJ23	LSU ribosomal protein L10AE OS=Giardia intestinalis OX=5741 GN=DHA2_	270.57	56.19	3	18	18
A8B1T3	60S ribosomal protein L27 OS=Giardia intestinalis (strain ATCC 50803 / W	266.33	56.30	5	10	10
A0A644FC41	Ribosomal L22e OS=Giardia intestinalis (strain ATCC 50803 / WB clone C	255.36	67.74	5	7	7
E1F4E2	60S acidic ribosomal protein P0 OS=Giardia intestinalis (strain P15) OX=6	245.29	26.38	1	1	11
V6TBT8	LSU ribosomal protein L13P OS=Giardia intestinalis OX=5741 GN=DHA2_	240.95	37.06	2	9	9
A8BMM8	Nucleolar GTP-binding protein 1 OS=Giardia intestinalis (strain ATCC 5080	237.10	50.59	8	32	32
A8BKF1	Ribosomal protein P1B OS=Giardia intestinalis (strain ATCC 50803 / WB c	229.42	50.00	3	3	4
V6TN59	SSU ribosomal protein S9P/S16 OS=Giardia intestinalis OX=5741 GN=DH	228.84	53.16	4	8	8
A0A644EZT7	Ribosomal protein L13 OS=Giardia intestinalis (strain ATCC 50803 / WB c	228.72	52.43	6	12	12
A8B3J7	Aldehyde-alcohol dehydrogenase OS=Giardia intestinalis (strain ATCC 508	222.87	33.00	5	28	28
E1F4M6	40S ribosomal protein S3a OS=Giardia intestinalis (strain P15) OX=65885	221.61	47.98	1	1	13
A8BUK9	Dynein heavy chain, putative OS=Giardia intestinalis (strain ATCC 50803 /	220.67	15.37	4	58	59
A0A644F9H9	Heat shock protein 90 OS=Giardia intestinalis (strain ATCC 50803 / WB cl	217.30	48.86	14	33	33
V6TAA9	SSU ribosomal protein S12E OS=Giardia intestinalis OX=5741 GN=DHA2_	215.98	40.00	4	5	5
A8BFF0	CytoDHC cytoplasmic dynein heavy chain OS=Giardia intestinalis (strain A	212.84	14.69	8	54	54
E1F4R1	Ribosomal protein L2 OS=Giardia intestinalis (strain P15) OX=658858 GN=	208.92	60.16	5	12	12
V6U0I7	40S ribosomal protein S4 OS=Giardia intestinalis OX=5741 GN=GSB_113	208.15	34.46	1	1	10
E1F0U9	4-alpha-glucanotransferase, amylo-alpha-1,6-glucosidase OS=Giardia inte	205.44	23.86	1	1	35
A0A644F4K5	Dynein heavy chain OS=Giardia intestinalis (strain ATCC 50803 / WB clone	201.30	13.98	3	13	58
V6T9R2	LSU ribosomal protein L6P OS=Giardia intestinalis OX=5741 GN=DHA2_1	201.06	67.03	3	11	11
A8BEM5	Dynein heavy chain OS=Giardia intestinalis (strain ATCC 50803 / WB clone	198.75	14.68	3	4	54
A8BFT3	Ribosomal protein L26 OS=Giardia intestinalis (strain ATCC 50803 / WB c	197.52	51.85	5	8	8
V6TG00	Dynein heavy chain OS=Giardia intestinalis OX=5741 GN=DHA2_40496 P	194.09	13.92	4	1	51
A8B852	Pyruvate-flavodoxin oxidoreductase OS=Giardia intestinalis (strain ATCC 5	193.91	33.78	4	9	31
Q76KS2	Ribosomal protein S15a OS=Giardia intestinalis OX=5741 PE=3 SV=1 - [Q	191.94	69.23	4	8	8
A0A644F1V6	Coiled-coil protein OS=Giardia intestinalis (strain ATCC 50803 / WB clone	190.33	35.34	5	43	47
A8BKJ8	Polyadenylate-binding protein, putative OS=Giardia intestinalis (strain ATC	181.88	39.37	4	1	17
V6TN35	Putative RNA binding or ribonucleoprotein domain protein OS=Giardia inte	179.00	39.37	4	1	17
E2RU97	14-3-3 protein OS=Giardia intestinalis (strain ATCC 50803 / WB clone C6)	176.22	58.47	2	17	17
A0A644EYT1	Ankyrin repeat protein 1 OS=Giardia intestinalis (strain ATCC 50803 / WB	173.83	34.63	7	44	44

V6TJY4	Protein Translation Initiation Factor 4A OS=Giardia intestinalis OX=5741 G	171.83	64.45	4	22	23
A8BMD9	Ribosomal protein L32 OS=Giardia intestinalis (strain ATCC 50803 / WB cl	163.54	54.41	4	9	9
A8BL65	Dynein heavy chain OS=Giardia intestinalis (strain ATCC 50803 / WB clone	160.23	22.58	5	41	41
A0A644F2W2	Outer-arm dynein gamma (Fragment) OS=Giardia intestinalis (strain ATCC	160.02	22.95	5	43	45
A8BBP3	Ribosomal protein S15 OS=Giardia intestinalis (strain ATCC 50803 / WB cl	157.86	47.59	5	8	8
A8B612	Nucleolar GTP-binding protein 2 OS=Giardia intestinalis (strain ATCC 5080	157.19	34.46	5	22	22
V6TU50	SSU ribosomal protein S19E OS=Giardia intestinalis OX=5741 GN=GSB_15	156.20	41.73	1	1	6
E3WCX1	Outer-arm dynein beta OS=Giardia intestinalis OX=5741 PE=2 SV=1 - [E3	154.72	21.87	14	45	45
E1F1F0	Dynein heavy chain OS=Giardia intestinalis (strain P15) OX=658858 GN=(	153.33	10.72	1	1	46
C6LR89	60S acidic ribosomal protein P0 OS=Giardia intestinalis (strain ATCC 5058	153.29	26.99	2	2	10
A8BVT2	Midasin OS=Giardia intestinalis (strain ATCC 50803 / WB clone C6) OX=18	152.47	11.91	5	7	42
V6TK71	Putative plectin/S10 domain protein OS=Giardia intestinalis OX=5741 GN=	150.49	44.78	3	5	5
A8BUY7	AAA family ATPase OS=Giardia intestinalis (strain ATCC 50803 / WB clone	149.24	37.93	5	26	26
A0A644F9X9	IAD-5 dynein heavy chain OS=Giardia intestinalis (strain ATCC 50803 / WI	148.99	11.32	8	41	44
A8BVW7	Dynein heavy chain OS=Giardia intestinalis (strain ATCC 50803 / WB clone	147.60	19.95	4	42	43
A8BYY5	Ser/Thr phosphatase 2A regulatory subunit A OS=Giardia intestinalis (stra	147.11	45.86	4	22	22
A0A644F9I4	Axoneme-associated protein GASP-180 OS=Giardia intestinalis (strain ATC	146.59	36.71	7	31	31
A0A644FBC3	Long chain fatty acid CoA ligase 5 OS=Giardia intestinalis (strain ATCC 50	144.66	35.52	4	8	22
V6TF35	LSU ribosomal protein L18E OS=Giardia intestinalis OX=5741 GN=DHA2_1	143.13	43.58	2	3	10
E1F780	Tubulin beta chain OS=Giardia intestinalis (strain P15) OX=658858 GN=G	142.05	49.89	12	16	16
A8BFS2	Dynein heavy chain OS=Giardia intestinalis (strain ATCC 50803 / WB clone	138.57	11.72	5	44	45
E1F7N3	Ribosomal protein L17 OS=Giardia intestinalis (strain P15) OX=658858 GN	138.39	39.02	3	7	7
A8B690	Long chain fatty acid CoA ligase, putative OS=Giardia intestinalis (strain A	134.66	34.43	5	12	26
V6TGG6	SSU ribosomal protein S10P OS=Giardia intestinalis OX=5741 GN=DHA2_(	134.46	57.94	5	9	9
E1F2X1	Ribosomal protein S14 OS=Giardia intestinalis (strain P15) OX=658858 GN	134.33	71.72	2	11	11
E1F6G8	Pyruvate-flavodoxin oxidoreductase OS=Giardia intestinalis (strain P15) O:	134.01	25.52	1	1	23
A8B440	Ankyrin repeat protein 1 OS=Giardia intestinalis (strain ATCC 50803 / WB	132.29	38.30	4	23	23
A8B8H7	40S ribosomal protein S25 OS=Giardia intestinalis (strain ATCC 50803 / W	131.01	30.68	2	3	3
V6TFM8	Midasin OS=Giardia intestinalis OX=5741 GN=DHA2_153310 PE=3 SV=1	130.74	10.77	1	1	36
A8B3S4	BRO1-like domain-containing protein OS=Giardia intestinalis (strain ATCC	128.49	40.92	4	27	27
A8B8Z3	Eukaryotic translation initiation factor 6 OS=Giardia intestinalis (strain ATC	128.18	75.20	5	11	11
Q6Y0Y2	Vacuolar sorting protein 35-like OS=Giardia intestinalis OX=5741 GN=Vps	122.34	29.15	4	19	19
A0A644F843	40S ribosomal protein S30 OS=Giardia intestinalis (strain ATCC 50803 / W	122.08	46.38	2	5	5
A8BBV7	Uncharacterized protein OS=Giardia intestinalis (strain ATCC 50803 / WB (	122.01	8.38	5	36	36
V6TWK4	LSU ribosomal protein L18E OS=Giardia intestinalis OX=5741 GN=GSB_11	117.62	36.31	1	1	8
A0A644F565	Coiled-coil protein OS=Giardia intestinalis (strain ATCC 50803 / WB clone (	116.79	23.89	5	23	23
V6TE11	Uncharacterized protein (Fragment) OS=Giardia intestinalis OX=5741 GN=	116.51	26.62	5	1	28
E1F400	Serine/threonine-protein phosphatase OS=Giardia intestinalis (strain P15)	114.55	48.38	3	15	16
A8BYT7	Uncharacterized protein OS=Giardia intestinalis (strain ATCC 50803 / WB (	114.47	24.64	5	1	28

A8BFR0	Elongation factor 1-gamma OS=Giardia intestinalis (strain ATCC 50803 / V	114.27	45.02	4	17	17
C6LW45	Ribosomal protein L11 OS=Giardia intestinalis (strain ATCC 50581 / GS clc	113.50	51.45	3	10	10
Q86QW8	Putative RNA helicase OS=Giardia intestinalis OX=5741 PE=4 SV=1 - [Q8(	112.97	51.54	6	19	19
Q8I856	Tubulin alpha chain (Fragment) OS=Giardia intestinalis OX=5741 PE=3 S\	112.45	46.70	9	16	16
A8BNB4	Dynein heavy chain OS=Giardia intestinalis (strain ATCC 50803 / WB clone	110.64	10.01	5	33	33
A8B7P2	Ribosomal protein L24 OS=Giardia intestinalis (strain ATCC 50803 / WB cl	110.55	31.22	5	6	6
V6THL0	Uncharacterized protein OS=Giardia intestinalis OX=5741 GN=DHA2_1088	109.89	48.50	4	12	12
A8BUI2	Uncharacterized protein OS=Giardia intestinalis (strain ATCC 50803 / WB	109.78	24.03	3	20	20
A8BAD0	Uncharacterized protein OS=Giardia intestinalis (strain ATCC 50803 / WB	109.61	30.27	4	16	16
A8BD37	Phosphorylase B kinase gamma catalytic chain OS=Giardia intestinalis (str	109.02	35.19	4	21	21
A8BZA6	Pescadillo homolog OS=Giardia intestinalis (strain ATCC 50803 / WB clone	109.01	38.38	7	20	20
A0A644F9R7	Putative Dynein heavy chain OS=Giardia intestinalis (strain ATCC 50803 /	107.88	8.33	5	36	37
A0A644F5K9	Ribosomal protein S27 OS=Giardia intestinalis (strain ATCC 50803 / WB cl	107.36	37.04	1	1	3
A8BG41	ATP-dependent RNA helicase p54, putative OS=Giardia intestinalis (strain	107.26	43.26	4	13	13
A0A644F3M5	Acyl-CoA synthetase OS=Giardia intestinalis (strain ATCC 50803 / WB clor	106.71	31.93	6	20	23
E1F941	Ribosomal protein L37a OS=Giardia intestinalis (strain P15) OX=658858 G	106.55	19.15	3	1	1
V6TC68	SSU ribosomal protein S27E OS=Giardia intestinalis OX=5741 GN=DHA2_	103.99	25.00	2	1	3
A8BJ87	IFT complex B OS=Giardia intestinalis (strain ATCC 50803 / WB clone C6)	102.91	16.99	5	27	27
A8B867	Uncharacterized protein OS=Giardia intestinalis (strain ATCC 50803 / WB	102.67	21.90	4	20	23
V6THI5	LSU ribosomal protein L34E OS=Giardia intestinalis OX=5741 GN=DHA2_	101.72	61.67	3	10	10
A8BDZ8	FYVE zinc finger domain-containing protein OS=Giardia intestinalis (strain	101.01	24.75	5	16	16
A0A644F4P9	Bip OS=Giardia intestinalis (strain ATCC 50803 / WB clone C6) OX=18492	100.16	40.48	8	23	23
A8BAF5	Coiled-coil protein OS=Giardia intestinalis (strain ATCC 50803 / WB clone	98.63	21.97	4	27	27
E1EXH7	Elongation factor 1-alpha OS=Giardia intestinalis (strain P15) OX=658858	98.45	41.63	19	17	17
A0A644F2E6	Interferon-related developmental regulator family protein OS=Giardia inte	97.73	24.80	7	11	11
A8BGY3	Translational activator GCN1 OS=Giardia intestinalis (strain ATCC 50803 /	96.44	11.94	5	26	26
A8B9W0	Kinase, NEK OS=Giardia intestinalis (strain ATCC 50803 / WB clone C6) O	96.38	36.44	3	16	17
A0A644FAD8	Ribosome assembly factor mrt4 OS=Giardia intestinalis (strain ATCC 5080	96.15	36.93	6	8	8
Q24982	Pyruvate:ferredoxin oxidoreductase OS=Giardia intestinalis OX=5741 PE=	95.10	19.95	5	22	22
V6TLD4	SSU ribosomal protein S28E OS=Giardia intestinalis OX=5741 GN=DHA2_	95.06	57.81	2	6	6
A8BLD9	Kinase, NEK OS=Giardia intestinalis (strain ATCC 50803 / WB clone C6) O	93.26	34.19	4	25	25
A0A644F2V1	Trophozoite antigen GTA-2 OS=Giardia intestinalis (strain ATCC 50803 / V	91.63	34.22	7	6	6
A8BS80	Ribosome biogenesis protein NOP53 OS=Giardia intestinalis (strain ATCC 5	91.58	64.76	4	19	19
A0A644EZH6	Putative 26S proteasome regulatory subunit OS=Giardia intestinalis (strair	91.39	26.44	5	25	25
A8B2A9	Kinase, CMGC CDK OS=Giardia intestinalis (strain ATCC 50803 / WB clone	90.73	51.89	2	13	13
E1F6U3	Uncharacterized protein OS=Giardia intestinalis (strain P15) OX=658858 G	89.22	11.03	1	1	20
A8BMA4	Uncharacterized protein OS=Giardia intestinalis (strain ATCC 50803 / WB	88.70	9.86	8	27	28
A0A644EY11	Ankyrin repeat protein 3 OS=Giardia intestinalis (strain ATCC 50803 / WB	88.15	18.07	5	22	22
A8BIH3	Uncharacterized protein OS=Giardia intestinalis (strain ATCC 50803 / WB	87.40	11.06	6	25	25

E1F7G3	Ribosomal protein S13 OS=Giardia intestinalis (strain P15) OX=658858 GN	86.36	46.75	4	10	10
A8BN44	Uncharacterized protein OS=Giardia intestinalis (strain ATCC 50803 / WB	86.11	28.72	5	21	21
A8BA45	Nucleolar GTPase OS=Giardia intestinalis (strain ATCC 50803 / WB clone C	85.97	36.04	6	20	20
A8BAA3	Uncharacterized protein OS=Giardia intestinalis (strain ATCC 50803 / WB	85.82	37.08	4	15	15
A8BPA7	Kinase, CDC7 OS=Giardia intestinalis (strain ATCC 50803 / WB clone C6) C	85.70	20.04	7	25	25
O97445	Ribosomal protein L35 OS=Giardia intestinalis OX=5741 GN=RPL35 PE=3	85.21	38.71	4	7	7
V6TBL5	SSU ribosomal protein S24E OS=Giardia intestinalis OX=5741 GN=DHA2_	84.54	59.09	3	6	6
A8BA54	Uncharacterized protein OS=Giardia intestinalis (strain ATCC 50803 / WB	83.85	36.57	4	20	20
V6T9J4	LSU ribosomal protein L15P OS=Giardia intestinalis OX=5741 GN=DHA2_1	83.66	50.34	3	9	10
A8B942	Uncharacterized protein OS=Giardia intestinalis (strain ATCC 50803 / WB	83.12	23.53	3	10	16
A8B4I9	Kinesin-6 OS=Giardia intestinalis (strain ATCC 50803 / WB clone C6) OX=	82.99	21.78	4	19	19
A0A644FBG0	Kinase, CAMK CAMKL OS=Giardia intestinalis (strain ATCC 50803 / WB clo	82.95	46.75	4	13	13
A8B3A0	Ankyrin repeat protein 1 OS=Giardia intestinalis (strain ATCC 50803 / WB	82.09	17.50	5	23	23
A8BM14	Uncharacterized protein OS=Giardia intestinalis (strain ATCC 50803 / WB	80.91	4.82	5	25	25
V6TCR6	LSU ribosomal protein L24E OS=Giardia intestinalis OX=5741 GN=DHA2_1	80.27	41.18	3	8	8
A8B515	Clathrin heavy chain OS=Giardia intestinalis (strain ATCC 50803 / WB clon	79.98	14.11	5	20	20
A8BQU9	Chaperone protein DnaJ OS=Giardia intestinalis (strain ATCC 50803 / WB	79.63	38.39	5	15	15
Q8MQT8	GTP-binding protein Sar1 OS=Giardia intestinalis OX=5741 GN=SAR1 PE=	79.45	87.43	4	12	12
A8BH19	Kinase, NEK OS=Giardia intestinalis (strain ATCC 50803 / WB clone C6) O	78.47	39.22	4	12	12
Q4VPP2	Alpha-11 giardin OS=Giardia intestinalis OX=5741 PE=1 SV=1 - [Q4VPP2_	77.55	43.65	4	10	10
Q8T4M7	Putative adaptor protein complex medium subunit OS=Giardia intestinalis	77.55	42.40	4	13	13
A0A644F9L3	Uncharacterized protein OS=Giardia intestinalis (strain ATCC 50803 / WB	77.49	17.82	6	17	17
A8BGS8	WD-repeat membrane protein OS=Giardia intestinalis (strain ATCC 50803	76.52	14.24	5	19	19
A8BG90	ATP-dependent RNA helicase HAS1, putative OS=Giardia intestinalis (strai	76.36	31.44	4	12	12
A8BRB8	Chromodomain helicase-DNA-binding protein, putative OS=Giardia intestin	76.17	11.34	5	20	21
A8BFR5	Threonine dehydratase OS=Giardia intestinalis (strain ATCC 50803 / WB c	75.86	27.64	6	19	19
A8B8Y7	Kinase, NEK OS=Giardia intestinalis (strain ATCC 50803 / WB clone C6) O	75.34	41.75	18	7	10
A8BGC8	RuvB-like helicase OS=Giardia intestinalis (strain ATCC 50803 / WB clone	75.21	53.85	3	21	21
A8BJX2	Kinase, NEK OS=Giardia intestinalis (strain ATCC 50803 / WB clone C6) O	74.41	54.22	6	12	12
A8B9N2	Arginyl-tRNA synthetase OS=Giardia intestinalis (strain ATCC 50803 / WB	74.25	33.01	5	16	16
A8BBN3	FtsJ cell division protein, putative OS=Giardia intestinalis (strain ATCC 508	74.23	19.56	5	19	19
E1F4P2	Protein 21.1 OS=Giardia intestinalis (strain P15) OX=658858 GN=GLP15_	74.21	47.27	3	12	12
A8BQ33	Nucleolar protein, Nop52 OS=Giardia intestinalis (strain ATCC 50803 / WB	73.79	40.04	5	13	13
E2RTY1	Head-stalk protein GHSP-115 OS=Giardia intestinalis OX=5741 PE=4 SV=	73.40	25.70	4	20	20
A8BEQ2	Uncharacterized protein OS=Giardia intestinalis (strain ATCC 50803 / WB	72.28	17.67	4	6	18
A8BAF1	IFT complex A OS=Giardia intestinalis (strain ATCC 50803 / WB clone C6)	72.24	12.65	4	17	17
A8BD66	Ribosome production factor 2 homolog OS=Giardia intestinalis (strain ATC	72.23	25.30	4	11	11
A8B4T8	Coatomer alpha subunit OS=Giardia intestinalis (strain ATCC 50803 / WB	71.28	22.63	3	20	20
A8BQ26	Pyruvate, phosphate dikinase OS=Giardia intestinalis (strain ATCC 50803 /	70.77	21.83	6	16	16

A8BH36	Kinesin-5 OS=Giardia intestinalis (strain ATCC 50803 / WB clone C6) OX=	70.72	28.05	8	20	21
A8BBI5	T-complex protein 1 subunit eta OS=Giardia intestinalis (strain ATCC 5080	70.44	33.45	5	13	13
A8B762	HEAT repeat-containing protein 1 OS=Giardia intestinalis (strain ATCC 508	70.31	11.00	5	20	20
A8B8R9	T-complex protein 1 subunit gamma OS=Giardia intestinalis (strain ATCC 5	70.06	37.94	5	16	16
A8BHN6	Uncharacterized protein OS=Giardia intestinalis (strain ATCC 50803 / WB c	68.90	45.43	6	14	15
V6TLU7	DUF1681 domain-containing protein OS=Giardia intestinalis OX=5741 GN=	68.42	62.92	2	9	9
A8BQI0	Axoneme central apparatus protein OS=Giardia intestinalis (strain ATCC 50	68.17	28.29	5	10	10
Q8WP40	Phosphopyruvate hydratase OS=Giardia intestinalis OX=5741 GN=enol PE	67.90	43.60	4	14	14
A8B549	Uncharacterized protein OS=Giardia intestinalis (strain ATCC 50803 / WB c	67.83	8.05	6	22	22
V6TD90	NADH oxidase OS=Giardia intestinalis OX=5741 GN=DHA2_9719 PE=4 SV	67.65	34.97	3	11	11
A8BWL4	Uncharacterized protein OS=Giardia intestinalis (strain ATCC 50803 / WB c	66.87	8.90	2	20	20
A8BAK9	Protein phosphatase 2A B' regulatory subunit Wdb1 OS=Giardia intestinali	66.31	29.96	4	17	17
A0A644F600	Kinase, CAMKK OS=Giardia intestinalis (strain ATCC 50803 / WB clone C6)	66.21	9.70	3	18	18
A8BNS2	STU2-like protein OS=Giardia intestinalis (strain ATCC 50803 / WB clone C	66.21	12.79	4	18	19
A0A644F2P7	Ankyrin repeat protein 1 OS=Giardia intestinalis (strain ATCC 50803 / WB	65.87	9.29	5	17	17
A8BPV3	Vacuolar ATP synthase subunit B OS=Giardia intestinalis (strain ATCC 508	65.62	29.38	5	10	10
A8BR51	Uncharacterized protein OS=Giardia intestinalis (strain ATCC 50803 / WB c	64.99	19.51	7	15	15
A0A644F001	Dynein heavy chain OS=Giardia intestinalis (strain ATCC 50803 / WB clone	64.65	4.46	5	18	18
A8BAU7	CCT-theta OS=Giardia intestinalis (strain ATCC 50803 / WB clone C6) OX=	64.37	34.28	5	12	12
A8BW44	Pyruvate kinase OS=Giardia intestinalis (strain ATCC 50803 / WB clone C6	63.73	25.23	6	13	13
A8BEY6	26S protease regulatory subunit 7 OS=Giardia intestinalis (strain ATCC 50	63.03	38.63	4	14	14
A8B8T3	RNA recognition motif-containing protein OS=Giardia intestinalis (strain AT	62.72	23.77	4	14	14
A8BKY7	Ankyrin repeat protein 1 OS=Giardia intestinalis (strain ATCC 50803 / WB	62.64	16.09	5	16	16
A8BJ15	Intraflagellar transport protein 122 homolog OS=Giardia intestinalis (strain	62.63	13.05	4	17	17
A8B636	Nucleolar protein NOP2 OS=Giardia intestinalis (strain ATCC 50803 / WB c	61.87	29.68	5	12	13
A8BGL6	Kinase, SCY1 OS=Giardia intestinalis (strain ATCC 50803 / WB clone C6) C	61.83	17.95	4	13	13
A8B9U8	AP complex subunit beta OS=Giardia intestinalis (strain ATCC 50803 / WB	61.23	16.70	5	15	15
A0A644F867	Vacuolar protein sorting 26 OS=Giardia intestinalis (strain ATCC 50803 / W	60.69	18.77	6	7	7
E1F802	Uncharacterized protein OS=Giardia intestinalis (strain P15) OX=658858 G	60.61	45.86	3	13	13
A8BKM6	Uncharacterized protein OS=Giardia intestinalis (strain ATCC 50803 / WB c	60.39	33.71	5	11	11
A0A644F2F6	Ankyrin repeat protein 1 OS=Giardia intestinalis (strain ATCC 50803 / WB	59.83	25.27	6	16	16
A8BLC3	Kinase, NEK OS=Giardia intestinalis (strain ATCC 50803 / WB clone C6) O	59.72	23.69	5	13	13
A8BQI1	Phosphatidylinositol transfer protein alpha isoform OS=Giardia intestinalis	59.53	45.22	4	14	14
A8BVR5	Coatomer gamma subunit OS=Giardia intestinalis (strain ATCC 50803 / W	59.11	21.15	5	15	15
Q8T6L4	Vesicle-fusing ATPase OS=Giardia intestinalis OX=5741 GN=VPS4 PE=3 S	58.89	35.90	6	9	15
A8BB76	V-type proton ATPase subunit C OS=Giardia intestinalis (strain ATCC 5080	58.69	23.89	3	11	11
A8B724	Uncharacterized protein OS=Giardia intestinalis (strain ATCC 50803 / WB c	58.52	13.20	4	15	15
A8B2F3	Ribosomal protein L10e OS=Giardia intestinalis (strain ATCC 50803 / WB c	58.49	42.90	5	13	13
A0A644F1R1	Vesicle-fusing ATPase OS=Giardia intestinalis (strain ATCC 50803 / WB clo	57.98	22.37	2	10	16

A0A644FA59	Vacuolar protein sorting 4b OS=Giardia intestinalis (strain ATCC 50803 / W	57.73	35.20	5	7	13
A8BDZ5	Kinase, NEK OS=Giardia intestinalis (strain ATCC 50803 / WB clone C6) O)	57.68	35.90	6	13	13
A8BHG1	Vacuolar ATP synthase subunit H OS=Giardia intestinalis (strain ATCC 508	57.67	35.85	5	13	13
A0A644EYQ7	Uncharacterized protein OS=Giardia intestinalis (strain ATCC 50803 / WB	57.39	10.32	6	15	15
E1F1K7	Kinase, STE STE20 OS=Giardia intestinalis (strain P15) OX=658858 GN=G	57.35	40.91	4	8	8
A8BDH0	Kinase, NEK OS=Giardia intestinalis (strain ATCC 50803 / WB clone C6) O)	57.01	30.82	4	15	16
Q9GU01	Chaperonin subunit epsilon CCTepsilon OS=Giardia intestinalis OX=5741 (	56.67	33.45	3	6	14
A8B1S2	Kinase, AGC MAST OS=Giardia intestinalis (strain ATCC 50803 / WB clone	56.27	12.35	4	12	12
Q0GK31	UPF1 OS=Giardia intestinalis OX=5741 PE=4 SV=1 - [Q0GK31_GIAIN]	56.24	16.79	4	15	15
A8BRR0	Kinase, NEK OS=Giardia intestinalis (strain ATCC 50803 / WB clone C6) O)	56.05	17.69	40	9	12
A8BQW1	ATP-dependent RNA helicase-like protein OS=Giardia intestinalis (strain AT	55.73	37.04	6	15	16
A8BR89	Prolyl-tRNA synthetase OS=Giardia intestinalis (strain ATCC 50803 / WB c	55.46	27.68	5	13	13
A8B9T1	Uncharacterized protein OS=Giardia intestinalis (strain ATCC 50803 / WB	52.77	3.24	5	14	15
E1F7R3	Serine/threonine-protein phosphatase OS=Giardia intestinalis (strain P15)	52.75	41.31	4	10	10
Q8ISM1	G protein beta subunit (Fragment) OS=Giardia intestinalis OX=5741 PE=2	52.51	21.19	5	15	15
A8BQU3	Kelch repeat-containing protein OS=Giardia intestinalis (strain ATCC 5080	52.37	14.07	4	15	15
A8B371	Uncharacterized protein OS=Giardia intestinalis (strain ATCC 50803 / WB	52.01	19.88	5	12	12
A8B963	Kinase, ULK OS=Giardia intestinalis (strain ATCC 50803 / WB clone C6) O)	51.09	11.90	3	8	15
A8BZ88	Uncharacterized protein OS=Giardia intestinalis (strain ATCC 50803 / WB	50.84	19.63	4	18	18
C6LRJ9	Mitogen-activated protein kinase OS=Giardia intestinalis (strain ATCC 505	50.79	46.94	5	13	13
V6TEC7	Putative TCP-1/cpn60 chaperonin family protein OS=Giardia intestinalis O)	50.60	21.29	4	13	13
V6TH75	Putative p23-like domain protein OS=Giardia intestinalis OX=5741 GN=DH	50.45	56.22	3	8	8
D3KH09	Uncharacterized protein OS=Giardia intestinalis (strain ATCC 50803 / WB	50.28	27.92	4	11	11
A8BE61	Intraflagellar transport protein IFT74/72 OS=Giardia intestinalis (strain AT	49.80	27.42	3	5	12
A8BXA7	Uncharacterized protein OS=Giardia intestinalis (strain ATCC 50803 / WB	49.70	29.10	5	12	12
A8BT95	Uncharacterized protein OS=Giardia intestinalis (strain ATCC 50803 / WB	49.69	22.91	4	7	7
A8BHW1	Kinase, CAMK CAMKL OS=Giardia intestinalis (strain ATCC 50803 / WB clo	49.62	41.20	4	12	12
A0A644F5K4	Protein phosphatase PP2A regulatory subunit A OS=Giardia intestinalis (st	49.17	29.83	5	15	15
A8BM09	WD-40 repeat protein OS=Giardia intestinalis (strain ATCC 50803 / WB clo	49.16	18.02	5	13	13
E1F796	Uncharacterized protein OS=Giardia intestinalis (strain P15) OX=658858 G	49.00	13.31	3	1	13
D3KGC1	RasGEF_N_2 domain-containing protein OS=Giardia intestinalis (strain AT(	48.85	7.26	3	11	11
A8BK50	Peptidylprolyl isomerase OS=Giardia intestinalis (strain ATCC 50803 / WB	48.51	23.16	4	6	6
A8BDT1	MSP domain-containing protein OS=Giardia intestinalis (strain ATCC 5080	48.32	5.50	5	12	12
A8BXE6	Ankyrin repeat protein 1 OS=Giardia intestinalis (strain ATCC 50803 / WB	48.17	19.22	2	8	8
A0A644F4Z1	Ankyrin repeat protein 1 OS=Giardia intestinalis (strain ATCC 50803 / WB	48.09	12.51	21	13	14
A0A644F5D1	Uncharacterized protein OS=Giardia intestinalis (strain ATCC 50803 / WB	47.86	7.76	4	15	15
A8BJ82	26S protease regulatory subunit 8 OS=Giardia intestinalis (strain ATCC 50	47.86	35.16	5	13	13
A8B296	IFT complex B OS=Giardia intestinalis (strain ATCC 50803 / WB clone C6)	47.78	24.26	5	13	13
A8BRD6	Zinc finger domain protein OS=Giardia intestinalis (strain ATCC 50803 / W	47.69	26.98	5	12	12

A8BU40	RuvB-like helicase OS=Giardia intestinalis (strain ATCC 50803 / WB clone	47.68	33.75	5	10	10
Q9GU04	CCT-beta (Fragment) OS=Giardia intestinalis OX=5741 GN=Cctb PE=3 SV	47.37	32.10	7	12	12
A8B607	TRP domain containing protein OS=Giardia intestinalis (strain ATCC 50803	47.32	10.04	5	13	14
A8B3P4	Coiled-coil protein OS=Giardia intestinalis (strain ATCC 50803 / WB clone	47.13	16.22	4	13	13
A8BKY9	Coiled-coil protein OS=Giardia intestinalis (strain ATCC 50803 / WB clone	47.05	8.71	5	14	14
A8B382	Ribosome biogenesis protein BMS1 OS=Giardia intestinalis (strain ATCC 50	46.97	14.08	5	14	14
A8B9M9	Long chain fatty acid CoA ligase 4 OS=Giardia intestinalis (strain ATCC 50	46.97	19.78	6	9	12
D3KHL8	SWIRM domain-containing protein OS=Giardia intestinalis (strain ATCC 50	46.94	17.95	3	11	11
A8BU04	Kinase, CMGC DYRK OS=Giardia intestinalis (strain ATCC 50803 / WB clon	46.83	13.53	4	13	13
A0A1Z1VV01	Alpha1-giardin OS=Giardia intestinalis OX=5741 PE=4 SV=1 - [A0A1Z1VV	46.75	32.88	28	4	9
A8BIN5	Uncharacterized protein OS=Giardia intestinalis (strain ATCC 50803 / WB	46.73	11.08	5	14	14
A8B4X8	Uncharacterized protein OS=Giardia intestinalis (strain ATCC 50803 / WB	46.54	9.90	3	10	10
A8B4U1	Uncharacterized protein OS=Giardia intestinalis (strain ATCC 50803 / WB	46.39	11.51	4	15	15
A8BHU2	Uncharacterized protein OS=Giardia intestinalis (strain ATCC 50803 / WB	46.30	19.72	5	14	14
A8BFR3	Uncharacterized protein OS=Giardia intestinalis (strain ATCC 50803 / WB	46.19	28.66	4	12	12
A8B4I3	Acyl-CoA synthetase OS=Giardia intestinalis (strain ATCC 50803 / WB clon	45.89	12.93	5	7	10
A8BRV4	Uncharacterized protein OS=Giardia intestinalis (strain ATCC 50803 / WB	45.80	7.79	3	13	13
A8W222	26S proteasome ATPase subunit S4 OS=Giardia intestinalis OX=5741 GN=	45.76	31.32	5	4	9
A8BIV9	Non-specific serine/threonine protein kinase OS=Giardia intestinalis (strain	45.70	5.14	5	13	13
A8BBN0	Nucleolar protein NOP5 OS=Giardia intestinalis (strain ATCC 50803 / WB c	45.60	28.06	5	14	14
A8BEU7	SH2_2 domain-containing protein OS=Giardia intestinalis (strain ATCC 508	45.55	5.78	4	14	14
A8BT70	Ciliary dynein heavy chain 11 OS=Giardia intestinalis (strain ATCC 50803 /	45.53	2.34	5	13	13
V6TH01	SSU ribosomal protein S8E OS=Giardia intestinalis OX=5741 GN=DHA2_1	45.52	39.62	4	11	11
A8BUL3	Eukaryotic translation initiation factor 5B OS=Giardia intestinalis (strain AT	45.40	18.03	4	12	12
E1F4K9	Tetratricopeptide repeat protein OS=Giardia intestinalis (strain P15) OX=6	44.82	27.22	3	12	12
A0A644F2H1	Vesicle-fusing ATPase OS=Giardia intestinalis (strain ATCC 50803 / WB clo	44.76	19.05	5	6	12
A8B2T4	Uncharacterized protein OS=Giardia intestinalis (strain ATCC 50803 / WB	44.73	28.44	4	7	7
A8B4M6	60S ribosomal export protein NMD3 OS=Giardia intestinalis (strain ATCC 5	44.45	22.40	4	9	9
A8BK70	Sec7 family protein OS=Giardia intestinalis (strain ATCC 50803 / WB clone	44.14	9.09	6	16	16
A0A0F6PP63	Beta-giardin (Fragment) OS=Giardia intestinalis OX=5741 GN=bg PE=3 S	44.03	61.30	373	1	12
A8B3K5	Transcription regulatory protein SNF2 OS=Giardia intestinalis (strain ATCC	43.92	9.24	5	13	13
V6TY52	Uncharacterized protein OS=Giardia intestinalis OX=5741 GN=GSB_16602	43.85	9.09	1	1	7
A8BWU7	Uncharacterized protein OS=Giardia intestinalis (strain ATCC 50803 / WB	43.81	18.66	5	13	14
A8BUR1	Spindle pole body protein, putative OS=Giardia intestinalis (strain ATCC 50	43.67	11.20	4	14	14
A8BC97	Uncharacterized protein OS=Giardia intestinalis (strain ATCC 50803 / WB	43.59	6.41	4	12	12
A0A644F0Y1	Gamma-tubulin OS=Giardia intestinalis (strain ATCC 50803 / WB clone C6	43.58	35.81	5	10	10
A8BD53	Sir2 family protein OS=Giardia intestinalis (strain ATCC 50803 / WB clone	43.46	25.59	2	11	11
V6TG04	Serine/threonine-protein phosphatase OS=Giardia intestinalis OX=5741 G	43.38	40.71	3	10	11
A0A3G9JZW8	Beta giardin (Fragment) OS=Giardia intestinalis OX=5741 PE=3 SV=1 - [A	43.30	57.33	147	2	12

A8B9C5	IFT complex B OS=Giardia intestinalis (strain ATCC 50803 / WB clone C6)	43.16	16.13	5	10	10
Q2VIU8	Beta-giardin (Fragment) OS=Giardia intestinalis OX=5741 PE=3 SV=1 - [(	43.13	63.52	637	1	13
A8BSN9	Protein kinase domain-containing protein OS=Giardia intestinalis (strain A	43.02	10.07	4	14	14
V6TKE0	ATP-dependent 26S proteasome regulatory subunit OS=Giardia intestinalis	42.68	24.87	3	7	7
A0A644F7Y6	DEAD box RNA helicase Vasa OS=Giardia intestinalis (strain ATCC 50803 /	42.68	25.79	6	11	13
D3KH05	Uncharacterized protein OS=Giardia intestinalis (strain ATCC 50803 / WB (	42.63	14.25	8	15	15
A0A644EYW9	Putative TPR repeat family protein OS=Giardia intestinalis (strain ATCC 50	42.57	23.92	5	13	13
Q86LC5	GTPase Rab11-like protein OS=Giardia intestinalis OX=5741 GN=Rab11 P	42.41	26.39	5	4	4
A8BE70	ABC transporter, ATP-binding protein OS=Giardia intestinalis (strain ATCC	42.39	18.69	5	11	11
A8B5J7	Inositol 5-phosphatase 4 OS=Giardia intestinalis (strain ATCC 50803 / WB	42.19	17.62	4	15	15
A8BIP6	HECT domain-containing protein OS=Giardia intestinalis (strain ATCC 508(	42.14	5.70	3	11	11
A0A644F070	Ankyrin repeat protein 1 OS=Giardia intestinalis (strain ATCC 50803 / WB	42.01	51.39	6	9	9
A8B5T0	Manganese-dependent inorganic pyrophosphatase, putative OS=Giardia ir	41.85	23.30	5	11	11
A0A644F6N5	KIF-binding protein OS=Giardia intestinalis (strain ATCC 50803 / WB clone	41.83	17.44	4	14	14
A8BQB1	Mitotic spindle checkpoint protein MAD2 OS=Giardia intestinalis (strain AT	41.70	34.98	3	1	5
A8BFG4	T-complex protein 1 subunit delta OS=Giardia intestinalis (strain ATCC 50	41.67	27.47	5	11	11
A8B508	Uncharacterized protein OS=Giardia intestinalis (strain ATCC 50803 / WB (	41.66	9.77	4	12	12
V6T9U7	LSU ribosomal protein L36E OS=Giardia intestinalis OX=5741 GN=DHA2_1	41.65	43.33	2	4	4
A0A644F4A6	Uncharacterized protein OS=Giardia intestinalis (strain ATCC 50803 / WB (	41.60	7.54	3	12	12
A8BK79	Xaa-Pro dipeptidase OS=Giardia intestinalis (strain ATCC 50803 / WB clon	41.59	26.13	4	9	9
A8BCS1	Putative Spindle pole protein OS=Giardia intestinalis (strain ATCC 50803 /	41.54	14.27	3	9	9
A8BED4	Rhodanese-like domain-containing protein OS=Giardia intestinalis (strain A	41.50	29.63	4	8	8
A8BXX3	Nicotinate phosphoribosyltransferase OS=Giardia intestinalis (strain ATCC	41.31	19.90	5	10	10
A8BQS0	ATP-dependent RNA helicase OS=Giardia intestinalis (strain ATCC 50803 /	41.27	15.44	5	9	9
A8BS83	rRNA biogenesis protein RRP5 OS=Giardia intestinalis (strain ATCC 50803	41.20	9.45	5	12	12
A8BPW2	Kinase, NAK OS=Giardia intestinalis (strain ATCC 50803 / WB clone C6) O	40.73	18.38	5	9	9
A8B6Y0	Lipopolysaccharide-responsive and beige-like anchor protein OS=Giardia ir	40.64	3.64	4	11	11
A8BRT4	Uncharacterized protein OS=Giardia intestinalis (strain ATCC 50803 / WB (	40.54	24.43	4	12	12
A8BQ17	26S proteasome regulatory complex component OS=Giardia intestinalis (s	40.24	37.68	3	11	11
A8BE80	START domain-containing protein OS=Giardia intestinalis (strain ATCC 50	40.24	43.51	7	8	8
O97452	Protein disulfide isomerase-2 OS=Giardia intestinalis OX=5741 GN=PDI-2	40.21	18.49	4	8	8
A8B6N1	Not1 N-terminal domain, CCR4-Not complex component-containing protei	40.15	18.66	4	9	9
A8B909	Uncharacterized protein OS=Giardia intestinalis (strain ATCC 50803 / WB (	40.15	7.10	4	13	13
A0A482G8E3	Thioredoxin peroxidase OS=Giardia intestinalis OX=5741 GN=prx1 PE=3 S	39.96	32.84	10	5	6
A8BV70	Actin OS=Giardia intestinalis (strain ATCC 50803 / WB clone C6) OX=184	39.72	28.00	11	8	8
A0A644F2A0	Ankyrin repeat protein 1 OS=Giardia intestinalis (strain ATCC 50803 / WB	39.68	21.73	3	10	10
A8BFX3	Kinase, NEK OS=Giardia intestinalis (strain ATCC 50803 / WB clone C6) O	39.40	13.73	13	10	10
A8BEZ0	Glutamyl-tRNA synthetase OS=Giardia intestinalis (strain ATCC 50803 / W	39.26	16.52	4	9	9
A8BJW0	RING-type domain-containing protein OS=Giardia intestinalis (strain ATCC	39.24	17.97	4	10	10

D3KGW1	Uncharacterized protein OS=Giardia intestinalis (strain ATCC 50803 / WB (	39.22	14.64	3	11	11
D3KHU3	Uncharacterized protein OS=Giardia intestinalis (strain ATCC 50803 / WB (	38.99	17.67	5	11	11
A8BTU8	Kinase, CMGC DYRK OS=Giardia intestinalis (strain ATCC 50803 / WB clon	38.95	15.00	6	11	11
D3KHQ2	Beige/BEACH domain-containing protein OS=Giardia intestinalis (strain AT	38.81	4.61	5	10	10
A8BIZ4	Kinase, CAMK CAMKL OS=Giardia intestinalis (strain ATCC 50803 / WB clo	38.66	22.42	4	10	10
A8B7I6	Uncharacterized protein OS=Giardia intestinalis (strain ATCC 50803 / WB (	38.66	22.57	4	8	8
D3KHJ6	Uncharacterized protein OS=Giardia intestinalis (strain ATCC 50803 / WB (	38.65	4.31	3	12	12
A8BKW6	Uncharacterized protein OS=Giardia intestinalis (strain ATCC 50803 / WB (	38.63	20.65	3	7	7
A8BMP5	Uncharacterized protein OS=Giardia intestinalis (strain ATCC 50803 / WB (	38.51	11.91	4	10	10
A8BLZ3	Kinase OS=Giardia intestinalis (strain ATCC 50803 / WB clone C6) OX=18	38.30	6.41	4	11	11
A8BXI4	Dynein-like protein OS=Giardia intestinalis (strain ATCC 50803 / WB clone	38.15	23.13	5	8	8
A8BDQ0	Coatomer beta' subunit OS=Giardia intestinalis (strain ATCC 50803 / WB c	38.14	15.55	6	11	11
C6LT33	Mitotic spindle checkpoint protein MAD2 OS=Giardia intestinalis (strain AT	37.84	34.98	2	1	5
A0A644F1P3	Uncharacterized protein OS=Giardia intestinalis (strain ATCC 50803 / WB (	37.78	9.77	5	12	12
A8BR71	Sec31 OS=Giardia intestinalis (strain ATCC 50803 / WB clone C6) OX=184	37.76	12.40	2	10	10
E1F3T7	Dynein light intermediate chain OS=Giardia intestinalis (strain P15) OX=6!	37.56	38.72	3	8	8
E1F1Q9	Uncharacterized protein OS=Giardia intestinalis (strain P15) OX=658858 G	37.52	41.56	3	3	3
A8BH24	Kinase, NEK OS=Giardia intestinalis (strain ATCC 50803 / WB clone C6) O:	37.49	20.25	11	8	8
A0A644F649	Intraflagellar transport protein IFT88 OS=Giardia intestinalis (strain ATCC	37.42	18.89	7	11	11
V6TDL4	Uncharacterized protein OS=Giardia intestinalis OX=5741 GN=DHA2_154	37.30	7.93	5	8	8
A0A644F1D4	Lipid binding protein OS=Giardia intestinalis (strain ATCC 50803 / WB clor	37.30	36.64	6	7	7
A8BER9	Median body protein OS=Giardia intestinalis (strain ATCC 50803 / WB clor	36.91	15.87	3	10	10
A0A644F0J2	Uncharacterized protein OS=Giardia intestinalis (strain ATCC 50803 / WB (	36.90	8.32	3	11	11
Q27657	Arginine deiminase OS=Giardia intestinalis OX=5741 PE=4 SV=1 - [Q276!	36.81	18.45	4	9	9
A8BBY3	ATP-dependent RNA helicase A OS=Giardia intestinalis (strain ATCC 5080	36.74	12.09	3	13	13
A8B4Z7	Kinesin-3 OS=Giardia intestinalis (strain ATCC 50803 / WB clone C6) OX=	36.57	13.45	7	5	11
A8BMB1	Serologically defined colon cancer antigen 1 OS=Giardia intestinalis (strair	36.51	15.24	4	13	13
A8BG63	Uncharacterized protein OS=Giardia intestinalis (strain ATCC 50803 / WB (	36.45	7.03	4	10	10
A8BUQ4	Chaperone protein DnaJ OS=Giardia intestinalis (strain ATCC 50803 / WB	36.41	21.82	5	10	10
A0A644EYB8	DNA-directed DNA polymerase OS=Giardia intestinalis (strain ATCC 50803	36.39	11.69	4	4	10
A8BGN3	CCAAT-box-binding transcription factor OS=Giardia intestinalis (strain ATC	36.36	11.43	4	10	10
A8BN33	26S protease regulatory subunit 6A OS=Giardia intestinalis (strain ATCC 5	36.25	18.36	4	9	9
D3KG16	Uncharacterized protein OS=Giardia intestinalis (strain ATCC 50803 / WB (	36.21	10.14	6	11	11
A8BZ95	Kinase, CMGC CDK OS=Giardia intestinalis (strain ATCC 50803 / WB clone	36.17	38.64	6	9	9
A8B433	Farnesyl diphosphate synthase OS=Giardia intestinalis (strain ATCC 50803	36.13	32.68	4	10	10
A8BLU8	Uncharacterized protein OS=Giardia intestinalis (strain ATCC 50803 / WB (	36.09	14.43	5	11	11
E1F085	Kinase, NEK OS=Giardia intestinalis (strain P15) OX=658858 GN=GLP15_	35.94	14.10	3	5	5
E1F889	Fibrillarin-like protein OS=Giardia intestinalis (strain P15) OX=658858 GN=	35.68	38.53	3	9	9
A8BBV1	Uncharacterized protein OS=Giardia intestinalis (strain ATCC 50803 / WB (	35.66	9.07	5	9	9

A0A644F0L5	Uncharacterized protein OS=Giardia intestinalis (strain ATCC 50803 / WB	35.43	21.79	4	8	8
A0A644F8L6	Uncharacterized protein OS=Giardia intestinalis (strain ATCC 50803 / WB	35.33	8.52	4	9	9
V6T7V8	Uncharacterized protein OS=Giardia intestinalis OX=5741 GN=DHA2_905	35.24	34.00	3	11	11
A8BV80	Kinase, NEK OS=Giardia intestinalis (strain ATCC 50803 / WB clone C6) O	35.21	11.08	4	12	12
V6TJ64	Uncharacterized protein OS=Giardia intestinalis OX=5741 GN=DHA2_804	35.17	22.69	3	8	8
A8BNR9	Uncharacterized protein OS=Giardia intestinalis (strain ATCC 50803 / WB	35.15	13.67	5	10	10
V6TWB6	Putative TCP-1/cpn60 chaperonin family protein OS=Giardia intestinalis O	35.01	19.17	1	1	9
A8BK61	Nop domain-containing protein OS=Giardia intestinalis (strain ATCC 5080	34.82	23.09	4	9	9
A8B2R0	Phosphatase 1 regulatory subunit, putative OS=Giardia intestinalis (strain	34.81	20.55	5	8	8
A8BBT9	Signal recognition particle OS=Giardia intestinalis (strain ATCC 50803 / W	34.79	19.44	5	9	9
A8B1Z3	Phosphoenolpyruvate carboxykinase (GTP) OS=Giardia intestinalis (strain	34.63	21.87	5	7	7
Q969C1	Kinesin-like protein (Fragment) OS=Giardia intestinalis OX=5741 GN=GIK	34.38	13.33	5	10	10
A8BC91	Ankyrin repeat protein 1 OS=Giardia intestinalis (strain ATCC 50803 / WB	34.37	15.87	5	10	10
D3KGT7	RING-type domain-containing protein OS=Giardia intestinalis (strain ATCC	34.33	15.97	3	10	10
D3KGF0	Uncharacterized protein OS=Giardia intestinalis (strain ATCC 50803 / WB	34.32	14.41	3	8	9
A8BNX5	Protein SDA1 OS=Giardia intestinalis (strain ATCC 50803 / WB clone C6)	34.21	13.90	4	13	13
A8BD41	Putative Ubiquitin protein OS=Giardia intestinalis (strain ATCC 50803 / W	34.17	28.92	5	8	8
A8BH18	Kinase, NEK OS=Giardia intestinalis (strain ATCC 50803 / WB clone C6) O	33.93	16.86	3	8	8
A0A644F9E9	PPPDE putative peptidase domain-containing protein OS=Giardia intestina	33.92	24.62	6	8	8
A0A644F3D5	Uncharacterized protein OS=Giardia intestinalis (strain ATCC 50803 / WB	33.64	15.20	2	10	10
Q9N9W6	26S proteasome ATPase subunit S4 (Fragment) OS=Giardia intestinalis O	33.61	36.44	1	1	6
A0A644F2Z7	Uncharacterized protein OS=Giardia intestinalis (strain ATCC 50803 / WB	33.54	20.47	6	10	10
A8B932	Kinase, NEK OS=Giardia intestinalis (strain ATCC 50803 / WB clone C6) O	33.46	10.43	2	5	10
A0A644FAV9	Aminoacyl-histidine dipeptidase OS=Giardia intestinalis (strain ATCC 5080	33.43	22.20	4	8	8
Q8MUV2	DNA-directed RNA polymerase subunit beta OS=Giardia intestinalis OX=5	33.38	12.53	5	11	11
A8BYQ3	Ubiquitin-protein ligase E3A OS=Giardia intestinalis (strain ATCC 50803 /	33.15	12.53	4	2	10
A0A644F872	Uncharacterized protein OS=Giardia intestinalis (strain ATCC 50803 / WB	33.13	10.44	3	9	9
A8BLA6	Nucleoside diphosphate kinase OS=Giardia intestinalis (strain ATCC 50803	33.10	23.77	5	8	8
A8B1Y6	Coatomer beta subunit OS=Giardia intestinalis (strain ATCC 50803 / WB c	33.08	15.46	5	12	12
A8B8H3	Eukaryotic translation initiation factor 3 subunit G OS=Giardia intestinalis	33.00	17.43	4	5	5
A8B488	Helicase OS=Giardia intestinalis (strain ATCC 50803 / WB clone C6) OX=1	32.99	9.92	3	10	10
A8BXX1	Eukaryotic translation initiation factor 2 beta subunit OS=Giardia intestinal	32.87	31.52	4	8	8
V6TAB2	Uncharacterized protein OS=Giardia intestinalis OX=5741 GN=DHA2_154	32.83	20.10	6	1	7
A8BBI7	Ubiquitin carboxyl-terminal hydrolase OS=Giardia intestinalis (strain ATCC	32.79	12.21	5	8	8
A8BCE5	Flagellar exit site protein OS=Giardia intestinalis (strain ATCC 50803 / WB	32.74	13.30	3	8	8
F6KZU6	Beta-giardin (Fragment) OS=Giardia intestinalis OX=5741 PE=3 SV=1 - [F	32.71	69.41	109	1	10
A8BPT7	Ankyrin repeat protein 3 OS=Giardia intestinalis (strain ATCC 50803 / WB	32.47	15.54	5	8	8
A8BFI8	Periodic tryptophan protein 2-like protein OS=Giardia intestinalis (strain A	32.34	11.26	4	8	8
A0A644F678	Kinase, NEK OS=Giardia intestinalis (strain ATCC 50803 / WB clone C6) O	32.30	12.33	6	10	10

A8BL74	Uncharacterized protein OS=Giardia intestinalis (strain ATCC 50803 / WB c	32.29	22.60	5	6	7
Q8ITF7	Alpha-7.3 giardin OS=Giardia intestinalis OX=5741 PE=4 SV=1 - [Q8ITF7_	32.23	30.85	7	2	8
A8BAR6	Alpha adaptin OS=Giardia intestinalis (strain ATCC 50803 / WB clone C6) (	32.21	14.69	5	10	10
A8BM39	Dynein intermediate chain OS=Giardia intestinalis (strain ATCC 50803 / W	32.13	17.83	5	9	9
A8BRK1	Uncharacterized protein OS=Giardia intestinalis (strain ATCC 50803 / WB c	31.75	40.74	3	8	8
A8BND4	Long chain fatty acid CoA ligase 5 OS=Giardia intestinalis (strain ATCC 50	31.48	14.30	4	5	8
A8BL07	Elongation initiation factor 5C OS=Giardia intestinalis (strain ATCC 50803 /	31.46	18.29	4	6	6
A0A644F164	Alpha-14 giardin OS=Giardia intestinalis (strain ATCC 50803 / WB clone C	31.44	31.82	6	9	9
A8B820	Ankyrin repeat protein 1 OS=Giardia intestinalis (strain ATCC 50803 / WB	31.43	16.69	4	8	8
A8BLE5	Uncharacterized protein OS=Giardia intestinalis (strain ATCC 50803 / WB c	31.35	15.98	3	8	8
E1F2J5	CCT-alpha OS=Giardia intestinalis (strain P15) OX=658858 GN=GLP15_11	31.35	21.45	5	10	10
A8BR64	Ankyrin repeat protein 1 OS=Giardia intestinalis (strain ATCC 50803 / WB	31.25	15.16	4	8	8
A8BAA9	Notchless OS=Giardia intestinalis (strain ATCC 50803 / WB clone C6) OX=	31.23	20.12	5	8	8
A8B474	Nuclear pore complex subunit OS=Giardia intestinalis (strain ATCC 50803	31.14	6.42	5	8	9
A8BGT4	Uncharacterized protein OS=Giardia intestinalis (strain ATCC 50803 / WB c	31.11	12.99	6	9	9
A8BD05	Serine/threonine-protein phosphatase 2A 55 kDa regulatory subunit B OS=	31.00	18.86	4	8	8
A0A644F2N0	Uncharacterized protein OS=Giardia intestinalis (strain ATCC 50803 / WB c	30.93	20.18	5	9	9
A8B4P5	Transducin OS=Giardia intestinalis (strain ATCC 50803 / WB clone C6) OX	30.86	18.30	5	7	7
A8B676	Uncharacterized protein OS=Giardia intestinalis (strain ATCC 50803 / WB c	30.65	14.54	4	8	8
A0A644FB52	Uncharacterized protein OS=Giardia intestinalis (strain ATCC 50803 / WB c	30.62	4.20	4	8	8
A8BJ39	Protein required for cell viability, possible role in assembly of the ribosome	30.61	26.93	5	9	9
A0A644F0T7	WD40 repeat protein OS=Giardia intestinalis (strain ATCC 50803 / WB clon	30.52	7.66	6	9	9
V6TRZ5	Uncharacterized protein OS=Giardia intestinalis OX=5741 GN=DHA2_2007	30.42	33.98	3	6	6
A8B8L6	Uncharacterized protein OS=Giardia intestinalis (strain ATCC 50803 / WB c	30.36	8.06	4	8	8
A0A644F066	Nucleotide-binding protein 35 OS=Giardia intestinalis (strain ATCC 50803 /	30.30	31.06	4	1	6
A8BMJ2	Uncharacterized protein OS=Giardia intestinalis (strain ATCC 50803 / WB c	30.21	6.30	3	7	7
A8B686	Uncharacterized protein OS=Giardia intestinalis (strain ATCC 50803 / WB c	30.12	14.57	4	9	9
A8BN35	Kinesin-like protein OS=Giardia intestinalis (strain ATCC 50803 / WB clone	29.76	13.98	6	6	7
A8BP66	Las1-like protein OS=Giardia intestinalis (strain ATCC 50803 / WB clone C	29.64	23.89	8	7	7
A8BCE6	Uncharacterized protein OS=Giardia intestinalis (strain ATCC 50803 / WB c	29.63	13.64	4	6	6
Q8WR13	Hybrid-cluster protein OS=Giardia intestinalis OX=5741 GN=HCP PE=2 SV	29.62	14.93	4	3	6
A8B4R5	Uncharacterized protein OS=Giardia intestinalis (strain ATCC 50803 / WB c	29.55	10.57	3	7	10
A8BN51	26S proteasome non-ATPase regulatory subunit 2 OS=Giardia intestinalis	29.52	11.04	4	10	10
A8BNL1	ZPR1 OS=Giardia intestinalis (strain ATCC 50803 / WB clone C6) OX=1849	29.48	21.01	4	8	8
D3KI74	Uncharacterized protein OS=Giardia intestinalis (strain ATCC 50803 / WB c	29.31	9.43	3	8	8
A8BIJ2	Eukaryotic translation initiation factor 2 alpha subunit OS=Giardia intestina	29.26	27.54	7	7	7
E1F554	Ribosomal protein L30 OS=Giardia intestinalis (strain P15) OX=658858 GN	29.20	60.55	4	5	5
A0A644F3D3	Uncharacterized protein OS=Giardia intestinalis (strain ATCC 50803 / WB c	29.18	6.53	4	10	10
A8BRU2	Uncharacterized protein OS=Giardia intestinalis (strain ATCC 50803 / WB c	29.17	10.97	4	8	9

E1EVJ6	Ubiquitin-protein ligase (E3) OS=Giardia intestinalis (strain P15) OX=6588	29.14	12.53	4	1	9
A8BIY8	LEK1 OS=Giardia intestinalis (strain ATCC 50803 / WB clone C6) OX=1849	28.98	16.48	5	8	8
A8B6J5	Kinase, NEK OS=Giardia intestinalis (strain ATCC 50803 / WB clone C6) O)	28.91	20.83	4	9	9
V6TIU4	Putative Brix domain protein OS=Giardia intestinalis OX=5741 GN=DHA2_	28.89	25.14	4	7	7
A8BRL1	Vacuolar ATP synthase subunit D OS=Giardia intestinalis (strain ATCC 508	28.82	29.48	4	7	7
A0A644EYT2	Uncharacterized protein OS=Giardia intestinalis (strain ATCC 50803 / WB	28.81	5.38	6	9	9
Q9U020	Serine/threonine protein kinase PKB OS=Giardia intestinalis OX=5741 PE=	28.73	19.80	4	6	6
Q06Z47	Kinesin-like protein OS=Giardia intestinalis OX=5741 PE=3 SV=1 - [Q06Z4	28.70	18.49	5	8	8
A8BIM7	Uncharacterized protein OS=Giardia intestinalis (strain ATCC 50803 / WB	28.67	22.45	2	7	7
A8BC13	Phosphomannomutase-2 OS=Giardia intestinalis (strain ATCC 50803 / WB	28.67	22.01	3	7	7
A0A644F5S5	Uncharacterized protein OS=Giardia intestinalis (strain ATCC 50803 / WB	28.60	5.86	4	7	7
Q9BKJ3	Fe-hydrogenase OS=Giardia intestinalis OX=5741 PE=3 SV=1 - [Q9BKJ3_	28.57	20.46	4	7	7
A8BYU8	Uncharacterized protein OS=Giardia intestinalis (strain ATCC 50803 / WB	28.56	9.19	3	9	9
A8BKM0	Sec1 family protein OS=Giardia intestinalis (strain ATCC 50803 / WB clone	28.50	16.22	5	8	8
A8BQ53	Uncharacterized protein OS=Giardia intestinalis (strain ATCC 50803 / WB	28.43	5.63	4	9	9
E1EY14	Cytosolic Fe-S cluster assembly factor NUBP1 homolog OS=Giardia intestir	28.41	31.37	3	1	6
A8B7R6	Phosphoprotein phosphatase 2A regulatory subunit OS=Giardia intestinalis	28.34	15.58	4	8	8
A0A644F8X8	Putative Manganese-dependent inorganic pyrophosphatase OS=Giardia int	28.17	11.19	5	6	6
V6TKQ2	Uncharacterized protein OS=Giardia intestinalis OX=5741 GN=DHA2_1532	28.15	19.72	5	7	7
A8BCJ6	Uncharacterized protein OS=Giardia intestinalis (strain ATCC 50803 / WB	28.12	8.09	3	8	8
A8B748	Serine/threonine-protein phosphatase OS=Giardia intestinalis (strain ATCC	28.08	32.90	4	9	10
A8BU43	Non-specific serine/threonine protein kinase OS=Giardia intestinalis (strain	28.01	14.93	4	7	7
A8BS78	WD-40 repeat protein OS=Giardia intestinalis (strain ATCC 50803 / WB clo	27.97	11.24	4	9	9
A8BB01	Eukaryotic rRNA processing protein EBP2 OS=Giardia intestinalis (strain AT	27.96	32.62	4	6	6
A8BFK8	Spindle pole body component OS=Giardia intestinalis (strain ATCC 50803 /	27.95	14.35	5	9	9
A8BB52	GTPase SAR1 family protein OS=Giardia intestinalis (strain ATCC 50803 / '	27.83	45.83	5	7	7
A0A644F483	VID27 cytoplasmic protein OS=Giardia intestinalis (strain ATCC 50803 / W	27.82	13.26	5	6	6
A8BB92	RING-type domain-containing protein OS=Giardia intestinalis (strain ATCC	27.81	14.11	4	9	9
A8BY75	Methionine--tRNA ligase OS=Giardia intestinalis (strain ATCC 50803 / WB	27.61	18.90	3	7	7
D3KGV4	Uncharacterized protein OS=Giardia intestinalis (strain ATCC 50803 / WB	27.57	6.59	5	8	8
A8B8M9	Phosphoinositide-3-kinase, class 3 OS=Giardia intestinalis (strain ATCC 50	27.56	6.96	4	8	8
C6LUI0	Intraflagellar transport protein component IFT74/72 OS=Giardia intestinal	27.47	21.43	2	1	8
A8B4L6	Kinase, NEK OS=Giardia intestinalis (strain ATCC 50803 / WB clone C6) O)	27.46	8.45	4	7	7
A8B9S8	G1 to S phase transition protein 1, putative OS=Giardia intestinalis (strain	27.36	19.57	6	6	6
A8BAY5	Uncharacterized protein OS=Giardia intestinalis (strain ATCC 50803 / WB	27.35	9.65	3	8	8
A8B4K9	Uncharacterized protein OS=Giardia intestinalis (strain ATCC 50803 / WB	27.35	8.87	3	8	8
A0A644F8Q4	Uncharacterized protein OS=Giardia intestinalis (strain ATCC 50803 / WB	27.32	10.86	4	11	11
A8BLG8	Ubiquitin carboxyl-terminal hydrolase 14 OS=Giardia intestinalis (strain AT	27.26	20.26	5	7	7
A8BQ40	Protein phosphatase 2A regulatory subunit, putative OS=Giardia intestinal	27.24	12.22	5	8	8

A8BP13	Alanine aminotransferase, putative OS=Giardia intestinalis (strain ATCC 50	27.24	14.61	3	7	7
A8B6Q2	DNA-dependent ATPase, putative OS=Giardia intestinalis (strain ATCC 508	27.21	7.60	5	8	8
A8B2N6	Uncharacterized protein OS=Giardia intestinalis (strain ATCC 50803 / WB	27.04	13.22	5	8	8
A8BUP1	Coiled-coil protein OS=Giardia intestinalis (strain ATCC 50803 / WB clone	26.98	8.33	5	8	8
Q4VPQ5	Alpha-2 giardin OS=Giardia intestinalis OX=5741 PE=4 SV=1 - [Q4VPQ5_	26.92	17.23	5	1	6
A8BSR3	26S proteasome non-ATPase regulatory subunit 7 OS=Giardia intestinalis	26.85	35.31	4	5	5
A8B2U8	Kinase, NEK OS=Giardia intestinalis (strain ATCC 50803 / WB clone C6) O	26.67	27.10	2	6	6
A8BMY9	Transducin OS=Giardia intestinalis (strain ATCC 50803 / WB clone C6) OX	26.65	12.11	3	6	6
A8BC70	Sec24-like OS=Giardia intestinalis (strain ATCC 50803 / WB clone C6) OX=	26.54	8.75	4	7	7
V6T807	Nucleolar protein 16 OS=Giardia intestinalis OX=5741 GN=DHA2_8002 PE	26.53	31.65	3	6	6
A0A644F2N5	E3 ubiquitin protein ligase OS=Giardia intestinalis (strain ATCC 50803 / W	26.41	15.51	6	8	8
A8BAX0	Uncharacterized protein OS=Giardia intestinalis (strain ATCC 50803 / WB	26.34	22.58	3	6	6
A0A644F7M7	Spindle pole body component OS=Giardia intestinalis (strain ATCC 50803	26.29	10.09	9	7	7
A8B6Z2	Putative Zinc finger protein OS=Giardia intestinalis (strain ATCC 50803 / W	26.04	9.71	3	8	8
A8BZI8	Kinase, NEK OS=Giardia intestinalis (strain ATCC 50803 / WB clone C6) O	26.03	28.05	4	7	7
A8B491	Uncharacterized protein OS=Giardia intestinalis (strain ATCC 50803 / WB	25.96	10.59	3	8	8
A8BEI4	Uncharacterized protein OS=Giardia intestinalis (strain ATCC 50803 / WB	25.94	4.00	5	8	8
A8BWB0	Uncharacterized protein OS=Giardia intestinalis (strain ATCC 50803 / WB	25.92	12.04	2	7	7
A8B908	Protein tyrosine phosphatase OS=Giardia intestinalis (strain ATCC 50803 /	25.90	14.80	4	7	7
A8BGM3	Uncharacterized protein OS=Giardia intestinalis (strain ATCC 50803 / WB	25.81	29.71	5	8	8
A8BHY1	Ankyrin repeat protein 1 OS=Giardia intestinalis (strain ATCC 50803 / WB	25.78	10.84	5	8	8
A8B7V7	Ankyrin repeat protein 1 OS=Giardia intestinalis (strain ATCC 50803 / WB	25.72	19.10	3	6	7
A8BGY2	Importin-like protein OS=Giardia intestinalis (strain ATCC 50803 / WB clon	25.53	9.00	5	9	9
Q964G2	Phosphomannomutase-2 OS=Giardia intestinalis OX=5741 PE=3 SV=1 - [	25.52	18.02	3	7	7
A8B3C3	Putative serine esterase OS=Giardia intestinalis (strain ATCC 50803 / WB	25.52	11.21	4	7	7
A0A644FA19	Ribosomal protein S29e OS=Giardia intestinalis (strain ATCC 50803 / WB	25.47	41.18	4	4	4
A8B7R7	DNA-directed RNA polymerase subunit OS=Giardia intestinalis (strain ATC	25.47	5.23	4	8	8
A8BXJ0	Uncharacterized protein OS=Giardia intestinalis (strain ATCC 50803 / WB	25.38	11.55	3	6	6
A0A644F348	Kinase, NEK OS=Giardia intestinalis (strain ATCC 50803 / WB clone C6) O	25.36	16.28	5	8	8
A8B9A6	Major facilitator superfamily transporter OS=Giardia intestinalis (strain AT	25.35	12.35	4	2	5
A8BA94	Uncharacterized protein OS=Giardia intestinalis (strain ATCC 50803 / WB	25.22	10.78	5	7	7
A8B4T4	Eukaryotic translation initiation factor 5 OS=Giardia intestinalis (strain AT	25.19	22.55	4	6	6
A0A644F722	Alcohol dehydrogenase 3 OS=Giardia intestinalis (strain ATCC 50803 / WB	25.06	18.47	6	5	5
V6TL86	Serine/threonine protein kinase OS=Giardia intestinalis OX=5741 GN=DH	25.05	23.03	3	9	9
D3KHH9	Uncharacterized protein OS=Giardia intestinalis (strain ATCC 50803 / WB	24.95	13.23	5	7	7
V6TAR5	Glyceraldehyde-3-phosphate dehydrogenase OS=Giardia intestinalis OX=5	24.90	30.36	5	7	7
A8BGG4	Leucine-rich repeat protein 1 virus receptor protein OS=Giardia intestinalis	24.79	12.68	6	8	8
A8BHM5	Kinesin-associated protein OS=Giardia intestinalis (strain ATCC 50803 / W	24.75	14.97	4	8	8
V6TA14	Putative WD-repeat family protein OS=Giardia intestinalis OX=5741 GN=D	24.72	17.07	3	10	10

A8B8P4	Uncharacterized protein OS=Giardia intestinalis (strain ATCC 50803 / WB	24.54	5.44	4	7	7
A8B915	Vacuolar protein sorting 29 OS=Giardia intestinalis (strain ATCC 50803 / W	24.53	12.61	5	5	5
A8BWM5	6-phosphogluconolactonase/Glucose-6-phosphate 1-dehydrogenase OS=G	24.50	12.26	5	8	8
A8BBN4	Uncharacterized protein OS=Giardia intestinalis (strain ATCC 50803 / WB	24.50	12.13	3	5	5
A0A644FBH1	60S ribosome subunit biogenesis protein NIP7 homolog OS=Giardia intest	24.50	35.56	5	6	6
E1EVU7	Uncharacterized protein OS=Giardia intestinalis (strain P15) OX=658858 G	24.47	6.43	2	1	2
A8B518	Kinesin-3 OS=Giardia intestinalis (strain ATCC 50803 / WB clone C6) OX=	24.45	8.22	5	2	8
A8BWW5	Kinase, NEK OS=Giardia intestinalis (strain ATCC 50803 / WB clone C6) O	24.43	17.51	19	4	8
A8BAB3	26S protease regulatory subunit 7 OS=Giardia intestinalis (strain ATCC 50	24.39	14.21	5	7	7
A8BB91	Kinesin-like protein OS=Giardia intestinalis (strain ATCC 50803 / WB clone	24.38	13.71	5	6	7
A8B2S4	Kinase, NEK OS=Giardia intestinalis (strain ATCC 50803 / WB clone C6) O	24.32	29.27	5	6	6
A8B397	Uncharacterized protein OS=Giardia intestinalis (strain ATCC 50803 / WB	24.30	14.49	5	6	6
A8B721	Microtubule associated protein OS=Giardia intestinalis (strain ATCC 50803	24.12	14.87	4	7	7
D3KHN3	Uncharacterized protein OS=Giardia intestinalis (strain ATCC 50803 / WB	24.10	7.86	4	8	8
A8BHA5	CBF/Mak21 family protein OS=Giardia intestinalis (strain ATCC 50803 / W	24.03	13.26	3	6	6
E1EZ64	Uridine kinase OS=Giardia intestinalis (strain P15) OX=658858 GN=GLP15	23.98	19.24	5	7	8
A8RHT9	Glutamate dehydrogenase (Fragment) OS=Giardia intestinalis OX=5741 G	23.97	26.41	552	7	7
A8B860	Kinase, NEK OS=Giardia intestinalis (strain ATCC 50803 / WB clone C6) O	23.89	14.30	4	6	6
A8BN10	Uncharacterized protein OS=Giardia intestinalis (strain ATCC 50803 / WB	23.83	24.00	3	7	7
A8BXS3	N-acetyltransferase-like protein OS=Giardia intestinalis (strain ATCC 5080	23.81	8.49	5	7	8
A0A644EY25	Uncharacterized protein OS=Giardia intestinalis (strain ATCC 50803 / WB	23.79	8.87	4	7	7
A8BS58	GTP-binding protein OS=Giardia intestinalis (strain ATCC 50803 / WB clon	23.69	17.64	4	8	8
A8B9Q0	Structural maintenance of chromosomes protein OS=Giardia intestinalis (s	23.51	7.87	5	8	8
A0A1M4M195	Beta-giardin (Fragment) OS=Giardia intestinalis OX=5741 GN=bg PE=3 S	23.48	65.22	56	1	8
A8BUY9	Uncharacterized protein OS=Giardia intestinalis (strain ATCC 50803 / WB	23.44	24.44	3	5	5
V6TE99	Dynein-like protein OS=Giardia intestinalis OX=5741 GN=DHA2_4463 PE=	23.43	44.92	2	7	7
A8B1U4	IFT complex B OS=Giardia intestinalis (strain ATCC 50803 / WB clone C6)	23.40	21.19	3	7	7
A8BGE6	Uncharacterized protein OS=Giardia intestinalis (strain ATCC 50803 / WB	23.39	3.35	4	5	6
A8BCR3	Putative U3 small nucleolar ribonucleoprotein IMP4 OS=Giardia intestinalis	23.36	27.92	4	6	6
V6TF75	Uncharacterized protein OS=Giardia intestinalis OX=5741 GN=DHA2_153	23.35	32.52	4	5	5
D3KIC1	Uncharacterized protein OS=Giardia intestinalis (strain ATCC 50803 / WB	23.26	6.74	5	7	7
E1EYM0	Kinase, NEK OS=Giardia intestinalis (strain P15) OX=658858 GN=GLP15_	23.23	4.65	2	1	6
A8B8T1	CDC19 OS=Giardia intestinalis (strain ATCC 50803 / WB clone C6) OX=18	23.13	6.76	5	8	8
A8B8E9	Major facilitator superfamily transporter OS=Giardia intestinalis (strain AT	23.11	12.20	7	1	4
A8B6X4	Dynein intermediate chain OS=Giardia intestinalis (strain ATCC 50803 / W	23.07	12.69	4	7	7
A8BYT1	26S proteasome non-ATPase regulatory subunit 6 OS=Giardia intestinalis	23.05	24.26	3	8	8
E2RTU0	Mitogen-activated protein kinase OS=Giardia intestinalis (strain ATCC 508	23.03	21.82	3	6	6
A0A644F3V1	Aspartyl-tRNA synthetase OS=Giardia intestinalis (strain ATCC 50803 / WB	23.03	20.87	4	7	7
A8BFH7	NPL4-like protein OS=Giardia intestinalis (strain ATCC 50803 / WB clone C	22.99	15.18	4	6	6

D3KGQ3	SWIRM domain-containing protein OS=Giardia intestinalis (strain ATCC 50	22.95	7.38	3	6	6
E1EWQ7	Kinase, ULK OS=Giardia intestinalis (strain P15) OX=658858 GN=GLP15_3	22.94	5.18	1	1	8
A8BB03	Uncharacterized protein OS=Giardia intestinalis (strain ATCC 50803 / WB c	22.84	4.18	5	7	7
A0A644F510	Methionine aminopeptidase OS=Giardia intestinalis (strain ATCC 50803 / V	22.76	18.94	6	5	5
A8BEZ3	Uncharacterized protein OS=Giardia intestinalis (strain ATCC 50803 / WB c	22.65	9.92	2	8	8
E1F6J9	Kinase, NEK OS=Giardia intestinalis (strain P15) OX=658858 GN=GLP15_4	22.64	12.24	5	5	5
A8BB33	Bystin OS=Giardia intestinalis (strain ATCC 50803 / WB clone C6) OX=184	22.58	20.88	5	7	7
D3KHB8	Uncharacterized protein OS=Giardia intestinalis (strain ATCC 50803 / WB c	22.57	5.68	5	8	8
A0A644F8T4	Uncharacterized protein OS=Giardia intestinalis (strain ATCC 50803 / WB c	22.51	20.08	3	6	6
A8B3B2	Uncharacterized protein OS=Giardia intestinalis (strain ATCC 50803 / WB c	22.41	14.65	5	7	7
V6TPF8	Proteasome endopeptidase complex OS=Giardia intestinalis OX=5741 GN=	22.39	40.00	3	2	7
A8BEY0	Serine/Threonine-protein kinase pkwA, putative OS=Giardia intestinalis (st	22.34	17.87	4	6	6
A0A644EZT4	TFIIS N-terminal domain-containing protein OS=Giardia intestinalis (strain	22.30	23.36	4	5	5
A8B9B7	Alpha-SNAP, putative OS=Giardia intestinalis (strain ATCC 50803 / WB clo	22.19	20.13	5	5	5
A8BCX5	p60 Katanin OS=Giardia intestinalis (strain ATCC 50803 / WB clone C6) O)	22.19	16.20	5	8	8
A8BBX8	Mitotic control protein dis3 OS=Giardia intestinalis (strain ATCC 50803 / W	21.94	7.30	11	8	8
A0A644F5S4	Uncharacterized protein OS=Giardia intestinalis (strain ATCC 50803 / WB c	21.81	16.53	6	6	6
V6TL88	5'-AMP-activated protein kinase, gamma-1 subunit OS=Giardia intestinalis	21.78	15.82	3	4	4
A8B2X2	L-serine dehydratase OS=Giardia intestinalis (strain ATCC 50803 / WB clor	21.70	12.40	5	4	4
A8BIE4	Katanin p60 ATPase-containing subunit A1 OS=Giardia intestinalis (strain /	21.59	16.11	5	7	7
A0A644F3X5	Cilia- and flagella-associated protein 206 OS=Giardia intestinalis (strain AT	21.59	8.13	4	2	5
A8BW40	Ankyrin repeat protein 1 OS=Giardia intestinalis (strain ATCC 50803 / WB	21.57	5.59	2	5	5
A8BNG7	DRAP deaminase OS=Giardia intestinalis (strain ATCC 50803 / WB clone C	21.50	14.61	4	6	6
V6TGX4	Serine/threonine protein kinase OS=Giardia intestinalis OX=5741 GN=DH/	21.49	21.21	3	5	5
A8BP53	Kinase, STE STE20 OS=Giardia intestinalis (strain ATCC 50803 / WB clone	21.47	21.99	3	4	6
A8BA67	Ankyrin repeat protein 1 OS=Giardia intestinalis (strain ATCC 50803 / WB	21.43	12.01	3	7	7
A8BVD3	Transketolase OS=Giardia intestinalis (strain ATCC 50803 / WB clone C6)	21.21	13.21	5	6	6
A8B3M2	CTP synthase OS=Giardia intestinalis (strain ATCC 50803 / WB clone C6) (	21.19	11.06	5	8	8
A8BPD8	WD40 repeat protein OS=Giardia intestinalis (strain ATCC 50803 / WB clor	21.17	15.98	4	6	6
A0A644F5X5	Ankyrin repeat protein 1 OS=Giardia intestinalis (strain ATCC 50803 / WB	21.16	5.72	6	7	7
A8BH74	Uncharacterized protein OS=Giardia intestinalis (strain ATCC 50803 / WB c	21.13	3.49	2	5	5
C6LR97	DNA polymerase OS=Giardia intestinalis (strain ATCC 50581 / GS clone H7	21.09	7.16	4	1	7
A0A644F385	Uncharacterized protein OS=Giardia intestinalis (strain ATCC 50803 / WB c	21.01	4.49	5	5	5
A8BNS7	Ankyrin repeat protein 1 OS=Giardia intestinalis (strain ATCC 50803 / WB	21.00	6.61	4	5	5
A8B8S5	Kinase, NEK OS=Giardia intestinalis (strain ATCC 50803 / WB clone C6) O)	20.99	5.56	3	5	5
A8BAU3	Kinase, VPS15 OS=Giardia intestinalis (strain ATCC 50803 / WB clone C6)	20.98	4.21	5	7	7
Q968V7	Glucose-6-phosphate isomerase OS=Giardia intestinalis OX=5741 GN=GP:	20.96	15.41	5	5	6
A8BFQ5	Ankyrin repeat protein 1 OS=Giardia intestinalis (strain ATCC 50803 / WB	20.96	10.93	3	6	6
A8B5Y5	DNA replication licensing factor MCM7 OS=Giardia intestinalis (strain ATC(	20.94	9.02	5	6	6

A8BNL6	Kinase, NEK OS=Giardia intestinalis (strain ATCC 50803 / WB clone C6) O)	20.94	5.90	4	4	4
A8BDS9	Glycine-rich protein OS=Giardia intestinalis (strain ATCC 50803 / WB clone	20.89	13.64	5	6	6
A8B8T7	Uncharacterized protein OS=Giardia intestinalis (strain ATCC 50803 / WB (	20.89	9.50	5	6	6
A8BNI1	Metalloendopeptidase OS=Giardia intestinalis (strain ATCC 50803 / WB clo	20.78	7.42	4	7	7
A0A644F136	Threonylcarbamoyl-AMP synthase OS=Giardia intestinalis (strain ATCC 508	20.77	21.14	4	6	6
A0A644F408	Kinase, NEK OS=Giardia intestinalis (strain ATCC 50803 / WB clone C6) O)	20.76	10.04	6	3	3
E1F4D4	Uncharacterized protein OS=Giardia intestinalis (strain P15) OX=658858 G	20.75	10.41	5	7	7
A8BJJ3	Putative Transcription factor OS=Giardia intestinalis (strain ATCC 50803 /	20.72	9.63	3	7	7
A8B619	Ankyrin repeat protein 1 OS=Giardia intestinalis (strain ATCC 50803 / WB	20.67	9.73	5	6	6
A8BAM0	Small glutamine-rich tetratricopeptide repeat-containing protein OS=Giard	20.66	28.33	5	5	5
A0A644F7I9	Uncharacterized protein OS=Giardia intestinalis (strain ATCC 50803 / WB (	20.61	4.51	8	7	7
A8B6X6	Uncharacterized protein OS=Giardia intestinalis (strain ATCC 50803 / WB (	20.55	10.66	2	5	5
A8BK93	Putative WD-repeat membrane protein OS=Giardia intestinalis (strain ATC	20.55	7.40	5	7	7
A0A644F3C7	Uncharacterized protein OS=Giardia intestinalis (strain ATCC 50803 / WB (	20.54	5.81	5	8	8
A8BJ67	Importin N-terminal domain-containing protein OS=Giardia intestinalis (str	20.53	7.85	4	7	7
Q24983	Protein phosphatase 2C homolog OS=Giardia intestinalis OX=5741 PE=3 S	20.53	20.83	5	5	5
V6THS9	Serine/threonine protein kinase OS=Giardia intestinalis OX=5741 GN=DHA	20.43	4.62	19	1	3
A0A644F598	Kinase, NEK OS=Giardia intestinalis (strain ATCC 50803 / WB clone C6) O)	20.42	5.95	5	5	5
A8BP90	Kinase, NEK OS=Giardia intestinalis (strain ATCC 50803 / WB clone C6) O)	20.39	11.87	1	5	6
A8B6S1	Utp14 protein OS=Giardia intestinalis (strain ATCC 50803 / WB clone C6) (	20.31	11.28	4	6	6
A8BNP4	Uncharacterized protein OS=Giardia intestinalis (strain ATCC 50803 / WB (	20.21	24.24	3	5	5
A8BNT2	Spindle pole protein, putative OS=Giardia intestinalis (strain ATCC 50803 /	20.19	12.27	6	5	5
A8BHB1	Spastin OS=Giardia intestinalis (strain ATCC 50803 / WB clone C6) OX=18	20.17	7.51	5	7	7
A8BPK8	Serine/threonine-protein kinase PLK OS=Giardia intestinalis (strain ATCC 5	20.16	11.36	6	6	7
D3KH93	Uncharacterized protein OS=Giardia intestinalis (strain ATCC 50803 / WB (	20.15	13.88	4	5	5
D3KGM9	Uncharacterized protein OS=Giardia intestinalis (strain ATCC 50803 / WB (	20.06	2.11	2	6	6
A8BJP5	Uncharacterized protein OS=Giardia intestinalis (strain ATCC 50803 / WB (	20.04	6.76	1	6	6
A8BWV4	DNA ligase OS=Giardia intestinalis (strain ATCC 50803 / WB clone C6) OX	20.00	10.12	4	5	5
A8BA51	Nin one binding protein-like protein OS=Giardia intestinalis (strain ATCC 5	20.00	18.22	5	8	8
A8B7N5	Coiled-coil protein OS=Giardia intestinalis (strain ATCC 50803 / WB clone (	20.00	5.80	4	6	6
D3KHX2	Uncharacterized protein OS=Giardia intestinalis (strain ATCC 50803 / WB (	19.92	4.91	2	5	5
A8BMZ5	Uncharacterized protein OS=Giardia intestinalis (strain ATCC 50803 / WB (	19.92	5.60	4	5	5
D3KGW3	Ankyrin repeat protein 1 OS=Giardia intestinalis (strain ATCC 50803 / WB	19.90	11.15	5	7	7
A8BS30	Dual specificity phosphatase, catalytic OS=Giardia intestinalis (strain ATCC	19.90	12.87	4	5	5
A0A644F5E8	Kinase, NEK OS=Giardia intestinalis (strain ATCC 50803 / WB clone C6) O)	19.89	8.99	3	5	5
A8B9C8	DNA-directed RNA polymerase subunit D OS=Giardia intestinalis (strain AT	19.89	23.71	5	6	6
V6TG85	Ankyrin repeat protein OS=Giardia intestinalis OX=5741 GN=DHA2_16227	19.87	17.11	3	6	6
A8B2R9	WD-repeat protein BING4 OS=Giardia intestinalis (strain ATCC 50803 / WI	19.81	13.70	5	6	6
A8BU45	Protein phosphatases PP1 regulatory subunit SDS22 OS=Giardia intestinal	19.78	12.82	5	5	5

A8BRP1	Coatomer delta subunit OS=Giardia intestinalis (strain ATCC 50803 / WB c	19.77	17.22	1	4	4
E1EYM4	G2/mitotic-specific cyclin B OS=Giardia intestinalis (strain P15) OX=65885	19.70	18.48	3	5	5
E1F6H7	Uncharacterized protein OS=Giardia intestinalis (strain P15) OX=658858 G	19.70	4.23	1	1	4
A8BRG3	Sperm tail domain-containing protein OS=Giardia intestinalis (strain ATCC	19.69	9.00	4	6	6
A0A644F7R8	Kinase, CMGC CDKL OS=Giardia intestinalis (strain ATCC 50803 / WB clon	19.68	14.76	5	4	5
A8BEQ9	Putative replication factor-a protein OS=Giardia intestinalis (strain ATCC 5	19.64	16.36	2	4	6
A8BDI0	E04F6.2 like protein OS=Giardia intestinalis (strain ATCC 50803 / WB clon	19.47	32.91	4	6	6
A8B4B6	GPN-loop GTPase OS=Giardia intestinalis (strain ATCC 50803 / WB clone C	19.45	16.80	5	4	4
A8BT34	AAA-type ATPase OS=Giardia intestinalis (strain ATCC 50803 / WB clone C	19.45	8.22	3	5	5
A8BXZ6	Uncharacterized protein OS=Giardia intestinalis (strain ATCC 50803 / WB c	19.39	8.05	2	7	7
A8BMN2	Putative Transcriptional activator OS=Giardia intestinalis (strain ATCC 508	19.37	4.79	4	6	7
A8BRJ7	Uncharacterized protein OS=Giardia intestinalis (strain ATCC 50803 / WB c	19.34	10.94	4	5	5
A8BSJ2	Dynein light chain OS=Giardia intestinalis (strain ATCC 50803 / WB clone C	19.34	40.00	4	5	5
A8BB10	Degreening related gene dee76 protein OS=Giardia intestinalis (strain ATC	19.30	19.88	5	5	5
A8BHF4	KH domain-containing protein OS=Giardia intestinalis (strain ATCC 50803	19.29	9.08	3	6	6
A8BBR2	Replication factor C, sub 2 OS=Giardia intestinalis (strain ATCC 50803 / W	19.27	17.66	5	5	5
V6TGD6	ATPase ASNA1 homolog OS=Giardia intestinalis OX=5741 GN=DHA2_795	19.27	28.53	4	5	5
A0A644F929	Ankyrin repeat protein 1 OS=Giardia intestinalis (strain ATCC 50803 / WB	19.17	26.83	5	5	5
V6TAR6	Serine/threonine protein kinase (Fragment) OS=Giardia intestinalis OX=57	19.16	8.57	18	1	3
A8BAI4	Putative U3 small nucleolar ribonucleoprotein MPP10 OS=Giardia intestina	19.09	18.18	4	5	5
Q9GQI5	Alanine--tRNA ligase OS=Giardia intestinalis OX=5741 GN=alaS PE=3 SV=	19.08	8.49	6	5	5
D3KHQ9	Uncharacterized protein OS=Giardia intestinalis (strain ATCC 50803 / WB c	19.06	15.68	4	6	6
A8BGN4	Uncharacterized protein OS=Giardia intestinalis (strain ATCC 50803 / WB c	19.03	42.94	5	6	6
D3KHR6	Uncharacterized protein OS=Giardia intestinalis (strain ATCC 50803 / WB c	18.94	3.82	3	5	6
A8BG44	Calcineurin-like phosphoesterase OS=Giardia intestinalis (strain ATCC 508	18.92	17.26	3	6	6
A8B6S6	Uncharacterized protein OS=Giardia intestinalis (strain ATCC 50803 / WB c	18.92	11.89	3	4	4
A8B6W3	SRP GTPase OS=Giardia intestinalis (strain ATCC 50803 / WB clone C6) O	18.88	17.74	4	7	7
D3KGT3	Dopey_N domain-containing protein OS=Giardia intestinalis (strain ATCC 5	18.76	3.48	3	5	5
Q964G4	Alpha-1,4 glucan phosphorylase OS=Giardia intestinalis OX=5741 PE=3 SV	18.72	8.33	5	6	6
A8BW38	Kinase, CMGC GSK OS=Giardia intestinalis (strain ATCC 50803 / WB clone	18.68	15.43	4	5	5
A8BS34	Actin OS=Giardia intestinalis (strain ATCC 50803 / WB clone C6) OX=184	18.63	9.85	5	3	3
A8BZ22	Translation elongation factor OS=Giardia intestinalis (strain ATCC 50803 /	18.61	28.64	5	5	5
A8B7M9	MYND finger domain-containing protein OS=Giardia intestinalis (strain ATC	18.57	13.96	2	5	5
A8BB85	Carbamate kinase OS=Giardia intestinalis (strain ATCC 50803 / WB clone C	18.56	25.32	11	7	7
A0A644F653	Non-specific serine/threonine protein kinase OS=Giardia intestinalis (strain	18.52	18.18	5	6	6
A8BVD1	Axonemal p66.0 OS=Giardia intestinalis (strain ATCC 50803 / WB clone C6	18.41	11.41	4	5	5
A8BML4	Homing endonuclease OS=Giardia intestinalis (strain ATCC 50803 / WB clo	18.41	12.27	4	5	5
A8B5H9	Glycerophosphocholine phosphodiesterase OS=Giardia intestinalis (strain A	18.35	10.99	2	5	5
V6TE08	Protein kinase, catalytic domain OS=Giardia intestinalis OX=5741 GN=DHA	18.26	19.70	2	7	7

A0A644F7J4	Kinase, AGC OS=Giardia intestinalis (strain ATCC 50803 / WB clone C6) O	18.24	10.19	6	4	4
V6TJ92	cAMP-dependent protein kinase, dimerization/docking domain protein OS=	18.18	29.13	3	4	4
A8B2T3	Uncharacterized protein OS=Giardia intestinalis (strain ATCC 50803 / WB	18.18	3.38	4	5	5
A8B3E2	Replication factor C, subunit 3 OS=Giardia intestinalis (strain ATCC 50803	18.14	20.87	4	4	4
A8B9D3	Ankyrin repeat protein 2 OS=Giardia intestinalis (strain ATCC 50803 / WB	18.12	6.80	3	3	4
A0A644EZF1	Uncharacterized protein OS=Giardia intestinalis (strain ATCC 50803 / WB	18.07	4.09	4	4	4
A8B666	Uncharacterized protein OS=Giardia intestinalis (strain ATCC 50803 / WB	17.95	8.44	4	7	7
A8B772	Uncharacterized protein OS=Giardia intestinalis (strain ATCC 50803 / WB	17.80	10.94	3	2	2
P92128	H(+)-transporting two-sector ATPase OS=Giardia intestinalis OX=5741 PE	17.75	10.40	5	6	6
A0A644FA15	Uncharacterized protein OS=Giardia intestinalis (strain ATCC 50803 / WB	17.74	4.32	3	6	6
A8BUQ3	Uncharacterized protein OS=Giardia intestinalis (strain ATCC 50803 / WB	17.71	6.73	3	3	4
D3KGB7	Uncharacterized protein OS=Giardia intestinalis (strain ATCC 50803 / WB	17.58	11.15	5	5	5
A8BH59	Vacuolar protein sorting 25 OS=Giardia intestinalis (strain ATCC 50803 / V	17.54	27.03	4	5	5
A8BFL3	Peter pan protein OS=Giardia intestinalis (strain ATCC 50803 / WB clone C	17.49	15.34	5	4	4
A8B7C8	Spindle pole protein, putative OS=Giardia intestinalis (strain ATCC 50803 /	17.44	16.71	5	4	4
A8BJW7	Putative U3 small nucleolar RNA-associated protein 6 OS=Giardia intestina	17.42	14.32	3	5	5
E1F0P0	Calmodulin OS=Giardia intestinalis (strain P15) OX=658858 GN=GLP15_2	17.39	28.10	1	4	4
E1EVJ7	Kinase, AGC PKA OS=Giardia intestinalis (strain P15) OX=658858 GN=GLF	17.37	12.80	3	4	4
A8BIJ7	Uncharacterized protein OS=Giardia intestinalis (strain ATCC 50803 / WB	17.32	19.53	4	4	4
A8BVF2	Kinase, NEK OS=Giardia intestinalis (strain ATCC 50803 / WB clone C6) O	17.27	6.44	5	5	5
A8B961	Uncharacterized protein OS=Giardia intestinalis (strain ATCC 50803 / WB	17.27	21.05	4	4	4
Q06Z46	EB1 OS=Giardia intestinalis OX=5741 GN=EB1 PE=3 SV=1 - [Q06Z46_GI	17.24	26.47	5	6	6
A8B3D5	Ankyrin repeat protein 1 OS=Giardia intestinalis (strain ATCC 50803 / WB	17.21	23.73	3	3	3
A8BAC4	Cell division control protein 48 OS=Giardia intestinalis (strain ATCC 50803	17.20	7.67	2	4	4
Q5KTW8	DNA-directed RNA polymerase subunit OS=Giardia intestinalis OX=5741 P	17.18	3.47	6	5	5
A8B2F1	Uncharacterized protein OS=Giardia intestinalis (strain ATCC 50803 / WB	17.17	34.20	4	4	4
A8BKB6	Uncharacterized protein OS=Giardia intestinalis (strain ATCC 50803 / WB	17.16	8.71	3	5	5
A8B7Q7	ATP-dependent RNA helicase OS=Giardia intestinalis (strain ATCC 50803 /	17.16	11.78	5	3	3
A8BCC2	ADP-ribosylation factor OS=Giardia intestinalis (strain ATCC 50803 / WB c	17.07	33.69	5	4	4
D3KI24	Uncharacterized protein OS=Giardia intestinalis (strain ATCC 50803 / WB	17.07	21.08	3	5	5
A8BP01	Uncharacterized protein OS=Giardia intestinalis (strain ATCC 50803 / WB	16.99	4.15	5	3	3
A8BWJ2	RNA helicase OS=Giardia intestinalis (strain ATCC 50803 / WB clone C6) C	16.97	3.10	5	5	5
A8BSS7	Ankyrin repeat protein 1 OS=Giardia intestinalis (strain ATCC 50803 / WB	16.97	8.84	21	5	6
A8BP44	Uncharacterized protein OS=Giardia intestinalis (strain ATCC 50803 / WB	16.97	17.01	4	3	3
A8BKV3	Uncharacterized protein OS=Giardia intestinalis (strain ATCC 50803 / WB	16.94	11.03	4	6	6
A8BDW2	Glycine-, glutamate-, thienylcyclohexylpiperidine-binding protein OS=Giar	16.93	5.05	5	5	5
A8BD46	Uncharacterized protein OS=Giardia intestinalis (strain ATCC 50803 / WB	16.92	17.12	5	5	5
A8BCS0	Prefoldin subunit 3 OS=Giardia intestinalis (strain ATCC 50803 / WB clone	16.89	38.54	3	6	6
A8BJK2	Uncharacterized protein OS=Giardia intestinalis (strain ATCC 50803 / WB	16.84	8.29	5	4	4

A8BJ58	Putative U3 small nucleolar ribonucleoprotein IMP4 OS=Giardia intestinalis	16.84	15.85	5	5	5
A8BPD3	20S proteasome alpha subunit 1 OS=Giardia intestinalis (strain ATCC 508	16.79	24.70	5	5	5
A8B659	Radial spokehead-like protein OS=Giardia intestinalis (strain ATCC 50803 /	16.78	7.86	4	5	5
A0A644F3U0	Acetyl-CoA synthetase OS=Giardia intestinalis (strain ATCC 50803 / WB cl	16.71	10.21	6	6	6
D3KHG2	Uncharacterized protein OS=Giardia intestinalis (strain ATCC 50803 / WB	16.69	7.86	5	5	5
A0A644F697	5'-3' exoribonuclease 2 OS=Giardia intestinalis (strain ATCC 50803 / WB c	16.67	2.91	6	5	5
A8B4B5	Putative U3 small nucleolar RNA interacting protein OS=Giardia intestinalis	16.67	12.69	5	6	6
A8BV59	Protein geranylgeranyltransferase type II OS=Giardia intestinalis (strain A	16.61	9.47	3	7	7
V6TWE1	Cilia- and flagella-associated protein 206 OS=Giardia intestinalis OX=5741	16.60	6.26	1	1	4
A0A644EYW8	Long chain fatty acid CoA ligase 5 OS=Giardia intestinalis (strain ATCC 50	16.56	10.09	5	5	5
A8BI87	Ankyrin repeat protein 1 OS=Giardia intestinalis (strain ATCC 50803 / WB	16.50	13.74	4	5	5
A8BZI5	Uncharacterized protein OS=Giardia intestinalis (strain ATCC 50803 / WB	16.36	4.12	2	6	6
A0A644F389	Coiled-coil protein OS=Giardia intestinalis (strain ATCC 50803 / WB clone	16.34	8.23	5	4	4
A8BX22	Stress-induced-phosphoprotein 1 OS=Giardia intestinalis (strain ATCC 508	16.34	11.75	4	4	4
A8BIK2	Uncharacterized protein OS=Giardia intestinalis (strain ATCC 50803 / WB	16.31	2.94	5	5	5
A0A644F6Z2	Uncharacterized protein OS=Giardia intestinalis (strain ATCC 50803 / WB	16.31	5.81	3	5	5
A8B614	Kinase, NEK OS=Giardia intestinalis (strain ATCC 50803 / WB clone C6) O	16.31	11.37	5	4	4
A8BAV7	Uncharacterized protein OS=Giardia intestinalis (strain ATCC 50803 / WB	16.27	1.07	2	3	4
A8BJE5	Proteasome subunit beta OS=Giardia intestinalis (strain ATCC 50803 / WB	16.26	21.53	4	3	3
A8BSY0	Flagella associated protein OS=Giardia intestinalis (strain ATCC 50803 / W	16.24	10.97	6	5	5
D3KFY9	Uncharacterized protein OS=Giardia intestinalis (strain ATCC 50803 / WB	16.21	29.86	4	4	4
O76458	Ornithine transcarbamylase OS=Giardia intestinalis OX=5741 GN=OTC PE	16.21	12.84	5	4	4
A8BBC1	ATP synthase subunit E family protein OS=Giardia intestinalis (strain ATCC	16.20	23.19	5	3	3
A0A644EYY1	NADH oxidase OS=Giardia intestinalis (strain ATCC 50803 / WB clone C6)	16.16	11.14	7	4	4
A8BBR4	Uncharacterized protein OS=Giardia intestinalis (strain ATCC 50803 / WB	16.15	3.52	2	5	5
A8BA85	Kinase, NAK OS=Giardia intestinalis (strain ATCC 50803 / WB clone C6) O	16.10	8.15	3	4	4
V6TPY1	Uncharacterized protein OS=Giardia intestinalis OX=5741 GN=DHA2_856	16.09	27.81	2	5	5
A8BRD0	Uncharacterized protein OS=Giardia intestinalis (strain ATCC 50803 / WB	16.06	4.94	3	4	4
V6TEL0	Ribosome biogenesis regulatory protein OS=Giardia intestinalis OX=5741	16.05	29.55	3	2	2
V6TK10	Non-specific serine/threonine protein kinase OS=Giardia intestinalis OX=5	15.99	10.35	3	5	5
A8B861	Ankyrin repeat protein 1 OS=Giardia intestinalis (strain ATCC 50803 / WB	15.92	8.34	2	6	6
A8BN59	Uncharacterized protein OS=Giardia intestinalis (strain ATCC 50803 / WB	15.85	11.73	5	5	5
A8BSN8	Kinase, NEK OS=Giardia intestinalis (strain ATCC 50803 / WB clone C6) O	15.76	18.62	3	5	5
D3KGH1	Uncharacterized protein OS=Giardia intestinalis (strain ATCC 50803 / WB	15.71	19.08	3	4	4
A8BA58	Ankyrin repeat protein 1 OS=Giardia intestinalis (strain ATCC 50803 / WB	15.71	5.17	4	5	5
A8BR17	D123 family protein OS=Giardia intestinalis (strain ATCC 50803 / WB clon	15.69	23.76	3	4	4
A8B7X7	Putative OTU-like cysteine protease OS=Giardia intestinalis (strain ATCC 5	15.69	18.60	4	5	5
A8B2L6	Eukaryotic peptide chain release factor subunit 1 OS=Giardia intestinalis (	15.67	13.35	6	5	5
Q5XTS3	Histone deacetylase OS=Giardia intestinalis OX=5741 PE=3 SV=1 - [Q5XT	15.67	10.28	4	3	3

A0A644F8A0	Ubiquitin family protein OS=Giardia intestinalis (strain ATCC 50803 / WB c	15.61	9.73	4	4	4
A8BIT7	C3HC4-type zinc-finger domain-containing protein OS=Giardia intestinalis	15.51	3.73	3	4	4
Q962W2	DEAD-box RNA helicase OS=Giardia intestinalis OX=5741 PE=4 SV=1 - [Q	15.49	12.45	4	4	4
A8BX77	ATP-dependent RNA helicase OS=Giardia intestinalis (strain ATCC 50803 /	15.49	9.45	2	5	5
A8BI36	Transcription factor TFIIS OS=Giardia intestinalis (strain ATCC 50803 / WI	15.45	20.81	4	5	5
A8B8N3	Tryptophanyl-tRNA synthetase OS=Giardia intestinalis (strain ATCC 50803	15.44	13.75	4	3	6
A8B7R2	SAS-6_N domain-containing protein OS=Giardia intestinalis (strain ATCC 5	15.43	30.16	5	4	4
A8BA76	Nar1 OS=Giardia intestinalis (strain ATCC 50803 / WB clone C6) OX=1849	15.43	11.88	3	5	5
A0A644F2L6	tRNA guanosine-2'-O-methyltransferase OS=Giardia intestinalis (strain ATC	15.43	4.08	5	5	5
A8B386	Kinase, NEK OS=Giardia intestinalis (strain ATCC 50803 / WB clone C6) O:	15.37	6.49	5	4	4
A8B1T9	Uncharacterized protein OS=Giardia intestinalis (strain ATCC 50803 / WB c	15.35	8.78	5	4	4
A8B3D2	Putative RNA binding OS=Giardia intestinalis (strain ATCC 50803 / WB clo	15.35	22.87	4	3	3
A8BAF3	Peptidylprolyl isomerase OS=Giardia intestinalis (strain ATCC 50803 / WB	15.32	16.86	4	4	4
A8BRV7	Kinase, NEK OS=Giardia intestinalis (strain ATCC 50803 / WB clone C6) O:	15.26	13.47	4	4	4
D3KG97	Uncharacterized protein OS=Giardia intestinalis (strain ATCC 50803 / WB c	15.20	5.12	3	3	3
A8B594	DUF4821 domain-containing protein OS=Giardia intestinalis (strain ATCC 5	15.19	3.40	5	4	4
A8B6M6	Uncharacterized protein OS=Giardia intestinalis (strain ATCC 50803 / WB c	15.11	11.40	5	5	5
A8BGV3	Clusterin associated protein 1, putative OS=Giardia intestinalis (strain ATC	15.09	11.83	4	4	4
A8B8J3	Kinase, CMGC CDK OS=Giardia intestinalis (strain ATCC 50803 / WB clone	15.08	16.47	4	6	6
A8B5Q9	Ankyrin repeat protein 1 OS=Giardia intestinalis (strain ATCC 50803 / WB	15.07	6.22	5	4	4
A8BEL5	Lysine--tRNA ligase OS=Giardia intestinalis (strain ATCC 50803 / WB clone	15.07	11.98	5	6	6
A8BHC2	Uncharacterized protein OS=Giardia intestinalis (strain ATCC 50803 / WB c	15.04	9.29	3	4	4
A8BGU6	Uncharacterized protein OS=Giardia intestinalis (strain ATCC 50803 / WB c	14.86	10.51	4	4	4
A8B9H1	Ankyrin repeat protein 3 OS=Giardia intestinalis (strain ATCC 50803 / WB	14.85	8.37	5	5	5
A0A644EZN0	Elongator complex protein 3 OS=Giardia intestinalis (strain ATCC 50803 /	14.73	13.13	7	5	5
A8BC94	Uncharacterized protein OS=Giardia intestinalis (strain ATCC 50803 / WB c	14.72	4.56	3	3	3
A8BZ45	Dynein binding protein, putative OS=Giardia intestinalis (strain ATCC 5080	14.67	8.32	3	5	5
A0A644F416	Non ATPase subunit MPR1 of 26S proteasome OS=Giardia intestinalis (stra	14.65	14.91	6	3	3
A0A644F350	Uncharacterized protein OS=Giardia intestinalis (strain ATCC 50803 / WB c	14.65	6.08	5	4	4
A0A644F3Y1	Amylomaltase OS=Giardia intestinalis (strain ATCC 50803 / WB clone C6)	14.61	5.84	4	6	6
A8BY40	Ankyrin repeat protein 1 OS=Giardia intestinalis (strain ATCC 50803 / WB	14.57	10.68	4	5	5
Q9U017	Putative glucokinase (Fragment) OS=Giardia intestinalis OX=5741 GN=gk	14.51	13.99	2	3	3
E1F5Y1	Proteasome endopeptidase complex OS=Giardia intestinalis (strain P15) O	14.46	31.76	1	1	6
A8BSF1	Proteasome subunit alpha type OS=Giardia intestinalis (strain ATCC 50803	14.43	32.24	5	4	4
A8BT82	Uncharacterized protein OS=Giardia intestinalis (strain ATCC 50803 / WB c	14.35	2.64	3	5	5
E1F0E7	Inorganic polyphosphate/ATP-NAD kinase, putative OS=Giardia intestinalis	14.27	10.87	3	4	5
A8B4R7	Kinase, NEK OS=Giardia intestinalis (strain ATCC 50803 / WB clone C6) O:	14.25	6.99	4	4	4
A0A644F6S4	Uncharacterized protein OS=Giardia intestinalis (strain ATCC 50803 / WB c	14.19	35.20	4	5	5
A8BFI3	Uncharacterized protein OS=Giardia intestinalis (strain ATCC 50803 / WB c	14.16	15.19	3	5	5

V6TJB9	Uncharacterized protein OS=Giardia intestinalis OX=5741 GN=DHA2_5543	14.16	3.85	5	4	4
A8B513	Uncharacterized protein OS=Giardia intestinalis (strain ATCC 50803 / WB	14.07	13.69	3	3	3
A8B247	Ankyrin repeat protein 1 OS=Giardia intestinalis (strain ATCC 50803 / WB	13.98	9.58	4	2	3
A8B7S5	DNA replication licensing factor MCM7 OS=Giardia intestinalis (strain ATC	13.94	6.31	4	4	4
Q24989	Ubiquitin (Fragment) OS=Giardia intestinalis OX=5741 PE=4 SV=1 - [Q24	13.89	32.89	3	2	2
A8BRX7	Alpha-snap OS=Giardia intestinalis (strain ATCC 50803 / WB clone C6) OX	13.86	17.47	3	3	3
A8BUZ0	WD40 repeat protein OS=Giardia intestinalis (strain ATCC 50803 / WB clo	13.85	9.89	4	4	4
A8BSR9	Putative Transcriptional regulator ATRX OS=Giardia intestinalis (strain AT	13.82	4.46	2	4	4
Q969C9	Kinesin-like protein (Fragment) OS=Giardia intestinalis OX=5741 GN=GlK	13.74	5.65	6	2	3
A8BBV4	RNA recognition motif family protein OS=Giardia intestinalis (strain ATCC	13.71	22.01	4	3	3
D3KGM7	Uncharacterized protein OS=Giardia intestinalis (strain ATCC 50803 / WB	13.71	3.58	5	7	7
A0A644EY39	Uncharacterized protein OS=Giardia intestinalis (strain ATCC 50803 / WB	13.70	1.97	1	2	2
A8BC06	Kinesin-related protein OS=Giardia intestinalis (strain ATCC 50803 / WB c	13.65	8.76	5	4	4
A8BB13	CPSF A subunit-containing protein OS=Giardia intestinalis (strain ATCC 50	13.64	3.40	4	4	5
A8BEN7	Ankyrin repeat protein 1 OS=Giardia intestinalis (strain ATCC 50803 / WB	13.64	8.19	2	2	2
A0A644F4I4	Acid phosphatase surE OS=Giardia intestinalis (strain ATCC 50803 / WB c	13.60	14.32	4	4	4
Q8ITF3	Putative purine nucleoside phosphorylase OS=Giardia intestinalis OX=574	13.58	18.39	5	4	4
A8B693	Uncharacterized protein OS=Giardia intestinalis (strain ATCC 50803 / WB	13.58	30.77	2	5	5
A0A644F6Q9	Uncharacterized protein OS=Giardia intestinalis (strain ATCC 50803 / WB	13.55	29.52	2	3	3
A8BBJ1	FAD-dependent glycerol-3-phosphate dehydrogenase OS=Giardia intestina	13.54	5.22	3	4	4
A8B3I6	CAF1 family ribonuclease OS=Giardia intestinalis (strain ATCC 50803 / WB	13.50	7.97	4	3	4
A0A644F9S2	Ankyrin repeat protein 1 OS=Giardia intestinalis (strain ATCC 50803 / WB	13.49	17.94	6	3	3
C6LSD2	Alpha-6 giardin OS=Giardia intestinalis (strain ATCC 50581 / GS clone H7)	13.37	16.16	3	3	3
A8BE44	CLASP N terminal domain-containing protein OS=Giardia intestinalis (strai	13.29	4.93	3	2	3
A8BB95	Basal body protein OS=Giardia intestinalis (strain ATCC 50803 / WB clone	13.28	8.60	2	3	3
A8BS74	Ubiquitin-conjugating enzyme E2 (E3-independent) OS=Giardia intestinalis	13.25	29.08	4	4	4
A8BHW4	Uncharacterized protein OS=Giardia intestinalis (strain ATCC 50803 / WB	13.20	0.82	1	4	4
A8B9G8	Ankyrin repeat protein OS=Giardia intestinalis (strain ATCC 50803 / WB c	13.19	3.60	2	4	4
A8BR81	Nop14-like family protein OS=Giardia intestinalis (strain ATCC 50803 / WB	13.19	6.75	6	4	4
A0A644F186	Fcf2 pre-rRNA processing protein OS=Giardia intestinalis (strain ATCC 508	13.19	21.89	6	4	4
A8BQQ3	Ankyrin repeat protein 1 OS=Giardia intestinalis (strain ATCC 50803 / WB	13.15	7.19	4	4	4
V6TF93	GTP-binding nuclear protein OS=Giardia intestinalis OX=5741 GN=DHA2_	13.11	24.78	2	3	3
A8BPS7	Cystatin OS=Giardia intestinalis (strain ATCC 50803 / WB clone C6) OX=1	13.06	31.58	2	2	2
A8BKZ4	R-SNARE 3 OS=Giardia intestinalis (strain ATCC 50803 / WB clone C6) OX	13.00	21.76	5	4	4
A8BRC3	Replication factor C, subunit 4 OS=Giardia intestinalis (strain ATCC 50803	12.94	15.22	5	4	4
A8B5J6	Signal recognition particle receptor OS=Giardia intestinalis (strain ATCC 5	12.92	9.11	4	3	3
D3KH71	Uncharacterized protein OS=Giardia intestinalis (strain ATCC 50803 / WB	12.92	8.87	5	3	3
A8B5K6	Ankyrin repeat protein 1 OS=Giardia intestinalis (strain ATCC 50803 / WB	12.92	6.05	5	3	3
A0A644F512	Uncharacterized protein OS=Giardia intestinalis (strain ATCC 50803 / WB	12.90	23.91	5	4	4

A8B970	Kinase, NEK OS=Giardia intestinalis (strain ATCC 50803 / WB clone C6) O[illegible]	12.89	16.84	3	3	3
A8BLE7	Uncharacterized protein OS=Giardia intestinalis (strain ATCC 50803 / WB [illegible]	12.80	7.20	5	4	4
E1EWH7	Uncharacterized protein OS=Giardia intestinalis (strain P15) OX=658858 [illegible]	12.80	12.84	4	3	3
A8BKV2	Uncharacterized protein OS=Giardia intestinalis (strain ATCC 50803 / WB [illegible]	12.79	5.99	3	3	3
A8B6P4	Kinase, NEK OS=Giardia intestinalis (strain ATCC 50803 / WB clone C6) O[illegible]	12.76	4.97	6	6	6
E1F0P4	AP complex subunit sigma OS=Giardia intestinalis (strain P15) OX=65885[illegible]	12.70	26.24	4	4	4
V6TWH0	GTP-binding protein OS=Giardia intestinalis OX=5741 GN=GSB_17149 PE	12.67	9.68	1	1	5
Q969C4	Kinesin-like protein (Fragment) OS=Giardia intestinalis OX=5741 GN=GIK[illegible]	12.64	6.04	5	3	4
A8B978	PCI domain-containing protein OS=Giardia intestinalis (strain ATCC 50803	12.64	13.05	2	3	3
D3KH30	Uncharacterized protein OS=Giardia intestinalis (strain ATCC 50803 / WB [illegible]	12.63	5.73	2	1	1
A8BDY5	ZipA OS=Giardia intestinalis (strain ATCC 50803 / WB clone C6) OX=1849	12.63	6.37	5	3	3
A8BK58	Glucose regulated protein 94 / Heat shock protein 90 OS=Giardia intestin[illegible]	12.63	6.50	5	4	4
A0A644F2T9	Ankyrin repeat protein 1 OS=Giardia intestinalis (strain ATCC 50803 / WB	12.57	9.87	3	5	5
A0A644F2Y8	Uncharacterized protein OS=Giardia intestinalis (strain ATCC 50803 / WB [illegible]	12.54	4.86	6	4	4
E1F3E5	40S ribosomal protein S26 OS=Giardia intestinalis (strain P15) OX=65885[illegible]	12.54	31.19	3	2	2
E1F517	UMP pyrophosphorylase OS=Giardia intestinalis (strain P15) OX=658858 [illegible]	12.51	23.22	4	3	3
A8BLM5	Ankyrin repeat protein 1 OS=Giardia intestinalis (strain ATCC 50803 / WB	12.44	12.41	5	2	2
A8BSE5	Uncharacterized protein OS=Giardia intestinalis (strain ATCC 50803 / WB [illegible]	12.43	2.86	4	4	4
E1F3P7	Protein F10G7.1 OS=Giardia intestinalis (strain P15) OX=658858 GN=GLP	12.42	7.40	5	4	4
C6LUB4	MIP-T3 domain-containing protein OS=Giardia intestinalis (strain ATCC 50[illegible]	12.37	5.23	5	2	2
V6TFE5	Dynein light chain OS=Giardia intestinalis OX=5741 GN=DHA2_4236 PE=[illegible]	12.36	28.97	2	3	3
A8BVS3	Uncharacterized protein OS=Giardia intestinalis (strain ATCC 50803 / WB [illegible]	12.31	19.26	2	3	3
A8BQV2	La ribonucleoprotein, putative OS=Giardia intestinalis (strain ATCC 50803	12.30	13.51	2	3	3
A8BH46	Translation machinery-associated protein 16 OS=Giardia intestinalis (strai[illegible]	12.29	28.24	3	5	5
V6THV2	Uncharacterized protein OS=Giardia intestinalis OX=5741 GN=DHA2_219[illegible]	12.29	23.53	3	3	3
A0A644F3U8	Uncharacterized protein OS=Giardia intestinalis (strain ATCC 50803 / WB [illegible]	12.27	8.47	4	3	3
A0A3G9K0A7	Beta giardin (Fragment) OS=Giardia intestinalis OX=5741 PE=3 SV=1 - [A[illegible]	12.27	19.56	170	1	4
A0A644F960	GTP-binding protein OS=Giardia intestinalis (strain ATCC 50803 / WB clon[illegible]	12.24	7.71	4	1	5
A8BHE5	GYF domain-containing protein OS=Giardia intestinalis (strain ATCC 5080[illegible]	12.16	13.39	3	3	3
A8BZP5	Dynein regulatory complex protein 10 OS=Giardia intestinalis (strain ATC[illegible]	12.16	12.07	4	2	2
A8BUF3	Actin related protein OS=Giardia intestinalis (strain ATCC 50803 / WB clon[illegible]	12.16	4.55	4	4	4
A8BSI3	Serine/threonine protein phosphatase 7 OS=Giardia intestinalis (strain AT[illegible]	12.16	11.72	4	4	4
V6TEN8	Dynein light chain OS=Giardia intestinalis OX=5741 GN=DHA2_13575 PE=[illegible]	12.11	28.97	2	3	3
Q8MUU5	RNA polymerase II subunit Rpb10 OS=Giardia intestinalis OX=5741 GN=r[illegible]	12.10	39.29	5	3	3
A8BBJ5	Replication factor C, subunit 5 OS=Giardia intestinalis (strain ATCC 50803	12.07	11.80	5	3	3
D3KGQ7	Uncharacterized protein OS=Giardia intestinalis (strain ATCC 50803 / WB [illegible]	12.06	5.71	2	4	4
A8BBV3	Uncharacterized protein OS=Giardia intestinalis (strain ATCC 50803 / WB [illegible]	12.06	3.92	3	3	3
A8BU72	Protein DPCD OS=Giardia intestinalis (strain ATCC 50803 / WB clone C6) [illegible]	12.04	25.91	4	3	3
A8B6C1	DNA mismatch repair protein OS=Giardia intestinalis (strain ATCC 50803 /	12.04	4.61	6	4	4

A8B390	Uncharacterized protein OS=Giardia intestinalis (strain ATCC 50803 / WB (	12.03	3.43	5	4	4
A0A644F2C9	Vacuolar ATP synthase subunit G OS=Giardia intestinalis (strain ATCC 508	12.03	25.47	5	2	2
C6M0E4	Gamma giardin OS=Giardia intestinalis (strain ATCC 50581 / GS clone H7)	11.98	14.79	2	3	3
E1EX17	Dynein light chain OS=Giardia intestinalis (strain P15) OX=658858 GN=GL	11.98	21.35	1	3	3
E1F6B0	cAMP-dependent protein kinase regulatory chain OS=Giardia intestinalis (s	11.95	10.65	5	4	4
V6U0W9	Snare-like domain protein OS=Giardia intestinalis OX=5741 GN=GSB_948	11.89	18.75	4	3	3
A8BG28	Uncharacterized protein OS=Giardia intestinalis (strain ATCC 50803 / WB (	11.86	3.63	5	4	4
A8BIA6	Uncharacterized protein OS=Giardia intestinalis (strain ATCC 50803 / WB (	11.85	9.58	3	3	3
A8B8F4	Protein transport protein SEC23 OS=Giardia intestinalis (strain ATCC 5080	11.84	5.69	4	3	3
A8B391	Transcription factor, CAP family protein OS=Giardia intestinalis (strain ATC	11.84	13.28	4	3	3
A0A1M4M1L9	Caltractin (Fragment) OS=Giardia intestinalis OX=5741 GN=calt PE=4 SV=	11.84	30.48	12	3	3
A8BMK5	Putative Rho GAP OS=Giardia intestinalis (strain ATCC 50803 / WB clone (	11.79	31.75	5	5	5
A8BE85	Ankyrin repeat protein 1 OS=Giardia intestinalis (strain ATCC 50803 / WB	11.78	4.22	3	3	3
A0A644F1Y2	Coiled-coil protein OS=Giardia intestinalis (strain ATCC 50803 / WB clone (	11.77	2.55	4	3	3
V6T812	Ubiquitin-like domain-containing protein OS=Giardia intestinalis OX=5741	11.76	14.29	3	3	3
A0A644F0Z2	Nucleotide-binding protein 35 OS=Giardia intestinalis (strain ATCC 50803 /	11.66	13.84	5	4	4
V6THB6	Proteasome endopeptidase complex OS=Giardia intestinalis OX=5741 GN=	11.65	19.18	3	2	2
V6T7C5	Phospholipid-binding Copine Family Protein OS=Giardia intestinalis OX=57	11.63	26.99	7	3	3
E1F4T5	Glycogen [starch] synthase OS=Giardia intestinalis (strain P15) OX=65885	11.60	6.51	4	3	3
A8B590	Uncharacterized protein OS=Giardia intestinalis (strain ATCC 50803 / WB (	11.58	10.91	1	3	3
A8BRK2	Leucine-rich repeat protein OS=Giardia intestinalis (strain ATCC 50803 / W	11.47	12.79	3	3	3
V6TIF6	Uncharacterized protein OS=Giardia intestinalis OX=5741 GN=DHA2_105	11.42	13.35	3	4	4
E1EZE3	Hybrid cluster protein lateral transfer candidate OS=Giardia intestinalis (st	11.41	10.39	2	1	4
A0A644F6P9	IFT complex B OS=Giardia intestinalis (strain ATCC 50803 / WB clone C6)	11.41	9.79	3	3	3
A0A644F581	Uncharacterized protein OS=Giardia intestinalis (strain ATCC 50803 / WB (	11.40	10.33	6	2	2
A8B2I5	Structural maintenance of chromosomes protein OS=Giardia intestinalis (s	11.36	3.74	2	3	3
A8BBA1	Spindle pole protein, putative OS=Giardia intestinalis (strain ATCC 50803 /	11.35	9.07	3	3	3
A8BDI6	Vacuolar protein sorting 22 OS=Giardia intestinalis (strain ATCC 50803 / V	11.35	11.72	3	3	3
D3KGC7	Uncharacterized protein OS=Giardia intestinalis (strain ATCC 50803 / WB (	11.32	6.44	4	3	3
A8BB56	Cilia- and flagella-associated protein 300 OS=Giardia intestinalis (strain AT	11.27	19.31	2	3	3
A8BE66	Radial spokehead-like protein OS=Giardia intestinalis (strain ATCC 50803 /	11.25	8.10	4	4	4
A8BA49	Uncharacterized protein OS=Giardia intestinalis (strain ATCC 50803 / WB (	11.23	3.66	4	2	2
A8BMD6	Uncharacterized protein OS=Giardia intestinalis (strain ATCC 50803 / WB (	11.16	4.71	5	4	4
A0A644F0W7	DNA helicase OS=Giardia intestinalis (strain ATCC 50803 / WB clone C6) (	11.15	5.47	3	3	3
A8B2V7	Uncharacterized protein OS=Giardia intestinalis (strain ATCC 50803 / WB (	11.15	20.63	3	4	4
A8B7A7	Vacuolar protein sorting 2 OS=Giardia intestinalis (strain ATCC 50803 / WI	11.12	20.50	4	4	4
A8BJN3	eIF-2B GDP-GTP exchange factor subunit alpha OS=Giardia intestinalis (st	11.10	13.55	5	3	3
A8BQL1	5'-AMP-activated protein kinase, beta-1 subunit OS=Giardia intestinalis (st	11.09	26.12	3	3	3
A8B498	Pyruvate kinase OS=Giardia intestinalis (strain ATCC 50803 / WB clone C6	11.08	11.39	4	4	4

A0A644F5T7	Ubiquitin binding protein, PLAA family protein OS=Giardia intestinalis (stra	11.07	5.37	3	4	4
A8BIG9	Translation initiation factor OS=Giardia intestinalis (strain ATCC 50803 / W	11.06	4.06	9	4	4
V6TD73	Polysacc_synt_4 domain-containing protein OS=Giardia intestinalis OX=57	11.06	25.60	2	3	4
A8BPH9	Uncharacterized protein OS=Giardia intestinalis (strain ATCC 50803 / WB c	11.04	0.69	2	3	3
E1F798	Uncharacterized protein OS=Giardia intestinalis (strain P15) OX=658858 G	11.01	9.11	1	2	4
E1EW13	NEK-like kinase OS=Giardia intestinalis (strain P15) OX=658858 GN=GLP1	10.99	10.41	18	1	5
A8BLY6	Uncharacterized protein OS=Giardia intestinalis (strain ATCC 50803 / WB c	10.97	9.55	2	3	3
A8B215	Uncharacterized protein OS=Giardia intestinalis (strain ATCC 50803 / WB c	10.92	4.38	3	3	3
E1F134	Rab2a OS=Giardia intestinalis (strain P15) OX=658858 GN=GLP15_1996 F	10.91	19.16	4	3	3
A8BLF9	Kinase, CAMK CAMKL OS=Giardia intestinalis (strain ATCC 50803 / WB clo	10.89	11.30	3	4	4
A8BQL3	Uncharacterized protein OS=Giardia intestinalis (strain ATCC 50803 / WB c	10.83	14.11	4	3	3
A8BFG8	Chaperone protein DnaJ subfamily B OS=Giardia intestinalis (strain ATCC !	10.75	15.50	3	3	3
A8B2L1	Kinase, NEK OS=Giardia intestinalis (strain ATCC 50803 / WB clone C6) O)	10.74	3.41	4	3	3
P49982	Adenylate kinase OS=Giardia intestinalis OX=5741 PE=3 SV=1 - [KAD_GI/	10.74	19.76	6	3	3
A8BLR8	Ubiquitin-protein ligase E3A OS=Giardia intestinalis (strain ATCC 50803 / V	10.72	3.28	5	3	3
A8BDA9	Dynein light chain roadblock OS=Giardia intestinalis (strain ATCC 50803 /	10.71	47.00	3	3	3
A0A644F3D1	Uncharacterized protein OS=Giardia intestinalis (strain ATCC 50803 / WB c	10.70	2.95	6	4	4
D3KGG6	CH-like domain-containing protein OS=Giardia intestinalis (strain ATCC 50	10.70	4.66	2	3	3
A8BMX7	ATP-dependent RNA helicase OS=Giardia intestinalis (strain ATCC 50803 /	10.66	10.06	3	5	5
A0A644EYW7	Uncharacterized protein OS=Giardia intestinalis (strain ATCC 50803 / WB c	10.62	10.87	4	4	4
V6TK60	Uncharacterized protein OS=Giardia intestinalis OX=5741 GN=DHA2_150	10.62	20.00	2	5	5
A8B953	Uncharacterized protein OS=Giardia intestinalis (strain ATCC 50803 / WB c	10.59	6.44	2	3	3
A8B8G6	Putative Serine peptidase OS=Giardia intestinalis (strain ATCC 50803 / WE	10.59	10.66	2	2	4
A8BR86	Impact-like protein OS=Giardia intestinalis (strain ATCC 50803 / WB clone	10.55	9.78	3	2	2
C6LQX3	SALP-1 OS=Giardia intestinalis (strain ATCC 50581 / GS clone H7) OX=59	10.54	17.25	3	5	5
C6LUS3	Rab2b OS=Giardia intestinalis (strain ATCC 50581 / GS clone H7) OX=598	10.52	21.59	5	4	4
A0A644F4B0	Ankyrin repeat protein 1 OS=Giardia intestinalis (strain ATCC 50803 / WB	10.51	20.00	5	3	3
A8BLV7	Ribosomal protein L37 OS=Giardia intestinalis (strain ATCC 50803 / WB cl	10.49	26.97	4	3	3
A8BLK0	Diadenosine tetraphosphate synthetase OS=Giardia intestinalis (strain ATC	10.47	6.60	3	3	3
A8BPX1	Uncharacterized protein OS=Giardia intestinalis (strain ATCC 50803 / WB c	10.44	15.71	2	2	3
Q4VPQ7	Alpha-19 giardin OS=Giardia intestinalis OX=5741 PE=4 SV=1 - [Q4VPQ7_	10.43	11.87	3	3	3
A8B470	Uncharacterized protein OS=Giardia intestinalis (strain ATCC 50803 / WB c	10.42	16.00	3	4	4
V6TCZ3	Putative TPR repeat family protein (Fragment) OS=Giardia intestinalis OX=	10.40	13.61	4	2	2
A8BHR8	Kinase, NEK OS=Giardia intestinalis (strain ATCC 50803 / WB clone C6) O)	10.38	6.05	3	3	3
D3KH67	DCUN1 domain-containing protein OS=Giardia intestinalis (strain ATCC 50	10.38	6.53	2	3	3
A8B9R3	Uncharacterized protein OS=Giardia intestinalis (strain ATCC 50803 / WB c	10.35	9.71	3	3	3
A8BFK6	Elongation factor 2 OS=Giardia intestinalis (strain ATCC 50803 / WB clone	10.33	2.25	2	2	2
A8BBJ9	Translation initiation factor OS=Giardia intestinalis (strain ATCC 50803 / W	10.28	6.13	5	4	4
E1EVQ3	Uncharacterized protein OS=Giardia intestinalis (strain P15) OX=658858 G	10.27	8.80	3	2	2

A8B5V6	CFAP91 domain-containing protein OS=Giardia intestinalis (strain ATCC 50	10.26	5.21	3	3	3
A8B4N4	Ankyrin repeat protein 1 OS=Giardia intestinalis (strain ATCC 50803 / WB	10.24	5.21	2	3	3
A8BJM1	Uncharacterized protein OS=Giardia intestinalis (strain ATCC 50803 / WB	10.24	6.48	2	3	3
Q4VPQ8	Alpha-13 giardin OS=Giardia intestinalis OX=5741 PE=4 SV=1 - [Q4VPQ8	10.22	11.88	4	3	3
A8BYF2	Hydroxymethylglutaryl-CoA synthase OS=Giardia intestinalis (strain ATCC	10.21	7.57	2	3	3
C6LRW6	Uncharacterized protein OS=Giardia intestinalis (strain ATCC 50581 / GS c	10.20	5.28	4	1	1
V6TKQ4	Uncharacterized protein OS=Giardia intestinalis OX=5741 GN=DHA2_168	10.18	21.29	2	3	3
A8BR53	Uncharacterized protein OS=Giardia intestinalis (strain ATCC 50803 / WB	10.17	4.97	5	4	4
A8BNM2	Type II inositol-1,4,5-trisphosphate 5-phosphatase OS=Giardia intestinalis	10.15	4.92	3	2	2
A8BVP0	Uncharacterized protein OS=Giardia intestinalis (strain ATCC 50803 / WB	10.13	3.04	3	3	3
A8BXB8	Ankyrin repeat protein 1 OS=Giardia intestinalis (strain ATCC 50803 / WB	10.12	12.43	4	4	4
E1F655	Uncharacterized protein OS=Giardia intestinalis (strain P15) OX=658858 G	10.11	10.16	3	3	3
A8BDB8	Uncharacterized protein OS=Giardia intestinalis (strain ATCC 50803 / WB	10.04	15.18	2	2	2
A8BIX5	Uncharacterized protein OS=Giardia intestinalis (strain ATCC 50803 / WB	10.03	3.38	2	3	3
C6LWL3	Delta giardin OS=Giardia intestinalis (strain ATCC 50581 / GS clone H7) O	10.03	11.57	5	3	3
Q86QW7	Protein argonaute OS=Giardia intestinalis OX=5741 GN=Ago PE=1 SV=2	10.02	4.61	3	3	3
A8B9P2	Bardet-Biedl syndrome 4 protein-like protein OS=Giardia intestinalis (strai	10.02	9.18	4	3	3
A0A644F7M5	Ankyrin repeat protein 1 OS=Giardia intestinalis (strain ATCC 50803 / WB	10.00	6.15	4	1	3
A8BCX9	ATP-dependent RNA helicase OS=Giardia intestinalis (strain ATCC 50803 /	9.98	5.28	4	2	2
A8B548	Uncharacterized protein OS=Giardia intestinalis (strain ATCC 50803 / WB	9.95	6.96	5	4	4
A0A644F182	Ankyrin repeat protein 1 OS=Giardia intestinalis (strain ATCC 50803 / WB	9.93	8.76	5	3	3
A0A644F6K5	G beta-like protein GBL OS=Giardia intestinalis (strain ATCC 50803 / WB c	9.93	11.39	4	4	4
A8BQA5	Kinesin-9 OS=Giardia intestinalis (strain ATCC 50803 / WB clone C6) OX=	9.88	6.78	5	4	4
A0A644FBS0	Putative Golgi/cell cycle associated protein OS=Giardia intestinalis (strain	9.85	6.81	3	2	2
A0A644EY58	Kinase, NEK OS=Giardia intestinalis (strain ATCC 50803 / WB clone C6) O	9.83	5.42	7	3	3
A0A644EZE9	Uncharacterized protein OS=Giardia intestinalis (strain ATCC 50803 / WB	9.83	4.90	3	2	2
A0A644F557	Uncharacterized protein OS=Giardia intestinalis (strain ATCC 50803 / WB	9.81	13.25	4	2	2
V6TBL3	Putative ADP-ribosylation factor OS=Giardia intestinalis OX=5741 GN=DH	9.80	21.47	2	3	3
A8BPV9	Uncharacterized protein OS=Giardia intestinalis (strain ATCC 50803 / WB	9.80	18.08	2	3	3
A0A644F5L9	3-mercaptopyruvate sulfurtransferase/Thiosulfate sulfurtransferase OS=Gi	9.75	10.08	5	3	3
A8BY54	Leucyl-tRNA synthetase OS=Giardia intestinalis (strain ATCC 50803 / WB	9.73	4.01	3	2	3
A8BAT3	Uncharacterized protein OS=Giardia intestinalis (strain ATCC 50803 / WB	9.72	4.57	2	3	3
A8BH99	WD-repeat protein OS=Giardia intestinalis (strain ATCC 50803 / WB clone	9.71	4.44	2	2	2
D3KG96	Uncharacterized protein OS=Giardia intestinalis (strain ATCC 50803 / WB	9.71	7.82	4	5	5
D3KGK9	Uncharacterized protein OS=Giardia intestinalis (strain ATCC 50803 / WB	9.70	4.27	3	3	3
A8B2R4	Uncharacterized protein OS=Giardia intestinalis (strain ATCC 50803 / WB	9.70	3.95	2	3	3
D3KGI8	GDSL-like Lipase/Acylhydrolase family protein OS=Giardia intestinalis (stra	9.67	18.75	2	2	2
C6LNQ4	Isoleucyl-tRNA synthetase OS=Giardia intestinalis (strain ATCC 50581 / G	9.67	3.69	5	3	3
A8BE03	RNA pol II accessory factor, Cdc73 family, C-terminal domain-containing p	9.65	7.81	2	4	4

A8BLT2	Uncharacterized protein OS=Giardia intestinalis (strain ATCC 50803 / WB c	9.64	5.23	2	2	2
A8BM23	Ankyrin repeat protein 1 OS=Giardia intestinalis (strain ATCC 50803 / WB	9.59	9.27	3	3	3
A8B8N1	Uncharacterized protein OS=Giardia intestinalis (strain ATCC 50803 / WB c	9.57	15.08	3	3	3
C6LU24	Uncharacterized protein OS=Giardia intestinalis (strain ATCC 50581 / GS c	9.57	10.56	5	3	3
A8BHR6	ZipA OS=Giardia intestinalis (strain ATCC 50803 / WB clone C6) OX=1849	9.57	8.39	2	3	3
A8BPC6	Uncharacterized protein OS=Giardia intestinalis (strain ATCC 50803 / WB c	9.54	2.71	2	1	1
A8BE24	Uncharacterized protein OS=Giardia intestinalis (strain ATCC 50803 / WB c	9.52	4.67	3	3	3
A8BBB7	Transitional endoplasmic reticulum ATPase OS=Giardia intestinalis (strain	9.48	8.62	2	3	3
A8BLV5	Uncharacterized protein OS=Giardia intestinalis (strain ATCC 50803 / WB c	9.45	2.40	5	3	3
A8B9T4	Sec13 OS=Giardia intestinalis (strain ATCC 50803 / WB clone C6) OX=184	9.42	14.63	3	4	4
V6TRC4	Maintenance of ploidy protein MOB1 OS=Giardia intestinalis OX=5741 GN=	9.42	15.64	3	3	3
Q4VPP3	Alpha-17 giardin OS=Giardia intestinalis OX=5741 PE=4 SV=1 - [Q4VPP3_	9.35	11.61	2	3	3
A8BRM0	Eukaryotic translation initiation factor 3 subunit 2 OS=Giardia intestinalis (	9.35	9.14	3	3	3
D3KI79	RING-type zinc-finger domain-containing protein OS=Giardia intestinalis (s	9.33	4.27	2	2	2
D3KHS5	C2 DOCK-type domain-containing protein OS=Giardia intestinalis (strain A	9.32	1.25	5	3	3
E1F720	Kinase, AGC PKA OS=Giardia intestinalis (strain P15) OX=658858 GN=GLI	9.31	10.03	5	3	3
E1F0X5	Giardia trophozoite antigen GTA-1 OS=Giardia intestinalis (strain P15) OX=	9.28	25.97	2	4	4
A8B577	DNA topoisomerase OS=Giardia intestinalis (strain ATCC 50803 / WB clone	9.26	3.91	3	3	3
D3KGD8	Uncharacterized protein OS=Giardia intestinalis (strain ATCC 50803 / WB c	9.24	1.58	3	3	3
V6TFD2	Chromosome segregation protein SMC OS=Giardia intestinalis OX=5741 G	9.24	2.31	3	3	3
D3KG53	Uncharacterized protein OS=Giardia intestinalis (strain ATCC 50803 / WB c	9.18	3.29	2	2	2
A0A644EYZ3	Uncharacterized protein OS=Giardia intestinalis (strain ATCC 50803 / WB c	9.17	18.91	5	3	3
A8BFW5	Uncharacterized protein OS=Giardia intestinalis (strain ATCC 50803 / WB c	9.14	14.20	2	3	3
A8BTG7	Cathepsin B OS=Giardia intestinalis (strain ATCC 50803 / WB clone C6) O)	9.13	11.67	14	3	3
A8B388	GTP--RNA guanylyltransferase OS=Giardia intestinalis (strain ATCC 50803	9.11	7.24	5	3	3
A8BCK2	Putative U2 small nuclear ribonucleoprotein A OS=Giardia intestinalis (stra	9.11	8.57	5	3	3
A8BGE5	Uncharacterized protein OS=Giardia intestinalis (strain ATCC 50803 / WB c	9.10	10.18	2	3	3
A8BGV6	Phosphoglycerate kinase OS=Giardia intestinalis (strain ATCC 50803 / WB	9.09	8.31	5	3	3
A8BFD8	Uncharacterized protein OS=Giardia intestinalis (strain ATCC 50803 / WB c	9.09	3.41	2	2	2
A0A644F4J8	R-SNARE 2 OS=Giardia intestinalis (strain ATCC 50803 / WB clone C6) OX	9.08	19.07	5	3	3
D3KHZ3	Uncharacterized protein OS=Giardia intestinalis (strain ATCC 50803 / WB c	9.07	3.71	4	3	3
D3KG52	Uncharacterized protein OS=Giardia intestinalis (strain ATCC 50803 / WB c	9.06	7.80	2	2	2
A8B700	Adenylate kinase OS=Giardia intestinalis (strain ATCC 50803 / WB clone C	9.06	18.42	3	3	3
A8B727	Alpha/beta hydrolase OS=Giardia intestinalis (strain ATCC 50803 / WB clo	9.03	5.25	2	3	3
E1EYS7	UBC3 OS=Giardia intestinalis (strain P15) OX=658858 GN=GLP15_3664 P	9.03	21.88	2	3	3
A8BS87	Uncharacterized protein OS=Giardia intestinalis (strain ATCC 50803 / WB c	9.02	9.84	3	2	2
A8BJK0	DNA-directed DNA polymerase OS=Giardia intestinalis (strain ATCC 50803	8.98	9.11	4	3	3
A0A644EYY9	Ankyrin repeat protein 1 OS=Giardia intestinalis (strain ATCC 50803 / WB	8.92	3.86	3	2	2
A8B4Q7	Ankyrin repeat protein 1 OS=Giardia intestinalis (strain ATCC 50803 / WB	8.90	9.17	4	2	3

A8BSN2	Serine/threonine protein phosphatase 5 OS=Giardia intestinalis (strain AT	8.89	7.51	5	2	2
V6THR8	Emp24/gp25L/p24 family/GOLD protein OS=Giardia intestinalis OX=5741	8.88	21.35	4	3	3
D3KGH9	Uncharacterized protein OS=Giardia intestinalis (strain ATCC 50803 / WB	8.87	2.92	3	3	3
A8BHX3	Uncharacterized protein OS=Giardia intestinalis (strain ATCC 50803 / WB	8.84	2.06	5	3	3
C6LQ95	Kinase, STE STE20 OS=Giardia intestinalis (strain ATCC 50581 / GS clone	8.82	11.35	2	1	3
Q5XTS7	Histone acetyltransferase Gcn5 OS=Giardia intestinalis OX=5741 PE=4 SV	8.81	8.33	4	2	2
A0A644F045	NAC domain-containing protein OS=Giardia intestinalis (strain ATCC 5080	8.81	34.35	3	3	3
A8BRW6	Uncharacterized protein OS=Giardia intestinalis (strain ATCC 50803 / WB	8.81	20.95	3	2	2
A8BSJ1	Kinase, NEK OS=Giardia intestinalis (strain ATCC 50803 / WB clone C6) O	8.80	12.00	2	2	2
D3KG01	Uncharacterized protein OS=Giardia intestinalis (strain ATCC 50803 / WB	8.78	13.48	2	2	2
A8B9I2	Uncharacterized protein OS=Giardia intestinalis (strain ATCC 50803 / WB	8.76	2.59	3	3	3
A8BFP2	Kinase, PEK OS=Giardia intestinalis (strain ATCC 50803 / WB clone C6) O	8.76	2.31	4	3	3
A8BSD2	Phosphatase subunit gene g4-1 OS=Giardia intestinalis (strain ATCC 5080	8.75	9.71	4	3	3
E1F932	C-CAP/cofactor C-like domain-containing protein OS=Giardia intestinalis (	8.74	7.62	8	3	3
A0A644F1U9	Kinase, STE STE20 OS=Giardia intestinalis (strain ATCC 50803 / WB clone	8.72	5.49	5	3	3
A8BJ86	Uncharacterized protein OS=Giardia intestinalis (strain ATCC 50803 / WB	8.72	5.21	5	4	4
E1EX52	Uncharacterized protein OS=Giardia intestinalis (strain P15) OX=658858	8.71	7.08	3	3	3
A0A644F3L2	DNA-directed RNA polymerase OS=Giardia intestinalis (strain ATCC 50803	8.71	2.67	6	3	3
A8BWP4	Uncharacterized protein OS=Giardia intestinalis (strain ATCC 50803 / WB	8.70	10.57	3	2	2
E1EVV6	Tryptophanyl-tRNA synthetase OS=Giardia intestinalis (strain P15) OX=65	8.68	10.42	3	1	4
A0A644FAU6	Ankyrin repeat protein 1 OS=Giardia intestinalis (strain ATCC 50803 / WB	8.67	1.87	5	2	2
A8B924	Heat shock factor binding protein OS=Giardia intestinalis (strain ATCC 50	8.64	50.00	2	2	2
D3KH26	Uncharacterized protein OS=Giardia intestinalis (strain ATCC 50803 / WB	8.64	23.78	3	2	2
D3KGV0	Uncharacterized protein OS=Giardia intestinalis (strain ATCC 50803 / WB	8.63	12.69	2	2	2
E1EXA3	Uncharacterized protein OS=Giardia intestinalis (strain P15) OX=658858	8.63	5.36	4	3	3
A8B3S9	Ankyrin repeat protein 1 OS=Giardia intestinalis (strain ATCC 50803 / WB	8.62	3.75	3	2	2
A8BWK0	Uncharacterized protein OS=Giardia intestinalis (strain ATCC 50803 / WB	8.57	3.90	3	2	2
E1F7D0	Dynein regulatory complex OS=Giardia intestinalis (strain P15) OX=65885	8.57	7.69	3	3	3
E1F5N8	Proteasome endopeptidase complex OS=Giardia intestinalis (strain P15) O	8.54	13.93	5	2	2
A8BWA7	Uncharacterized protein OS=Giardia intestinalis (strain ATCC 50803 / WB	8.52	4.25	2	3	3
A8BL13	Kinase, Uni1 OS=Giardia intestinalis (strain ATCC 50803 / WB clone C6) O	8.51	1.26	2	3	3
A8B8F2	GiMOMP35 OS=Giardia intestinalis (strain ATCC 50803 / WB clone C6) OX	8.50	11.46	3	3	3
A8B240	RNA 3'-terminal phosphate cyclase-like protein OS=Giardia intestinalis (str	8.50	7.37	5	3	3
A8BHP4	Uncharacterized protein OS=Giardia intestinalis (strain ATCC 50803 / WB	8.49	1.04	3	3	3
A0A644F1R5	Cleavage and polyadenylation specificity factor OS=Giardia intestinalis (str	8.47	5.17	5	3	3
A8B621	Raptor_N domain-containing protein OS=Giardia intestinalis (strain ATCC	8.47	1.39	4	3	3
A0A644F199	Prefoldin subunit OS=Giardia intestinalis (strain ATCC 50803 / WB clone C	8.47	23.77	3	2	2
A8BZ00	Uncharacterized protein OS=Giardia intestinalis (strain ATCC 50803 / WB	8.46	14.84	4	3	3
V6TLS8	Translation initiation factor OS=Giardia intestinalis OX=5741 GN=DHA2_1	8.44	22.86	3	2	2

A8BVJ3	CH-like domain-containing protein OS=Giardia intestinalis (strain ATCC 50	8.43	4.29	3	3	3
A8BQY4	Kinase, ULK OS=Giardia intestinalis (strain ATCC 50803 / WB clone C6) O	8.43	1.48	5	2	3
A8B288	Uncharacterized protein OS=Giardia intestinalis (strain ATCC 50803 / WB	8.43	2.87	3	3	3
A8BW96	DNA polymerase OS=Giardia intestinalis (strain ATCC 50803 / WB clone C	8.42	1.85	6	3	3
D3KGD0	Uncharacterized protein OS=Giardia intestinalis (strain ATCC 50803 / WB	8.42	4.32	3	3	3
V6TL48	Serine/threonine protein kinase OS=Giardia intestinalis OX=5741 GN=DH	8.40	6.20	3	3	3
A8BQG5	Developmentally regulated GTP-binding protein 1 OS=Giardia intestinalis (	8.40	10.19	4	2	2
A8BIK9	EH domain binding protein epsin 2 OS=Giardia intestinalis (strain ATCC 50	8.39	7.90	3	2	2
A8BIN3	Ankyrin repeat protein 1 OS=Giardia intestinalis (strain ATCC 50803 / WB	8.39	7.54	9	5	5
D3KHU2	Uncharacterized protein OS=Giardia intestinalis (strain ATCC 50803 / WB	8.39	4.59	2	2	2
E1F817	Serine peptidase, putative OS=Giardia intestinalis (strain P15) OX=65885	8.38	7.68	1	1	3
E1F9T7	Translation elongation factor OS=Giardia intestinalis (strain P15) OX=658	8.36	11.90	3	2	2
A8BDV4	FixW protein, putative OS=Giardia intestinalis (strain ATCC 50803 / WB cl	8.36	33.08	4	3	3
A8BQH5	Kinase, CMGC CDK OS=Giardia intestinalis (strain ATCC 50803 / WB clone	8.33	7.71	3	2	2
A8BQ24	Putative Ribonuclease OS=Giardia intestinalis (strain ATCC 50803 / WB cl	8.33	3.21	2	2	2
V6TEU3	Uncharacterized protein OS=Giardia intestinalis OX=5741 GN=DHA2_524	8.33	13.79	1	2	2
A8B1X1	Endothelin-converting enzyme 2 OS=Giardia intestinalis (strain ATCC 508	8.33	15.49	6	3	3
A8BDU0	Uncharacterized protein OS=Giardia intestinalis (strain ATCC 50803 / WB	8.32	8.01	4	2	2
A0A644F7G2	N-alpha-acetyltransferase 40 OS=Giardia intestinalis (strain ATCC 50803 /	8.27	11.62	2	2	2
A0A644F922	Proteasome endopeptidase complex OS=Giardia intestinalis (strain ATCC 5	8.24	14.55	6	2	2
A8B3J9	Uncharacterized protein OS=Giardia intestinalis (strain ATCC 50803 / WB	8.23	3.02	2	2	2
A0A644EZV9	Kinase, NEK OS=Giardia intestinalis (strain ATCC 50803 / WB clone C6) O	8.22	3.08	4	1	2
A0A644EZ07	Kinase, NEK OS=Giardia intestinalis (strain ATCC 50803 / WB clone C6) O	8.20	4.09	4	2	2
D3KGL3	Uncharacterized protein OS=Giardia intestinalis (strain ATCC 50803 / WB	8.19	3.45	2	2	2
A8B355	Kinase, NEK OS=Giardia intestinalis (strain ATCC 50803 / WB clone C6) O	8.18	5.97	4	2	2
A8B6T4	Histidyl-tRNA synthetase OS=Giardia intestinalis (strain ATCC 50803 / WB	8.17	3.33	3	3	3
V6TEU2	Dynein light chain OS=Giardia intestinalis OX=5741 GN=DHA2_17371 PE=	8.15	11.29	2	1	1
V6THJ7	Serine/threonine protein kinase OS=Giardia intestinalis OX=5741 GN=DH	8.12	7.92	3	3	3
V6TVR8	Chromosome segregation protein SMC (Fragment) OS=Giardia intestinalis	8.12	7.35	1	1	4
A0A644F4S5	Putative Eukaryotic initiation factor 4E OS=Giardia intestinalis (strain ATC	8.10	15.67	5	4	4
A8B5V9	Basal body protein OS=Giardia intestinalis (strain ATCC 50803 / WB clone	8.10	9.48	2	2	2
A8B4V3	Uncharacterized protein OS=Giardia intestinalis (strain ATCC 50803 / WB	8.07	3.62	3	2	2
A8BQ80	Methyltransferase domain-containing protein OS=Giardia intestinalis (strai	8.06	4.37	2	2	2
V6U203	Serine/threonine-protein kinase NEK OS=Giardia intestinalis OX=5741 GN	8.02	2.83	4	2	2
A8B5Z8	Putative zinc-RING and/or ribbon domain-containing protein OS=Giardia i	8.01	4.43	2	3	3
Q8IT51	KRR1 small subunit processome component OS=Giardia intestinalis OX=5	8.01	10.16	4	3	3
A8BPD4	Uncharacterized protein OS=Giardia intestinalis (strain ATCC 50803 / WB	8.00	3.30	4	2	2
A8BBU2	Kinase, NEK OS=Giardia intestinalis (strain ATCC 50803 / WB clone C6) O	7.98	5.19	3	2	2
A8BNF9	Ankyrin repeat protein 1 OS=Giardia intestinalis (strain ATCC 50803 / WB	7.98	9.04	3	2	2

A8BCE3	Kinase, NEK OS=Giardia intestinalis (strain ATCC 50803 / WB clone C6) O)	7.98	6.28	4	3	3
A8BPE1	TPR_REGION domain-containing protein OS=Giardia intestinalis (strain AT	7.97	8.22	4	2	3
A8B9W1	Ankyrin repeat protein 1 OS=Giardia intestinalis (strain ATCC 50803 / WB	7.97	7.07	12	1	1
A8BT15	Glutamine-tRNA synthetase OS=Giardia intestinalis (strain ATCC 50803 / V	7.96	4.72	4	3	3
A0A644FBG1	Uncharacterized protein OS=Giardia intestinalis (strain ATCC 50803 / WB (	7.96	2.74	5	2	2
A8BK19	Uncharacterized protein OS=Giardia intestinalis (strain ATCC 50803 / WB (	7.95	1.71	7	3	3
A0A644FAS5	Prefoldin subunit OS=Giardia intestinalis (strain ATCC 50803 / WB clone C	7.92	20.75	5	2	2
D3KGL0	Uncharacterized protein OS=Giardia intestinalis (strain ATCC 50803 / WB (	7.87	3.98	3	2	2
A0A644FAW5	Uncharacterized protein OS=Giardia intestinalis (strain ATCC 50803 / WB (	7.86	9.69	2	2	2
A8BYZ5	Uncharacterized protein OS=Giardia intestinalis (strain ATCC 50803 / WB (	7.84	2.74	5	3	3
A8B5J3	Phosphatidylinositol-4,5-bisphosphate 3-kinase catalytic subunit alpha/bet	7.84	1.39	4	2	3
A8B638	Uncharacterized protein OS=Giardia intestinalis (strain ATCC 50803 / WB (	7.82	1.69	4	4	4
A8B9M4	Kinase, NEK OS=Giardia intestinalis (strain ATCC 50803 / WB clone C6) O)	7.81	7.23	2	2	2
A8BLL1	Transglutaminase-like superfamily protein OS=Giardia intestinalis (strain A	7.81	8.87	3	2	2
A8BX98	Kinase, NEK OS=Giardia intestinalis (strain ATCC 50803 / WB clone C6) O)	7.80	7.46	2	2	2
A8B671	Uncharacterized protein OS=Giardia intestinalis (strain ATCC 50803 / WB (	7.76	17.39	2	2	2
A8BBW8	Ankyrin repeat protein 1 OS=Giardia intestinalis (strain ATCC 50803 / WB	7.74	3.53	3	2	2
A0A644F6J5	Uncharacterized protein OS=Giardia intestinalis (strain ATCC 50803 / WB (	7.73	15.14	5	2	2
D3KGT6	Uncharacterized protein OS=Giardia intestinalis (strain ATCC 50803 / WB (	7.71	5.73	5	1	2
A8BUB9	DNA polymerase II subunit 2 OS=Giardia intestinalis (strain ATCC 50803 /	7.70	6.46	2	2	2
A8B467	Uncharacterized protein OS=Giardia intestinalis (strain ATCC 50803 / WB (	7.70	3.89	2	4	4
A8BAD7	N-terminal acetyltransferase complex ARD1 subunit OS=Giardia intestinali	7.68	8.41	4	3	3
E1F2F1	Uncharacterized protein OS=Giardia intestinalis (strain P15) OX=658858 G	7.68	4.14	5	2	2
E1F0A8	Uridine kinase OS=Giardia intestinalis (strain P15) OX=658858 GN=GLP15	7.68	4.27	3	3	3
C6LU00	DNA-directed RNA polymerase RPB3 OS=Giardia intestinalis (strain ATCC 5	7.67	8.90	3	2	3
A0A644EZX6	Uncharacterized protein OS=Giardia intestinalis (strain ATCC 50803 / WB (	7.66	7.08	2	2	2
A0A644EXU7	Shwachman-Bodian-Diamond protein OS=Giardia intestinalis (strain ATCC	7.65	12.65	4	2	2
A0A644F2R2	Putative Phosphatidylinositol-4-phosphate 5-kinase OS=Giardia intestinalis	7.63	5.59	6	3	3
A0A644F4H3	Uncharacterized protein OS=Giardia intestinalis (strain ATCC 50803 / WB (	7.62	7.16	4	3	3
A8BFE8	Kinase, NEK OS=Giardia intestinalis (strain ATCC 50803 / WB clone C6) O)	7.56	1.91	2	2	2
A8BLK1	Kinase, NEK OS=Giardia intestinalis (strain ATCC 50803 / WB clone C6) O)	7.51	5.34	2	3	3
A8B3U3	Uncharacterized protein OS=Giardia intestinalis (strain ATCC 50803 / WB (	7.51	5.04	2	4	4
A8BMY1	Uncharacterized protein OS=Giardia intestinalis (strain ATCC 50803 / WB (	7.50	7.61	2	1	1
A8BB71	Ankyrin repeat protein 1 OS=Giardia intestinalis (strain ATCC 50803 / WB	7.49	5.29	4	2	2
E1F5Z6	ARL1 OS=Giardia intestinalis (strain P15) OX=658858 GN=GLP15_1895 PI	7.48	16.76	4	3	3
A8BDI3	Sas10 domain-containing protein OS=Giardia intestinalis (strain ATCC 508	7.47	8.69	2	2	2
D3KHK3	tRNA-intron lyase OS=Giardia intestinalis (strain ATCC 50803 / WB clone (	7.45	4.18	2	2	2
A0A644F383	Phasin superfamily protein OS=Giardia intestinalis (strain ATCC 50803 / W	7.45	10.61	3	2	2
V6TEB8	Serine/threonine protein kinase OS=Giardia intestinalis OX=5741 GN=DH/	7.43	5.03	3	3	3

A8BHP0	DUF667 domain-containing protein OS=Giardia intestinalis (strain ATCC 50	7.42	5.92	5	3	3
D3KH27	Uncharacterized protein OS=Giardia intestinalis (strain ATCC 50803 / WB	7.42	2.57	2	2	2
A0A644F893	VSP with INR OS=Giardia intestinalis (strain ATCC 50803 / WB clone C6) (	7.35	4.59	10	3	3
A8BJQ4	Uncharacterized protein OS=Giardia intestinalis (strain ATCC 50803 / WB	7.32	3.09	2	2	2
V6TUF0	Uncharacterized protein OS=Giardia intestinalis OX=5741 GN=GSB_13262	7.29	7.85	4	2	2
A8B3F8	Ankyrin repeat protein 2 OS=Giardia intestinalis (strain ATCC 50803 / WB	7.29	2.18	3	2	2
D3KFZ8	Uncharacterized protein OS=Giardia intestinalis (strain ATCC 50803 / WB	7.24	6.50	2	2	2
D3KHH1	Uncharacterized protein OS=Giardia intestinalis (strain ATCC 50803 / WB	7.23	13.64	3	2	2
A8B7N7	WD-repeat domain 17 isoform 1 OS=Giardia intestinalis (strain ATCC 508	7.22	1.92	2	2	2
A0A644F300	Translation initiation factor eIF-2B delta subunit OS=Giardia intestinalis (s	7.22	5.27	5	3	3
V6TAI6	Serine/threonine protein kinase OS=Giardia intestinalis OX=5741 GN=DH	7.21	4.48	1	1	1
V6TRA0	A-type flavoprotein lateral transfer candidate OS=Giardia intestinalis OX=5	7.18	10.63	1	1	2
A8B354	Ubiquitinyl hydrolase 1 OS=Giardia intestinalis (strain ATCC 50803 / WB c	7.18	3.20	3	2	2
A8BT31	Ribosomal RNA-processing protein 8 OS=Giardia intestinalis (strain ATCC	7.11	8.55	5	2	2
A0A644F7X2	Uncharacterized protein OS=Giardia intestinalis (strain ATCC 50803 / WB	7.10	5.88	4	2	2
E1F2W8	Parkin-co-regulated protein, putative OS=Giardia intestinalis (strain P15) (	7.09	13.03	3	2	2
A0A644F285	Uncharacterized protein OS=Giardia intestinalis (strain ATCC 50803 / WB	7.07	2.10	4	3	3
V6TZN3	Uncharacterized protein OS=Giardia intestinalis OX=5741 GN=GSB_15275	7.04	4.63	7	1	2
E1F2F7	Ribonucleoprotein OS=Giardia intestinalis (strain P15) OX=658858 GN=GL	7.04	15.57	3	2	2
A8BB65	Zinc finger domain protein OS=Giardia intestinalis (strain ATCC 50803 / W	7.04	21.88	4	2	2
A8BY06	Uncharacterized protein OS=Giardia intestinalis (strain ATCC 50803 / WB	7.04	5.55	4	3	3
A8B3E4	Kinase, NEK OS=Giardia intestinalis (strain ATCC 50803 / WB clone C6) O	7.03	2.11	5	2	2
E1F8E2	Uncharacterized protein OS=Giardia intestinalis (strain P15) OX=658858 (	7.01	2.91	1	1	4
E1EYU5	Adenylate kinase OS=Giardia intestinalis (strain P15) OX=658858 GN=GL	7.01	8.70	2	2	2
A8BBM5	Uncharacterized protein OS=Giardia intestinalis (strain ATCC 50803 / WB	7.01	6.12	2	2	2
A8B493	Ribosomal RNA-processing protein 43 OS=Giardia intestinalis (strain ATCC	7.01	11.78	4	2	2
A8B933	Uncharacterized protein OS=Giardia intestinalis (strain ATCC 50803 / WB	6.97	0.59	2	2	2
A8BYQ2	Serine/threonine-protein phosphatase OS=Giardia intestinalis (strain ATCC	6.97	3.59	5	1	1
A8BU21	UBA-like domain-containing protein OS=Giardia intestinalis (strain ATCC 5	6.94	6.03	2	1	1
A8BPX8	Uncharacterized protein OS=Giardia intestinalis (strain ATCC 50803 / WB	6.94	11.20	6	2	2
A8BA65	Ankyrin repeat protein 1 OS=Giardia intestinalis (strain ATCC 50803 / WB	6.92	3.73	3	2	2
A8B8J9	Chaperone protein DnaJ OS=Giardia intestinalis (strain ATCC 50803 / WB	6.92	11.08	4	2	2
A8BAH7	Uncharacterized protein OS=Giardia intestinalis (strain ATCC 50803 / WB	6.91	5.98	4	2	2
A0A644F7L5	Uncharacterized protein OS=Giardia intestinalis (strain ATCC 50803 / WB	6.90	21.67	3	3	3
A8BFQ0	Uncharacterized protein OS=Giardia intestinalis (strain ATCC 50803 / WB	6.89	1.68	4	2	2
A8B6D7	Uncharacterized protein OS=Giardia intestinalis (strain ATCC 50803 / WB	6.87	4.80	2	2	2
Q86QZ1	A-type flavoprotein OS=Giardia intestinalis OX=5741 GN=fprA PE=1 SV=1	6.86	8.45	3	2	3
A8BFX0	Kinase, NEK OS=Giardia intestinalis (strain ATCC 50803 / WB clone C6) O	6.85	5.43	2	2	2
A0A644F660	HSP70 subfamily B suppressor 1 OS=Giardia intestinalis (strain ATCC 508	6.83	4.47	5	2	2

A8B9E2	Kinase, NEK OS=Giardia intestinalis (strain ATCC 50803 / WB clone C6) O	6.81	3.35	2	2	2
A8B8X0	Chromosome segregation ATPase OS=Giardia intestinalis (strain ATCC 508	6.78	1.90	2	2	2
A8BQ84	Phosphatidylinositol-4-phosphate 5-kinase, putative OS=Giardia intestinali	6.78	11.35	4	3	3
A8BRU1	Thymidine kinase OS=Giardia intestinalis (strain ATCC 50803 / WB clone (	6.73	6.12	2	2	2
A8BU63	dTMP kinase OS=Giardia intestinalis (strain ATCC 50803 / WB clone C6) C	6.72	9.06	4	2	2
A8B6N6	WD40 repeat protein OS=Giardia intestinalis (strain ATCC 50803 / WB clor	6.69	1.49	5	2	2
A8BD51	NAD-dependent histone deacetylase Sir2 OS=Giardia intestinalis (strain AT	6.69	5.37	5	2	2
A8BIF5	Uncharacterized protein OS=Giardia intestinalis (strain ATCC 50803 / WB	6.66	6.21	4	2	2
A8BUD4	BBS1 domain-containing protein OS=Giardia intestinalis (strain ATCC 5080	6.66	4.24	3	2	2
E1EVM2	60S ribosomal protein L29 OS=Giardia intestinalis (strain P15) OX=65885	6.65	14.52	3	1	1
A8BNI9	Alpha/beta hydrolase family protein OS=Giardia intestinalis (strain ATCC 5	6.63	9.45	3	2	2
A8B810	kinase, wee OS=Giardia intestinalis (strain ATCC 50803 / WB clone C6) O	6.60	1.07	2	2	2
Q9GQ39	Cysteinyl-tRNA synthetase OS=Giardia intestinalis OX=5741 GN=cysS PE=	6.56	4.96	2	2	2
A8BJB0	Uncharacterized protein OS=Giardia intestinalis (strain ATCC 50803 / WB	6.56	15.04	4	3	3
A8BQG2	Uncharacterized protein OS=Giardia intestinalis (strain ATCC 50803 / WB	6.54	1.80	2	2	2
A8B3A6	Uncharacterized protein OS=Giardia intestinalis (strain ATCC 50803 / WB	6.53	5.17	3	2	2
A8BJ49	Uncharacterized protein OS=Giardia intestinalis (strain ATCC 50803 / WB	6.52	17.47	2	2	2
Q969D0	Kinesin-like protein (Fragment) OS=Giardia intestinalis OX=5741 GN=GlKl	6.52	4.23	5	2	2
Q4VPP6	Alpha-15 giardin OS=Giardia intestinalis OX=5741 PE=4 SV=1 - [Q4VPP6_	6.48	8.25	2	2	2
A8BY03	Coiled-coil protein OS=Giardia intestinalis (strain ATCC 50803 / WB clone	6.48	2.77	5	2	2
A0A644F542	Uncharacterized protein OS=Giardia intestinalis (strain ATCC 50803 / WB	6.46	4.33	4	2	2
A8BCL1	Nitroreductase Fd-NR2 OS=Giardia intestinalis (strain ATCC 50803 / WB cl	6.45	8.71	2	2	2
A8B2T1	Coiled-coil protein OS=Giardia intestinalis (strain ATCC 50803 / WB clone	6.44	1.74	3	2	2
D3KG04	Uncharacterized protein OS=Giardia intestinalis (strain ATCC 50803 / WB	6.44	2.86	2	2	2
A8B367	Spindle pole body component OS=Giardia intestinalis (strain ATCC 50803	6.43	2.93	4	2	2
A8BDW9	Uncharacterized protein OS=Giardia intestinalis (strain ATCC 50803 / WB	6.42	3.78	2	2	2
Q8MUU7	RNA polymerase II subunit Rpb7 OS=Giardia intestinalis OX=5741 GN=rp	6.42	17.45	6	2	2
A8BA42	Sperm-associated WD-repeat protein OS=Giardia intestinalis (strain ATCC	6.41	3.77	5	2	2
A8BGC6	Proteasome endopeptidase complex OS=Giardia intestinalis (strain ATCC 5	6.40	10.86	2	2	2
A8BGN5	Kinase, NEK OS=Giardia intestinalis (strain ATCC 50803 / WB clone C6) O	6.40	2.33	5	2	2
A8BUZ8	Putative Lecithin-cholesterol acyl transferase OS=Giardia intestinalis (strai	6.40	1.13	5	2	2
A0A482G348	Thioredoxin peroxidase OS=Giardia intestinalis OX=5741 GN=prx1 PE=4 S	6.40	12.60	5	2	3
D3KGK0	Uncharacterized protein OS=Giardia intestinalis (strain ATCC 50803 / WB	6.40	5.54	3	2	2
A8BUD0	Uncharacterized protein OS=Giardia intestinalis (strain ATCC 50803 / WB	6.36	3.80	3	4	4
A8BLX3	Epsilon-tubulin OS=Giardia intestinalis (strain ATCC 50803 / WB clone C6)	6.33	4.81	5	1	1
A8BZ35	Eukaryotic translation initiation factor 3 30 kDa subunit OS=Giardia intesti	6.32	8.43	3	2	2
D3KHW1	Uncharacterized protein OS=Giardia intestinalis (strain ATCC 50803 / WB	6.31	6.81	2	3	3
D3KH07	Uncharacterized protein OS=Giardia intestinalis (strain ATCC 50803 / WB	6.30	1.10	2	2	2
V6TL11	ER lumen protein retaining receptor OS=Giardia intestinalis OX=5741 GN=	6.29	5.12	1	1	1

V6TJI0	Putative Rho GTPase-activating protein OS=Giardia intestinalis OX=5741 (	6.28	17.79	2	2	2
A8B532	Kinase OS=Giardia intestinalis (strain ATCC 50803 / WB clone C6) OX=18	6.27	5.88	3	3	3
A8B903	Putative Rho GAP OS=Giardia intestinalis (strain ATCC 50803 / WB clone (	6.26	3.72	4	2	2
A8BW17	Uncharacterized protein OS=Giardia intestinalis (strain ATCC 50803 / WB (	6.24	2.42	2	2	2
A8BPR0	Proteasome subunit beta OS=Giardia intestinalis (strain ATCC 50803 / WB	6.24	13.04	5	2	2
V6T9Z8	Putative bromodomain motif protein OS=Giardia intestinalis OX=5741 GN=	6.23	13.62	3	3	3
A8B5W8	Uncharacterized protein OS=Giardia intestinalis (strain ATCC 50803 / WB (	6.22	7.40	3	2	2
A8BQ45	Uncharacterized protein OS=Giardia intestinalis (strain ATCC 50803 / WB (	6.20	4.28	3	2	2
D3KGF9	Uncharacterized protein OS=Giardia intestinalis (strain ATCC 50803 / WB (	6.18	2.46	2	2	2
E1F8R2	Ribosomal protein P1B OS=Giardia intestinalis (strain P15) OX=658858 GI	6.17	35.29	2	1	2
A8BW54	Kinase, NEK OS=Giardia intestinalis (strain ATCC 50803 / WB clone C6) O:	6.17	3.44	3	2	2
A8BH12	DNA helicase OS=Giardia intestinalis (strain ATCC 50803 / WB clone C6) (	6.13	3.23	4	2	2
C6LT55	Serine/threonine-protein phosphatase OS=Giardia intestinalis (strain ATC(	6.13	6.46	4	2	2
D3KGS9	Uncharacterized protein OS=Giardia intestinalis (strain ATCC 50803 / WB (	6.12	4.07	5	2	2
V6TRP0	Uncharacterized protein OS=Giardia intestinalis OX=5741 GN=DHA2_111	6.11	7.41	4	2	2
V6T942	Centromere/microtubule binding protein cbf5 (Fragment) OS=Giardia inte	6.11	10.55	4	3	3
A8B5Z0	Uncharacterized protein OS=Giardia intestinalis (strain ATCC 50803 / WB (	6.07	7.03	3	1	1
A0A482ETX1	Histone H2A OS=Giardia intestinalis OX=5741 GN=DHA2_152990 PE=3 S	6.07	17.74	5	1	1
O97451	Protein disulfide isomerase-1 OS=Giardia intestinalis OX=5741 GN=PDI-1	6.07	10.26	3	2	2
A8BSF3	Centrosomal protein POC5 OS=Giardia intestinalis (strain ATCC 50803 / W	6.06	3.27	5	1	1
A8BG73	Uncharacterized protein OS=Giardia intestinalis (strain ATCC 50803 / WB (	6.00	1.61	5	3	3
A8BGH6	Kinase, NEK OS=Giardia intestinalis (strain ATCC 50803 / WB clone C6) O:	5.98	1.87	3	2	2
A8BRU6	SOF1 protein OS=Giardia intestinalis (strain ATCC 50803 / WB clone C6) (	5.97	4.07	4	2	2
A8BKY4	Uncharacterized protein OS=Giardia intestinalis (strain ATCC 50803 / WB (	5.96	6.63	3	2	2
A8B2M2	Uncharacterized protein OS=Giardia intestinalis (strain ATCC 50803 / WB (	5.95	1.13	2	2	2
C6LQS0	Serine/threonine-protein phosphatase OS=Giardia intestinalis (strain ATC(	5.93	7.55	4	2	2
A8BD23	Translation initiation factor 2 gamma subunit OS=Giardia intestinalis (strai	5.93	5.86	4	2	2
A8B322	PX domain-containing protein OS=Giardia intestinalis (strain ATCC 50803 ,	5.93	2.15	4	2	2
A8B780	Nucleoporin complex subunit 54 OS=Giardia intestinalis (strain ATCC 508(	5.90	6.36	3	2	2
A8BZP3	Phenylalanine--tRNA ligase OS=Giardia intestinalis (strain ATCC 50803 / V	5.90	5.73	2	2	2
A8BSF6	Ribosomal RNA-processing protein 7 OS=Giardia intestinalis (strain ATCC !	5.90	9.09	4	2	2
A8B3I5	tRNA pseudouridine synthase OS=Giardia intestinalis (strain ATCC 50803 /	5.86	5.78	5	2	2
V6TGK4	Uncharacterized protein OS=Giardia intestinalis OX=5741 GN=DHA2_144	5.85	17.16	2	2	2
D3KH75	Uncharacterized protein OS=Giardia intestinalis (strain ATCC 50803 / WB (	5.82	2.33	2	2	2
A8BH45	Ankyrin repeat protein 1 OS=Giardia intestinalis (strain ATCC 50803 / WB	5.82	4.55	3	1	1
E1F5G6	Protein MAK16 homolog OS=Giardia intestinalis (strain P15) OX=658858 (	5.81	10.59	3	2	2
D3KG13	Uncharacterized protein OS=Giardia intestinalis (strain ATCC 50803 / WB (	5.75	5.58	3	3	3
D3KG41	Uncharacterized protein OS=Giardia intestinalis (strain ATCC 50803 / WB (	5.74	9.32	3	2	2
V6T716	FOP_dimer domain-containing protein OS=Giardia intestinalis OX=5741 G	5.74	23.66	3	2	2

A8BNE2	Uncharacterized protein OS=Giardia intestinalis (strain ATCC 50803 / WB	5.71	2.52	2	2	2
A8BBM0	Obg-like ATPase 1 OS=Giardia intestinalis (strain ATCC 50803 / WB clone	5.71	5.42	6	2	2
D3KHN8	Uncharacterized protein OS=Giardia intestinalis (strain ATCC 50803 / WB	5.67	3.38	1	1	1
A0A644F2V0	AAA domain-containing protein OS=Giardia intestinalis (strain ATCC 5080	5.63	10.15	3	2	2
A8BGC2	Kinase, NEK OS=Giardia intestinalis (strain ATCC 50803 / WB clone C6) O	5.63	4.75	6	3	3
A8BDU9	Uncharacterized protein OS=Giardia intestinalis (strain ATCC 50803 / WB	5.61	3.73	2	1	1
E1F923	RabB OS=Giardia intestinalis (strain P15) OX=658858 GN=GLP15_2945 P	5.61	9.29	4	1	1
A8BMM2	Uncharacterized protein OS=Giardia intestinalis (strain ATCC 50803 / WB	5.61	9.85	4	2	2
Q9NBW5	Alcohol dehydrogenase 2 OS=Giardia intestinalis OX=5741 GN=Adh2 PE=	5.60	5.64	5	2	2
A0A644F5K8	DNA replication licensing factor MCM7 OS=Giardia intestinalis (strain ATC	5.59	2.41	3	1	1
A0A644F9X7	Kinase, NEK OS=Giardia intestinalis (strain ATCC 50803 / WB clone C6) O	5.58	9.28	2	2	2
A8B6U5	Shwachman-Bodian-Diamond syndrome like protein OS=Giardia intestinali	5.58	8.37	3	2	2
D3KHP2	Uncharacterized protein OS=Giardia intestinalis (strain ATCC 50803 / WB	5.57	3.35	4	2	2
E1F7Z4	ANK_REP_REGION domain-containing protein OS=Giardia intestinalis (stra	5.56	5.66	3	2	2
A8BD15	Heat shock protein 70 OS=Giardia intestinalis (strain ATCC 50803 / WB cl	5.54	2.09	2	2	2
A0A644EYR3	Uncharacterized protein OS=Giardia intestinalis (strain ATCC 50803 / WB	5.54	1.76	6	3	3
V6TMA9	Uncharacterized protein OS=Giardia intestinalis OX=5741 GN=DHA2_151	5.53	0.59	2	1	1
A8BBA2	GPN-loop GTPase 3 OS=Giardia intestinalis (strain ATCC 50803 / WB clone	5.52	7.49	1	1	1
V6U262	Protein Translation Initiation Factor 1A (IF-1A) OS=Giardia intestinalis OX	5.52	8.18	4	1	1
B0F2C7	Histone H2B (Fragment) OS=Giardia intestinalis OX=5741 GN=H2B PE=3	5.51	21.43	12	2	2
A8BGD8	Uncharacterized protein OS=Giardia intestinalis (strain ATCC 50803 / WB	5.49	1.99	1	1	1
A8BTP5	Ankyrin repeat protein 2 OS=Giardia intestinalis (strain ATCC 50803 / WB	5.48	4.28	5	2	2
A8BGJ7	Uncharacterized protein OS=Giardia intestinalis (strain ATCC 50803 / WB	5.47	8.43	4	2	2
A8BUB7	Uncharacterized protein OS=Giardia intestinalis (strain ATCC 50803 / WB	5.47	8.05	3	2	2
E1EW51	Uncharacterized protein OS=Giardia intestinalis (strain P15) OX=658858 G	5.46	4.58	2	2	2
A8BU38	Putative RNA binding protein OS=Giardia intestinalis (strain ATCC 50803 /	5.45	3.80	3	3	3
A8BHC4	Uncharacterized protein OS=Giardia intestinalis (strain ATCC 50803 / WB	5.42	5.61	3	2	2
V6TCH3	Ribonucleoprotein OS=Giardia intestinalis OX=5741 GN=DHA2_11287 PE=	5.41	11.48	1	1	1
A0A644F4M0	Uncharacterized protein OS=Giardia intestinalis (strain ATCC 50803 / WB	5.41	1.75	3	1	1
A8B796	Uncharacterized protein OS=Giardia intestinalis (strain ATCC 50803 / WB	5.38	20.31	4	2	2
A8BQU8	Ribosomal protein L44 OS=Giardia intestinalis (strain ATCC 50803 / WB cl	5.35	17.92	4	2	2
A0A644F5U5	Activator of Hsp90 ATPase OS=Giardia intestinalis (strain ATCC 50803 / W	5.34	18.37	5	3	3
A8BKE5	Ubiquitin fusion degradation protein 1 OS=Giardia intestinalis (strain ATC	5.33	8.95	4	2	2
A8B3J0	Uncharacterized protein OS=Giardia intestinalis (strain ATCC 50803 / WB	5.32	0.33	2	2	2
A8BXR8	Tubulin tyrosine ligase OS=Giardia intestinalis (strain ATCC 50803 / WB cl	5.32	2.98	4	2	2
A8BPW1	Hydrolase, putative OS=Giardia intestinalis (strain ATCC 50803 / WB clone	5.32	9.65	2	2	2
A8BTW5	Uncharacterized protein OS=Giardia intestinalis (strain ATCC 50803 / WB	5.31	7.10	3	2	2
A8BC16	ATPase of the PP-loop superfamily implicated in cell cycle control, putative	5.30	4.84	1	1	1
A8BL35	Uncharacterized protein OS=Giardia intestinalis (strain ATCC 50803 / WB	5.29	3.84	3	2	2

C6LSU7	Protein 21.1 OS=Giardia intestinalis (strain ATCC 50581 / GS clone H7) OX	5.26	3.25	5	2	2
A8BDU7	Ankyrin repeat protein 1 OS=Giardia intestinalis (strain ATCC 50803 / WB	5.26	1.59	2	2	2
A8BI79	Uncharacterized protein OS=Giardia intestinalis (strain ATCC 50803 / WB	5.26	1.24	2	1	1
V6TH97	Uncharacterized protein OS=Giardia intestinalis OX=5741 GN=DHA2_150	5.24	9.06	2	2	2
A0A644EY49	Uncharacterized protein OS=Giardia intestinalis (strain ATCC 50803 / WB	5.24	4.30	4	2	2
A8B8X6	Uncharacterized protein OS=Giardia intestinalis (strain ATCC 50803 / WB	5.22	3.98	2	1	1
A8B3R8	Uncharacterized protein OS=Giardia intestinalis (strain ATCC 50803 / WB	5.21	1.47	2	2	2
D3KGQ5	Uncharacterized protein OS=Giardia intestinalis (strain ATCC 50803 / WB	5.20	5.65	4	2	2
A8B2V6	Ankyrin repeat protein 1 OS=Giardia intestinalis (strain ATCC 50803 / WB	5.20	2.12	2	2	2
A8B9T2	Kinase, NEK OS=Giardia intestinalis (strain ATCC 50803 / WB clone C6) O	5.18	3.59	3	2	2
D3KGQ4	Uncharacterized protein OS=Giardia intestinalis (strain ATCC 50803 / WB	5.18	1.68	3	2	2
A8B4P6	Alpha/beta hydrolase family protein OS=Giardia intestinalis (strain ATCC 5	5.18	11.64	2	1	1
A8BM21	UAA transporter family protein OS=Giardia intestinalis (strain ATCC 50803	5.15	5.94	2	1	1
A8B827	DNA-directed RNA polymerase subunit beta OS=Giardia intestinalis (strain	5.15	2.59	3	3	3
E1EXC6	WD-40 repeat protein family OS=Giardia intestinalis (strain P15) OX=6588	5.14	6.14	2	1	1
A8BKZ3	ZZ-type domain-containing protein OS=Giardia intestinalis (strain ATCC 50	5.12	1.23	3	2	2
A8BD81	Tctex-1 family protein OS=Giardia intestinalis (strain ATCC 50803 / WB cl	5.12	15.20	4	2	2
A8B980	Uncharacterized protein OS=Giardia intestinalis (strain ATCC 50803 / WB	5.11	8.33	2	1	1
A8BGG9	FAD synthetase OS=Giardia intestinalis (strain ATCC 50803 / WB clone C6	5.08	3.70	4	2	2
D3KFZ5	Uncharacterized protein OS=Giardia intestinalis (strain ATCC 50803 / WB	5.05	5.54	2	1	1
D3KHJ8	Uncharacterized protein OS=Giardia intestinalis (strain ATCC 50803 / WB	5.05	2.25	4	2	2
E1EY57	Uncharacterized protein OS=Giardia intestinalis (strain P15) OX=658858 G	5.04	0.59	3	1	2
A0A644F560	tRNA(Phe) (4-demethylwyosine(37)-C(7)) aminocarboxypropyltransferase	5.03	5.58	6	2	2
A0A644F9B4	Uncharacterized protein OS=Giardia intestinalis (strain ATCC 50803 / WB	5.01	3.69	3	2	2
A8BWS9	Kinase, NEK OS=Giardia intestinalis (strain ATCC 50803 / WB clone C6) O	5.01	2.69	3	2	2
Q8MUU9	RNA polymerase II subunit Rpb5b OS=Giardia intestinalis OX=5741 GN=r	5.00	8.46	2	1	1
Q8T694	RabF (Fragment) OS=Giardia intestinalis OX=5741 GN=RabF PE=4 SV=2	5.00	7.55	2	1	1
A8BAK7	Uncharacterized protein OS=Giardia intestinalis (strain ATCC 50803 / WB	4.98	11.44	2	1	1
A8BA69	tRNA/rRNA cytosine-C5-methylase OS=Giardia intestinalis (strain ATCC 50	4.98	4.80	5	2	2
E1F7F5	Pyrophosphate--fructose 6-phosphate 1-phosphotransferase OS=Giardia i	4.98	4.04	4	1	1
E1F1M5	Kinase (Fragment) OS=Giardia intestinalis (strain P15) OX=658858 GN=G	4.97	1.13	3	1	1
A8BXI7	Topoisomerase I-related protein OS=Giardia intestinalis (strain ATCC 508	4.95	3.32	3	2	2
A8BD86	Phosphorylated CTD interacting factor PCIF1 OS=Giardia intestinalis (strai	4.95	3.34	4	2	2
V6U2C2	Protein kinase, CMGC GSK OS=Giardia intestinalis OX=5741 GN=GSB_911	4.92	6.04	4	1	1
A8B717	Uncharacterized protein OS=Giardia intestinalis (strain ATCC 50803 / WB	4.88	3.34	2	1	1
A0A644F8I6	Uncharacterized protein OS=Giardia intestinalis (strain ATCC 50803 / WB	4.86	5.81	6	1	1
A8BGI6	V-type proton ATPase subunit OS=Giardia intestinalis (strain ATCC 50803	4.86	5.98	5	2	2
A8BWF2	EamA-like transporter family protein OS=Giardia intestinalis (strain ATCC 5	4.85	4.50	2	1	1
A0A644F9M7	Kinesin light chain protein OS=Giardia intestinalis (strain ATCC 50803 / W	4.84	4.23	6	1	2

A8B9D9	Uncharacterized protein OS=Giardia intestinalis (strain ATCC 50803 / WB c	4.84	4.31	1	1	1
A8B3Q2	Uncharacterized protein OS=Giardia intestinalis (strain ATCC 50803 / WB c	4.83	4.73	2	2	2
A8BC93	Long chain fatty acid CoA ligase 5 OS=Giardia intestinalis (strain ATCC 508	4.83	1.25	4	2	2
A8B642	Protein YIPF OS=Giardia intestinalis (strain ATCC 50803 / WB clone C6) O	4.82	8.98	2	1	1
A8B4D2	Uncharacterized protein OS=Giardia intestinalis (strain ATCC 50803 / WB c	4.81	10.67	3	2	2
D3KGN8	Uncharacterized protein OS=Giardia intestinalis (strain ATCC 50803 / WB c	4.80	2.03	5	2	2
V6THK3	tRNA phosphoribosyl transferase OS=Giardia intestinalis OX=5741 GN=DH	4.80	4.81	2	1	1
A8B9W5	Non-specific serine/threonine protein kinase OS=Giardia intestinalis (strain	4.80	4.38	4	2	2
A8B5N5	Skp1 family, dimerization domain-containing protein OS=Giardia intestinal	4.79	27.21	5	3	3
A8B946	Uncharacterized protein OS=Giardia intestinalis (strain ATCC 50803 / WB c	4.77	2.64	3	2	2
Q9Y1T9	Transcription factor IIS homolog (Fragment) OS=Giardia intestinalis OX=5	4.77	24.29	5	1	1
A8B966	4-methyl-5-thiazole monophosphate biosynthesis enzyme OS=Giardia inte	4.75	10.75	2	1	1
A8BX15	Uncharacterized protein OS=Giardia intestinalis (strain ATCC 50803 / WB c	4.75	8.78	5	2	2
A0A644F836	tRNA-uridine aminocarboxypropyltransferase OS=Giardia intestinalis (strai	4.74	3.91	3	1	1
A8BRW3	Uncharacterized protein OS=Giardia intestinalis (strain ATCC 50803 / WB c	4.74	2.68	2	2	2
A0A644F012	Phosphatidylinositol-3,4,5-trisphosphate 3-phosphatase OS=Giardia intesti	4.73	3.26	2	1	1
E1F1V8	Uncharacterized protein OS=Giardia intestinalis (strain P15) OX=658858 G	4.73	11.36	3	2	2
C6LW67	Uncharacterized protein OS=Giardia intestinalis (strain ATCC 50581 / GS c	4.73	0.80	2	1	1
D3KGC0	Uncharacterized protein OS=Giardia intestinalis (strain ATCC 50803 / WB c	4.72	3.04	2	2	2
Q9N9W9	Proteasome subunit beta (Fragment) OS=Giardia intestinalis OX=5741 GN	4.71	12.61	6	2	2
A8BYH8	RING-type zinc-finger domain-containing protein OS=Giardia intestinalis (s	4.69	8.56	2	2	2
A8BWY0	Signal recognition particle 19 kDa protein OS=Giardia intestinalis (strain A	4.69	10.34	2	1	1
A8BKC8	Serpin 1 OS=Giardia intestinalis (strain ATCC 50803 / WB clone C6) OX=1	4.69	6.55	2	2	2
D3KHQ6	Uncharacterized protein OS=Giardia intestinalis (strain ATCC 50803 / WB c	4.67	8.56	2	2	2
A0A644EZW7	Kinase, NEK OS=Giardia intestinalis (strain ATCC 50803 / WB clone C6) O)	4.65	5.35	3	1	1
D3KG08	Uncharacterized protein OS=Giardia intestinalis (strain ATCC 50803 / WB c	4.64	12.77	6	1	1
A8BQV1	RING-type domain-containing protein OS=Giardia intestinalis (strain ATCC	4.64	1.14	2	1	1
A8B320	Uncharacterized protein OS=Giardia intestinalis (strain ATCC 50803 / WB c	4.62	3.18	2	1	1
A8BFI5	ATP-dependent RNA helicase OS=Giardia intestinalis (strain ATCC 50803 /	4.62	2.91	5	2	2
D3KHR2	Uncharacterized protein OS=Giardia intestinalis (strain ATCC 50803 / WB c	4.62	3.98	4	1	1
A8BMD0	Developmentally regulated GTP-binding protein 1 OS=Giardia intestinalis (	4.61	7.34	3	2	2
A8B299	Hexose transporter OS=Giardia intestinalis (strain ATCC 50803 / WB clone	4.59	4.10	2	1	1
A8B366	Ankyrin repeat protein 1 OS=Giardia intestinalis (strain ATCC 50803 / WB	4.59	2.46	2	1	1
A0A644F762	V-type H-transporting ATPase subunit F OS=Giardia intestinalis (strain ATC	4.59	19.59	4	2	2
A8B1S5	Ankyrin repeat protein 1 OS=Giardia intestinalis (strain ATCC 50803 / WB	4.58	4.61	3	2	2
D3KGR2	Uncharacterized protein OS=Giardia intestinalis (strain ATCC 50803 / WB c	4.54	2.39	2	1	1
A8BW27	Ankyrin repeat protein 1 OS=Giardia intestinalis (strain ATCC 50803 / WB	4.54	3.26	2	1	1
A8B746	Uncharacterized protein OS=Giardia intestinalis (strain ATCC 50803 / WB c	4.53	3.24	1	1	1
A8BT62	NYD-SP28 protein OS=Giardia intestinalis (strain ATCC 50803 / WB clone (	4.53	3.12	3	1	1

A8B4B2	Uncharacterized protein OS=Giardia intestinalis (strain ATCC 50803 / WB c	4.51	2.02	5	2	2
E1F7L7	Uncharacterized protein OS=Giardia intestinalis (strain P15) OX=658858 G	4.51	3.59	2	1	2
E1EYZ3	Protein 21.1 OS=Giardia intestinalis (strain P15) OX=658858 GN=GLP15_2	4.51	5.19	4	2	2
A8BXC6	Uncharacterized protein OS=Giardia intestinalis (strain ATCC 50803 / WB c	4.50	7.95	4	2	2
E1F4C7	Coiled-coil protein OS=Giardia intestinalis (strain P15) OX=658858 GN=GL	4.49	2.50	2	1	1
A8B8B0	WAC domain-containing protein OS=Giardia intestinalis (strain ATCC 5080	4.48	1.36	1	1	1
A8B720	Uncharacterized protein OS=Giardia intestinalis (strain ATCC 50803 / WB c	4.47	7.36	2	2	2
A0A644F0Y6	Glutamate-rich WD-repeat protein OS=Giardia intestinalis (strain ATCC 50	4.46	3.78	2	1	1
A0A644EXT6	Uncharacterized protein OS=Giardia intestinalis (strain ATCC 50803 / WB c	4.45	5.97	2	1	1
V6U941	Uncharacterized protein OS=Giardia intestinalis OX=5741 GN=GSB_15024	4.44	12.23	6	1	2
E1F691	Protein 21.1 OS=Giardia intestinalis (strain P15) OX=658858 GN=GLP15_1	4.44	1.44	3	1	1
A8B4X7	Prefoldin subunit OS=Giardia intestinalis (strain ATCC 50803 / WB clone C	4.43	13.22	3	1	1
A0A644EZX7	RER1-like protein-retention of ER protein OS=Giardia intestinalis (strain AT	4.41	10.06	6	2	2
A8BNC5	Uncharacterized protein OS=Giardia intestinalis (strain ATCC 50803 / WB c	4.40	5.93	2	1	1
A8BVU1	Caffeine-induced death protein 1-like protein OS=Giardia intestinalis (strai	4.40	2.51	3	1	1
A0A644F5E5	Uncharacterized protein OS=Giardia intestinalis (strain ATCC 50803 / WB c	4.40	6.14	2	1	1
A8BL39	Eukaryotic initiation factor 4E OS=Giardia intestinalis (strain ATCC 50803 /	4.39	9.63	4	1	1
A8B2C0	Uncharacterized protein OS=Giardia intestinalis (strain ATCC 50803 / WB c	4.39	2.15	2	1	1
A8BVE2	Uncharacterized protein OS=Giardia intestinalis (strain ATCC 50803 / WB c	4.37	34.43	1	2	2
A8BS26	Uncharacterized protein OS=Giardia intestinalis (strain ATCC 50803 / WB c	4.37	9.84	1	1	1
D3KI37	Uncharacterized protein OS=Giardia intestinalis (strain ATCC 50803 / WB c	4.36	1.45	2	1	1
C6LTR8	Uncharacterized protein OS=Giardia intestinalis (strain ATCC 50581 / GS c	4.35	0.82	5	1	1
A8BZH8	Calmodulin OS=Giardia intestinalis (strain ATCC 50803 / WB clone C6) OX	4.35	18.01	3	1	1
D3KGI2	Uncharacterized protein OS=Giardia intestinalis (strain ATCC 50803 / WB c	4.35	4.02	2	1	1
A8BCL6	Uncharacterized protein OS=Giardia intestinalis (strain ATCC 50803 / WB c	4.32	1.51	2	2	2
E1F0W9	Alcohol dehydrogenase lateral transfer candidate OS=Giardia intestinalis (s	4.30	4.42	3	3	3
A8BGB4	V-type proton ATPase subunit a OS=Giardia intestinalis (strain ATCC 5080	4.30	1.61	2	1	1
A0A644EZZ4	S-adenosylmethionine synthetase OS=Giardia intestinalis (strain ATCC 508	4.29	4.63	5	1	1
A8BAG8	Ankyrin repeat protein 1 OS=Giardia intestinalis (strain ATCC 50803 / WB	4.29	4.96	1	1	1
V6TGL0	Ubiquitin-like protein SUMO OS=Giardia intestinalis OX=5741 GN=DHA2_7	4.29	13.73	1	1	1
A8BT44	Poly(A) polymerase OS=Giardia intestinalis (strain ATCC 50803 / WB clone	4.28	5.97	2	3	3
B3FXL8	1-acyl-sn-glycerol-3-phosphate acyl transferase 2 (Fragment) OS=Giardia	4.25	8.00	3	1	1
E2RTV5	Disc-associated protein OS=Giardia intestinalis (strain ATCC 50803 / WB c	4.25	5.95	2	1	1
A8B5M0	Ankyrin repeat protein 1 OS=Giardia intestinalis (strain ATCC 50803 / WB	4.24	1.37	2	1	1
A6QL36	Putative RNase L inhibitor-like protein OS=Giardia intestinalis OX=5741 GI	4.24	2.08	3	1	1
A8BCC6	Uncharacterized protein OS=Giardia intestinalis (strain ATCC 50803 / WB c	4.24	3.04	2	2	2
A8BWI4	Cathepsin L OS=Giardia intestinalis (strain ATCC 50803 / WB clone C6) OX	4.24	3.29	2	1	1
A8BQF9	Coiled-coil protein OS=Giardia intestinalis (strain ATCC 50803 / WB clone c	4.22	1.43	2	1	1
A0A644EZI2	Alpha-snap OS=Giardia intestinalis (strain ATCC 50803 / WB clone C6) OX	4.22	6.74	5	2	2

D3KH83	Uncharacterized protein OS=Giardia intestinalis (strain ATCC 50803 / WB c	4.22	3.32	2	1	1
A8BW33	Trimethylguanosine synthase OS=Giardia intestinalis (strain ATCC 50803 /	4.19	7.75	2	1	1
E1F4U6	Uncharacterized protein OS=Giardia intestinalis (strain P15) OX=658858 G	4.19	5.50	3	1	1
A8BMB7	Uncharacterized protein OS=Giardia intestinalis (strain ATCC 50803 / WB c	4.19	4.98	4	1	2
A8BYS6	Ankyrin repeat protein 2 OS=Giardia intestinalis (strain ATCC 50803 / WB	4.19	6.99	2	2	2
C6LS37	Protein 21.1 OS=Giardia intestinalis (strain ATCC 50581 / GS clone H7) OX	4.17	1.60	8	1	2
A8BZD5	Uncharacterized protein OS=Giardia intestinalis (strain ATCC 50803 / WB c	4.15	3.20	3	1	2
A8B2J7	Putative Ribosome biogenesis protein NEP1 OS=Giardia intestinalis (strain	4.14	10.39	5	1	1
A8BR52	Uncharacterized protein OS=Giardia intestinalis (strain ATCC 50803 / WB c	4.12	6.28	2	1	1
V6TE24	SSU ribosomal protein S27AE / Ubiquitin OS=Giardia intestinalis OX=5741	4.11	13.87	1	1	1
E1EVV8	RNA polymerase II subunit Rpb11 OS=Giardia intestinalis (strain P15) OX=	4.11	10.69	3	1	1
A8BZ31	Uncharacterized protein OS=Giardia intestinalis (strain ATCC 50803 / WB c	4.09	2.40	3	1	1
D3KHL5	Uncharacterized protein OS=Giardia intestinalis (strain ATCC 50803 / WB c	4.09	2.77	2	1	1
A8BC09	Uncharacterized protein OS=Giardia intestinalis (strain ATCC 50803 / WB c	4.09	4.02	2	1	1
A0A644EYA2	LSM domain-containing protein OS=Giardia intestinalis (strain ATCC 50803	4.07	17.59	2	1	1
A8BJP4	Uncharacterized protein OS=Giardia intestinalis (strain ATCC 50803 / WB c	4.07	3.62	1	1	1
C6LYX0	Uncharacterized protein OS=Giardia intestinalis (strain ATCC 50581 / GS c	4.07	2.50	5	1	1
V6TET0	Putative rhodanese-like proteiny domain superfamily protein OS=Giardia ir	4.06	12.50	1	1	1
A8BNH3	Uncharacterized protein OS=Giardia intestinalis (strain ATCC 50803 / WB c	4.06	3.87	2	1	1
A8BAW3	Kinase, NEK OS=Giardia intestinalis (strain ATCC 50803 / WB clone C6) OX	4.05	1.27	2	1	1
A8BRX4	Sec24-like OS=Giardia intestinalis (strain ATCC 50803 / WB clone C6) OX=	4.05	1.61	1	1	1
A8BC99	Uncharacterized protein OS=Giardia intestinalis (strain ATCC 50803 / WB c	4.04	1.28	2	1	1
A8BYP9	p24, putative OS=Giardia intestinalis (strain ATCC 50803 / WB clone C6) C	4.04	18.18	4	1	1
A8BI65	DUF4485 domain-containing protein OS=Giardia intestinalis (strain ATCC 5	4.04	1.73	2	1	1
A8BBT0	Uncharacterized protein OS=Giardia intestinalis (strain ATCC 50803 / WB c	4.02	4.80	4	1	1
A8BP02	Uncharacterized protein OS=Giardia intestinalis (strain ATCC 50803 / WB c	4.01	1.21	2	1	1
E1F8J3	Kinesin like protein OS=Giardia intestinalis (strain P15) OX=658858 GN=G	4.01	1.63	5	1	1
A8BUX7	Kinase, NEK OS=Giardia intestinalis (strain ATCC 50803 / WB clone C6) OX	4.00	2.70	3	2	2
A8BHF9	MORN repeat-containing protein 5 OS=Giardia intestinalis (strain ATCC 50	4.00	13.33	3	1	1
D3KGV8	Uncharacterized protein OS=Giardia intestinalis (strain ATCC 50803 / WB c	3.99	1.37	1	1	1
A8BFG6	Chromatin associated protein KTI12 OS=Giardia intestinalis (strain ATCC 5	3.98	4.08	1	1	1
E1EWC8	Copine I OS=Giardia intestinalis (strain P15) OX=658858 GN=GLP15_519	3.98	7.48	2	1	1
A8BQK4	Ubiquitin-conjugating enzyme E2-28.4 kDa OS=Giardia intestinalis (strain	3.96	13.66	3	1	1
A8BUG4	Uncharacterized protein OS=Giardia intestinalis (strain ATCC 50803 / WB c	3.96	3.61	3	1	1
A8BE38	Malate dehydrogenase OS=Giardia intestinalis (strain ATCC 50803 / WB cl	3.96	2.51	3	1	1
A8B8T0	Uncharacterized protein OS=Giardia intestinalis (strain ATCC 50803 / WB c	3.95	7.34	3	1	1
A8BW14	Protein SEY1 homolog OS=Giardia intestinalis (strain ATCC 50803 / WB cl	3.95	1.45	2	1	1
A0A644EZ80	Uncharacterized protein OS=Giardia intestinalis (strain ATCC 50803 / WB c	3.94	2.37	2	1	1
C6LNA0	Polysacc_synt_4 domain-containing protein OS=Giardia intestinalis (strain	3.94	13.10	1	1	2

A0A644F0P9	20S proteasome alpha subunit 7 OS=Giardia intestinalis (strain ATCC 508(	3.91	7.35	5	2	2
A8BHC9	Uncharacterized protein OS=Giardia intestinalis (strain ATCC 50803 / WB (	3.90	15.79	2	1	1
A8BXK0	Uncharacterized protein OS=Giardia intestinalis (strain ATCC 50803 / WB (	3.90	5.05	2	1	1
A8BED1	Putative Spindle pole protein OS=Giardia intestinalis (strain ATCC 50803 /	3.90	2.72	3	1	1
D3KGK2	Ankyrin repeat protein 1 OS=Giardia intestinalis (strain ATCC 50803 / WB	3.90	3.36	2	1	1
A8BB64	Uncharacterized protein OS=Giardia intestinalis (strain ATCC 50803 / WB (	3.89	4.67	3	1	1
D3KI76	Uncharacterized protein OS=Giardia intestinalis (strain ATCC 50803 / WB (	3.89	1.74	1	1	1
D3KHZ1	Annexin OS=Giardia intestinalis (strain ATCC 50803 / WB clone C6) OX=1	3.88	5.62	4	2	2
A0A644F713	Sec61 alpha family protein OS=Giardia intestinalis (strain ATCC 50803 / W	3.87	2.54	6	1	1
Q9N9X0	Proteasome subunit beta OS=Giardia intestinalis OX=5741 GN=beta 5_Gl	3.87	5.36	6	1	1
C6LWK5	Sec1, putative OS=Giardia intestinalis (strain ATCC 50581 / GS clone H7) (	3.86	2.74	2	1	1
A0A644F462	Proteasome activator pa28 beta subunit OS=Giardia intestinalis (strain AT(	3.85	5.06	4	1	1
A8BIE7	Uncharacterized protein OS=Giardia intestinalis (strain ATCC 50803 / WB (	3.85	2.29	1	1	1
A8BN58	Kinesin-like protein OS=Giardia intestinalis (strain ATCC 50803 / WB clone	3.84	2.93	4	1	2
A8BSQ4	Uncharacterized protein OS=Giardia intestinalis (strain ATCC 50803 / WB (	3.82	5.56	3	1	1
V6T9C1	GTPase OS=Giardia intestinalis OX=5741 GN=DHA2_16979 PE=4 SV=1 -	3.82	4.38	2	1	1
A8BCP4	Phospholipid-transporting ATPase IIB, putative OS=Giardia intestinalis (str	3.82	1.26	2	1	1
D3KG98	Uncharacterized protein OS=Giardia intestinalis (strain ATCC 50803 / WB (	3.81	2.34	2	1	1
Q8MZS1	Putative adaptor protein complex large chain subunit BetaA OS=Giardia in	3.80	1.93	3	1	1
E1EY28	Malate dehydrogenase OS=Giardia intestinalis (strain P15) OX=658858 GN	3.80	5.14	3	1	1
V6TE23	Uncharacterized protein OS=Giardia intestinalis OX=5741 GN=DHA2_150{	3.80	8.78	2	1	1
A8BFJ6	MYND finger domain-containing protein OS=Giardia intestinalis (strain AT(	3.79	1.82	2	1	1
A8B4B7	Uncharacterized protein OS=Giardia intestinalis (strain ATCC 50803 / WB (	3.79	6.45	2	1	1
D3KI69	Uncharacterized protein OS=Giardia intestinalis (strain ATCC 50803 / WB (	3.78	1.57	1	1	1
A8BQI8	Uncharacterized protein OS=Giardia intestinalis (strain ATCC 50803 / WB (	3.77	5.91	1	1	1
D3KGQ9	Uncharacterized protein OS=Giardia intestinalis (strain ATCC 50803 / WB (	3.77	6.86	3	1	1
A8B787	MYG1 protein OS=Giardia intestinalis (strain ATCC 50803 / WB clone C6) (	3.76	6.09	1	1	1
A8B4B0	Uncharacterized protein OS=Giardia intestinalis (strain ATCC 50803 / WB (	3.75	8.33	2	1	1
A8BQA4	Methyltransferase OS=Giardia intestinalis (strain ATCC 50803 / WB clone (	3.74	4.02	2	1	1
A8B4M7	Uncharacterized protein OS=Giardia intestinalis (strain ATCC 50803 / WB (	3.73	10.81	3	1	1
A8BA73	Uncharacterized protein OS=Giardia intestinalis (strain ATCC 50803 / WB (	3.73	7.06	1	1	1
A8B7Y8	Uncharacterized protein OS=Giardia intestinalis (strain ATCC 50803 / WB (	3.73	1.76	4	1	1
A8BYY6	Uncharacterized protein OS=Giardia intestinalis (strain ATCC 50803 / WB (	3.73	9.41	2	1	1
A8BVE9	Ankyrin repeat protein 1 OS=Giardia intestinalis (strain ATCC 50803 / WB	3.72	5.04	5	1	1
V6TKU5	FeS_assembly_P domain-containing protein OS=Giardia intestinalis OX=57	3.70	13.10	1	1	1
V6TU74	Uncharacterized protein OS=Giardia intestinalis OX=5741 GN=GSB_5890	3.69	9.80	4	1	1
D3KGJ8	Uncharacterized protein OS=Giardia intestinalis (strain ATCC 50803 / WB (	3.69	2.66	2	1	1
A8B893	Uncharacterized protein OS=Giardia intestinalis (strain ATCC 50803 / WB (	3.67	2.57	2	1	1
A8BLY8	Intraflagellar transport protein IFT46/ FAP32 OS=Giardia intestinalis (strai	3.67	7.21	1	1	1

A8BW36	HMG (High mobility group) box-containing protein OS=Giardia intestinalis	3.67	6.86	2	1	1
A8BDM4	DNA-directed DNA polymerase OS=Giardia intestinalis (strain ATCC 50803	3.67	0.62	2	1	1
A0A644FBQ5	Uncharacterized protein OS=Giardia intestinalis (strain ATCC 50803 / WB	3.66	5.53	3	2	2
A8BEZ9	Uncharacterized protein OS=Giardia intestinalis (strain ATCC 50803 / WB	3.66	5.02	3	1	1
A8B4P8	CYTH domain-containing protein OS=Giardia intestinalis (strain ATCC 508	3.66	5.58	2	1	1
A8BRL8	Uncharacterized protein OS=Giardia intestinalis (strain ATCC 50803 / WB	3.66	3.82	2	1	1
A8BLK6	Uncharacterized protein OS=Giardia intestinalis (strain ATCC 50803 / WB	3.64	10.16	1	1	1
A8BD07	Rab GTPase-like family protein OS=Giardia intestinalis (strain ATCC 50803	3.64	7.30	5	1	1
V6U2X6	Ankyrin repeat protein OS=Giardia intestinalis OX=5741 GN=GSB_151087	3.63	0.81	4	1	1
E1F1U7	DDI1-like DNA-damage inducible protein OS=Giardia intestinalis (strain P1	3.63	7.00	2	1	1
A8BBA9	Cornichon protein OS=Giardia intestinalis (strain ATCC 50803 / WB clone	3.63	7.86	3	1	1
V6TKU0	Mitogen-activated protein kinase kinase kinase OS=Giardia intestinalis OX	3.63	2.47	1	1	1
D3KG43	Ankyrin repeat protein 1 OS=Giardia intestinalis (strain ATCC 50803 / WB	3.62	2.71	2	1	1
A8BCZ2	Coiled-coil protein OS=Giardia intestinalis (strain ATCC 50803 / WB clone	3.62	1.37	3	1	1
A8B8I9	Putative U3 small nucleolar ribonucleoprotein IMP3 OS=Giardia intestinalis	3.61	8.15	4	1	1
A0A644EZI6	Ankyrin repeat protein 1 OS=Giardia intestinalis (strain ATCC 50803 / WB	3.60	1.98	6	2	2
A8BHG9	Uncharacterized protein OS=Giardia intestinalis (strain ATCC 50803 / WB	3.59	7.14	3	1	1
D3KHG0	Uncharacterized protein OS=Giardia intestinalis (strain ATCC 50803 / WB	3.59	1.73	2	1	1
A8BDY6	Uncharacterized protein OS=Giardia intestinalis (strain ATCC 50803 / WB	3.58	4.27	3	1	1
D3KG06	Uncharacterized protein OS=Giardia intestinalis (strain ATCC 50803 / WB	3.58	4.60	1	1	1
A8B4W1	PHD domain-containing protein OS=Giardia intestinalis (strain ATCC 5080	3.57	1.36	2	1	1
A8BEC1	Uncharacterized protein OS=Giardia intestinalis (strain ATCC 50803 / WB	3.57	3.73	2	1	1
A8B4N1	Uncharacterized protein OS=Giardia intestinalis (strain ATCC 50803 / WB	3.56	5.21	2	1	1
D3KGM6	Uncharacterized protein OS=Giardia intestinalis (strain ATCC 50803 / WB	3.56	1.54	3	1	1
A8B8V5	Ankyrin repeat protein 1 OS=Giardia intestinalis (strain ATCC 50803 / WB	3.56	4.34	2	1	1
A0A644EXS9	Uncharacterized protein OS=Giardia intestinalis (strain ATCC 50803 / WB	3.55	5.05	5	2	2
Q964G8	L-serine dehydratase SD1 OS=Giardia intestinalis OX=5741 PE=4 SV=1 -	3.54	3.93	4	1	1
A8B4V8	Uncharacterized protein OS=Giardia intestinalis (strain ATCC 50803 / WB	3.54	10.10	2	1	1
A0A644F391	Transporter, MFS superfamily protein OS=Giardia intestinalis (strain ATCC	3.54	2.40	4	1	1
A8BB99	Uncharacterized protein OS=Giardia intestinalis (strain ATCC 50803 / WB	3.53	5.74	3	1	1
A8BLM1	ELMO/CED-12 family protein OS=Giardia intestinalis (strain ATCC 50803 /	3.51	6.64	3	1	1
A8BE77	Partner of Nob1 OS=Giardia intestinalis (strain ATCC 50803 / WB clone C6	3.50	7.46	3	1	1
A8B564	Rho GDI OS=Giardia intestinalis (strain ATCC 50803 / WB clone C6) OX=1	3.48	9.45	2	1	1
A8BK53	Uncharacterized protein OS=Giardia intestinalis (strain ATCC 50803 / WB	3.48	4.31	2	1	1
A8BEU4	Uncharacterized protein OS=Giardia intestinalis (strain ATCC 50803 / WB	3.48	0.47	2	1	1
A8BK56	Ankyrin repeat protein 1 OS=Giardia intestinalis (strain ATCC 50803 / WB	3.47	4.07	2	1	1
A0A644F9K3	Uncharacterized protein OS=Giardia intestinalis (strain ATCC 50803 / WB	3.46	2.55	3	1	1
A8BJB2	Uncharacterized protein OS=Giardia intestinalis (strain ATCC 50803 / WB	3.46	0.82	2	1	1
A8BII2	Proteasome subunit beta OS=Giardia intestinalis (strain ATCC 50803 / WB	3.46	9.68	5	2	2

A8BC83	ATPases of the PP-loop superfamily OS=Giardia intestinalis (strain ATCC 5	3.46	6.98	3	1	1
V6U4T6	Uncharacterized protein OS=Giardia intestinalis OX=5741 GN=GSB_15254	3.46	4.59	5	1	1
A8B1Z6	Ankyrin repeat protein 1 OS=Giardia intestinalis (strain ATCC 50803 / WB	3.45	0.93	1	1	1
V6T9G4	Transcriptional regulator, Xre family protein OS=Giardia intestinalis OX=57	3.44	19.63	2	1	1
A8BBP6	Ubiquitin-conjugating enzyme E1 OS=Giardia intestinalis (strain ATCC 508	3.43	1.10	2	1	1
A0A644F9X3	Ankyrin repeat protein 1 OS=Giardia intestinalis (strain ATCC 50803 / WB	3.43	1.53	4	1	1
A8BQ63	Uncharacterized protein OS=Giardia intestinalis (strain ATCC 50803 / WB c	3.43	2.07	2	1	1
A8BUM7	Uncharacterized protein OS=Giardia intestinalis (strain ATCC 50803 / WB c	3.42	3.02	3	1	1
D3KI07	Uncharacterized protein OS=Giardia intestinalis (strain ATCC 50803 / WB c	3.42	1.54	2	1	1
A8BRM2	Signal transduction protein OS=Giardia intestinalis (strain ATCC 50803 / W	3.42	6.55	2	1	1
E1F184	Pyridoxal phosphate homeostasis protein OS=Giardia intestinalis (strain P1	3.40	5.45	2	1	1
A0A644F354	Uncharacterized protein OS=Giardia intestinalis (strain ATCC 50803 / WB c	3.40	0.81	3	1	1
E1F731	Uncharacterized protein OS=Giardia intestinalis (strain P15) OX=658858 G	3.39	5.49	3	1	1
A8B3R0	FixW protein, putative OS=Giardia intestinalis (strain ATCC 50803 / WB clo	3.38	6.64	4	1	1
A8BLW7	Uncharacterized protein OS=Giardia intestinalis (strain ATCC 50803 / WB c	3.37	2.05	4	2	2
A8B7J7	Mediator of RNA polymerase II transcription subunit 6 OS=Giardia intestin	3.37	10.59	4	2	2
E1EXF6	Ribose-phosphate pyrophosphokinase OS=Giardia intestinalis (strain P15)	3.36	3.78	3	1	1
A0A644F5J3	tRNA (guanine(9)-N(1))-methyltransferase OS=Giardia intestinalis (strain A	3.35	6.41	3	1	1
E1F644	DNA-directed RNA polymerase II RPB6 OS=Giardia intestinalis (strain P15)	3.35	12.50	2	1	1
A8BPN1	Kinase, NEK OS=Giardia intestinalis (strain ATCC 50803 / WB clone C6) OX	3.35	2.21	2	1	1
E1F273	Kinase, putative OS=Giardia intestinalis (strain P15) OX=658858 GN=GLP	3.34	5.42	2	1	1
D3KG23	Uncharacterized protein OS=Giardia intestinalis (strain ATCC 50803 / WB c	3.34	2.27	2	1	1
A8BBZ4	ASF1 like histone chaperone OS=Giardia intestinalis (strain ATCC 50803 /	3.33	8.15	1	1	1
A8BW22	Nicotinamide-nucleotide adenylyltransferase OS=Giardia intestinalis (strain	3.33	5.73	2	1	1
A8B9C7	Putative Rho GAP OS=Giardia intestinalis (strain ATCC 50803 / WB clone C	3.33	7.58	2	1	1
A0A644F577	VSP OS=Giardia intestinalis (strain ATCC 50803 / WB clone C6) OX=18492	3.32	12.63	4	1	1
A0A644EZP4	Uncharacterized protein OS=Giardia intestinalis (strain ATCC 50803 / WB c	3.32	9.41	4	2	2
A8B672	Flap endonuclease 1 OS=Giardia intestinalis (strain ATCC 50803 / WB clon	3.32	3.32	3	1	1
A8BM27	Nuclear ATP/GTP-binding protein OS=Giardia intestinalis (strain ATCC 508	3.32	0.76	2	1	1
V6TJ31	Uncharacterized protein OS=Giardia intestinalis OX=5741 GN=DHA2_1505	3.31	6.42	1	1	1
V6TBG2	Nuclear transport factor 2 OS=Giardia intestinalis OX=5741 GN=DHA2_67	3.30	9.02	1	1	1
A0A644FBQ8	Ankyrin repeat protein 1 OS=Giardia intestinalis (strain ATCC 50803 / WB	3.29	7.24	2	1	1
A8BW28	Kinase, NEK OS=Giardia intestinalis (strain ATCC 50803 / WB clone C6) OX	3.29	1.35	3	1	1
A8BS98	Meiosis-specific nuclear structural protein 1 OS=Giardia intestinalis (strain	3.29	2.23	1	1	1
A0A644F8J5	O-sialoglycoprotein endopeptidase OS=Giardia intestinalis (strain ATCC 50	3.29	5.29	4	1	1
C6LYN6	Uncharacterized protein OS=Giardia intestinalis (strain ATCC 50581 / GS c	3.28	4.65	2	1	2
D3KG10	WD40 repeat protein OS=Giardia intestinalis (strain ATCC 50803 / WB clon	3.28	0.84	1	1	1
A8B455	Uncharacterized protein OS=Giardia intestinalis (strain ATCC 50803 / WB c	3.27	4.17	1	1	1
A8B4C9	Pantothenate kinase 4 OS=Giardia intestinalis (strain ATCC 50803 / WB cl	3.27	4.18	3	1	1

A8BSH3	Transcriptional regulator OS=Giardia intestinalis (strain ATCC 50803 / WB	3.27	1.63	3	1	1
A8BMX2	GTP-binding protein ARD-1, putative OS=Giardia intestinalis (strain ATCC	3.26	2.51	5	1	1
A0A482ERN7	Putative NADPH oxidoreductase OS=Giardia intestinalis OX=5741 GN=DH	3.26	7.32	5	1	1
D3KGA0	Uncharacterized protein OS=Giardia intestinalis (strain ATCC 50803 / WB	3.26	3.13	1	1	1
E1F1Y7	tRNA(Ile)-2-lysyl-cytidine synthase OS=Giardia intestinalis (strain P15) OX	3.26	3.88	9	1	1
V6TQA4	Ubiquitin-conjugating enzyme E2 OS=Giardia intestinalis OX=5741 GN=D	3.25	6.78	1	1	1
E1EYM3	Ubiquitin-conjugating enzyme E2-17 kDa OS=Giardia intestinalis (strain P	3.25	8.86	2	1	1
V6T8E4	Uncharacterized protein OS=Giardia intestinalis OX=5741 GN=DHA2_358	3.24	8.96	1	1	1
A8BMQ1	Putative Yip interacting protein OS=Giardia intestinalis (strain ATCC 5080	3.24	6.25	3	1	1
A8BYX6	Acid phosphatase OS=Giardia intestinalis (strain ATCC 50803 / WB clone	3.24	7.79	2	1	1
A8B7L1	Uncharacterized protein OS=Giardia intestinalis (strain ATCC 50803 / WB	3.23	1.19	2	1	1
A8BX05	HZGJ OS=Giardia intestinalis (strain ATCC 50803 / WB clone C6) OX=184	3.22	6.65	2	1	1
A8B3D0	Putative tRNA (cytidine(32)/guanosine(34)-2'-O)-methyltransferase OS=G	3.22	4.48	2	1	1
D3KIA9	CNDH2_N domain-containing protein OS=Giardia intestinalis (strain ATCC	3.21	2.60	2	1	1
A8BEB7	Uncharacterized protein OS=Giardia intestinalis (strain ATCC 50803 / WB	3.20	1.37	2	2	2
A8BKG7	Diphosphomevalonate decarboxylase OS=Giardia intestinalis (strain ATCC	3.20	5.07	2	1	1
E1F8E8	Periodic tryptophan protein 1, putative OS=Giardia intestinalis (strain P15)	3.20	2.73	4	1	1
A8BPS6	Ankyrin repeat protein 2 OS=Giardia intestinalis (strain ATCC 50803 / WB	3.20	1.87	1	1	1
E1F5L4	Protein 21.1 OS=Giardia intestinalis (strain P15) OX=658858 GN=GLP15_	3.19	3.21	4	2	2
A0A644F4H9	Uncharacterized protein OS=Giardia intestinalis (strain ATCC 50803 / WB	3.18	5.23	3	1	1
A0A644EXP1	Cell motility MEMO family protein OS=Giardia intestinalis (strain ATCC 508	3.18	8.42	4	2	2
A8BD91	RNA polymerase I subunit A43 OS=Giardia intestinalis (strain ATCC 50803	3.17	5.68	4	1	1
A8B3N8	Uncharacterized protein OS=Giardia intestinalis (strain ATCC 50803 / WB	3.17	5.70	2	1	1
A8B5G5	Putative RNA methylase OS=Giardia intestinalis (strain ATCC 50803 / WB	3.17	3.63	2	1	1
A0A644FC05	Putative Sec1 OS=Giardia intestinalis (strain ATCC 50803 / WB clone C6)	3.17	3.17	3	1	1
E1EZ01	Protein kinase domain-containing protein OS=Giardia intestinalis (strain P	3.16	1.71	1	1	1
A0A644EYL3	ABC transporter, ATP-binding protein OS=Giardia intestinalis (strain ATCC	3.15	1.77	3	1	1
A8BYL5	Dpy-30 motif-containing protein OS=Giardia intestinalis (strain ATCC 5080	3.15	1.35	1	1	1
A8BSG2	Threonyl-tRNA synthetase OS=Giardia intestinalis (strain ATCC 50803 / W	3.14	1.58	4	1	1
A8B5Z9	Ankyrin repeat protein 1 OS=Giardia intestinalis (strain ATCC 50803 / WB	3.13	1.59	2	1	1
D3KH91	Uncharacterized protein OS=Giardia intestinalis (strain ATCC 50803 / WB	3.13	1.29	3	1	1
D3KGS5	Uncharacterized protein OS=Giardia intestinalis (strain ATCC 50803 / WB	3.13	5.10	2	1	1
D3KH44	Eukaryotic initiation factor 4E OS=Giardia intestinalis (strain ATCC 50803 /	3.12	4.24	3	1	1
E1F5C6	U3 small nucleolar RNA-associated protein 11, putative OS=Giardia intesti	3.12	5.83	4	1	1
A8B793	Ankyrin repeat protein 1 OS=Giardia intestinalis (strain ATCC 50803 / WB	3.11	0.83	2	1	1
V6U196	Dynein light chain roadblock OS=Giardia intestinalis OX=5741 GN=GSB_1	3.10	10.20	3	1	1
A8BKL5	Nuclear LIM interactor-interacting factor 1 OS=Giardia intestinalis (strain	3.09	2.55	3	1	1
E1F540	Uncharacterized protein OS=Giardia intestinalis (strain P15) OX=658858	3.09	6.88	3	1	1
A8BCP8	Actin related protein OS=Giardia intestinalis (strain ATCC 50803 / WB clon	3.09	3.78	3	1	1

A8BCK3	Coiled-coil protein OS=Giardia intestinalis (strain ATCC 50803 / WB clone	3.09	2.67	4	2	2
A8BTI9	Myotubularin-like phosphatase OS=Giardia intestinalis (strain ATCC 50803	3.08	1.84	2	1	1
E1F8B0	Kinesin motor domain-containing protein OS=Giardia intestinalis (strain P1	3.08	3.75	2	1	1
C6LZV0	Ribokinase OS=Giardia intestinalis (strain ATCC 50581 / GS clone H7) OX=	3.08	5.81	1	1	1
E1F7L1	Centrin OS=Giardia intestinalis (strain P15) OX=658858 GN=GLP15_3166	3.07	8.07	1	1	1
A8BFW1	Uncharacterized protein OS=Giardia intestinalis (strain ATCC 50803 / WB	3.07	2.71	4	1	1
V6TS76	Thioredoxin-like protein OS=Giardia intestinalis OX=5741 GN=GSB_9355	3.07	14.91	4	1	1
A8BVJ9	Uncharacterized protein OS=Giardia intestinalis (strain ATCC 50803 / WB	3.07	0.75	2	1	1
D3KHX4	Uncharacterized protein OS=Giardia intestinalis (strain ATCC 50803 / WB	3.06	1.10	2	1	1
A8B7B3	Cytidine deaminase OS=Giardia intestinalis (strain ATCC 50803 / WB clone	3.06	21.64	4	2	2
A8BBZ0	Rab-GAP TBC domain-containing protein OS=Giardia intestinalis (strain AT	3.06	3.05	3	1	1
A8BR06	Myotubularin-like protein OS=Giardia intestinalis (strain ATCC 50803 / WB	3.05	1.27	2	1	1
V6T9Y9	Cilia- and flagella-associated protein 299 OS=Giardia intestinalis OX=5741	3.05	5.58	2	1	1
A8B7P0	Uncharacterized protein OS=Giardia intestinalis (strain ATCC 50803 / WB	3.05	2.76	2	1	1
A8B9K0	Uncharacterized protein OS=Giardia intestinalis (strain ATCC 50803 / WB	3.05	1.14	1	1	1
A8BA91	Trafficking protein particle complex subunit OS=Giardia intestinalis (strain	3.05	6.42	2	1	1
A8BLU4	Uncharacterized protein OS=Giardia intestinalis (strain ATCC 50803 / WB	3.04	3.67	2	1	1
A8BW19	Queuine tRNA-ribosyltransferase catalytic subunit 1 OS=Giardia intestinali	3.03	4.23	3	1	1
V6TGK6	Regulatory subunit of type II PKA R-subunit OS=Giardia intestinalis OX=5	3.03	21.43	1	1	1
D3KG88	Uncharacterized protein OS=Giardia intestinalis (strain ATCC 50803 / WB	3.02	3.65	2	1	1
V6TKB9	Putative TB2/DP1, HVA22 family protein OS=Giardia intestinalis OX=5741	3.02	11.01	3	1	1
A8BQJ1	SET domain-containing protein OS=Giardia intestinalis (strain ATCC 50803	3.01	4.16	2	1	1
A8B5V3	Ankyrin repeat protein 2 OS=Giardia intestinalis (strain ATCC 50803 / WB	3.01	1.32	2	1	1
A8BXE1	Ubiquitin/Ribosomal protein L40e OS=Giardia intestinalis (strain ATCC 508	3.00	7.87	3	1	1
E9M2K3	Histone H4 (Fragment) OS=Giardia intestinalis OX=5741 GN=h4 PE=3 SV	3.00	16.67	4	1	1
D3KH02	Uncharacterized protein OS=Giardia intestinalis (strain ATCC 50803 / WB	2.99	1.81	2	1	1
A8B2K9	Tetratricopeptide repeat protein OS=Giardia intestinalis (strain ATCC 5080	2.99	1.38	3	1	1
A8BYL1	Uncharacterized protein OS=Giardia intestinalis (strain ATCC 50803 / WB	2.99	2.85	2	1	1
A8B4G5	Ankyrin repeat protein 1 OS=Giardia intestinalis (strain ATCC 50803 / WB	2.99	1.27	2	1	1
A8BTT3	Putative Spindle pole protein OS=Giardia intestinalis (strain ATCC 50803 /	2.99	3.60	3	1	2
A8BT24	Kinase, NEK OS=Giardia intestinalis (strain ATCC 50803 / WB clone C6) O	2.98	0.86	2	1	1
V6TD34	ANK_REP_REGION domain-containing protein OS=Giardia intestinalis OX=	2.98	2.30	3	1	1
A8BJM2	Uncharacterized protein OS=Giardia intestinalis (strain ATCC 50803 / WB	2.98	3.24	2	1	1
A8BNP2	Uncharacterized protein OS=Giardia intestinalis (strain ATCC 50803 / WB	2.98	6.25	1	1	1
A0A644FB10	Src-associated protein-like protein OS=Giardia intestinalis (strain ATCC 50	2.97	2.21	4	1	1
A8BI91	Uncharacterized protein OS=Giardia intestinalis (strain ATCC 50803 / WB	2.97	1.03	2	1	1
A8B232	Uncharacterized protein OS=Giardia intestinalis (strain ATCC 50803 / WB	2.97	5.84	2	1	1
C6LNL0	IgE-dependent histamine-releasing factor, putative OS=Giardia intestinalis	2.96	7.28	3	1	1
A8BR33	Uncharacterized protein OS=Giardia intestinalis (strain ATCC 50803 / WB	2.96	7.07	3	1	1

D3KH20	Uncharacterized protein OS=Giardia intestinalis (strain ATCC 50803 / WB c	2.95	0.75	3	1	1
E1F6J4	Splicing factor-like protein, putative OS=Giardia intestinalis (strain P15) OX	2.94	0.48	3	1	1
V6T886	Uncharacterized protein OS=Giardia intestinalis OX=5741 GN=DHA2_7007	2.94	13.95	2	1	1
A0A644FAU9	Uncharacterized protein OS=Giardia intestinalis (strain ATCC 50803 / WB c	2.93	1.90	4	1	1
A8B669	Kinase, NEK OS=Giardia intestinalis (strain ATCC 50803 / WB clone C6) OX	2.93	4.38	2	1	1
A8BTV9	ATP binding protein associated with cell differentiation, putative OS=Giard	2.93	10.87	4	2	2
A0A644FBS7	Uncharacterized protein OS=Giardia intestinalis (strain ATCC 50803 / WB c	2.93	4.16	2	1	1
A8BV49	WD_REPEATS_REGION domain-containing protein OS=Giardia intestinalis	2.92	0.41	3	1	1
E1EWX0	Kinase, NEK OS=Giardia intestinalis (strain P15) OX=658858 GN=GLP15_4	2.92	6.32	1	1	1
A8B4B8	Kinase, CMGC CMGC-GL1 OS=Giardia intestinalis (strain ATCC 50803 / WB	2.92	3.75	3	1	1
A8B7M0	Alpha-3 giardin OS=Giardia intestinalis (strain ATCC 50803 / WB clone C6)	2.92	4.39	4	1	1
A8BBQ8	Uncharacterized protein OS=Giardia intestinalis (strain ATCC 50803 / WB c	2.91	5.81	2	1	1
D3KG22	Uncharacterized protein OS=Giardia intestinalis (strain ATCC 50803 / WB c	2.90	0.91	2	1	1
A0A644F228	Uncharacterized protein OS=Giardia intestinalis (strain ATCC 50803 / WB c	2.89	6.58	4	1	1
A8BSZ4	Uncharacterized protein OS=Giardia intestinalis (strain ATCC 50803 / WB c	2.88	2.56	2	1	1
A8BES0	Coiled-coil protein OS=Giardia intestinalis (strain ATCC 50803 / WB clone C	2.88	0.97	2	1	1
A8BQK8	Ankyrin repeat protein 1 OS=Giardia intestinalis (strain ATCC 50803 / WB	2.88	3.28	2	1	1
A8B6X8	Leucine zipper transcription factor-like protein 1 OS=Giardia intestinalis (st	2.88	5.24	2	1	1
V6T880	Synaptic glycoprotein SC2 OS=Giardia intestinalis OX=5741 GN=DHA2_88	2.87	3.48	1	1	1
E1EY18	Nuclear ATP/GTP-binding protein OS=Giardia intestinalis (strain P15) OX=	2.87	1.19	5	1	2
A8BCZ7	Uncharacterized protein OS=Giardia intestinalis (strain ATCC 50803 / WB c	2.87	2.88	3	1	1
A8BZ07	Ankyrin repeat protein 1 OS=Giardia intestinalis (strain ATCC 50803 / WB	2.85	1.52	3	1	1
A0A644EYK7	Uncharacterized protein OS=Giardia intestinalis (strain ATCC 50803 / WB c	2.85	3.69	2	1	1
C6LVM6	Uncharacterized protein OS=Giardia intestinalis (strain ATCC 50581 / GS c	2.85	5.73	4	1	1
V6TQ09	Uncharacterized protein OS=Giardia intestinalis OX=5741 GN=GSB_15380	2.85	1.98	4	1	1
E1EWN2	Uncharacterized protein (Fragment) OS=Giardia intestinalis (strain P15) O	2.85	2.35	5	1	1
Q966X6	Rab-like protein A (Fragment) OS=Giardia intestinalis OX=5741 GN=GlRab	2.85	6.32	4	1	1
E1F5W5	Uncharacterized protein OS=Giardia intestinalis (strain P15) OX=658858 G	2.84	3.61	2	1	1
A8BHX8	Ankyrin repeat protein 1 OS=Giardia intestinalis (strain ATCC 50803 / WB	2.84	1.61	2	1	1
E1F0J1	ERP1 OS=Giardia intestinalis (strain P15) OX=658858 GN=GLP15_4192 PI	2.84	3.61	2	1	1
E1EWN9	Mu adaptin OS=Giardia intestinalis (strain P15) OX=658858 GN=GLP15_3	2.83	2.23	3	1	1
E1F632	ERCC4 domain-containing protein OS=Giardia intestinalis (strain P15) OX=	2.83	1.81	1	1	1
A8BP11	Uncharacterized protein OS=Giardia intestinalis (strain ATCC 50803 / WB c	2.83	5.18	3	1	1
V6TGP4	Uncharacterized protein OS=Giardia intestinalis OX=5741 GN=DHA2_2445	2.82	15.83	1	1	1
A0A644FAQ9	Uncharacterized protein OS=Giardia intestinalis (strain ATCC 50803 / WB c	2.82	1.63	4	1	1
A8B1R9	Inositol-hexakisphosphate/diphosphoinositol-pentakisphosphate 1-kinase (	2.82	0.87	5	2	2
A8BJ01	Uncharacterized protein OS=Giardia intestinalis (strain ATCC 50803 / WB c	2.82	5.11	2	1	1
C6LV46	ZipA OS=Giardia intestinalis (strain ATCC 50581 / GS clone H7) OX=59874	2.81	1.66	2	1	1
V6TPB0	Ribonuclease P protein subunit p29 OS=Giardia intestinalis OX=5741 GN=	2.81	5.56	1	1	1

A0A644F693	Uncharacterized protein OS=Giardia intestinalis (strain ATCC 50803 / WB c	2.80	3.64	3	1	1
A8BJV9	Kinesin-like protein OS=Giardia intestinalis (strain ATCC 50803 / WB clone	2.80	3.36	3	1	1
A8B660	Putative tRNA (Uracil-5-)-methyltransferase OS=Giardia intestinalis (strain	2.80	1.42	2	1	1
V6TKK5	Uncharacterized protein OS=Giardia intestinalis OX=5741 GN=DHA2_162	2.78	0.44	2	1	1
A8B618	Kinase OS=Giardia intestinalis (strain ATCC 50803 / WB clone C6) OX=18	2.78	1.83	2	1	1
A8B9S6	Uncharacterized protein OS=Giardia intestinalis (strain ATCC 50803 / WB c	2.78	3.06	2	1	1
A0A644F835	TLD domain-containing protein OS=Giardia intestinalis (strain ATCC 5080	2.78	0.98	3	1	1
D3KHG4	TOG domain-containing protein OS=Giardia intestinalis (strain ATCC 5080	2.77	1.18	3	1	1
A8BW25	Uncharacterized protein OS=Giardia intestinalis (strain ATCC 50803 / WB c	2.76	0.84	2	1	1
V6TH09	Uncharacterized protein OS=Giardia intestinalis OX=5741 GN=DHA2_154	2.76	10.48	3	1	1
V6TST6	Uncharacterized protein OS=Giardia intestinalis OX=5741 GN=GSB_15361	2.76	2.15	1	1	1
A8BST8	Uncharacterized protein OS=Giardia intestinalis (strain ATCC 50803 / WB c	2.76	0.91	2	1	1
V6TEJ9	Uncharacterized protein OS=Giardia intestinalis OX=5741 GN=DHA2_151	2.75	0.67	2	1	1
E1F056	Uncharacterized protein (Fragment) OS=Giardia intestinalis (strain P15) O	2.75	2.74	2	1	1
C6LRS2	Spindle pole protein, putative OS=Giardia intestinalis (strain ATCC 50581 /	2.74	2.31	2	1	1
A8B569	Ankyrin repeat protein 1 OS=Giardia intestinalis (strain ATCC 50803 / WB	2.74	1.97	2	1	1
C6LU53	Uncharacterized protein OS=Giardia intestinalis (strain ATCC 50581 / GS c	2.74	3.07	2	1	1
E1F776	ANK_REP_REGION domain-containing protein OS=Giardia intestinalis (stra	2.74	0.82	3	1	1
A8BPS8	Uncharacterized protein OS=Giardia intestinalis (strain ATCC 50803 / WB c	2.73	5.79	3	1	1
C6LYV3	C2 DOCK-type domain-containing protein OS=Giardia intestinalis (strain A	2.73	0.36	4	1	1
A0A644EYZ4	Kinase, NEK OS=Giardia intestinalis (strain ATCC 50803 / WB clone C6) O	2.72	5.85	3	2	2
D3KHL4	Uncharacterized protein OS=Giardia intestinalis (strain ATCC 50803 / WB c	2.72	2.16	2	1	1
E1F9P8	AAA_12 domain-containing protein OS=Giardia intestinalis (strain P15) OX	2.72	2.29	8	1	1
V6U2K0	AAA superfamily protein OS=Giardia intestinalis OX=5741 GN=GSB_4244	2.71	2.67	5	1	1
A0A644F5Z1	Uncharacterized protein OS=Giardia intestinalis (strain ATCC 50803 / WB c	2.71	7.64	1	1	1
A8B8N7	Protein phosphatase 2C OS=Giardia intestinalis (strain ATCC 50803 / WB c	2.71	2.15	2	1	1
A8B7N4	Kinase, NEK OS=Giardia intestinalis (strain ATCC 50803 / WB clone C6) O	2.71	1.19	2	1	1
A8BU06	Uncharacterized protein OS=Giardia intestinalis (strain ATCC 50803 / WB c	2.70	5.04	2	1	1
A8BUG1	Uncharacterized protein OS=Giardia intestinalis (strain ATCC 50803 / WB c	2.68	1.99	2	1	1
V6TEX1	Serine/threonine protein kinase (Fragment) OS=Giardia intestinalis OX=57	2.67	10.91	3	1	1
A8BTX4	1,4-alpha-glucan branching enzyme OS=Giardia intestinalis (strain ATCC 5	2.67	1.40	2	1	1
V6TDN9	F5/8 type C domain containing protein (Fragment) OS=Giardia intestinalis	2.67	10.69	5	1	1
A0A644F664	Putative SnRNP Sm-like protein OS=Giardia intestinalis (strain ATCC 5080	2.65	8.70	4	1	1
A0A644EZM8	Uncharacterized protein OS=Giardia intestinalis (strain ATCC 50803 / WB c	2.65	2.58	5	1	1
A8BAG0	Uncharacterized protein OS=Giardia intestinalis (strain ATCC 50803 / WB c	2.64	1.89	2	1	1
A8B911	RAN binding protein 1 OS=Giardia intestinalis (strain ATCC 50803 / WB cl	2.64	8.43	2	1	1
A8BTJ9	Trichohyalin OS=Giardia intestinalis (strain ATCC 50803 / WB clone C6) O	2.64	0.76	1	1	1
A8BXC1	Uncharacterized protein OS=Giardia intestinalis (strain ATCC 50803 / WB c	2.64	6.00	4	1	1
A8BAI9	Protein-tyrosine-phosphatase OS=Giardia intestinalis (strain ATCC 50803 /	2.63	1.97	3	1	1

A8B6R1	Ankyrin repeat protein 1 OS=Giardia intestinalis (strain ATCC 50803 / WB	2.62	2.62	2	1	1
A8B9H3	Ankyrin repeat protein 1 OS=Giardia intestinalis (strain ATCC 50803 / WB	2.62	1.17	4	1	1
A8B3I9	Uncharacterized protein OS=Giardia intestinalis (strain ATCC 50803 / WB	2.61	6.74	2	1	1
E1EXT3	Uncharacterized protein OS=Giardia intestinalis (strain P15) OX=658858 G	2.61	0.39	1	1	2
A8BY44	Ankyrin repeat protein 1 OS=Giardia intestinalis (strain ATCC 50803 / WB	2.60	1.78	3	1	1
A0A644F3W6	Ankyrin repeat protein 1 OS=Giardia intestinalis (strain ATCC 50803 / WB	2.60	4.89	2	1	1
A0A644F3T9	Uncharacterized protein OS=Giardia intestinalis (strain ATCC 50803 / WB	2.59	10.38	5	1	1
D3KGA2	Uncharacterized protein OS=Giardia intestinalis (strain ATCC 50803 / WB	2.58	1.12	2	1	1
Q4VPQ2	Alpha-16 giardin OS=Giardia intestinalis OX=5741 PE=4 SV=1 - [Q4VPQ2_	2.58	2.74	2	1	1
D3KGF8	Uncharacterized protein OS=Giardia intestinalis (strain ATCC 50803 / WB	2.58	3.31	2	1	1
Q86BT9	DNA topoisomerase 2 OS=Giardia intestinalis OX=5741 GN=GlTOP2 PE=3	2.58	0.94	4	1	1
A8BQ62	Pyridoxamine 5'-phosphate oxidase OS=Giardia intestinalis (strain ATCC 5	2.58	10.69	3	1	1
A8BG96	Uncharacterized protein OS=Giardia intestinalis (strain ATCC 50803 / WB	2.58	4.13	4	1	1
V6T994	rRNA adenine N(6)-methyltransferase OS=Giardia intestinalis OX=5741 GI	2.57	3.80	4	1	1
A8BBA6	Uncharacterized protein OS=Giardia intestinalis (strain ATCC 50803 / WB	2.57	2.86	1	1	1
V6TXB2	Serine/threonine protein kinase OS=Giardia intestinalis OX=5741 GN=GSE	2.57	1.39	1	1	1
D3KHI5	Uncharacterized protein OS=Giardia intestinalis (strain ATCC 50803 / WB	2.56	3.09	3	1	1
A0A644FA23	Uncharacterized protein OS=Giardia intestinalis (strain ATCC 50803 / WB	2.56	0.23	3	1	1
C6LUT3	WD-40 repeat protein OS=Giardia intestinalis (strain ATCC 50581 / GS clo	2.54	1.03	5	1	1
A8BTG5	Rav1p_C domain-containing protein OS=Giardia intestinalis (strain ATCC 5	2.54	0.40	2	1	1
D3KHT7	Uncharacterized protein OS=Giardia intestinalis (strain ATCC 50803 / WB	2.54	4.88	2	1	1
A8BPL1	Uncharacterized protein OS=Giardia intestinalis (strain ATCC 50803 / WB	2.53	1.07	3	1	1
A8BTT1	Heat shock protein 70 OS=Giardia intestinalis (strain ATCC 50803 / WB clo	2.52	1.78	1	1	1
D3KI05	WW domain-containing protein OS=Giardia intestinalis (strain ATCC 50803	2.52	1.76	2	1	1
A8B5R0	Ankyrin repeat protein 1 OS=Giardia intestinalis (strain ATCC 50803 / WB	2.51	2.33	2	2	2
E1F224	CTP synthase OS=Giardia intestinalis (strain P15) OX=658858 GN=GLP15_	2.50	1.78	3	1	1
D3KHE0	Uncharacterized protein OS=Giardia intestinalis (strain ATCC 50803 / WB	2.49	2.51	2	1	1
A8BPQ8	SMC1 beta-like protein OS=Giardia intestinalis (strain ATCC 50803 / WB cl	2.49	0.96	3	1	1
A8BF99	Ubiquitin-like domain-containing protein OS=Giardia intestinalis (strain AT	2.49	12.37	2	1	1
A8BQ31	Annexin OS=Giardia intestinalis (strain ATCC 50803 / WB clone C6) OX=1	2.49	4.00	2	1	1
D3KG82	Actin OS=Giardia intestinalis (strain ATCC 50803 / WB clone C6) OX=184	2.48	2.36	2	1	1
V6TLL2	C2H2-type domain-containing protein OS=Giardia intestinalis OX=5741 GN	2.48	13.33	1	1	1
E1F1X4	Uncharacterized protein OS=Giardia intestinalis (strain P15) OX=658858 G	2.47	1.41	3	1	1
A8BFQ9	Uncharacterized protein OS=Giardia intestinalis (strain ATCC 50803 / WB	2.47	3.39	3	1	1
V6TR55	Uncharacterized protein OS=Giardia intestinalis OX=5741 GN=GSB_15214	2.47	1.67	4	1	1
V6TC91	Uncharacterized protein OS=Giardia intestinalis OX=5741 GN=DHA2_154	2.47	3.35	1	1	1
A8BPQ1	Tetratricopeptide repeat-containing protein OS=Giardia intestinalis (strain	2.46	4.44	2	1	1
A8BHG7	Uncharacterized protein OS=Giardia intestinalis (strain ATCC 50803 / WB	2.46	4.27	4	1	1
E1EXK4	Ubiquitin-conjugating enzyme E2-17 kDa 3 OS=Giardia intestinalis (strain	2.46	11.41	5	2	2

V6TEV0	Putative Dpy-30 motif protein OS=Giardia intestinalis OX=5741 GN=DHA2	2.46	13.64	2	1	1
E1F028	Fructose-bisphosphate aldolase OS=Giardia intestinalis (strain P15) OX=6	2.46	2.48	4	1	1
A0A644F6D9	Separase OS=Giardia intestinalis (strain ATCC 50803 / WB clone C6) OX=	2.46	0.80	3	1	1
V6TNA4	Uncharacterized protein OS=Giardia intestinalis OX=5741 GN=DHA2_151	2.45	18.97	2	1	1
V6U864	Uncharacterized protein OS=Giardia intestinalis OX=5741 GN=GSB_1509	2.44	1.88	4	1	1
V6TF97	Putative calcium-binding family protein OS=Giardia intestinalis OX=5741	2.43	6.01	1	1	1
E1F0S3	Uncharacterized protein OS=Giardia intestinalis (strain P15) OX=658858	2.42	1.57	5	1	1
A8BYT4	Uncharacterized protein OS=Giardia intestinalis (strain ATCC 50803 / WB	2.42	4.89	1	1	1
A8BB38	Uncharacterized protein OS=Giardia intestinalis (strain ATCC 50803 / WB	2.41	1.27	3	1	1
Q9NCP0	DNA repair and recombination protein RadB (Fragment) OS=Giardia intest	2.39	5.93	6	1	1
A8B3S7	Uncharacterized protein OS=Giardia intestinalis (strain ATCC 50803 / WB	2.39	3.54	3	1	1
A8BNT9	3 exoribonuclease, putative OS=Giardia intestinalis (strain ATCC 50803 /	2.38	4.21	2	1	1
A8B3N5	Ankyrin repeat protein 1 OS=Giardia intestinalis (strain ATCC 50803 / WB	2.38	1.12	3	1	1
V6TUX2	ANK_REP_REGION domain-containing protein (Fragment) OS=Giardia inte	2.37	3.82	1	1	1
A8BUV6	Zinc finger domain protein OS=Giardia intestinalis (strain ATCC 50803 / W	2.37	1.71	2	1	1
A8BI15	ATP adenylyltransferase OS=Giardia intestinalis (strain ATCC 50803 / WB	2.35	3.16	1	1	1
A8BBX9	Ankyrin repeat protein 1 OS=Giardia intestinalis (strain ATCC 50803 / WB	2.35	1.37	2	1	1
A8B8Z9	Trafficking protein particle complex subunit OS=Giardia intestinalis (strain	2.34	4.82	4	1	1
A8BDA2	Tubulin specific chaperone D OS=Giardia intestinalis (strain ATCC 50803 /	2.34	0.70	2	1	1
A0A644F413	Uncharacterized protein OS=Giardia intestinalis (strain ATCC 50803 / WB	2.34	3.49	4	1	1
A8BJI8	Uncharacterized protein OS=Giardia intestinalis (strain ATCC 50803 / WB	2.33	2.87	3	1	1
D3KH45	Uncharacterized protein OS=Giardia intestinalis (strain ATCC 50803 / WB	2.33	1.24	1	1	1
A8BBV5	Guanylate kinase OS=Giardia intestinalis (strain ATCC 50803 / WB clone C	2.32	6.34	1	1	1
V6TXQ4	Ankyrin repeat protein OS=Giardia intestinalis OX=5741 GN=GSB_17568	2.32	2.39	5	1	1
A8BAV5	Uncharacterized protein OS=Giardia intestinalis (strain ATCC 50803 / WB	2.32	3.27	2	1	1
A8BAI0	Uncharacterized protein OS=Giardia intestinalis (strain ATCC 50803 / WB	2.31	0.18	1	1	1
A0A644F7D5	Uncharacterized protein OS=Giardia intestinalis (strain ATCC 50803 / WB	2.30	5.06	4	2	2
A8B6T8	Uncharacterized protein OS=Giardia intestinalis (strain ATCC 50803 / WB	2.30	0.84	2	1	1
E1F5V3	WW domain-containing protein OS=Giardia intestinalis (strain P15) OX=65	2.29	1.31	4	1	1
A8B2R7	Kinase, STE STE20 OS=Giardia intestinalis (strain ATCC 50803 / WB clone	2.28	2.47	4	1	1
E1EXG2	Uncharacterized protein OS=Giardia intestinalis (strain P15) OX=658858	2.28	6.91	4	2	2
A8BXS8	Kinase, CMGC RCK OS=Giardia intestinalis (strain ATCC 50803 / WB clone	2.28	3.02	3	1	1
A8BD31	Uncharacterized protein OS=Giardia intestinalis (strain ATCC 50803 / WB	2.27	0.91	5	1	1
A0A644FAV0	ATP-dependent RNA helicase OS=Giardia intestinalis (strain ATCC 50803 /	2.27	1.19	4	1	1
A8B7N0	Putative RNA binding protein OS=Giardia intestinalis (strain ATCC 50803 /	2.26	4.40	2	1	1
A8B519	Uncharacterized protein OS=Giardia intestinalis (strain ATCC 50803 / WB	2.26	1.04	3	1	1
A0A644F0M5	Ribonuclease P OS=Giardia intestinalis (strain ATCC 50803 / WB clone C6)	2.25	14.05	2	1	1
V6U0N8	UDENN domain-containing protein OS=Giardia intestinalis OX=5741 GN=	2.25	1.12	3	1	1
C6LU67	ABC transporter family protein OS=Giardia intestinalis (strain ATCC 50581	2.24	1.17	4	1	1

C6LWH2	Kinase, NEK OS=Giardia intestinalis (strain ATCC 50581 / GS clone H7) OX	2.24	2.22	5	1	1
A8B8V3	Transcription factor RRN3 OS=Giardia intestinalis (strain ATCC 50803 / WI	2.24	2.16	2	1	1
A0A644F2P3	Uncharacterized protein OS=Giardia intestinalis (strain ATCC 50803 / WB	2.24	0.57	3	1	1
A8BPX4	tRNA (guanine(26)-N(2))-dimethyltransferase OS=Giardia intestinalis (stra	2.23	1.88	1	1	1
D3KG56	LisH domain-containing protein OS=Giardia intestinalis (strain ATCC 5080	2.23	2.62	2	1	1
A0A644F0E8	Cyclin B-like protein OS=Giardia intestinalis (strain ATCC 50803 / WB clon	2.22	1.50	4	1	1
V6TYZ4	Pumilio family RNA binding domain protein OS=Giardia intestinalis OX=57	2.22	1.80	4	1	1
A8B5S2	Phosphatidylinositol-4-phosphate 5-kinase, putative OS=Giardia intestinali	2.22	2.82	4	1	1
C6LPX3	Uncharacterized protein OS=Giardia intestinalis (strain ATCC 50581 / GS c	2.21	0.86	2	1	1
E1F5N3	Uncharacterized protein OS=Giardia intestinalis (strain P15) OX=658858 G	2.21	2.76	3	1	1
A8BBC8	Polyadenylation specificity factor family protein OS=Giardia intestinalis (st	2.20	0.62	2	1	1
A8BRW5	Uncharacterized protein OS=Giardia intestinalis (strain ATCC 50803 / WB	2.20	24.44	3	1	1
A8B657	Putative Transcriptional repressor NOT4Hp OS=Giardia intestinalis (strain	2.19	2.92	2	1	1
E1EYS6	Programmed cell death protein-like protein OS=Giardia intestinalis (strain	2.18	4.44	3	1	1
A0A644F280	Mucin-like protein OS=Giardia intestinalis (strain ATCC 50803 / WB clone	2.18	1.50	1	1	1
A8B6T1	RNA recognition motif-containing protein OS=Giardia intestinalis (strain AT	2.18	5.70	2	1	1
A0A644FB72	MCT-1 protein-like protein OS=Giardia intestinalis (strain ATCC 50803 / W	2.17	8.10	2	1	1
V6TZV9	Uncharacterized protein OS=Giardia intestinalis OX=5741 GN=GSB_13164	2.16	6.80	1	1	1
D3KGH6	Uncharacterized protein OS=Giardia intestinalis (strain ATCC 50803 / WB	2.16	0.87	2	1	1
E1F2S6	Nucleoside diphosphate kinase OS=Giardia intestinalis (strain P15) OX=65	2.16	5.96	3	1	1
A8BE55	Coiled-coil protein OS=Giardia intestinalis (strain ATCC 50803 / WB clone	2.15	0.39	6	1	1
A8BNN3	Ankyrin repeat protein 1 OS=Giardia intestinalis (strain ATCC 50803 / WB	2.14	1.77	2	2	2
A0A644F4T4	Uncharacterized protein OS=Giardia intestinalis (strain ATCC 50803 / WB	2.14	0.24	2	1	1
A8BPU4	Uncharacterized protein OS=Giardia intestinalis (strain ATCC 50803 / WB	2.14	1.20	2	1	1
A8BIV5	Nuclear ATP/GTP-binding protein OS=Giardia intestinalis (strain ATCC 508	2.13	3.52	1	1	1
A8BNW6	Chromosome segregation protein Spc25 OS=Giardia intestinalis (strain AT	2.13	4.17	4	1	1
C6LQ78	Phospholipid-transporting ATPase IIB, putative OS=Giardia intestinalis (str	2.13	1.33	2	1	1
A0A644EYQ9	Uncharacterized protein OS=Giardia intestinalis (strain ATCC 50803 / WB	2.13	4.66	3	1	1
A8BHX1	Uncharacterized protein OS=Giardia intestinalis (strain ATCC 50803 / WB	2.10	4.29	2	1	1
E1F611	ATP-dependent DNA helicase OS=Giardia intestinalis (strain P15) OX=658	2.10	1.69	5	1	1
A0A644EYU0	Uncharacterized protein OS=Giardia intestinalis (strain ATCC 50803 / WB	2.10	2.40	4	1	1
A8BI18	WD-40 repeat protein OS=Giardia intestinalis (strain ATCC 50803 / WB clo	2.09	1.79	4	1	1
A8BAA0	Uncharacterized protein OS=Giardia intestinalis (strain ATCC 50803 / WB	2.08	2.06	2	1	1
A8B3T5	Coiled-coil protein OS=Giardia intestinalis (strain ATCC 50803 / WB clone	2.08	1.19	3	1	1
V6TX38	Putative SP-RING zinc finger protein OS=Giardia intestinalis OX=5741 GN	2.06	1.56	4	1	1
V6TXF0	Uncharacterized protein OS=Giardia intestinalis OX=5741 GN=GSB_15423	2.05	0.70	2	1	1
V6TRP2	TPR_REGION domain-containing protein OS=Giardia intestinalis OX=5741	2.01	2.97	2	1	1
A8B374	Phosphorylase B kinase gamma catalytic chain OS=Giardia intestinalis (str	2.01	1.10	3	2	2
E1F6J8	WD40 protein OS=Giardia intestinalis (strain P15) OX=658858 GN=GLP15	2.00	3.02	4	1	1

E1EVR6	Kinase, NEK OS=Giardia intestinalis (strain P15) OX=658858 GN=GLP15_	2.00	1.85	5	1	1
A8BM93	Uncharacterized protein OS=Giardia intestinalis (strain ATCC 50803 / WB	2.00	3.69	4	1	1
E1F569	Protein 21.1 OS=Giardia intestinalis (strain P15) OX=658858 GN=GLP15_	1.98	0.94	3	1	1
E1F816	UBCE14 OS=Giardia intestinalis (strain P15) OX=658858 GN=GLP15_448	1.98	6.71	1	1	1
A8BDQ6	Heat shock 70kD protein binding protein OS=Giardia intestinalis (strain AT	1.98	3.07	4	1	1
V6T9J2	Uncharacterized protein OS=Giardia intestinalis OX=5741 GN=DHA2_841	1.97	3.72	1	1	1
A8BER7	Uncharacterized protein OS=Giardia intestinalis (strain ATCC 50803 / WB	1.92	1.92	5	1	1
V6TJ18	Ankyrin repeat protein OS=Giardia intestinalis OX=5741 GN=DHA2_1523	1.91	3.40	5	1	1
Q9NI39	20S proteasome alpha subunit 2 (Fragment) OS=Giardia intestinalis OX=5	1.91	5.83	4	1	1
Q969C2	Kinesin-like protein (Fragment) OS=Giardia intestinalis OX=5741 GN=GlK	1.91	2.25	5	2	2
A8B204	Ankyrin repeat protein 1 OS=Giardia intestinalis (strain ATCC 50803 / WB	1.89	1.04	1	1	1
A8B4A9	Ubiquitin-conjugating enzyme E2-17 kDa OS=Giardia intestinalis (strain A	1.89	6.92	4	1	1
A8B740	Coiled-coil protein OS=Giardia intestinalis (strain ATCC 50803 / WB clone	1.87	1.51	2	1	1
Q966X0	Rac-like protein A (Fragment) OS=Giardia intestinalis OX=5741 GN=GlRac	1.87	4.26	3	1	1
Q6WD94	Rad26 OS=Giardia intestinalis OX=5741 PE=4 SV=2 - [Q6WD94_GIAIN]	1.86	0.97	3	1	1
C6LNA5	Uncharacterized protein OS=Giardia intestinalis (strain ATCC 50581 / GS	1.85	1.34	5	1	1
A8B4P7	Putative U6 snRNA-associated Sm-like protein LSm3 OS=Giardia intestinal	1.84	7.14	5	1	1
A8BQ89	Thioredoxin-like domain-containing protein OS=Giardia intestinalis (strain	1.82	6.25	3	1	1
A8BGW7	Phosphatase OS=Giardia intestinalis (strain ATCC 50803 / WB clone C6)	1.79	1.82	5	1	1
A8BP59	Serine/threonine-protein phosphatase 2A activator OS=Giardia intestinalis	1.77	5.84	7	2	2
A8W232	Cyclin N-terminal domain-containing protein OS=Giardia intestinalis OX=5	1.77	4.22	2	1	1
A0A644EY12	Superoxide reductase (Desulfoferredoxin) OS=Giardia intestinalis (strain A	1.77	5.41	6	1	1
E1F3A1	Kinase, CMGC CDK OS=Giardia intestinalis (strain P15) OX=658858 GN=G	1.77	2.56	3	1	1
D3KG00	Uncharacterized protein OS=Giardia intestinalis (strain ATCC 50803 / WB	1.76	5.75	3	2	2
A8BNZ7	Sec61-gamma OS=Giardia intestinalis (strain ATCC 50803 / WB clone C6)	1.76	10.81	2	1	1
A0A644F0U4	Putative Small nuclear ribonucleoprotein Sm D2 OS=Giardia intestinalis (st	1.76	8.43	3	1	1
E1F460	UBC2, putative OS=Giardia intestinalis (strain P15) OX=658858 GN=GLP1	1.75	4.65	2	1	1
A8BM26	Uncharacterized protein OS=Giardia intestinalis (strain ATCC 50803 / WB	1.75	0.66	2	1	1
A8BS32	Uncharacterized protein OS=Giardia intestinalis (strain ATCC 50803 / WB	1.74	3.70	5	1	1
V6TUZ4	Uncharacterized protein OS=Giardia intestinalis OX=5741 GN=GSB_1521	1.74	0.94	1	1	1
E1F4J8	Uncharacterized protein OS=Giardia intestinalis (strain P15) OX=658858	1.74	3.74	3	1	1
A8B3E3	Uncharacterized protein OS=Giardia intestinalis (strain ATCC 50803 / WB	1.73	3.80	4	1	1
A8BFN4	UDP-N-acetylglucosamine--peptide N-acetylglucosaminyltransferase OS=G	1.71	0.74	2	1	1
C6LU99	Uncharacterized protein OS=Giardia intestinalis (strain ATCC 50581 / GS	1.69	4.70	5	1	2
E1F9G6	Protein 21.1 OS=Giardia intestinalis (strain P15) OX=658858 GN=GLP15_	1.67	1.38	1	1	1
C6LXJ9	Replication factor C, subunit 1 OS=Giardia intestinalis (strain ATCC 50581	1.66	1.02	5	1	1
A0A644F576	Uncharacterized protein OS=Giardia intestinalis (strain ATCC 50803 / WB	1.64	3.89	5	1	1
E1F1G9	Lipase_3 domain-containing protein OS=Giardia intestinalis (strain P15) O	1.62	1.04	4	1	1
A0A644EYZ1	Uncharacterized protein OS=Giardia intestinalis (strain ATCC 50803 / WB	1.62	0.97	4	1	1

A8B663	Uncharacterized protein OS=Giardia intestinalis (strain ATCC 50803 / WB [illegible]	1.61	5.66	5	1	1
V6TK86	Uncharacterized protein OS=Giardia intestinalis OX=5741 GN=DHA2_151[illegible]	0.00	0.69	1	1	1
Q86LL4	Leucine-rich repeat protein 1 OS=Giardia intestinalis OX=5741 GN=LRP1 [illegible]	0.00	1.87	3	1	1
V6U524	Uncharacterized protein OS=Giardia intestinalis OX=5741 GN=GSB_1508[illegible]	0.00	0.36	3	1	1
V6U104	Tyrosine-protein kinase SRK3 OS=Giardia intestinalis OX=5741 GN=GSB_[illegible]	0.00	2.24	1	1	1
V6TYE9	Uncharacterized protein OS=Giardia intestinalis OX=5741 GN=GSB_1510[illegible]	0.00	0.70	1	1	1
E1F1F4	CHY-type domain-containing protein OS=Giardia intestinalis (strain P15) [illegible]	0.00	15.00	2	1	1
E1F5P5	Protein 21.1 OS=Giardia intestinalis (strain P15) OX=658858 GN=GLP15_[illegible]	0.00	1.35	3	1	1
E1F2Z7	RNA polymerase II subunit Rpb5a OS=Giardia intestinalis (strain P15) OX[illegible]	0.00	3.06	3	1	1
E1F2I5	Uncharacterized protein OS=Giardia intestinalis (strain P15) OX=658858 [illegible]	0.00	1.67	4	1	1
A8B3L0	Uncharacterized protein OS=Giardia intestinalis (strain ATCC 50803 / WB [illegible]	0.00	1.41	2	1	1
A8B798	Hydroxyacylglutathione hydrolase OS=Giardia intestinalis (strain ATCC 50[illegible]	0.00	5.36	2	1	1
A8B755	CGI-35 protein, putative OS=Giardia intestinalis (strain ATCC 50803 / WB [illegible]	0.00	3.16	5	1	1
A8BM24	Prefoldin subunit 2 OS=Giardia intestinalis (strain ATCC 50803 / WB clone[illegible]	0.00	7.41	3	1	1
A0A644F6Q2	Uncharacterized protein OS=Giardia intestinalis (strain ATCC 50803 / WB [illegible]	0.00	2.33	2	1	1
A8BP52	Uncharacterized protein OS=Giardia intestinalis (strain ATCC 50803 / WB [illegible]	0.00	1.40	3	1	1
D3KHZ8	Uncharacterized protein OS=Giardia intestinalis (strain ATCC 50803 / WB [illegible]	0.00	0.66	1	1	1
D3KHZ6	Uncharacterized protein OS=Giardia intestinalis (strain ATCC 50803 / WB [illegible]	0.00	4.81	1	1	1
A8BAZ6	Uncharacterized protein OS=Giardia intestinalis (strain ATCC 50803 / WB [illegible]	0.00	2.30	2	1	1
A8BSM8	Uncharacterized protein OS=Giardia intestinalis (strain ATCC 50803 / WB [illegible]	0.00	1.24	2	1	1
A8BUF6	Uncharacterized protein OS=Giardia intestinalis (strain ATCC 50803 / WB [illegible]	0.00	1.28	2	1	1
C6LT88	Seven transmembrane protein 1 OS=Giardia intestinalis (strain ATCC 505[illegible]	0.00	2.05	5	1	1
C6LN12	Uncharacterized protein OS=Giardia intestinalis (strain ATCC 50581 / GS [illegible]	0.00	0.12	2	1	1
A8BX96	Sin-like protein OS=Giardia intestinalis (strain ATCC 50803 / WB clone C6[illegible]	0.00	1.76	3	1	1
A8BKA3	Protein-serine/threonine phosphatase OS=Giardia intestinalis (strain ATC[illegible]	0.00	2.15	2	1	1
A8B716	Ubiquitin-conjugating enzyme E2 OS=Giardia intestinalis (strain ATCC 508[illegible]	0.00	2.16	3	1	1
A8B1Y9	BUB3 OS=Giardia intestinalis (strain ATCC 50803 / WB clone C6) OX=184[illegible]	0.00	2.20	4	1	1
D3KH62	Uncharacterized protein OS=Giardia intestinalis (strain ATCC 50803 / WB [illegible]	0.00	1.69	2	1	1
A8BDU6	Ankyrin repeat protein 1 OS=Giardia intestinalis (strain ATCC 50803 / WB [illegible]	0.00	2.80	1	1	1
A8BCH0	Uncharacterized protein OS=Giardia intestinalis (strain ATCC 50803 / WB [illegible]	0.00	2.71	5	1	1
A8BN53	Uncharacterized protein OS=Giardia intestinalis (strain ATCC 50803 / WB [illegible]	0.00	28.95	1	1	1
A8BWD9	Multidrug resistance-associated protein Mrp2 OS=Giardia intestinalis (stra[illegible]	0.00	3.55	6	1	1
A0A644F0F6	Uncharacterized protein OS=Giardia intestinalis (strain ATCC 50803 / WB [illegible]	0.00	1.31	6	1	1
E1EZ66	Kinase, NEK OS=Giardia intestinalis (strain P15) OX=658858 GN=GLP15_[illegible]	0.00	2.40	1	1	1

# PSMs	Area	# AAs	MW [kDa]	calc. pI
474	3.034E10	379	42.6	10.07
338	1.944E10	664	71.6	5.39
276	9.244E9	1627	179.2	4.50
275	9.244E9	1627	179.2	4.50
248	3.271E10	316	35.0	10.52
225	2.596E10	122	11.9	4.59
237	1.986E10	732	79.5	8.12
214	2.080E10	268	30.4	9.99
174	1.840E10	326	35.0	5.64
206	2.922E10	244	27.9	5.00
201	2.797E10	248	28.3	9.94
202	2.528E10	189	21.6	10.65
169	1.082E10	154	17.5	10.35
188	1.656E10	189	21.7	10.46
156	2.100E10	174	19.4	10.32
181	2.720E10	242	26.7	9.70
173	1.570E10	141	16.0	10.17
138	2.787E10	217	24.6	9.79
139	2.412E9	1904	213.6	8.16
142	1.672E10	217	24.7	9.25
139	1.401E10	151	17.3	9.09
142	1.243E10	196	23.2	11.08
127	2.070E10	182	19.6	9.04
122	4.449E9	895	99.3	6.55
115	1.029E10	198	21.9	8.41
89	6.753E9	89	9.7	8.51
106	6.338E9	1641	180.8	4.50
103	1.226E10	190	21.0	9.85
101	6.464E9	143	15.9	11.44
84	1.886E10	131	14.8	10.10
112	1.179E10	223	25.1	9.94
130	2.751E10	235	27.0	9.73
79	1.411E10	159	18.2	10.87
118	2.275E9	1904	213.7	8.27
104	2.310E10	139	15.4	10.36
105	1.957E10	190	21.7	9.61
94	1.610E9	1731	195.7	6.49
104	5.101E9	123	13.9	10.04

95	1.193E10	232	26.8	10.20
120	1.253E10	172	20.4	9.85
76	4.843E9	106	12.2	9.79
96	3.421E9	1151	127.6	4.88
103	1.635E10	248	28.0	10.10
99	1.472E10	204	24.3	11.49
94	2.799E9	142	15.4	10.54
126	6.343E9	297	33.9	9.91
80	7.131E9	137	15.7	10.30
113	1.028E10	210	24.2	10.17
82	2.709E10	135	15.3	9.50
68	1.225E10	124	14.4	6.57
82	1.308E10	326	35.0	6.09
72	1.042E10	197	22.9	9.63
81	3.561E9	676	77.6	9.13
31	9.411E9	118	11.8	4.06
68	2.077E10	158	17.6	10.51
66	1.550E10	206	23.4	11.49
76	3.958E9	888	97.1	6.77
77	2.462E10	248	28.3	9.91
64	2.312E8	5550	622.6	5.67
73	4.663E9	704	80.7	5.11
63	4.704E9	125	14.3	8.18
63	2.139E8	5412	600.7	6.28
60	6.555E9	251	27.0	11.06
80	9.225E9	267	30.4	10.02
67	1.351E9	1731	195.8	6.55
60	5.960E8	5573	629.2	6.32
52	1.449E10	185	20.8	9.39
58	2.983E8	4877	553.1	5.60
54	3.308E9	135	15.6	10.76
56	2.983E8	4879	553.3	5.62
55	1.771E9	1199	131.7	6.86
67	1.238E10	130	14.7	10.21
53	6.475E8	3226	362.4	4.78
68	6.228E9	442	50.0	9.04
68	6.228E9	442	49.9	9.04
51	4.207E9	248	28.6	5.17
51	4.429E8	1620	182.4	4.94

55	2.642E9	391	43.2	5.80
54	9.341E9	136	15.9	11.05
47	3.495E8	2675	301.9	5.78
51	3.285E8	2270	258.8	6.01
53	9.101E9	145	16.7	11.69
47	1.388E9	737	83.6	9.23
59	7.197E9	139	15.4	10.29
48	2.502E8	2565	291.8	5.55
48	5.960E8	5577	629.8	6.30
56	1.308E10	326	35.1	6.42
45	1.133E8	4835	539.4	5.26
51	1.305E10	134	15.4	8.53
40	9.337E8	870	96.3	5.60
46	1.972E8	5117	570.0	5.45
46	2.954E8	2406	273.9	5.41
39	6.518E8	652	71.7	6.39
38	8.813E8	8035	889.1	4.75
42	2.177E9	746	82.2	6.81
54	1.006E10	179	20.1	10.48
43	2.720E9	447	50.0	4.87
45	2.369E8	5163	577.9	5.35
47	9.093E9	164	18.8	10.24
46	1.911E9	758	84.5	7.28
61	1.604E9	126	14.0	8.91
40	1.995E10	145	15.7	10.18
39	1.750E9	1199	131.8	6.92
40	2.242E9	786	87.6	5.81
29	6.273E9	88	10.1	10.11
38	1.133E8	4838	540.4	5.27
37	1.316E9	848	94.7	6.83
31	3.794E9	246	26.8	5.55
36	1.413E9	765	87.4	6.51
39	1.759E10	69	7.9	11.31
37	1.044E8	6458	707.3	6.51
44	5.573E9	179	20.1	10.42
27	4.738E8	1595	175.4	4.65
35	9.354E8	1454	163.1	6.42
39	3.376E9	308	35.5	5.45
35	8.916E8	1453	163.1	6.47

29	2.914E8	402	45.2	6.47
50	1.033E10	173	19.8	9.67
37	1.558E9	454	50.0	6.99
34	3.349E9	439	48.9	5.58
33	1.371E8	4773	522.7	6.35
29	6.131E8	189	22.7	10.45
35	1.507E9	301	33.1	5.16
29	2.758E8	1086	121.2	6.61
33	1.288E9	664	73.7	6.07
35	5.893E8	770	85.2	5.71
35	1.561E9	581	65.8	6.92
38	6.970E7	5572	633.0	6.42
27	8.572E8	81	9.1	8.37
28	6.226E8	430	48.4	8.18
38	7.956E8	902	99.0	6.35
24	3.056E9	94	10.5	10.54
33	7.963E8	124	14.1	9.17
30	4.588E8	1960	215.3	6.01
28	3.213E8	1680	187.0	5.92
28	2.987E9	120	13.5	11.00
27	1.019E9	990	103.1	6.58
29	1.019E9	662	72.6	5.10
28	3.115E8	1675	190.4	5.02
34	1.343E9	442	49.1	8.40
40	2.819E9	375	41.8	6.07
29	2.415E8	2832	315.2	6.47
26	8.403E8	697	76.1	5.41
25	2.639E9	241	27.2	8.13
31	6.736E8	1253	138.8	6.93
32	8.567E9	64	7.3	10.71
27	2.257E8	1006	111.4	6.06
25	1.964E9	225	25.3	6.99
30	1.241E9	403	46.1	9.06
29	3.966E8	1339	146.5	6.05
25	1.230E9	291	32.8	8.90
42	1.038E9	1904	214.5	7.99
30	1.600E8	3417	383.5	7.15
23	5.998E7	1832	205.1	4.77
28	1.976E8	2911	322.6	6.70

37	7.139E9	154	17.6	10.56
25	4.513E8	1062	115.7	8.34
28	5.434E8	541	60.2	8.95
23	4.161E8	480	53.4	6.74
26	1.477E8	1697	189.9	9.14
32	2.405E10	124	14.3	10.74
28	7.386E9	132	14.8	10.11
21	8.499E8	886	97.3	6.90
29	2.169E10	149	17.1	11.00
26	6.155E8	782	86.0	9.11
21	1.907E8	1194	130.3	6.98
21	1.119E9	338	38.4	7.02
23	2.564E8	1851	201.3	6.73
26	2.268E8	6739	751.5	6.89
38	7.264E9	102	12.0	11.37
21	3.033E8	1871	206.8	5.44
25	2.437E9	409	45.1	7.69
20	9.046E8	191	21.2	7.43
19	1.404E9	515	57.0	5.07
24	1.612E9	307	35.0	6.10
22	5.133E8	434	48.4	7.49
21	3.112E8	1386	153.1	6.90
22	1.730E8	1945	213.3	5.91
19	4.779E8	547	60.6	8.35
22	1.496E8	2645	300.3	5.66
24	6.331E8	937	104.0	6.54
24	7.452E8	285	31.4	8.65
22	6.330E8	468	51.4	7.37
20	9.548E8	308	32.4	6.25
23	7.530E8	621	70.2	6.93
27	6.930E8	1084	122.9	8.82
20	1.334E9	330	36.6	5.26
17	5.143E8	542	62.4	5.00
21	1.098E8	1039	115.9	4.72
19	3.055E8	1330	149.7	6.92
18	2.372E8	1929	212.2	5.91
18	6.825E8	332	37.7	8.65
21	3.373E8	1277	139.2	7.14
22	4.408E8	884	97.6	6.73

22	2.393E8	1066	116.4	6.33
18	5.483E8	595	64.7	7.55
23	1.521E8	2381	262.3	5.45
24	5.581E8	564	61.5	6.70
23	1.583E9	383	42.4	9.42
24	6.204E8	178	18.9	8.40
16	5.310E8	502	54.2	7.01
19	9.784E8	445	48.2	5.66
23	1.037E8	2981	334.8	6.15
22	2.521E9	429	47.0	7.83
20	1.629E8	3080	340.9	6.52
20	3.943E8	821	93.1	6.87
18	7.544E7	2711	298.9	5.45
21	1.924E8	1712	187.7	6.58
18	1.271E8	2703	298.8	6.57
22	5.783E8	497	54.7	6.55
17	1.961E8	1107	122.5	5.88
19	1.243E8	5631	631.9	5.88
16	5.450E8	563	60.6	5.21
17	4.323E8	654	70.7	8.46
18	5.418E8	510	56.4	7.64
20	3.637E8	871	98.4	5.45
17	1.400E8	1504	164.0	6.55
18	1.496E8	1709	187.9	6.62
19	7.506E8	502	55.3	7.83
18	5.189E8	936	103.3	6.90
20	4.271E8	1132	122.8	5.44
22	5.850E8	506	54.6	5.48
21	2.184E9	314	35.4	7.88
16	5.835E8	528	56.8	5.47
17	1.578E8	819	89.8	5.94
17	3.759E8	650	71.5	8.70
20	1.751E9	345	39.3	5.87
17	2.105E8	960	105.2	5.54
19	1.134E9	454	49.7	9.06
17	5.555E8	494	55.6	6.42
17	6.995E7	1424	159.5	7.03
18	2.302E9	338	38.3	5.64
19	4.726E8	809	88.2	7.61

15	1.260E9	483	52.6	8.59
15	6.423E8	493	55.1	6.43
16	6.284E8	530	59.4	6.60
16	6.172E7	2180	239.3	6.40
15	8.879E8	352	38.9	6.37
20	6.481E8	584	64.4	6.76
17	5.037E8	553	61.2	5.83
14	6.062E7	1514	169.2	6.49
16	2.967E8	1304	147.4	6.05
17	3.033E8	865	95.6	5.71
19	5.524E8	656	73.3	7.99
16	4.056E8	542	61.0	7.12
16	3.961E7	6320	716.0	7.44
15	1.009E9	305	35.4	6.33
16	6.741E8	755	84.8	7.08
15	8.219E7	1514	166.1	5.69
13	2.116E8	1026	112.7	9.48
15	1.425E8	1874	212.1	7.20
19	2.889E8	983	110.5	7.03
15	7.350E8	360	41.2	6.40
19	5.979E8	559	60.9	5.77
14	1.590E9	185	20.8	4.53
15	3.556E8	480	53.1	7.24
13	2.268E8	569	63.3	5.92
14	2.746E8	622	69.3	6.35
14	4.417E8	275	31.5	9.88
14	3.751E8	432	49.2	6.54
17	6.056E8	647	72.6	7.02
14	6.730E7	960	106.2	6.29
14	2.846E8	1330	149.9	6.87
12	5.615E7	2467	275.6	6.89
14	5.400E8	354	38.8	6.92
12	2.146E7	4034	447.6	7.59
12	2.683E8	588	64.2	6.89
14	7.146E7	1399	153.1	7.42
15	6.891E7	2538	282.1	6.09
17	5.955E8	401	45.0	8.24
13	3.493E8	672	75.5	7.05
14	1.228E9	467	53.9	7.03

12	4.384E8	483	52.6	5.06
14	3.254E8	514	55.4	6.67
15	1.255E8	1822	200.7	5.94
13	5.232E7	1190	132.9	4.93
14	4.832E7	2343	259.5	5.48
14	9.637E7	1293	145.8	7.81
13	3.810E8	804	90.1	7.71
14	1.071E8	925	104.4	5.99
15	1.663E8	1234	136.4	6.52
13	1.266E9	295	33.8	6.55
15	1.434E8	1661	181.7	6.52
12	2.010E8	1414	157.2	6.43
15	1.171E8	1999	224.2	6.07
15	2.447E8	994	109.2	6.55
13	3.101E8	621	69.8	6.84
19	4.143E8	905	99.4	6.24
13	5.421E7	2363	262.5	7.20
11	2.723E8	447	50.2	7.71
13	1.110E8	3227	363.0	6.37
14	5.086E8	613	69.0	9.19
14	7.667E7	3044	346.9	6.37
13	3.997E7	7449	834.2	6.62
18	1.734E9	260	30.1	10.55
12	2.086E8	926	103.5	6.74
14	2.845E9	529	60.1	5.73
15	3.119E8	803	87.1	7.08
10	2.607E8	429	47.9	7.33
17	4.674E8	491	55.3	7.09
16	1.115E8	2222	248.6	6.89
13	2.351E8	230	26.1	5.35
13	1.088E8	2079	232.5	6.67
15	2.622E8	781	86.4	8.72
16	3.206E8	793	89.5	6.24
14	8.981E7	1670	182.5	7.34
13	1.135E8	2745	305.3	6.73
12	4.169E8	472	51.8	5.58
11	2.235E8	680	74.5	6.14
14	7.106E8	339	38.3	6.54
12	2.036E8	225	25.7	5.52

10	2.722E8	1079	115.0	7.11
13	2.500E8	233	26.5	5.16
14	3.683E7	1906	213.9	7.71
13	3.495E8	390	43.3	5.54
13	2.548E8	667	74.3	9.03
15	9.368E7	1263	144.8	7.01
14	2.390E8	673	74.9	5.17
12	8.640E8	216	23.6	8.54
12	2.781E8	781	87.0	6.23
15	1.574E8	1107	124.0	6.43
12	8.919E7	2632	291.9	5.20
11	6.759E8	251	27.6	4.81
12	2.454E8	691	76.1	6.21
15	1.862E8	923	104.7	5.15
11	1.043E9	203	22.5	5.25
14	2.109E8	517	56.3	6.61
12	6.368E7	1710	190.9	6.58
12	1.018E9	90	10.6	10.61
12	5.213E7	2109	234.1	6.29
12	5.069E8	444	49.6	5.43
10	1.545E8	995	109.2	5.63
9	1.915E8	378	42.0	7.83
13	1.603E8	618	69.6	6.27
11	3.753E8	900	100.1	9.35
12	5.365E7	1842	204.0	6.58
13	7.408E8	642	69.6	7.77
12	8.308E7	4207	467.2	6.55
13	2.042E8	696	77.8	5.08
11	5.814E8	414	46.1	6.16
12	8.227E8	262	28.6	7.02
11	5.034E8	449	50.4	8.31
10	4.816E7	686	76.8	7.56
13	3.838E7	2421	269.1	6.51
14	9.785E8	201	22.6	6.89
13	1.040E9	375	41.6	5.31
10	3.570E8	612	67.1	5.82
11	2.089E8	823	90.5	6.68
11	7.396E7	702	80.1	7.20
11	2.224E8	807	90.3	6.09

11	1.921E8	1038	116.6	6.47
12	8.471E7	883	99.3	6.61
13	9.824E7	860	97.2	5.34
11	3.323E7	3147	352.3	6.65
13	7.383E7	629	69.9	6.35
9	1.403E8	567	62.7	5.58
12	3.832E7	4360	488.8	7.49
10	2.878E8	523	58.8	5.40
12	1.593E8	1075	115.8	6.98
11	5.816E7	2245	248.6	7.25
12	1.612E8	467	52.5	4.35
12	2.695E8	1003	109.1	5.36
11	1.043E9	203	22.5	5.02
12	2.096E8	1525	172.9	7.68
11	4.928E8	1024	109.7	5.72
10	4.860E8	235	26.9	7.85
12	5.499E9	77	8.7	9.57
9	1.416E8	706	78.0	4.74
11	2.484E8	831	93.2	6.42
10	1.860E8	1399	155.8	6.44
10	8.702E8	262	28.9	6.93
10	5.173E7	857	100.5	5.40
12	4.723E7	1646	181.3	6.77
10	2.498E8	580	64.1	6.54
14	6.741E7	1249	140.6	6.49
11	2.267E8	1026	114.5	5.52
14	1.224E8	1063	118.8	6.96
10	7.682E7	1863	209.8	5.85
10	5.598E8	614	67.3	8.13
10	5.240E7	1300	145.5	7.65
10	3.906E7	1059	119.1	7.53
13	3.697E8	501	55.9	5.45
11	8.948E7	1568	174.0	6.55
9	2.961E8	308	35.2	6.96
13	4.105E8	407	46.2	5.72
11	3.820E7	908	102.8	7.12
11	4.972E8	376	42.4	8.53
11	3.935E8	327	35.1	9.85
9	6.245E7	1058	120.0	6.49

9	2.169E8	615	69.1	8.44
9	5.029E7	1666	186.0	6.05
11	3.814E8	447	50.5	5.22
12	9.347E7	1462	161.7	6.16
10	3.578E8	498	55.2	4.96
10	7.350E7	1068	120.0	7.75
11	4.577E8	553	61.0	5.76
12	2.952E8	498	55.4	9.25
10	1.890E8	477	53.6	4.77
9	2.095E8	607	67.6	7.90
7	9.757E7	654	73.8	6.07
11	1.566E8	870	95.8	6.10
11	6.800E7	794	86.8	6.28
10	6.217E7	952	106.9	8.41
9	2.465E8	1069	118.4	6.54
13	2.488E8	863	98.0	7.06
9	2.182E8	498	55.6	5.03
8	3.988E8	777	85.1	4.54
11	2.293E8	455	49.8	5.67
10	7.642E7	829	94.0	6.77
8	1.326E8	247	27.7	7.18
10	1.529E8	596	67.4	9.00
11	1.772E8	1419	154.8	6.44
9	1.355E8	509	54.9	5.86
11	6.560E7	1293	144.9	8.03
10	1.993E8	950	107.8	5.38
13	2.534E8	1111	123.0	5.08
10	3.970E8	387	43.1	6.02
13	1.677E8	1048	114.8	6.70
9	4.501E8	350	39.5	6.71
10	5.253E7	1361	151.8	6.28
10	2.012E8	330	37.1	6.57
10	5.144E8	413	45.8	8.37
17	4.389E8	819	93.0	5.97
8	1.362E8	932	104.0	4.65
10	2.036E8	170	19.2	5.25
8	1.068E8	785	83.9	5.83
8	6.136E7	1172	130.5	5.99
11	1.843E8	1144	124.3	7.50

9	7.717E8	385	42.7	9.66
12	5.206E8	295	33.0	7.27
11	2.834E8	783	87.0	5.31
9	2.199E8	757	83.8	6.29
9	1.365E8	270	30.0	5.34
8	3.520E8	853	94.3	7.87
10	2.759E8	410	46.9	5.20
9	2.640E8	330	37.7	6.65
9	2.561E8	623	66.2	6.77
8	9.944E7	682	76.5	5.00
10	2.671E8	550	59.2	6.24
9	6.254E7	739	80.1	5.02
12	4.015E8	512	56.9	7.65
9	3.017E7	1853	203.4	7.64
9	8.243E7	939	102.5	5.05
9	1.661E8	472	52.5	6.61
9	1.375E8	768	83.2	5.26
9	1.339E8	481	53.5	6.05
8	1.455E8	839	92.2	5.73
8	3.000E7	3070	338.7	6.54
9	2.322E8	505	55.1	6.61
9	9.074E7	1528	167.9	6.47
8	2.932E8	256	29.4	5.53
8	7.394E7	1576	174.2	5.60
8	1.699E8	322	34.8	5.71
8	5.548E7	1524	171.9	5.60
9	2.012E8	755	83.6	5.52
8	1.038E8	837	93.2	7.11
7	1.652E8	494	54.4	6.38
8	1.548E8	638	70.1	5.11
8	1.263E8	556	61.1	6.81
10	1.442E8	1135	126.6	6.23
10	2.661E8	1232	135.8	6.76
8	1.044E8	476	52.4	5.02
10	1.628E8	1029	110.7	5.11
9	1.001E8	334	38.1	6.25
11	1.855E10	109	11.7	9.45
10	2.562E7	2282	259.5	6.35
10	2.142E8	875	98.4	5.91

9	1.993E8	950	107.9	5.45
9	5.541E7	716	79.0	5.08
9	1.815E8	509	57.2	6.30
11	1.583E8	346	40.3	5.97
9	2.440E8	268	29.5	7.81
9	4.724E7	2287	252.8	5.54
7	4.185E7	586	65.9	8.66
8	6.730E7	714	79.6	6.81
8	3.200E8	432	47.1	5.22
10	2.614E8	527	59.0	6.62
8	6.325E7	1928	215.5	6.98
8	1.736E8	474	52.1	6.93
10	6.099E7	1143	126.8	5.40
8	8.764E7	641	71.6	5.87
9	4.016E7	2041	228.2	5.59
8	1.611E8	322	34.9	5.71
8	8.278E7	764	85.5	5.45
9	9.535E7	670	73.7	7.11
8	5.617E8	492	51.9	8.03
8	6.498E7	1310	146.6	5.05
12	4.732E8	310	35.3	5.14
7	2.676E8	643	71.2	6.89
9	7.788E7	1041	115.2	7.11
8	3.162E8	279	32.4	9.74
9	4.239E7	892	101.1	7.74
9	2.455E8	192	21.3	5.59
7	9.973E7	724	78.7	6.46
10	8.258E7	914	103.7	6.76
7	6.602E7	582	65.4	6.89
8	4.001E7	2032	227.2	6.76
8	3.596E7	1638	183.2	6.46
8	2.068E8	560	62.3	5.55
9	7.825E7	1030	112.6	8.03
8	5.811E7	465	51.3	7.20
8	2.362E7	1181	129.7	5.21
8	4.755E7	1431	157.7	6.76
11	8.472E7	1133	126.2	6.49
9	2.636E8	459	50.5	4.92
12	1.081E8	892	101.1	5.69

11	2.512E8	479	53.1	5.24
9	6.088E7	1276	144.8	6.34
9	1.829E8	734	80.8	4.98
9	7.041E7	1080	124.0	7.62
9	4.206E8	296	33.9	6.54
5	2.570E8	286	31.3	5.44
8	9.442E7	321	36.0	5.86
9	1.196E8	545	60.4	5.73
8	1.071E8	971	105.8	7.17
8	2.080E8	237	27.4	9.47
9	9.082E7	664	74.7	5.55
9	7.487E7	403	45.3	9.60
7	6.290E7	961	107.4	8.16
8	6.682E7	989	109.2	7.34
8	3.955E8	303	34.6	6.64
9	1.243E8	1010	114.9	6.54
8	1.078E7	3175	354.4	6.28
7	2.426E7	947	105.3	5.58
8	2.893E8	608	68.8	8.10
9	7.456E7	313	35.9	5.06
8	8.344E7	1042	114.3	5.34
7	5.858E7	513	56.6	6.42
9	1.606E8	1055	118.9	6.10
7	9.313E7	655	73.9	5.53
9	1.136E8	767	85.4	8.16
9	2.403E8	51	6.1	9.39
8	4.288E7	2142	238.6	7.33
6	5.952E7	909	99.9	7.46
9	7.664E7	596	66.0	8.57
8	1.492E8	656	72.7	8.43
7	8.432E7	1076	120.1	6.07
7	3.192E8	337	38.2	5.16
7	1.495E8	406	44.7	5.66
9	2.514E8	482	55.0	7.62
7	2.526E7	839	92.6	6.54
7	3.608E8	336	36.3	7.18
8	1.043E8	749	85.4	5.78
8	3.626E7	768	86.2	7.97
10	1.958E8	627	70.0	5.81

7	2.820E7	1801	199.1	6.57
7	3.890E8	452	48.3	6.43
8	9.324E7	742	83.0	7.47
6	3.275E7	734	79.6	6.38
7	3.475E8	180	20.3	9.19
4	9.875E7	669	73.0	9.32
8	1.615E8	1095	120.4	5.77
8	4.779E7	634	70.5	8.29
9	3.338E8	401	44.2	6.99
8	9.535E7	642	72.6	6.13
7	7.067E7	287	32.6	6.60
6	1.382E8	621	68.1	5.82
7	6.583E7	639	71.3	6.81
8	8.937E7	1081	120.2	6.93
6	1.682E7	792	87.8	6.29
8	6.308E7	582	66.0	6.89
7	2.043E7	337	36.8	8.53
6	5.717E7	741	82.0	9.01
7	1.275E8	375	40.9	4.82
8	1.361E8	1025	115.1	8.18
7	9.159E7	1094	122.4	5.29
8	8.926E7	601	68.2	8.12
8	2.332E7	1435	160.5	6.06
8	2.036E8	138	15.9	5.19
6	1.585E8	315	34.4	5.11
7	3.559E8	187	20.9	8.59
7	1.923E8	387	41.4	5.14
6	2.590E7	3377	376.2	6.64
7	5.222E7	283	32.4	9.54
7	6.821E8	206	22.9	5.10
8	1.499E8	1187	131.0	5.06
7	1.192E8	1419	154.8	6.44
8	2.330E7	1317	147.7	6.92
7	1.201E8	656	72.5	8.29
7	7.266E7	804	88.3	4.89
8	4.564E8	408	46.4	5.91
7	4.404E7	385	43.9	5.91
7	1.177E8	551	62.0	7.14
7	9.049E7	527	59.1	6.58

7	6.378E7	1097	124.2	8.69
8	9.566E7	1873	212.3	7.15
7	2.100E7	2345	258.0	7.20
7	6.931E7	396	44.1	6.37
8	1.297E8	988	108.7	4.77
7	1.291E8	523	58.8	6.80
8	1.652E8	479	53.1	8.31
8	4.038E7	1847	202.1	6.47
6	1.827E7	473	52.0	6.32
7	1.004E8	669	73.1	7.31
7	1.990E8	255	28.2	5.49
8	4.974E8	375	40.6	6.52
5	9.903E7	321	36.9	5.69
8	1.509E8	298	33.2	5.30
8	3.426E7	648	72.5	6.24
8	7.052E7	1110	125.2	6.38
8	2.596E8	593	65.9	6.43
5	2.340E8	354	39.6	6.47
5	1.143E8	516	56.0	5.97
7	8.036E7	509	55.9	8.28
6	1.948E7	910	100.1	6.62
6	4.583E7	1307	144.3	6.47
6	7.237E7	568	63.8	7.61
6	1.284E8	297	33.9	6.44
6	1.809E8	423	47.0	7.28
7	4.336E7	666	72.9	6.32
6	7.256E7	719	77.5	6.81
9	5.230E7	895	99.3	5.69
6	6.533E7	513	56.1	6.92
7	5.177E7	1609	177.8	5.11
5	2.600E7	2462	270.7	6.20
7	2.883E7	1299	145.4	7.33
5	1.493E7	1736	193.5	7.71
7	1.833E8	1044	113.9	6.25
5	1.253E7	1618	179.8	6.11
7	3.789E7	2088	233.9	7.01
6	1.084E8	584	64.5	7.25
6	3.849E7	750	82.0	7.05
6	1.565E7	1075	119.9	6.11

5	2.348E7	1084	119.3	7.69
6	6.631E7	484	53.6	6.25
8	1.022E8	674	75.0	6.38
7	2.502E7	1172	131.4	5.52
6	1.164E8	369	38.9	5.30
5	4.935E7	508	56.3	5.06
7	4.529E7	663	74.2	5.08
7	4.292E7	945	105.1	6.42
6	4.199E7	946	103.3	5.66
5	1.291E8	293	31.6	5.43
7	2.706E7	1906	208.3	6.74
6	3.323E7	610	66.4	6.10
7	5.586E7	960	106.3	6.57
8	8.148E7	1481	165.1	6.09
7	3.006E8	981	108.7	5.74
6	1.263E8	360	39.1	7.68
6	3.578E8	671	74.1	6.06
5	5.122E7	1497	163.1	6.79
6	5.509E7	733	81.7	5.33
6	3.842E7	674	75.9	6.54
6	1.520E8	198	22.6	6.57
7	1.814E8	554	62.2	5.97
7	6.597E7	1118	123.4	9.29
8	8.499E7	678	77.1	8.53
5	1.120E8	497	54.4	5.68
6	1.759E7	3609	404.0	7.40
6	4.926E7	1242	138.0	6.01
5	9.829E6	810	90.4	6.64
8	1.005E8	461	50.5	8.37
6	3.636E7	1448	158.0	5.66
6	2.429E7	1528	167.1	5.06
5	2.784E7	1268	140.5	6.37
7	5.654E7	789	87.3	5.36
5	9.247E7	707	78.4	8.18
5	4.469E7	834	92.4	6.29
6	1.172E8	350	39.9	5.19
7	1.172E8	485	54.6	5.78
6	1.007E8	562	62.7	9.11
5	3.303E7	593	66.0	6.35

5	1.013E8	302	32.9	6.46
6	1.515E8	341	39.5	5.53
5	1.006E8	1680	187.8	6.00
6	1.234E7	678	76.8	5.41
5	9.279E7	542	60.3	8.92
7	2.592E8	483	53.5	6.55
6	6.240E7	237	27.0	8.40
5	2.204E7	369	42.1	4.94
6	7.367E7	754	84.0	7.01
7	1.093E8	1044	116.9	6.92
7	2.399E7	1859	210.2	7.40
5	1.519E8	530	60.7	8.88
5	1.053E8	170	19.2	4.64
5	1.447E8	332	37.5	6.46
7	3.762E7	870	95.8	7.02
6	7.749E7	351	39.6	7.69
5	7.358E7	354	39.5	5.03
5	4.188E7	287	31.1	5.68
6	6.909E8	420	46.5	7.31
5	2.777E7	374	42.9	4.36
5	5.283E7	954	106.7	6.64
6	6.602E7	555	62.1	6.43
6	5.844E8	163	17.9	5.17
6	3.969E7	1728	191.9	5.48
6	4.415E7	504	57.6	6.48
4	9.633E7	597	64.7	5.55
7	1.149E8	530	58.6	8.32
5	6.341E7	2096	231.7	6.49
7	7.170E7	924	104.8	6.87
8	4.981E7	350	39.9	8.63
4	1.578E8	467	51.4	6.47
7	7.337E8	220	24.7	5.00
5	2.785E7	609	70.1	5.74
7	1.946E8	316	33.9	7.85
6	1.000E8	407	46.6	7.09
5	9.950E7	552	62.6	5.54
7	1.379E8	440	49.5	5.72
6	7.027E7	619	68.5	6.62
7	2.619E8	401	46.5	8.91

5	2.011E8	589	65.3	7.87
5	1.061E8	206	23.2	5.39
5	1.851E7	2251	250.3	6.93
4	8.206E7	321	34.7	7.18
5	1.464E7	971	107.6	5.69
5	2.901E7	1270	143.6	7.44
7	8.224E8	995	110.1	5.62
5	6.093E8	192	21.2	9.22
6	3.483E7	654	71.8	6.20
6	1.336E7	2037	226.6	6.76
5	1.310E8	669	73.1	9.32
5	9.505E7	583	64.1	7.88
6	3.122E8	185	21.4	5.82
5	8.872E7	352	39.6	9.45
5	4.150E7	353	40.5	5.64
6	7.284E7	468	53.8	7.62
4	1.191E8	153	17.3	4.44
5	2.246E7	414	46.6	5.66
4	7.570E7	343	37.6	6.19
6	1.924E7	808	86.7	6.55
4	6.439E7	304	32.1	5.25
6	1.420E8	238	27.2	6.15
4	6.104E7	177	19.8	6.27
5	4.017E7	717	78.5	6.27
5	1.849E7	2076	230.2	6.87
4	1.148E8	193	21.0	7.99
5	7.332E7	758	85.2	6.10
4	3.118E7	450	49.2	8.60
5	7.708E7	187	20.4	6.11
6	8.948E7	223	24.4	6.13
4	4.305E7	1471	162.0	5.43
5	1.870E7	2421	273.4	7.50
6	5.821E7	871	95.2	6.71
4	6.207E7	294	32.5	5.03
6	8.694E7	562	62.5	9.69
5	2.877E7	1110	121.8	7.55
5	1.575E7	473	53.7	4.78
6	2.320E8	192	21.8	5.03
4	1.158E7	1025	111.9	6.67

6	4.225E7	366	43.1	9.66
5	5.138E7	247	27.3	5.12
5	6.238E7	840	97.6	4.56
6	5.663E7	715	76.6	7.61
5	2.007E8	827	89.9	6.61
5	1.762E7	1956	221.0	7.46
6	1.157E8	449	50.0	8.32
7	4.728E7	1130	123.2	6.06
5	1.891E7	910	100.1	6.70
5	8.709E7	684	76.9	7.09
5	8.069E7	422	46.3	6.42
6	1.991E7	2015	228.8	8.87
4	1.433E7	826	94.0	5.55
4	1.220E7	587	65.3	5.59
5	3.007E7	2479	273.8	6.06
5	7.315E7	1102	121.3	4.78
5	3.386E7	466	52.2	5.57
4	4.016E7	6939	776.4	7.09
4	8.475E7	209	23.7	6.54
5	7.336E7	647	74.0	6.92
5	1.081E8	144	16.8	9.99
6	3.588E8	327	36.4	7.02
5	3.006E8	207	23.4	7.23
6	5.089E7	458	50.3	6.44
5	3.690E7	2413	266.4	6.77
4	5.666E7	675	74.3	6.35
7	6.370E8	151	16.9	6.79
4	2.124E7	1113	123.4	5.80
3	6.745E8	176	20.1	9.60
5	8.343E7	570	64.2	6.80
6	1.741E8	815	88.9	4.27
5	1.575E8	469	53.0	7.72
5	3.154E7	349	38.8	6.73
4	3.114E7	346	35.8	6.93
5	3.179E7	1121	125.0	4.98
5	3.796E7	303	34.5	5.68
6	5.382E7	328	36.5	6.81
5	1.256E8	457	51.1	5.33
4	1.218E8	467	52.0	5.29

5	1.036E7	596	64.9	6.74
4	3.233E7	1367	153.5	7.09
4	3.089E7	554	62.1	8.35
5	1.958E7	730	82.6	8.21
5	8.856E7	346	39.2	5.99
6	1.630E8	429	48.2	7.81
5	1.118E8	189	21.4	5.71
5	4.232E7	480	52.0	6.58
5	3.660E7	1691	187.9	6.24
5	2.082E7	771	84.7	6.32
4	6.608E7	831	91.9	5.68
5	4.673E8	188	22.0	7.42
4	1.213E8	338	37.6	5.30
6	3.201E8	386	43.1	8.19
4	1.310E7	1016	111.6	5.52
4	2.972E7	1826	202.2	5.03
6	3.169E7	579	64.5	6.80
4	8.554E7	431	48.6	5.03
6	5.309E8	340	38.1	6.87
5	5.840E7	933	102.6	5.36
6	3.363E7	576	65.9	6.51
4	6.613E7	775	84.7	6.23
4	2.179E7	571	64.9	6.13
5	1.707E7	681	75.8	5.66
5	8.807E7	579	64.5	9.45
4	1.879E7	965	108.1	5.16
5	3.851E7	745	81.6	5.78
3	8.397E7	322	35.8	6.74
4	3.797E7	970	107.9	5.87
6	3.123E7	1198	136.0	6.90
5	5.159E7	646	71.1	6.13
4	1.407E8	343	37.7	6.09
6	1.990E8	255	28.2	5.49
4	1.116E8	214	23.2	6.64
5	1.302E7	2730	305.2	5.58
5	1.565E7	552	61.1	7.40
4	8.158E7	772	85.2	9.28
5	4.724E7	179	20.4	5.57
5	1.744E7	553	61.8	6.62

4	1.072E7	1507	167.7	7.18
3	4.075E7	314	35.0	4.84
4	2.541E7	574	61.9	8.12
4	4.137E7	824	91.0	6.43
4	4.870E6	76	8.6	8.50
3	4.121E7	292	32.4	5.05
4	1.315E8	546	60.8	4.75
4	2.422E7	1367	154.0	6.76
4	6.252E7	619	69.2	5.43
4	1.753E8	159	18.6	9.91
7	4.910E7	1760	195.0	5.48
3	1.791E7	1626	178.9	6.65
5	3.339E7	639	69.5	6.25
5	1.764E7	2057	229.0	7.65
3	1.587E7	403	44.5	5.58
4	5.877E7	391	42.8	6.32
5	4.068E7	310	34.3	6.89
5	6.892E7	156	17.0	6.30
3	3.648E7	166	18.8	4.60
4	1.579E7	1111	118.8	7.75
5	2.442E7	665	74.9	6.55
3	8.432E7	223	24.5	5.10
3	7.925E7	297	33.3	7.23
3	9.596E6	974	107.1	7.59
3	1.664E7	605	67.2	7.84
4	3.006E8	196	22.0	4.77
4	1.492E7	6973	780.2	6.65
4	3.243E7	1500	165.0	6.92
4	7.539E7	845	96.2	8.43
4	2.933E7	201	23.1	9.60
4	1.178E7	834	93.9	5.47
3	3.146E7	226	25.4	5.72
3	2.144E8	95	10.3	8.50
4	3.651E7	239	26.3	9.33
4	2.904E7	322	35.6	7.18
3	2.497E7	571	62.7	6.40
3	9.556E7	575	63.9	5.66
3	5.371E7	925	102.6	5.36
4	8.933E7	230	25.8	9.07

3	2.167E8	297	32.8	5.87
5	4.033E7	722	80.3	7.25
4	5.910E7	335	36.5	6.47
3	1.605E8	851	95.3	6.10
6	3.698E7	1208	133.1	6.92
5	2.511E8	141	16.8	6.87
5	5.259E7	496	55.4	7.37
5	8.655E7	679	76.8	5.73
3	1.718E8	406	45.6	5.97
2	1.076E8	349	38.1	8.44
3	2.222E7	832	91.8	5.10
4	7.174E7	877	99.8	5.45
5	1.183E8	770	85.1	5.92
4	1.942E7	1091	118.6	6.73
3	5.944E7	109	12.8	10.51
3	6.482E7	211	23.2	7.01
2	1.646E7	435	46.7	7.74
5	9.393E6	2066	229.7	6.21
4	9.413E7	824	92.8	7.06
3	2.482E7	459	51.6	5.96
3	2.916E7	107	11.9	6.52
3	2.768E7	270	30.1	6.27
3	1.865E7	348	39.2	8.48
5	1.082E8	170	19.5	9.74
4	3.373E8	153	17.1	6.55
3	3.595E7	496	54.8	8.24
4	4.745E7	225	25.7	5.30
5	4.425E7	493	55.6	6.84
3	1.496E7	381	42.1	5.22
2	1.418E7	406	45.5	7.12
4	6.035E7	968	106.4	6.21
4	8.185E7	418	47.3	6.54
3	8.174E7	107	12.0	5.87
4	5.976E7	84	9.5	8.16
3	3.894E7	373	41.6	8.27
4	2.210E7	876	96.7	8.28
3	6.331E6	1277	139.9	7.64
3	2.835E7	193	21.7	9.00
4	1.752E7	1106	123.0	8.00

4	3.864E7	1632	179.2	6.25
3	6.675E7	106	12.1	6.58
3	5.400E7	311	35.6	6.98
5	4.043E8	89	10.4	7.50
4	7.525E7	460	50.8	5.49
3	1.414E8	208	22.9	8.13
4	2.407E7	1544	174.8	6.51
3	8.582E7	428	46.7	4.84
3	4.103E7	861	95.1	5.55
3	1.662E7	369	40.7	6.29
4	6.225E8	105	12.5	4.86
5	5.547E7	189	21.7	6.80
3	1.555E7	1209	133.8	5.87
3	7.446E6	1920	213.9	5.12
3	1.053E8	329	36.5	4.73
4	1.772E7	354	37.8	5.14
2	3.010E8	245	26.7	6.43
3	1.118E8	226	25.8	5.36
3	1.821E7	753	84.9	6.61
3	5.272E7	440	48.6	5.30
3	4.120E7	383	42.7	5.80
4	9.813E6	352	40.1	7.09
4	8.294E7	568	62.6	6.11
3	1.151E8	419	44.5	6.06
3	8.207E7	368	39.6	4.92
3	2.290E7	1231	136.4	5.43
3	6.191E7	518	59.1	5.66
3	9.563E7	239	27.3	5.50
3	2.565E7	699	78.6	7.09
3	1.687E8	259	29.7	6.09
4	5.079E7	840	96.0	4.59
3	2.108E7	875	98.6	5.50
4	4.110E7	1231	137.5	6.55
3	3.525E7	950	105.4	5.22
4	3.493E8	223	24.8	5.00
4	4.350E7	200	22.8	8.68
3	1.561E8	332	36.7	7.27
3	3.904E8	245	27.8	5.82
4	2.725E8	553	60.5	7.17

4	1.527E7	819	90.2	5.88
4	5.046E7	1305	145.7	5.06
4	1.652E8	168	19.3	5.67
3	1.339E7	7100	796.4	7.31
5	1.593E8	483	53.5	6.80
5	2.153E7	634	70.5	8.07
3	3.964E7	513	57.4	6.80
3	2.336E7	936	103.3	5.69
3	7.817E7	214	23.8	5.73
4	4.629E7	469	52.0	8.22
3	3.173E7	319	36.9	8.34
3	8.536E7	329	37.2	6.57
3	1.566E8	1025	111.4	8.00
3	1.510E8	248	27.9	9.00
3	3.125E7	1158	131.3	6.55
3	4.216E7	100	11.3	5.50
4	4.051E7	1693	188.2	6.49
3	3.058E7	1030	114.5	7.21
5	6.840E7	616	68.9	9.00
4	1.776E8	368	42.3	5.16
5	2.049E8	270	30.9	9.10
3	2.164E7	714	80.2	5.95
4	4.558E7	469	53.3	5.52
3	5.489E7	276	30.6	5.52
5	3.216E7	255	29.9	5.55
4	1.226E8	227	24.6	5.87
3	2.701E7	245	26.6	5.27
5	5.043E8	89	10.3	11.08
3	3.983E7	621	69.8	6.44
3	7.017E7	261	30.4	5.38
3	4.849E7	438	47.8	5.74
4	5.372E7	300	33.0	6.57
2	6.154E7	316	34.7	6.92
3	1.633E7	860	92.7	6.47
3	2.047E7	613	67.4	8.24
3	1.050E8	412	46.4	5.31
3	4.107E7	1198	132.6	6.23
4	2.458E7	685	78.2	7.12
3	1.121E8	341	37.2	5.94

3	1.070E7	748	83.9	8.78
4	8.340E7	826	89.9	6.95
3	2.655E7	772	82.1	8.41
3	1.733E7	345	38.6	8.72
3	4.825E7	489	53.7	6.00
2	3.299E7	417	44.6	5.59
3	1.889E7	263	29.7	5.47
4	9.941E7	825	90.5	7.15
2	1.591E7	711	80.5	6.23
3	8.020E6	1844	206.9	6.34
4	1.660E8	370	40.6	5.63
3	1.807E7	384	43.1	8.27
2	4.507E7	336	37.9	6.46
3	7.904E7	1272	140.3	5.22
3	9.590E7	268	30.7	5.88
3	3.635E7	781	87.5	9.06
3	1.910E7	425	46.6	6.90
3	4.710E7	715	77.4	5.40
2	3.158E7	625	70.8	9.70
4	1.436E8	805	88.7	5.31
3	2.708E7	468	53.1	5.10
4	8.312E7	316	33.3	5.97
4	2.228E7	767	84.1	6.24
2	3.040E7	470	53.4	4.86
3	2.182E7	683	74.3	5.17
2	1.067E7	878	97.9	5.60
2	6.822E7	317	33.7	6.89
3	3.098E7	191	21.8	8.47
4	2.201E8	177	19.4	6.38
3	3.320E7	377	42.0	6.10
3	1.428E7	1173	132.3	5.59
3	2.309E7	831	93.2	7.68
4	1.017E7	653	71.3	6.87
5	5.318E7	703	78.9	6.16
3	1.045E7	984	110.6	6.43
3	1.961E7	963	107.3	6.70
2	1.820E7	224	24.6	7.05
3	2.657E7	1165	133.6	7.14
4	5.063E7	679	75.4	8.57

2	3.744E7	574	63.4	5.22
3	3.857E7	464	51.6	5.90
3	3.614E7	252	28.3	5.12
3	1.442E8	322	34.2	6.33
3	1.463E7	620	68.1	5.72
2		702	77.7	6.76
3	9.053E6	750	84.2	5.22
3	1.247E8	429	47.1	6.13
3	9.155E6	1670	186.2	6.77
4	2.674E8	294	31.4	5.78
3	2.555E8	211	24.5	5.47
3	6.233E7	310	34.6	7.47
3	2.664E8	350	38.4	6.68
2	1.774E7	773	86.6	6.06
3	8.039E6	3123	347.7	7.39
3	2.760E7	359	40.5	5.50
4	8.414E7	181	20.7	5.20
3	9.091E6	973	107.0	8.32
3	5.622E6	2406	267.1	6.28
3	6.170E6	2253	249.5	6.13
2	1.597E7	1003	111.1	8.51
3	1.260E7	201	23.7	6.60
3	3.835E7	324	36.3	6.83
3	1.627E8	300	33.0	5.29
3	3.014E7	511	58.0	6.10
3	6.029E7	385	44.3	5.48
3	2.603E7	393	43.5	4.82
3	4.775E7	409	43.6	7.31
2	1.876E7	1026	114.6	6.27
3	1.826E8	215	23.4	8.50
3	9.508E6	1346	146.1	6.40
2	6.174E7	449	50.4	5.90
3	3.161E8	190	21.3	7.36
3	9.529E6	971	109.7	8.53
3	9.491E7	192	22.1	8.15
2	9.159E6	437	47.6	9.36
3	1.674E7	450	48.6	5.71
2	1.193E7	804	89.4	6.43
3	2.735E7	480	52.5	5.20

2	2.943E7	386	42.8	6.28
3	4.370E7	178	19.5	9.06
3	1.824E7	1985	222.8	6.57
3	1.346E7	2430	266.3	5.97
3	9.526E7	423	46.8	6.98
2	3.842E7	408	46.7	6.61
3	3.970E7	131	14.4	4.65
2	1.566E7	210	24.1	5.72
2	1.188E7	300	33.8	6.34
2	8.342E7	267	29.5	5.27
3	1.178E7	1661	186.4	7.21
3	1.142E7	1602	178.8	6.27
3	4.116E7	453	51.7	5.74
3	2.616E7	551	60.2	6.37
3	2.557E7	729	80.0	6.65
4	1.189E7	864	97.4	5.85
3	3.553E7	438	50.1	6.68
3	1.458E7	1236	135.8	8.35
2	1.513E7	331	37.2	6.89
4	7.013E7	432	48.9	8.05
3	4.074E7	1174	127.3	7.03
2	1.761E8	62	6.8	4.59
2	1.208E7	164	18.7	6.98
2	3.833E7	268	29.3	5.36
3	4.454E8	821	94.0	6.01
2	1.501E7	1146	124.3	6.25
2	5.784E6	769	87.3	5.71
3	1.218E7	468	54.2	6.13
2	1.125E8	244	26.7	5.91
3	1.512E7	895	97.9	7.72
3	2.941E7	3180	356.3	8.02
3	1.709E7	314	34.7	7.59
3	2.542E7	380	42.1	7.23
3	7.863E6	3836	423.3	6.98
3	8.000E6	735	82.1	6.87
3	2.546E7	2653	295.6	7.71
2	5.180E7	122	13.4	8.98
3	6.322E7	256	28.7	6.34
3	9.227E7	105	12.1	9.47

3	2.306E7	1096	121.8	5.77
3	1.555E7	2294	257.0	6.40
3	2.705E7	1287	142.5	7.49
3	2.305E7	1730	194.6	7.40
3	1.329E7	787	86.6	6.29
3	4.969E7	581	64.6	7.50
2	5.859E7	373	41.3	8.09
2	9.134E7	405	45.2	8.10
5	4.592E7	729	80.9	5.96
2	7.002E7	676	75.9	6.62
3	5.166E7	469	53.3	6.01
3	1.372E8	168	19.9	6.28
3	1.492E8	133	14.9	6.15
2	1.136E7	545	60.9	6.44
2	5.647E7	1029	115.9	6.62
2	2.924E7	290	32.4	8.44
3	1.745E8	213	24.7	5.07
2	1.636E7	387	43.4	6.04
2	3.120E7	198	22.6	5.66
2	4.838E7	220	24.5	5.20
2	1.353E7	1058	118.2	6.16
2	9.524E7	1138	124.1	6.14
2	2.199E7	806	88.4	6.54
2	1.551E7	1131	125.5	5.53
2	1.295E7	536	61.3	5.99
3	1.213E7	961	105.2	6.43
2	9.368E7	124	14.4	7.78
3	5.303E7	568	62.2	5.35
4	1.002E8	558	63.1	4.79
4	4.509E8	268	30.1	7.39
2	3.915E6	443	48.0	8.59
2	2.302E7	1216	134.8	5.05
2	5.285E6	686	76.8	6.60
3	5.188E7	882	100.2	8.90
3	4.965E7	767	83.8	6.13
3	8.575E7	305	34.5	9.63
2		819	92.9	4.88
2	8.998E6	597	66.0	8.15
2	5.072E7	332	37.4	5.49

3	6.206E7	557	61.9	6.09
3	9.492E7	365	40.6	5.34
2	5.146E7	184	20.2	7.05
3	1.480E7	763	87.5	7.03
2	2.912E7	1498	169.1	5.39
3	1.269E7	1640	182.3	7.97
2	8.355E7	159	17.4	5.36
2	1.550E7	753	83.0	7.58
2	8.506E6	320	33.5	5.27
3	5.846E7	1167	128.4	6.10
3	6.324E7	2155	242.1	7.39
4	1.183E7	2607	289.2	6.20
2	1.934E7	484	53.0	6.43
2	2.570E7	496	55.4	9.09
2	1.444E7	523	58.0	5.88
2	6.032E7	161	18.0	5.35
2	1.052E7	934	103.9	5.20
2	2.377E7	218	25.5	5.83
3	1.310E8	384	42.2	9.10
2	1.438E7	542	60.2	6.27
4	3.482E7	1261	141.5	4.93
3	3.675E7	214	24.3	5.33
2	2.277E7	700	78.5	5.48
3	1.066E7	679	77.0	6.49
3	1.281E7	326	36.3	5.12
2	3.388E7	424	46.5	6.83
2	1.034E7	253	27.4	7.39
3	1.066E7	555	61.4	6.74
3	3.365E7	335	37.0	8.00
2	1.454E7	1412	155.0	7.08
3	2.489E7	1049	114.1	8.37
4	2.922E7	853	93.6	6.28
2	5.370E7	184	20.8	5.68
2	9.056E7	567	60.8	6.19
3	8.789E7	179	19.9	8.24
2	5.456E7	495	55.3	9.25
2	9.872E6	742	83.3	7.28
2	1.271E7	311	34.5	6.14
3	4.427E7	597	66.9	8.06

3	3.552E8	625	68.2	5.06
2	5.452E6	1090	121.8	6.95
3	1.731E7	719	73.8	5.52
2	1.992E7	841	93.9	6.74
2	4.357E7	331	36.8	4.96
2	3.962E7	1331	146.1	5.91
2	1.407E7	554	61.2	7.50
2	2.668E7	198	22.8	6.60
2	2.197E7	1616	176.8	6.96
3	2.564E7	740	81.4	5.82
2	5.573E7	335	37.9	6.14
3	1.861E7	414	46.4	7.03
2	8.211E6	813	91.7	5.41
2	6.658E7	304	33.8	8.82
2	8.684E6	442	47.2	8.13
2	1.959E7	238	27.5	8.94
3	2.383E7	1379	155.4	6.51
3	1.345E8	367	41.9	8.37
3	9.850E7	122	13.3	9.14
2	1.468E8	128	14.7	9.58
3	1.586E7	541	62.4	5.48
2	2.256E7	1230	135.6	5.64
4	9.519E7	1135	127.0	6.28
2	9.111E6	253	28.3	6.11
2	2.208E7	735	81.3	6.24
2	1.674E7	314	34.3	5.97
2	6.109E6	4561	512.7	6.92
2	1.809E8	306	35.0	5.57
2	2.416E6	398	42.6	8.91
2	6.017E7	259	28.3	4.44
2		723	78.4	5.29
2	4.810E6	361	40.8	8.16
2	2.892E7	234	26.6	5.58
3	2.676E7	180	20.7	5.64
2	8.255E6	1669	188.4	6.19
2	2.738E7	813	90.9	6.16
3	1.940E7	414	46.6	6.90
2	1.072E7	497	53.0	9.23
2	2.014E7	604	66.0	7.49

2	6.200E7	1044	116.0	5.38
2	1.541E7	1576	173.4	6.57
3	3.499E7	282	32.3	5.08
2	2.599E7	392	42.4	5.66
2	9.906E6	254	28.8	6.68
2	1.003E7	2015	224.2	7.96
2	1.818E7	559	62.8	5.99
2	1.571E7	515	57.6	6.79
2	4.756E7	661	71.4	6.73
2	7.282E7	62	7.3	11.37
2	8.775E7	307	34.8	7.18
2	1.402E7	2528	279.3	7.59
2	9.182E6	645	72.9	6.64
3	6.985E7	226	25.3	7.39
2	5.771E6	1614	181.3	6.90
2		464	51.9	6.02
2	6.820E7	229	25.0	5.16
2	6.866E6	662	72.3	5.64
2	3.019E7	303	33.9	6.98
2	4.379E7	867	98.9	5.55
2	1.480E7	624	69.7	5.90
2	2.310E7	264	28.5	7.56
2	1.151E7	1839	204.1	5.20
2	2.180E7	1295	141.8	6.18
2	1.088E7	853	97.3	7.05
2	3.509E7	687	77.6	7.05
2	4.626E7	149	17.1	7.12
2	2.255E7	716	78.9	6.46
2	1.042E8	221	24.1	7.24
2	1.228E7	1117	124.7	7.69
2	1.631E7	2125	233.3	6.65
3	7.271E7	246	27.6	6.95
2	1.538E7	433	48.6	5.52
5	2.095E7	1263	136.7	5.73
1	2.053E7	478	52.2	6.42
2	8.467E7	261	29.2	5.45
3	3.346E7	499	55.0	5.49
2	1.100E7	2370	267.3	5.99
2	2.497E8	215	25.3	9.70

2	8.174E7	163	18.4	8.73
3	9.826E6	799	88.4	7.80
2	1.597E7	618	69.3	5.24
2	1.128E7	1156	129.6	5.35
2	5.660E7	207	23.0	6.20
3	7.944E7	257	29.3	5.52
2	3.903E7	365	40.9	5.58
2	1.194E7	677	75.2	7.36
2	2.407E7	1096	120.5	5.63
2	8.769E7	119	11.9	4.03
2	1.210E7	785	86.1	7.33
2	9.521E6	899	98.8	6.77
2	6.007E7	325	37.1	6.13
2	6.871E7	614	66.8	6.48
2	4.186E7	297	33.0	5.00
3	1.917E7	275	30.4	9.29
1		427	47.0	7.12
1	5.449E7	124	13.8	10.45
2	1.572E7	234	25.8	8.54
1	3.880E7	612	67.6	7.08
3	1.132E7	1869	209.4	6.76
2	2.536E7	1233	136.5	5.30
2	3.318E7	516	58.0	8.82
2		407	45.8	5.85
2	4.937E6	2388	267.7	5.25
2	3.617E7	318	36.4	5.48
2	1.096E8	478	52.3	8.60
2	9.692E6	1025	115.2	4.79
2	3.153E7	330	36.2	8.76
2	9.839E6	541	60.7	8.00
2	2.428E7	275	32.2	7.84
2	7.961E6	467	52.2	7.30
2	8.801E7	134	15.6	5.12
2	6.269E7	1030	116.0	6.80
1		505	56.2	7.05
4	3.356E7	236	27.9	9.11
3	6.037E6	520	57.7	5.69
2	5.315E7	236	26.3	7.75
2	8.785E7	131	14.5	4.94

2	1.585E7	953	105.8	6.33
2	9.635E6	406	45.4	7.43
1	1.592E7	650	72.7	5.91
2	3.181E7	197	23.0	5.69
3	1.369E7	653	72.0	7.61
1	9.111E6	563	62.9	5.77
1	2.082E7	183	20.0	8.62
2	5.256E7	264	29.3	8.59
2	5.371E7	408	45.2	6.24
1		704	78.0	6.16
2	9.935E6	291	32.7	4.73
2	5.774E7	251	27.9	7.43
2	2.405E7	686	77.1	5.64
2	2.724E7	442	48.4	6.62
2	3.003E7	1243	139.2	5.41
3	2.703E7	1649	178.4	6.39
3	4.918E6	1530	170.1	5.94
1	1.483E8	267	29.0	4.41
2	7.631E7	159	18.0	5.53
2	1.876E8	112	12.4	9.33
1	3.608E6	1308	145.5	6.28
2	3.999E7	561	61.5	5.52
2	7.145E6	344	38.6	9.33
2	2.453E7	261	28.9	5.26
2	2.292E7	284	30.2	6.23
3	1.262E7	1025	116.3	8.84
2	6.597E7	321	35.2	5.68
1	1.299E8	122	12.8	6.29
1	2.239E7	1085	121.8	6.39
2	9.100E6	128	14.4	9.31
3	1.419E9	106	12.3	10.96
3	1.911E8	147	16.8	5.43
2	1.086E8	313	35.2	8.21
2	1.180E7	8161	915.7	6.64
2	1.975E7	772	87.2	9.17
2	3.744E7	228	25.0	4.88
2	1.321E7	366	40.7	7.81
1	2.495E7	351	39.0	8.68
2	7.780E7	703	78.3	6.37

2	4.057E7	801	87.3	5.68
2	1.537E7	1255	139.2	6.05
1	8.881E6	1692	188.5	7.58
2	1.699E8	265	29.7	5.48
2	1.172E7	512	56.4	5.02
1	9.203E6	679	74.3	6.23
2	1.735E7	1360	153.8	7.23
2	3.976E7	372	40.7	5.44
2	3.647E6	1321	145.1	5.07
2	1.944E7	697	77.1	6.25
2	1.295E7	1252	138.4	8.29
1		275	30.9	6.58
1	4.298E7	387	42.4	9.19
3	3.787E7	1235	137.9	8.35
1	3.256E7	342	38.5	8.25
2	1.190E7	1954	219.3	6.70
2	1.207E8	125	14.2	8.44
1	1.397E7	264	30.3	8.38
2	3.931E7	621	69.4	5.81
1	8.229E7	397	42.7	7.77
2	7.037E7	977	108.8	5.45
2	1.450E7	3378	377.7	6.52
2	9.865E6	430	48.6	6.61
2	1.451E7	650	73.9	5.58
2	2.129E7	744	83.2	6.49
1	2.256E7	201	22.8	6.87
1	3.537E7	278	30.0	6.79
1		201	21.6	4.97
2	1.415E7	458	50.5	8.56
1	2.612E7	544	59.7	7.43
1		1858	203.8	5.44
2	1.140E7	512	57.0	5.86
2	2.854E7	599	69.1	6.84
1	2.728E7	298	33.9	8.21
1	1.452E7	509	56.5	8.76
1	1.490E7	344	38.4	6.93
2	9.118E7	351	39.5	4.64
1	4.235E6	422	46.4	8.37
2	1.538E8	568	62.7	6.70

1	3.671E7	441	48.4	7.91
2	6.036E6	528	58.2	6.83
2		1523	167.2	7.78
1	5.050E6	245	26.8	6.29
2	6.500E7	178	20.1	8.84
2	1.086E7	1037	115.4	6.74
1	1.383E7	499	55.5	7.05
2	5.182E7	434	50.3	8.81
3	5.423E7	147	16.4	4.61
2	1.490E7	644	71.9	6.18
1	3.717E6	70	8.0	7.02
1	8.653E7	186	19.6	6.79
2	3.202E7	376	41.5	7.23
1	6.097E6	512	57.1	7.96
2	2.766E7	1118	121.7	5.67
1	4.969E6	491	55.1	7.58
2	3.938E7	176	20.8	6.71
2	6.661E6	1499	175.0	10.02
2	3.900E7	723	79.6	5.27
2	3.312E7	238	25.5	8.16
2	5.313E7	292	33.0	7.39
1		116	12.9	9.16
2	3.353E7	351	38.8	6.43
2	3.324E7	432	48.8	6.47
1		299	33.7	5.50
1	6.021E7	141	15.9	7.65
1	3.280E6	1403	154.6	7.24
1	4.378E7	628	64.7	9.26
2	4.298E7	756	84.4	9.38
1	2.614E7	452	50.6	8.25
2	8.465E7	368	40.6	8.59
1	1.120E7	512	56.3	8.19
1	2.183E7	651	70.7	7.02
2	4.514E7	97	10.3	4.49
2		825	90.7	6.83
1	2.719E7	711	78.5	6.28
1	1.559E7	552	61.3	4.93
1	6.008E6	617	69.9	7.50
1	4.046E7	449	52.1	7.53

2	1.879E7	1139	128.5	7.88
2	1.785E7	974	107.2	7.84
2	1.846E7	751	83.2	5.43
2	1.166E8	239	26.4	6.89
1	6.357E7	800	91.7	6.24
1	3.585E6	1769	201.1	6.15
2	1.225E7	326	36.4	6.54
1	1.094E7	529	57.8	4.75
1	5.895E7	268	30.2	7.61
2	8.813E7	139	16.0	5.36
1	1.906E7	1252	137.3	5.96
1	1.067E8	121	13.8	9.04
3	7.609E7	179	21.1	8.98
1	1.784E7	270	29.9	5.11
1	7.659E6	677	76.0	7.31
1	3.311E7	342	37.5	6.38
1	2.025E7	218	25.1	7.18
1		1023	110.1	6.57
2	3.282E8	61	7.2	4.21
1	7.979E7	183	20.6	8.15
1		1722	189.3	7.25
1	7.955E6	1698	188.6	7.44
1	1.992E6	161	18.0	4.31
1	8.053E7	348	39.8	7.44
2	1.299E7	1459	161.1	7.46
3	7.499E6	407	45.1	6.47
1	1.273E7	933	104.1	5.64
1		432	48.3	5.80
1	3.309E6	343	37.0	6.80
1		102	11.5	5.27
3	1.417E7	536	60.8	6.20
1	4.087E7	200	23.3	8.38
1	8.180E7	252	29.7	6.81
1	3.479E7	1024	112.8	5.40
1		674	75.0	8.13
2	4.792E7	593	65.7	5.83
1	3.246E6	577	65.1	5.50
1	6.178E6	1118	126.4	6.21
2	1.809E7	282	31.3	5.86

1		783	86.1	6.64
1	9.376E6	258	29.0	7.87
1	1.987E7	291	32.6	6.77
2	3.812E7	321	35.9	5.29
2	8.335E6	429	48.5	5.44
2	1.061E7	1442	160.9	5.78
2	3.313E7	688	76.8	6.62
1	3.488E7	231	25.6	9.31
1	1.741E7	239	25.9	5.38
1	1.308E7	137	15.1	9.70
1	5.717E7	131	14.8	6.52
1	1.414E7	624	69.5	5.78
1	1.896E7	542	59.4	5.12
1	4.566E7	498	55.6	5.94
1	9.378E6	108	11.5	5.00
1	1.776E7	608	69.1	5.72
1	6.715E6	519	58.2	5.67
1	8.137E7	120	13.4	6.09
1	9.915E6	413	44.3	8.68
1	6.066E6	1182	130.4	6.10
1	1.119E7	1177	126.2	6.37
1	2.018E7	1168	131.3	7.28
1	1.111E7	99	11.3	9.91
1		985	106.3	6.20
1	1.284E7	354	38.9	8.97
1	2.155E7	1236	136.3	6.81
1	1.396E7	1106	121.6	8.38
2	6.046E6	999	108.7	7.08
1	9.452E5	135	15.2	4.89
1		1241	137.1	6.54
1	3.161E7	294	32.2	6.90
1	1.722E7	254	29.3	5.33
1	5.305E7	161	18.1	7.55
1		388	43.3	5.08
1	1.234E7	557	61.5	6.87
1	6.864E7	177	19.6	5.19
1	1.169E7	1035	116.1	6.19
1		716	78.7	5.49
2	8.332E7	168	19.2	5.48

2	9.350E7	272	29.5	5.44
1	1.065E7	152	17.1	10.08
1	1.631E7	317	35.3	8.91
1	6.189E6	588	66.0	5.01
1	8.293E6	447	49.5	6.23
1	2.029E7	300	34.9	7.75
1		805	89.9	7.30
2	2.587E7	409	46.2	6.71
1	1.013E8	472	51.9	9.47
1	1.600E7	261	28.7	6.27
1		694	76.7	7.20
1	2.226E8	257	29.9	5.94
1	2.100E7	525	57.9	7.68
2	2.300E7	923	102.3	5.40
1	2.151E7	342	37.6	6.62
1	1.439E8	274	30.2	7.55
1		1432	159.4	8.70
1	2.979E7	813	90.2	7.99
1	2.677E7	672	74.2	5.62
1	1.434E7	331	35.6	7.59
1	1.666E7	148	16.6	8.28
1	1.169E7	549	60.7	6.98
1	2.332E7	217	24.6	5.24
1	1.402E7	894	97.4	6.06
1	8.710E7	254	28.7	5.15
1	1.379E7	204	22.8	5.60
1		361	40.8	5.77
1	5.639E7	252	28.3	7.46
1	1.672E7	323	36.8	5.38
1	4.680E6	185	21.4	8.88
1	2.488E7	269	28.0	8.18
1	9.377E6	737	80.7	7.36
1	9.407E6	202	21.5	6.65
1	5.309E6	238	25.6	9.26
1	2.128E7	145	16.3	6.77
1	3.976E6	102	11.9	8.41
1	1.221E7	527	57.0	5.58
1		583	63.7	7.91
1	1.877E8	319	35.6	4.73

1	4.221E7	204	23.9	7.59
1	6.465E6	2590	293.6	7.01
2	2.249E7	380	44.0	5.48
1	1.722E7	259	29.0	8.53
1	2.501E7	251	27.7	8.25
1		419	47.5	9.36
1	1.002E7	128	14.2	5.22
1	5.600E7	178	19.2	7.43
1	2.141E7	1356	148.6	7.18
1	4.027E6	257	28.5	6.01
1	1.137E8	140	16.4	9.72
1	9.450E6	486	53.7	5.95
1	1.449E7	590	65.0	8.19
1		1022	115.5	5.83
1	1.248E7	184	21.7	9.80
2	6.272E6	1513	164.8	6.46
1	2.746E7	182	20.8	5.91
1	1.043E7	750	84.5	5.33
1	5.074E6	328	36.8	8.63
1	2.356E7	478	54.8	9.77
1	6.493E6	806	90.5	6.98
1		402	45.4	5.19
1	1.925E7	192	22.1	9.70
1	2.471E7	910	102.0	5.62
1		346	37.3	6.51
2	8.613E7	495	55.7	7.58
1	1.550E7	484	52.8	6.96
1	1.482E7	198	21.9	5.03
1	2.299E7	583	63.2	8.68
1	1.739E7	209	23.0	7.09
1	5.199E7	211	24.0	6.79
1		201	22.3	9.61
1		201	22.1	5.58
1	3.043E6	325	35.2	7.46
1	3.774E6	2760	310.2	8.70
1		369	40.1	5.68
1		510	57.9	5.76
1	2.452E7	2077	235.1	8.15
2	1.088E8	217	23.4	5.76

1	1.530E7	301	33.9	8.73
1	9.737E6	370	42.5	5.90
1		1511	165.4	6.33
1		107	12.0	9.63
1	5.560E6	1092	121.3	5.40
1	3.834E7	721	77.0	7.34
1	8.895E6	823	91.5	8.87
1	1.153E7	562	63.2	6.80
1	7.724E6	780	87.3	5.90
1	2.283E7	168	19.0	5.12
1	7.672E7	220	23.9	7.97
1	2.213E7	1859	205.9	8.35
1		255	29.6	4.70
1	4.065E7	211	22.9	9.32
2	8.195E6	1073	119.0	7.01
2	2.319E7	170	20.2	4.89
1	9.347E6	370	40.6	6.77
1	3.761E7	234	26.0	8.31
1	8.117E6	104	11.8	9.09
1	2.665E7	680	74.6	5.05
1	1.637E7	240	27.4	8.34
1	4.991E6	793	87.7	6.81
1	5.612E6	233	25.6	4.42
1	5.220E7	227	25.4	8.43
1	2.197E7	198	22.6	7.20
1	5.933E8	1029	108.9	4.78
2	3.325E7	170	18.9	8.46
1	1.076E7	361	40.0	7.81
1		1571	173.5	8.00
1	1.620E7	218	24.8	4.60
1	2.940E8	122	13.4	6.25
1	2.259E7	221	23.9	6.02
1	4.289E6	1109	123.0	6.79
1	1.652E7	537	63.1	5.73
1		227	26.0	6.15
2	3.313E7	688	76.4	6.44
1		2013	221.1	6.46
1	4.932E7	264	29.9	4.53
1	8.728E7	359	38.3	6.37

1	1.132E7	738	81.3	6.83
1	2.080E7	399	44.4	6.76
1	7.428E6	164	18.7	8.10
1	9.980E6	320	34.3	9.92
1	2.070E7	258	27.9	7.75
1		177	20.3	8.43
1	2.392E7	158	17.6	7.99
1	7.311E7	134	14.6	4.79
1	5.328E7	224	24.7	9.89
1	1.341E8	154	17.7	7.78
1		1093	118.6	6.89
1	8.134E7	376	41.1	6.68
1	1.913E7	268	29.9	5.55
1	3.649E7	615	67.8	5.58
2	9.625E6	1603	178.2	5.60
1	9.811E6	335	36.2	8.09
1	3.323E7	476	51.8	4.69
1	2.592E6	749	82.1	5.16
2		716	79.2	6.61
1	7.960E6	363	40.3	5.76
2	2.967E7	285	31.4	6.67
1	8.486E7	229	25.7	5.11
1	9.230E6	263	29.4	6.07
1		496	55.4	7.65
1		694	76.9	7.62
1		877	98.9	5.53
1	1.242E7	846	93.9	6.48
1	1.454E7	1262	146.6	4.81
1	4.259E7	695	79.0	6.74
1		819	89.2	5.47
1	3.509E7	927	101.6	4.89
1	3.034E7	294	32.1	7.27
1	3.020E7	236	26.2	6.90
1	9.150E6	223	25.8	9.86
1	1.548E7	1452	157.2	5.43
1	1.502E8	98	10.8	7.18
1	1.873E7	432	47.3	5.85
1	9.521E6	189	21.7	8.10
1	7.982E6	344	37.4	5.25

2	1.219E7	900	103.8	7.31
1	1.460E7	705	79.2	5.31
1	1.589E7	320	35.6	6.06
1	1.227E7	344	36.3	6.04
1	1.464E8	161	18.7	4.78
1	9.791E6	369	40.7	4.84
1		114	12.7	5.22
1	1.342E7	2143	234.9	5.96
1	6.474E6	1180	133.2	8.76
2	5.498E7	134	14.4	5.94
1	1.684E7	459	50.7	5.92
1	1.089E7	1025	114.2	7.58
1	5.573E6	233	27.1	7.18
1	9.396E6	507	54.9	8.65
1	1.931E7	1229	138.4	5.45
2	3.657E7	187	21.3	5.39
1	1.672E7	463	49.8	8.91
1	3.937E6	402	44.2	7.31
1	1.927E7	56	6.5	7.18
1	9.829E6	301	32.8	7.05
1	6.044E7	109	12.6	6.01
1	5.079E6	409	46.8	6.52
1	1.065E7	907	100.3	5.73
1	5.792E8	127	14.3	9.99
1	2.023E7	72	8.3	10.98
1		1159	129.3	8.66
1		1375	155.1	6.84
1		386	43.6	5.00
1		869	93.9	6.76
2	1.566E8	500	59.9	7.46
1	1.268E7	1392	151.9	6.81
1		782	87.2	6.84
1	9.686E6	524	55.3	8.22
1	3.718E7	256	29.1	7.08
1	1.520E8	544	59.7	6.58
1		1262	140.2	6.19
1		257	28.6	9.92
1	3.216E7	151	17.4	4.70
1	3.210E7	184	20.3	9.60

1	2.800E7	1593	175.8	5.83
1	2.714E7	2307	261.0	8.82
1	4.082E8	86	9.8	5.05
1	8.345E6	578	65.4	9.39
1	1.061E7	297	33.5	7.06
2	1.577E7	184	20.8	5.82
1	6.765E6	361	39.7	6.58
1	1.374E7	3171	354.0	6.95
1		680	76.9	5.87
1		613	69.9	7.28
1	6.816E7	296	33.3	8.37
1	1.572E7	241	26.1	9.45
1		1101	120.6	6.81
1		304	34.3	8.12
1	1.189E7	390	43.5	5.30
1	5.179E6	1652	186.3	4.91
1	1.549E8	305	33.7	7.24
1	1.846E7	191	21.2	5.17
1	5.499E7	345	40.0	9.28
2	1.144E8	1423	160.0	7.66
1	5.783E6	486	53.0	6.71
1	1.440E7	1055	116.7	6.35
1	1.988E7	325	35.3	8.92
1	1.843E7	227	24.3	5.25
1	6.545E6	706	78.6	9.29
1	4.191E6	468	52.7	6.83
1	2.379E6	190	20.7	7.84
1	2.182E7	332	37.3	7.52
1	4.227E6	1179	129.0	5.03
1	3.798E7	194	21.9	8.27
1	3.974E7	448	50.1	7.34
1	9.669E7	882	98.7	6.71
1	4.606E6	193	22.1	5.22
1		139	15.5	8.69
1	1.768E7	857	94.4	5.00
2	7.802E6	2183	245.2	7.18
1	1.086E7	274	30.9	5.02
1	2.900E7	604	66.6	5.72
1	1.270E7	198	22.3	9.41

1	1.583E7	522	58.1	6.48
1	2.866E7	625	69.2	7.42
1	9.197E6	705	77.8	6.46
1	6.261E6	2290	253.5	8.47
1	2.657E6	601	66.9	6.27
1	2.020E7	294	32.3	6.79
1	2.004E7	1017	113.1	8.59
1	1.749E7	844	91.5	6.48
1	1.131E7	1306	143.7	6.51
1		105	12.3	9.11
1	7.600E6	839	93.8	6.43
1	1.228E7	1215	133.0	8.97
1	6.229E6	2247	250.7	6.46
1	1.308E7	365	41.0	6.32
1	1.118E7	648	72.5	5.20
1	3.708E6	814	90.5	6.54
1	1.094E7	488	54.8	7.91
1		1334	147.5	4.94
1		311	34.4	4.78
1	1.937E7	3619	407.1	6.52
2	5.596E6	547	61.6	6.46
1	2.665E7	463	50.6	5.38
1	2.441E7	436	49.0	8.31
1	4.702E6	787	87.2	8.34
1	5.277E7	157	17.3	6.76
1	9.408E6	511	56.7	6.87
1		1174	130.4	5.78
1		238	27.1	5.36
1	8.769E6	703	78.3	7.17
1	1.089E7	110	11.8	4.84
1	1.187E7	783	91.4	6.23
1	3.429E7	131	14.3	6.77
1	9.265E7	115	12.5	6.00
1	1.241E7	388	43.1	5.87
1	3.530E6	634	69.8	5.81
1	4.848E6	166	18.7	5.54
1	3.877E6	1587	176.7	5.99
1	1.321E7	150	17.1	6.79
1	2.463E7	610	66.2	9.63

1	1.764E6	305	33.6	6.71
1		768	84.0	7.94
1	6.436E7	178	20.2	5.59
2		6933	776.7	7.17
1		619	67.5	4.96
1	8.727E7	225	24.5	6.54
1	4.455E7	106	11.7	9.38
1	1.084E7	1248	138.9	7.75
1	1.170E7	365	41.4	6.86
1	3.743E7	302	34.4	7.28
1	6.303E6	1491	168.3	8.25
1	1.265E7	131	14.5	6.11
1	3.452E6	339	37.6	5.74
1	4.150E6	316	34.9	9.31
1		560	61.5	5.77
1	1.711E6	1007	110.8	6.06
1		486	54.1	8.54
1		6401	716.9	6.54
1	9.073E7	779	83.1	6.48
1		2497	282.0	7.12
1	4.206E6	328	37.2	8.22
1	1.796E7	1220	136.2	6.15
1	1.316E7	618	68.7	6.86
1	1.437E7	1021	113.8	8.82
2	1.626E7	1028	112.7	6.65
1	2.013E7	619	67.9	6.76
1	3.171E7	439	49.0	8.94
1	2.807E6	1353	153.1	6.44
1	3.212E7	97	10.9	8.06
1	1.597E7	325	36.4	5.85
1	9.168E7	381	42.2	4.82
1	4.291E8	90	10.0	7.90
1	8.028E6	779	88.3	9.16
1	8.797E6	295	33.5	4.91
1	1.551E7	598	65.5	5.83
1	1.939E7	328	34.8	6.14
1	5.298E7	315	34.3	4.84
1	3.289E7	211	24.6	6.02
2	2.088E6	149	16.8	7.66

1	7.543E7	66	7.3	5.21
1	4.569E7	323	35.2	6.67
1	6.308E6	1617	178.6	6.92
1		116	12.9	6.11
1	4.225E7	797	87.4	5.81
1	6.928E7	183	20.9	5.21
1	7.764E6	637	70.1	6.90
1	2.269E6	368	41.2	5.12
1	3.210E6	869	96.6	5.95
1	1.689E7	236	25.2	6.40
1	5.613E6	254	28.6	6.58
1	1.766E7	261	27.8	6.39
1	2.048E7	1156	125.8	6.99
1	4.855E7	523	57.7	5.27
1		467	53.6	5.53
1		316	34.8	6.05
1	1.329E7	952	104.6	6.49
1		166	18.1	5.43
1	6.984E6	1291	144.4	5.82
1	1.681E7	229	24.6	6.19
1	5.155E6	488	55.2	7.65
1		886	99.1	8.03
1	4.644E7	205	23.7	6.93
1	9.224E6	502	55.6	6.54
1		275	31.9	4.86
1	1.532E7	7838	883.9	7.09
2	8.227E6	356	40.8	6.73
1		1661	183.4	7.08
1	1.643E7	841	94.2	6.21
1	1.246E7	527	58.0	5.44
2	1.430E8	246	27.1	9.13
1		397	44.9	7.02
1	2.244E7	882	96.0	8.10
1	2.519E6	757	84.5	7.20
1	1.778E7	273	31.5	6.10
1	1.391E7	865	96.7	4.86
1	2.946E6	121	13.7	10.80
1	4.964E7	1337	150.9	8.59
1	4.773E6	937	105.1	8.37

1	1.967E6	451	50.4	8.29
1	1.790E7	556	60.8	5.07
1		1741	189.7	6.54
1		532	59.2	7.66
1		572	63.2	6.47
1		535	59.5	9.03
1	6.808E6	501	55.3	7.40
1	3.377E7	568	61.7	6.49
1		2081	227.1	6.27
1	2.960E7	254	28.6	4.48
1	1.235E7	1457	161.0	6.55
1	8.898E6	45	5.0	4.73
1	3.283E7	342	39.0	8.21
1	4.486E8	180	20.0	6.11
1	1.035E7	932	102.2	4.77
1	7.066E6	158	17.4	5.71
1	1.759E7	210	23.4	8.43
1	1.622E7	147	16.4	5.67
1	2.621E7	1031	113.2	6.10
1	3.340E7	151	16.8	7.91
1	1.009E7	2590	285.3	5.40
2		1015	113.1	6.74
1	1.219E7	6956	776.2	6.70
1		1917	210.9	6.07
1		654	73.6	8.48
1	8.919E6	240	27.8	4.70
1		1432	159.1	8.44
1		236	26.4	8.63
1	4.544E7	233	26.7	9.44
1	1.039E7	769	86.5	7.80
1	4.279E7	333	36.1	5.00
1		558	59.7	7.68
1	1.068E7	339	38.6	7.21
1		1008	112.9	5.38
1	5.501E6	514	57.9	5.90
1		1582	180.7	7.28
1	1.956E7	437	48.8	6.46
2	3.427E7	1461	160.5	8.97
1	6.953E6	331	37.0	5.86

1	1.873E7	594	65.8	6.38
1	6.348E7	217	24.2	9.32
1	2.440E7	742	83.3	7.37
1	2.482E7	164	18.5	6.38
1	4.144E7	293	32.3	4.89
1	3.017E7	269	30.2	9.04
1		988	112.1	6.02
1	1.318E8	235	25.6	8.43
1	2.283E7	103	11.2	5.34
2	1.835E7	976	109.9	5.94
1		1252	138.4	5.63
1	9.060E6	159	18.5	7.27
1	7.129E6	597	67.5	5.41
1	1.198E7	188	20.9	7.96
1	8.173E6	925	105.1	6.80
1	8.697E6	597	65.5	6.49
1	1.297E7	126	14.1	5.20
1	1.791E7	160	17.3	9.07
1	9.147E6	385	42.2	6.61
2	5.507E7	308	36.1	6.90
1	8.451E7	166	19.3	8.07
1	5.888E7	111	12.5	6.77
1		351	39.9	6.84
2	2.922E7	261	29.4	6.21
1	6.262E7	74	7.9	10.01
1	3.445E7	83	9.6	5.41
1		172	19.6	7.21
1	2.228E7	1221	135.2	5.29
1	1.981E6	270	29.9	8.16
1	8.943E6	745	84.3	5.14
1		214	24.4	5.69
1	2.063E6	158	17.8	8.95
1		1480	166.3	6.62
2	3.100E7	404	46.3	8.60
1	2.220E7	654	71.9	6.13
1	1.276E7	886	97.1	7.75
1	8.889E7	180	20.8	6.14
1	4.807E6	863	97.4	8.84
1		1031	114.6	7.11

1	1.373E7	106	12.5	4.84
1	4.577E6	1879	212.3	7.59
1		695	77.9	5.31
1	2.913E6	3024	338.7	9.23
1		625	69.3	7.99
1	4.682E6	863	94.2	8.40
1	2.283E6	140	16.4	5.22
1		519	57.1	6.77
1		229	26.3	8.91
1		539	59.2	6.93
1		1065	118.5	6.57
2	4.021E7	224	24.8	6.27
1	4.690E7	190	22.1	9.47
1	8.086E7	108	12.4	5.36
1		430	46.1	10.15
1	2.131E7	929	102.9	6.04
1		1205	134.4	5.92
1	2.656E7	291	31.8	4.82
1	2.817E7	610	69.5	5.69
1	1.348E7	564	62.0	5.26
1	8.638E6	469	51.7	5.88
1	4.007E7	293	32.8	8.60
1	2.362E8	6946	775.9	7.27
1	3.113E7	512	58.4	5.82
1	3.439E6	604	68.7	7.06
1	8.174E6	324	35.7	5.01
1	5.613E6	363	40.0	8.13
1		532	60.6	7.17
1	1.044E8	786	84.9	6.90
1	1.267E7	332	36.3	7.02
1		38	4.2	4.15
1	1.875E7	310	35.3	8.94
1		457	49.4	7.11
1	5.865E6	708	79.6	7.96
