# Supplementary material for: Insights into translocation mechanism and ribosome evolution from cryo-EM structures of translocation intermediates of Giardia intestinalis
Source: Nucleic Acids Res. 2023 Mar 13;51(7):3436–51. doi: 10.1093/nar/gkad176 (PMC10123126; doi:10.1093/nar/gkad176)
Supplement: gkad176_Supplemental_Files [file gkad176_supplemental_files.zip › GI_NAR_Supplementary_20230203.pdf]

Supplementary Information

Insights into Translocation Mechanism and Ribosome Evolution from Cryo-EM Structures of Translocation-Intermediates of *Giardia intestinalis*

Soneya Majumdar[†1], Andrew Emmerich[†1], Sascha Krakovka[1], Chandra Sekhar Mandava[1], Staffan Svärd[1], Suparna Sanyal[*1].

1 Department of Cell and Molecular Biology, Uppsala University, Box-596, 75124 Uppsala, Sweden

† These authors contributed equally to this work.

* To whom correspondence should be addressed. Email: suparna.sanyal@icm.uu.se

This document contains

Supplementary Figures 1 – 15

Supplementary Tables 1 - 4

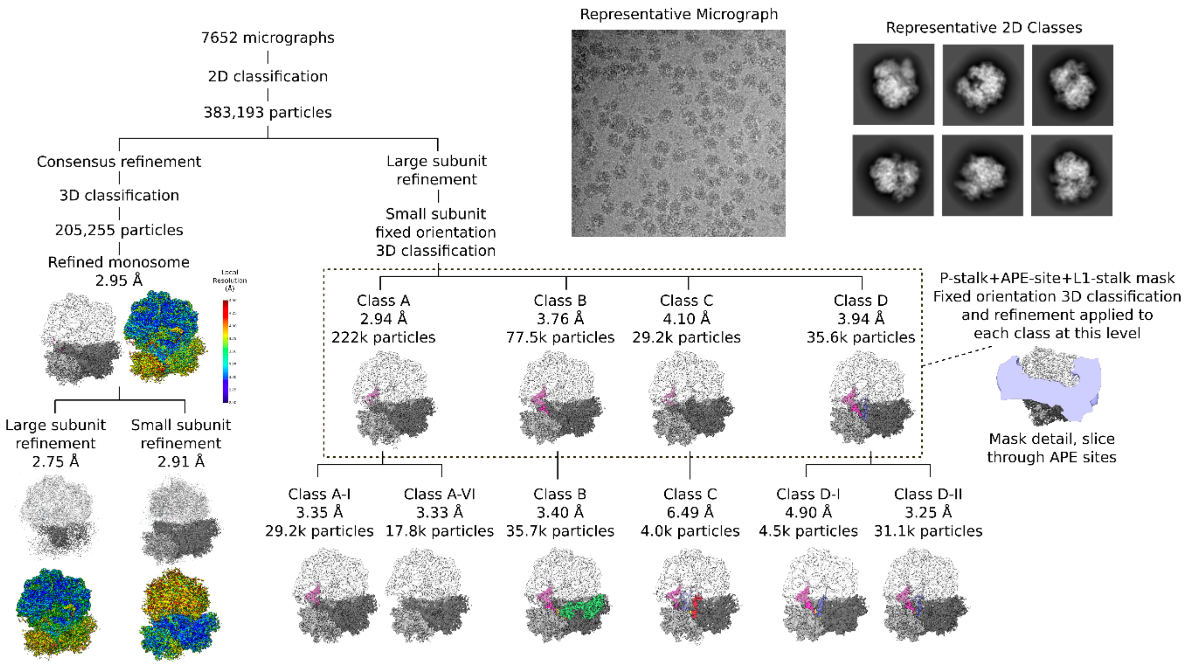

Supplementary Figure 1a. Cryo-EM single particle image reconstruction workflow dealing with ribosome conformational and compositional heterogeneity.

From a data set containing 7652 micrographs a consensus refinement (left path) yielded three maps with global followed by LSU and SSU masked refinements, with which a *de novo* structure was constructed. Structural heterogeneity (right path) was dealt with by performing a LSU masked refinement of all 383k particles followed by a fixed orientation SSU masked 3D classification yielding four major classes with different SSU conformations: A, B, C and D. These four classes were then subjected to a further classification to deal with remaining compositional heterogeneity using an APE site mask (shown on the right in purple) yielding additional classes (right side, bottom row) with varying tRNA/eEF2 compositions.

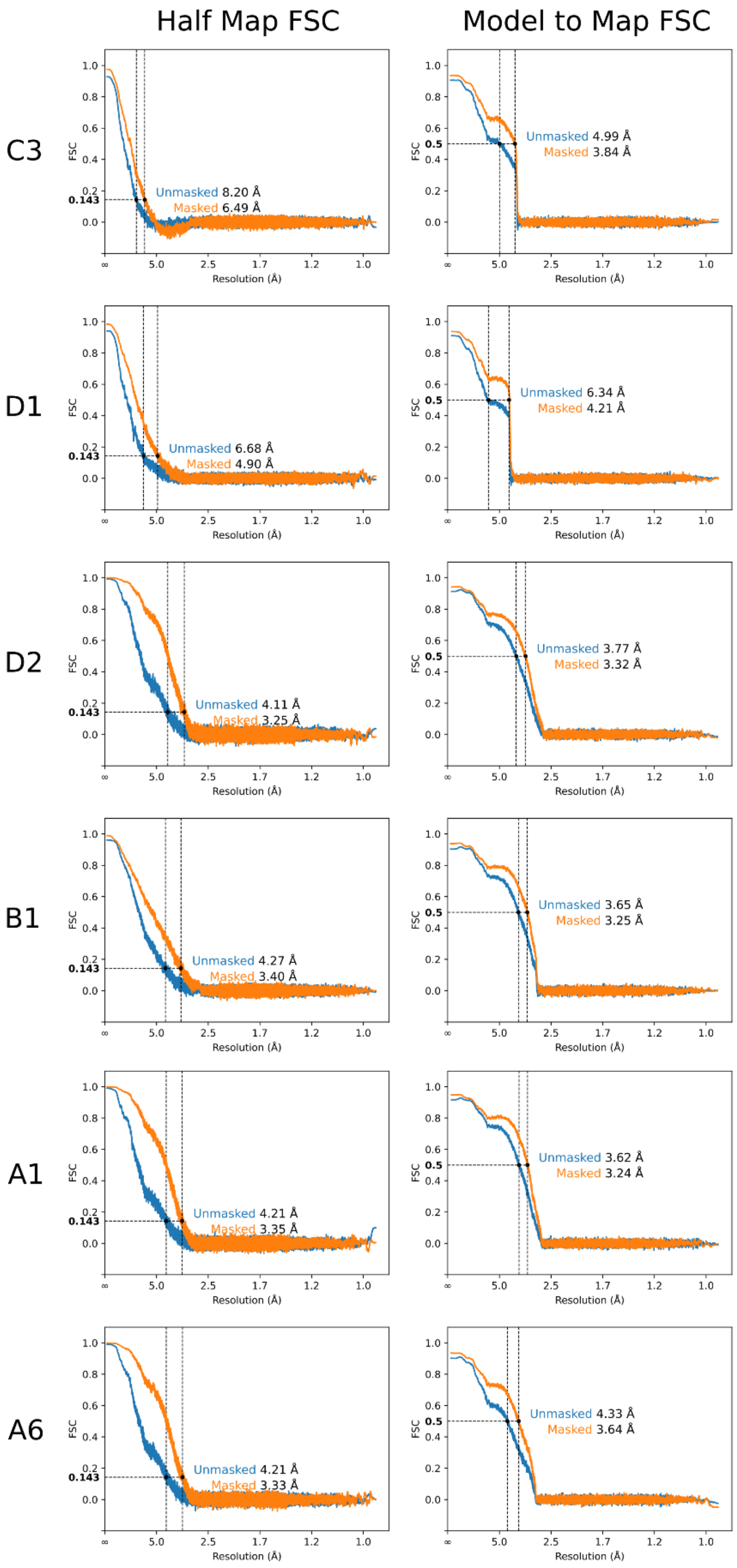

Supplementary Figure 1b. Fourier shell correlation (FSC) curves for half maps (left panel) and map to model (right panel) with and without masking for different translocation states of *Giardia* ribosome. The resolution is determined at the FSC=0.143 cut-off (from the masked half maps).

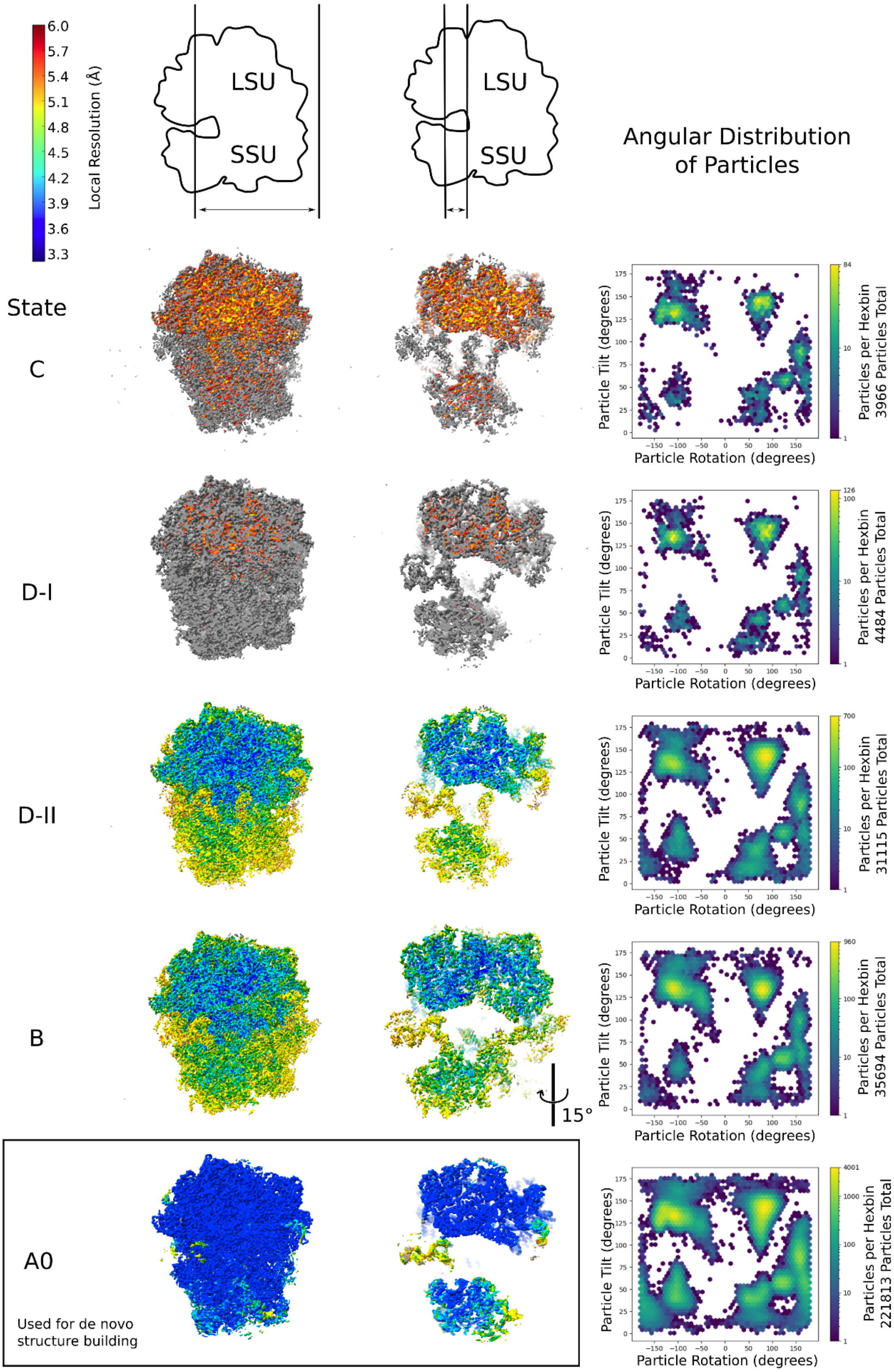

Supplementary Figure 2a. Local resolution (left and middle) and angular distribution of particles (right) in different translocation states of *Giardia* ribosome.

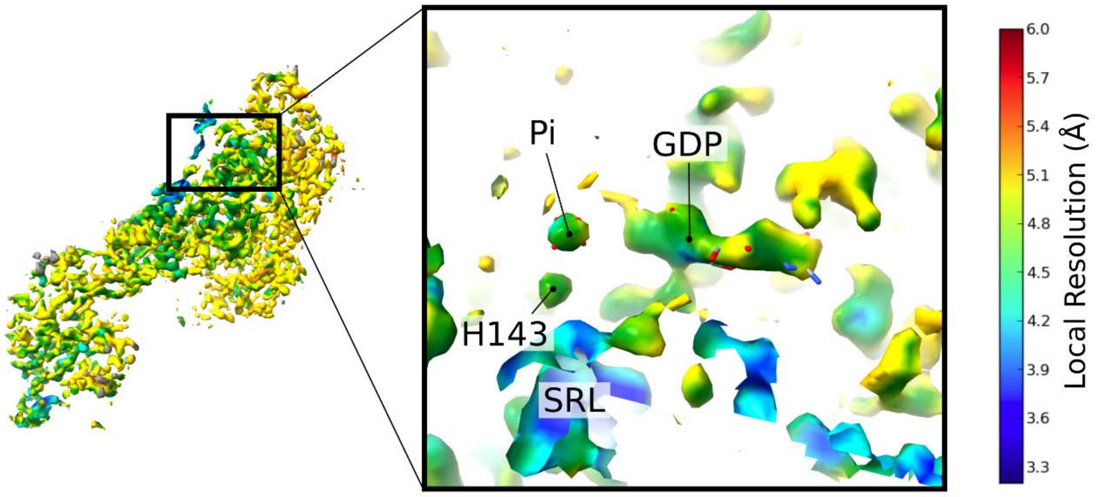

Supplementary Figure 2b. Local surface resolution of eEF2 bound to state B of the translocating *Giardia* ribosome.

Local resolution of eEF2 bound to the state B (left). The inset shows zoomed view of the nucleotide binding pocket with density for GDP and Pi. Part of the Sarcin ricin loop (SRL) can also be seen in the selected section.

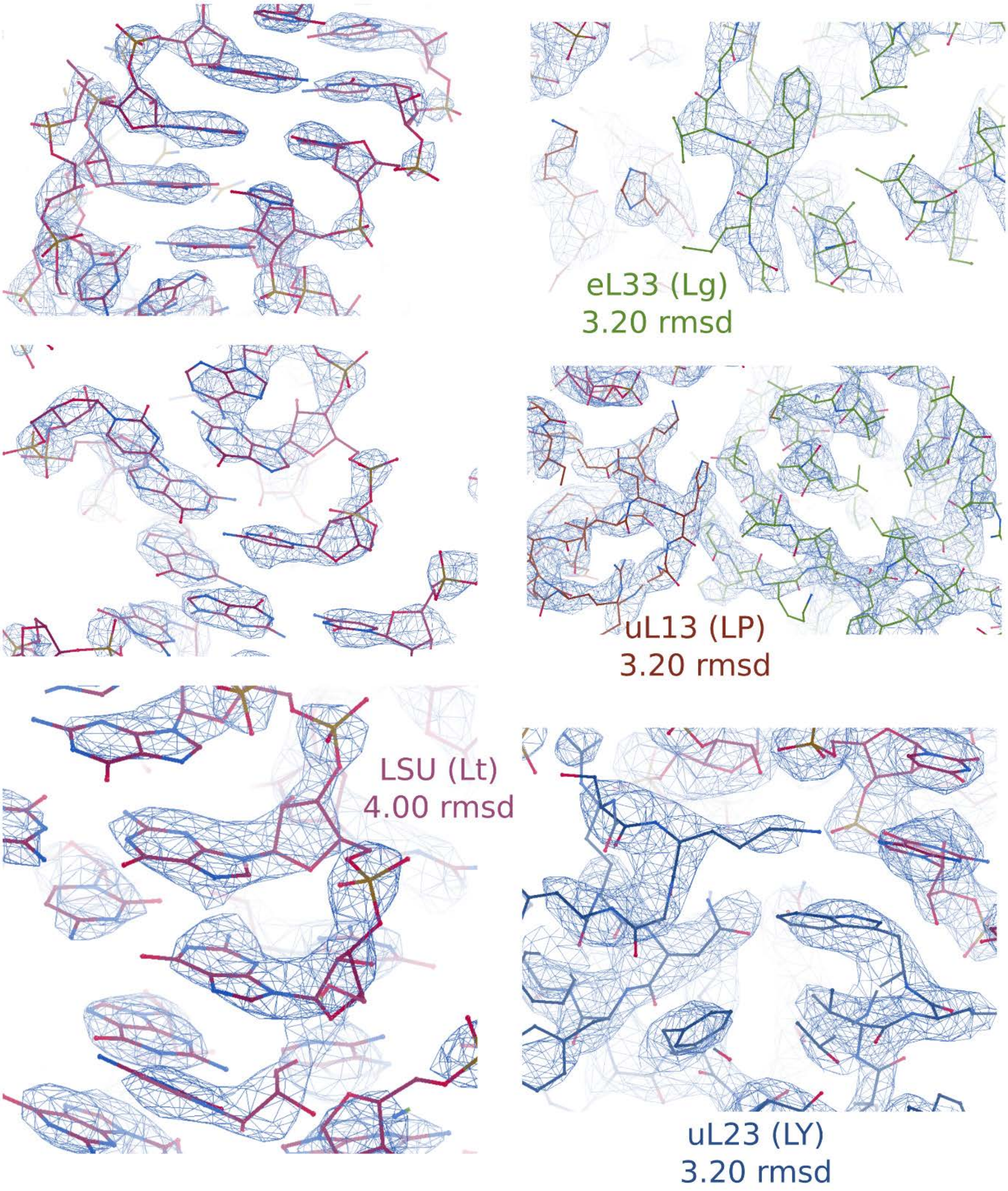

Supplementary Figure 3. Representative sections of cryo-EM densities depicting high-resolution features of the map for *Giardia* ribosomes (class A1).

Left- randomly selected segments of LSU rRNA. Right- randomly picked regions of LSU ribosomal proteins, eL33, uL13 and uL23.

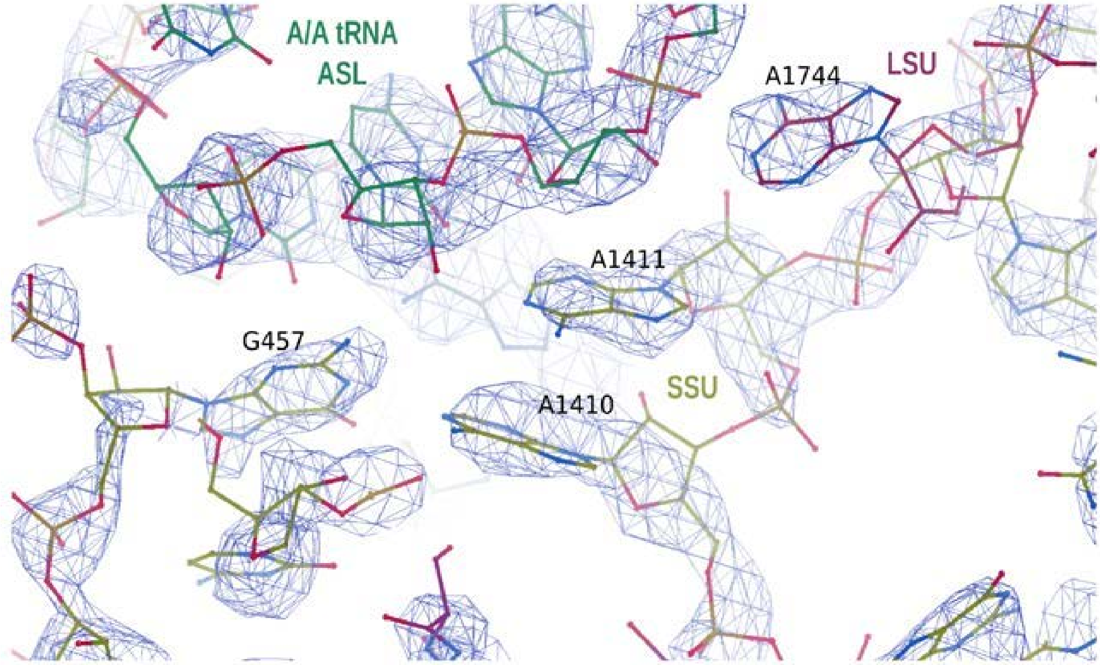

Supplementary Figure 4. Cryo-EM density map and model fitting of the decoding center (DC) of the *Giardia* ribosome.

Clear density can be seen for the three monitoring bases A1410, A1411 and G457 at the DC together with the anti-codon step loop (ASL) of the A/A tRNA as seen in State C (Map level 0.0157V, 3.20 σ).

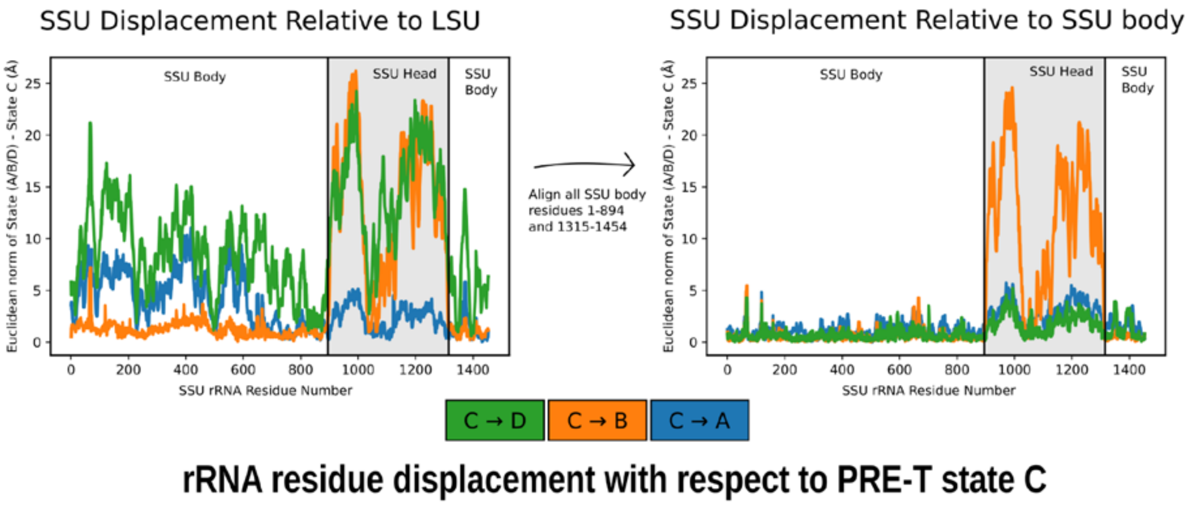

Supplementary Figure 5. Displacement of rRNA residues of states D, B and A with respect to the pre-translocation state, C.

(Left) SSU displacement relative to LSU shows concerted movement of both the SSU head and body during ratcheting (C→D), with only a large head conformational difference between the classic PRE-T and chimeric hybrid state B, and moderate SSU body movement whilst undergoing back ratcheting (C→A, identical to A→C).

(Right) When aligning the SSU body residues (1-849, 1315-1452) of all four translocation intermediates, we see only minor fluctuations in the displacement of the SSU body atomic positions, while the SSU head of states D and A compared to state C show moderate movement, limited to a maximum of 5 Å. The internal SSU rearrangement consisting of SSU head movement is highlighted in the comparison of C→B, where we see a maximum displacement of around 25 Å.

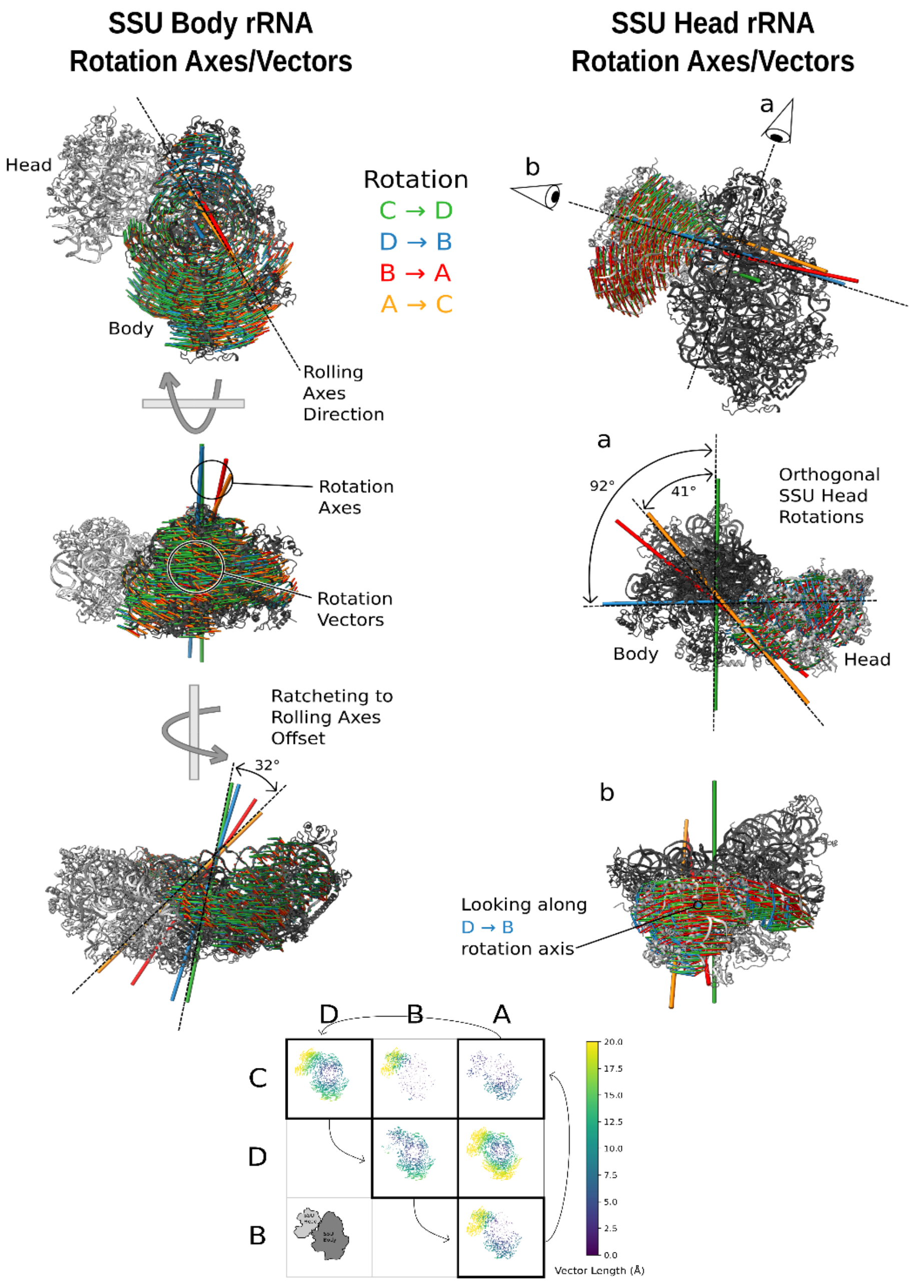

Supplementary Figure 6. **Analysis of the axes and vectors for 'subunit rolling' and 'subunit rotation/ratcheting' during translocation in *Giardia* ribosomes.**

The axes and vectors of 'subunit rolling' and 'subunit rotation' in the SSU body (left panel) and SSU head (right panel) are identified using atomic coordinates of the translocation intermediate states of *Giardia* ribosome. We find the angle of intersection of the ratcheting (C→D) and rolling (A→C) axes to be 32° (bottom left) in *Giardia* ribosome, which was previously found to be orthogonal in higher eukaryotes. SSU head movements show a dramatic change in rotation axes (right panel). Looking along sight line 'a' we note an angular intersection of 92° of the SSU head movement axes from the PRE-T states, upon head swivel.

(Bottom) Pairwise comparison of SSU rRNA atomic positions, with proposed translocation order indicated with thick-lined boxes and arrows. Length of vectors plotted and colored with purple indicating no movement and yellow 20 Å.

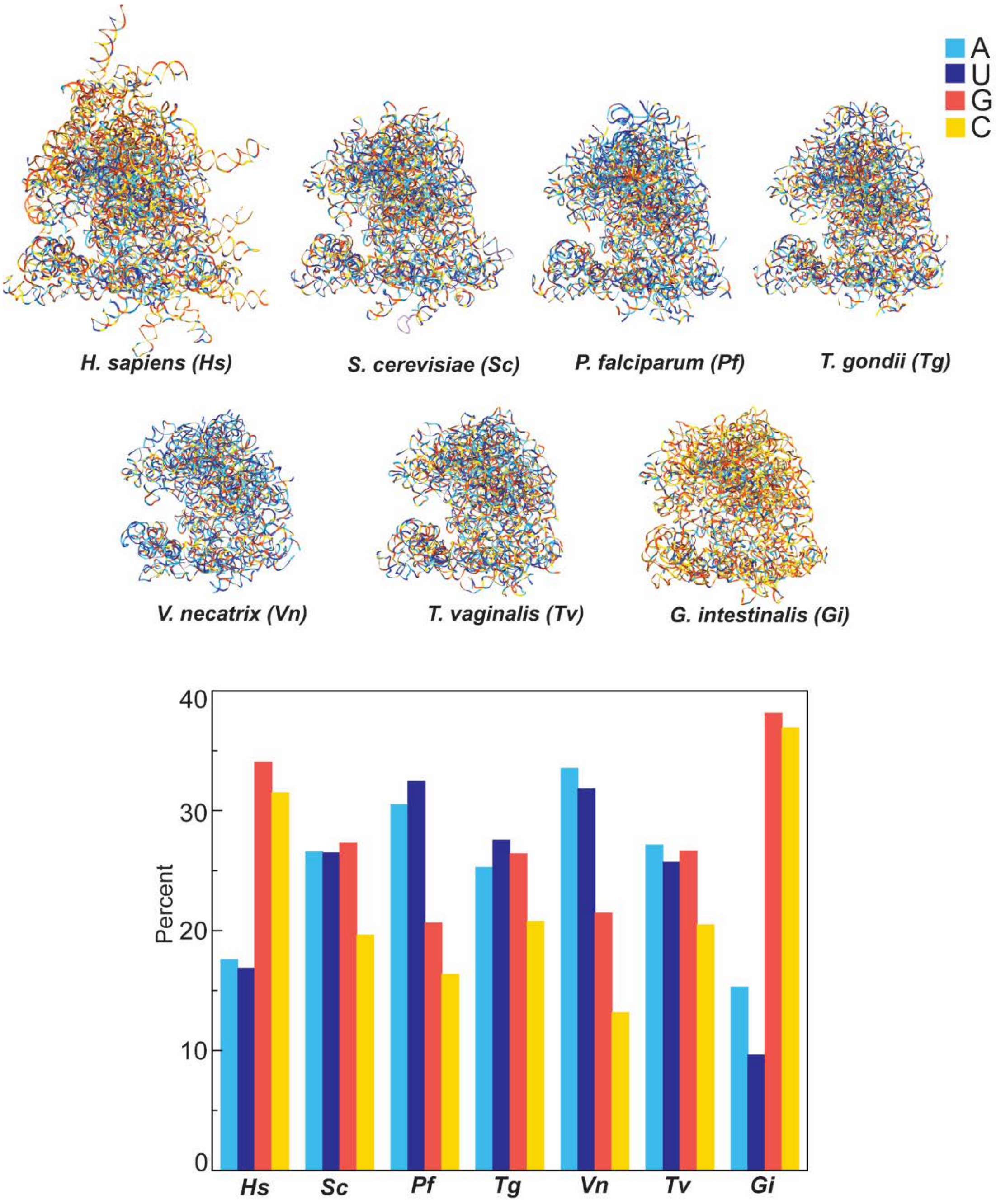

Supplementary Figure 7. *Giardia* ribosomes have high rRNA GC content compared to ribosomes from other eukaryotes.

(Top) Distribution of A, U, G, and C nucleotides across the rRNA in *Homo sapiens* (Hs), *Saccharomyces cerevisiae* (Sc), *Plasmodium falciparum* (Pf), *Toxoplasma gondii* (Tg), *Vairimorpha necatrix* (Vn), *Trichomonas vaginalis* (Tv) and *Giardia intestinalis* (Gi), (bottom), percent A, U, G and C of the ribosomes from the above organisms compared in a histogram.

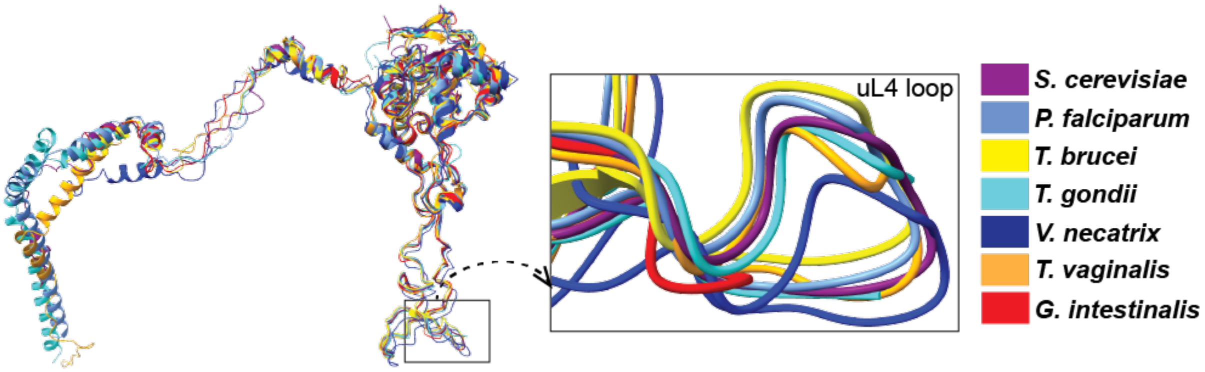

Supplementary Figure 8. Comparison of the uL4 loop at the ribosomal exit tunnel between different ribosomes.

Superimposition of uL4 from *Saccharomyces cerevisiae* (purple), *Plasmodium falciparum* (cornflower blue), *Trypanosoma brucei* (yellow), *Toxoplasma gondii* (cyan), *Vairimorpha necatrix* (navy blue), *Trichomonas vaginalis* (orange) and *Giardia intestinalis* (red). *Giardia* uL4 (red) has a uniquely shorter uL4 loop (red arrow) at the peptide exit tunnel compared to the other eukaryotic organisms, which is why the exit-tunnel of the *Giardia* ribosome has only one constriction similar to bacterial ribosomes.

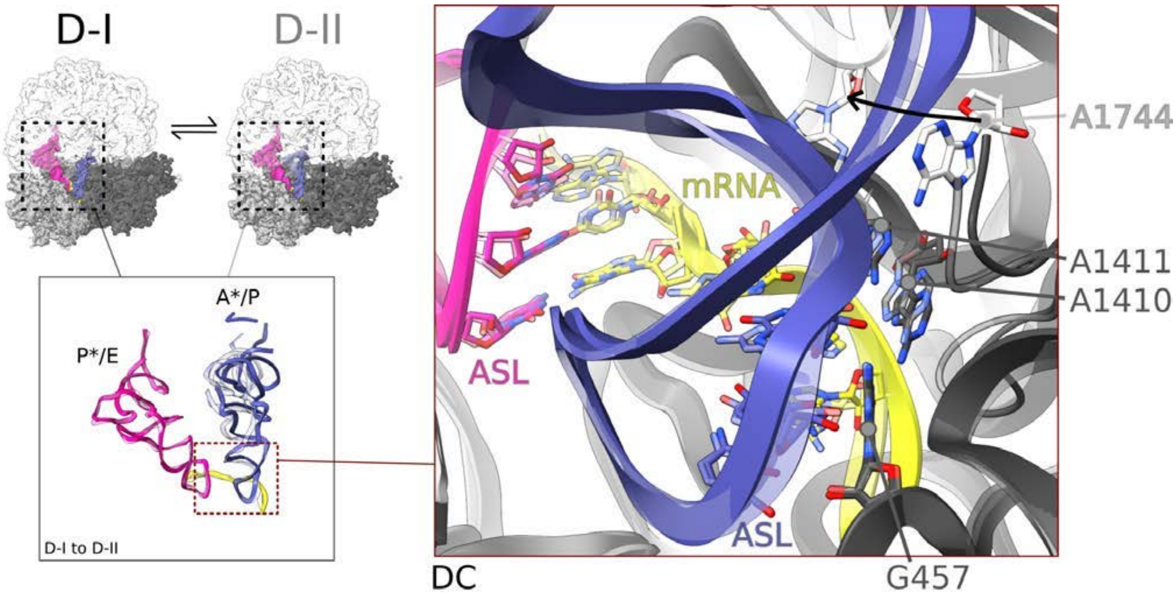

Supplementary Figure 9. Comparison of the D-I and D-II states with focus on the tRNAs and rRNA bases in the DC.

D-I and D-II are representations of the dynamic state of the pre-translocation ribosome with tRNAs bound to hybrid P*/E and A*/P states. Superimposition of the tRNAs (left, bottom inset) shows higher deflection of the A*/P tRNA in D-II state (purple transparent) from D-I state (purple opaque) compared to the P*/E tRNA. The magnified view of the DC shows that Helix 69 is pulled away during transition from D-I to D-II and the ASL (A*/P tRNA) interaction with H69 nucleotide A1744 breaks due to difference in conformation of A1744 (black arrow) on Helix 69 between D-I and D-II state.

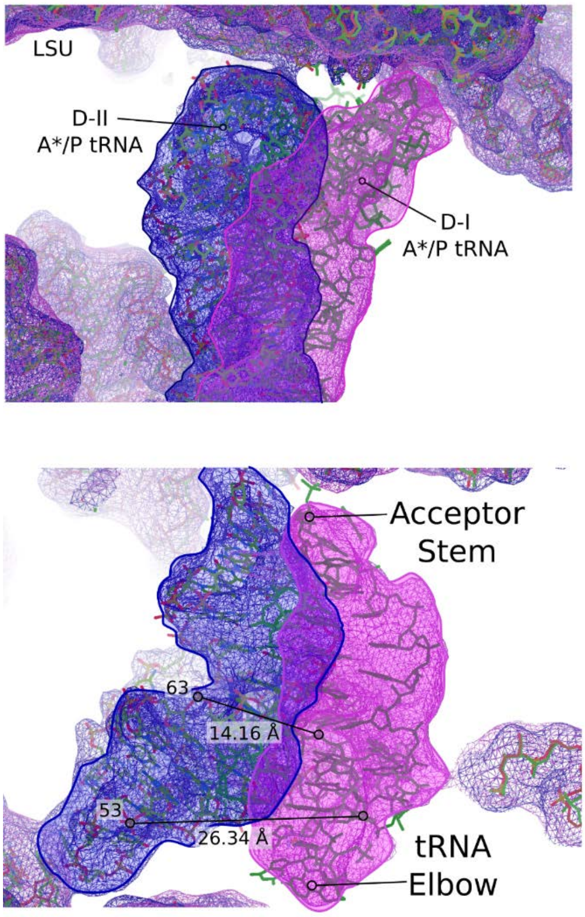

Supplementary Figure 10. Movement of the A*/P tRNA elbow while transition from the translocation state D-I to D-II.

Electron density maps displayed in two orientations for D-I (pink) and D-II (blue) focused on the A*/P tRNA. (top) The view is oriented with LSU/acceptor stem uppermost and SSU/ASL lowermost. (bottom) Orientation looking from the LSU side of the tRNAs towards the SSU. The acceptor stem and elbow of the tRNAs are indicated. The minimum and maximum displacement of the A*/P tRNA elbow is shown (14.16 Å and 26.34 Å).

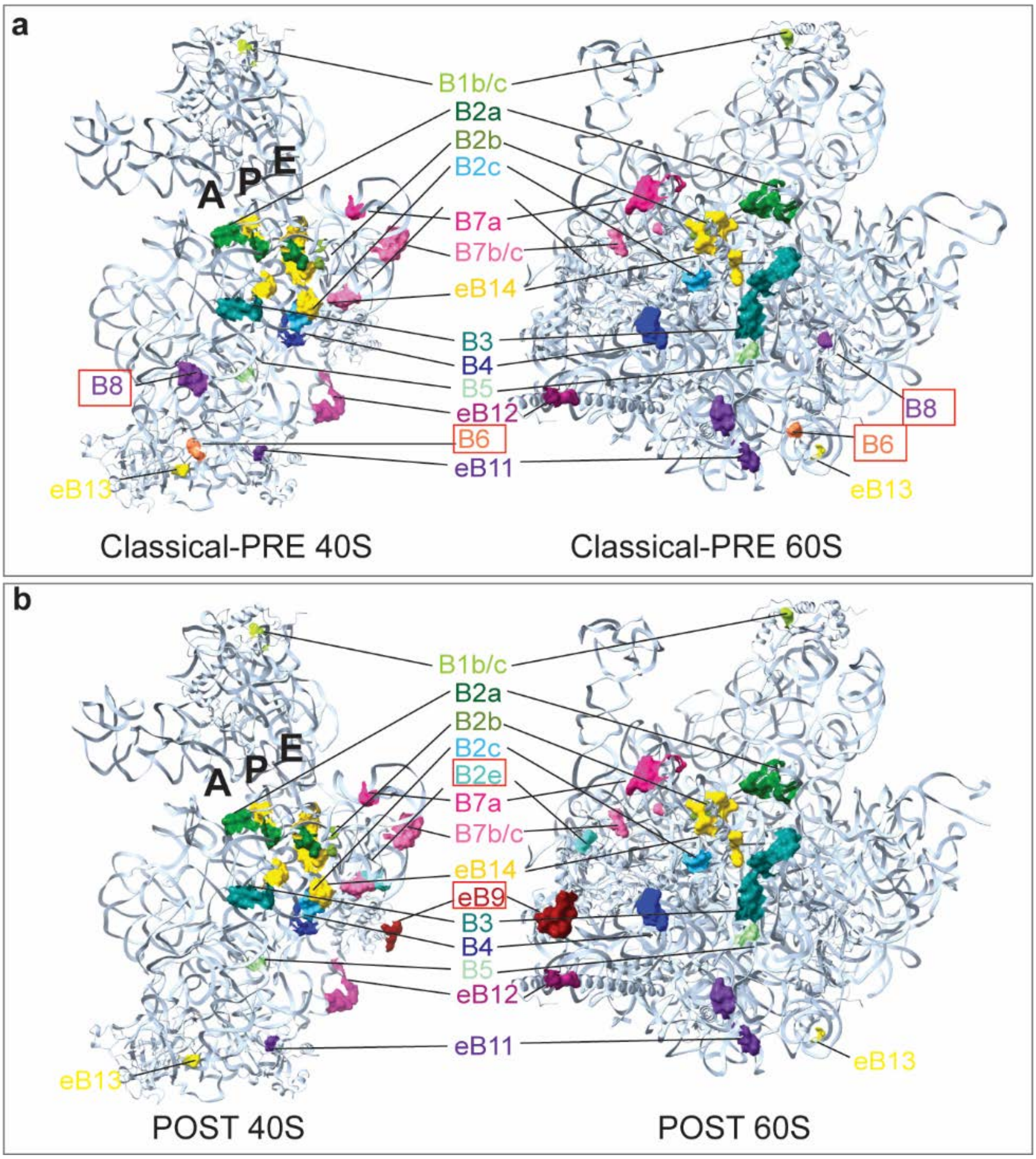

Supplementary Figure 11. Remodeling of ribosomal inter-subunit bridges of *Giardia* ribosome due to SSU rolling.

The inter-subunit bridges show alteration due to subunit rolling during translocation, which can be seen by comparing those in the post-translocation and classical pre-translocation state (*see* Figure 4). Regions of the SSU/40S (left) and LSU/60S (right) contributing to inter-subunit bridges are colored and labeled similarly in the classical pre-translocation state (a) and the post-translocation state (b). The bridges unique to each state are boxed.

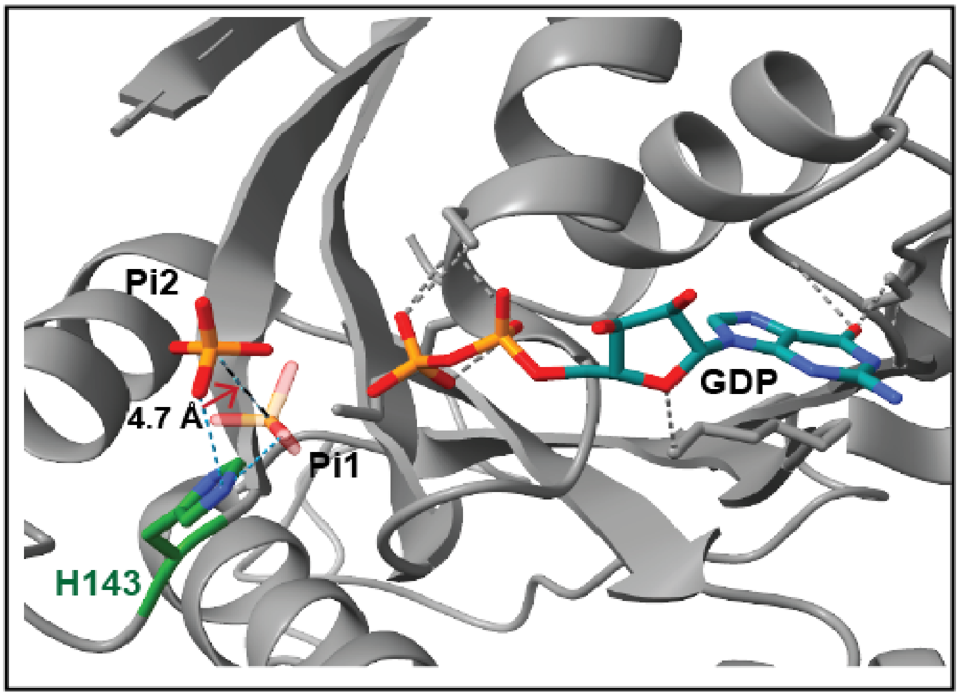

Supplementary Figure 12. Close view of the nucleotide binding pocket of *Giardia* eEF2 and *E. coli* EF-G in the GDP+Pi state.

Superimposition of *Giardia* eEF2 bound to GDP and Pi (shown as Pi2) with *E. coli* EF-G bound to GDP and Pi (shown as Pi1) (PDB:7PJV). The comparison clearly portrays that the positioning of the Pi2 in our structure is distinctly different from the Pi1 in the previously reported GDP+Pi state. Pi2 is positioned 4.7 Å away towards the exit path of Pi, for which we name it as 'leaving Pi'. The catalytic His (H143) from Switch-II, however, can interact with the Pi at both positions, Pi1 and Pi2.

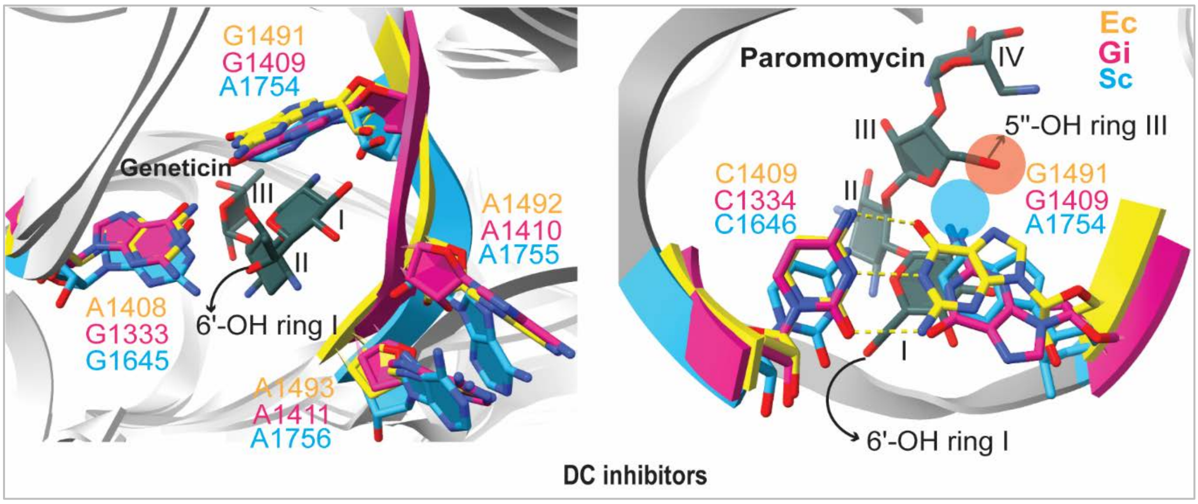

Supplementary Figure 13a. Close view of the decoding center (DC) with or without DC inhibitors.

Superimposition of *S. cerevisiae* (Sc) structure in complex with Geneticin (cyan, PDB:5NDG)) on the *E. coli* (Ec, yellow, PDB: 7K00) and *G. intestinalis* (Gi, pink) ribosome (left). Superimposition of *E. coli* structure in complex with paromomycin (yellow, PDB:7K00)) on the *S. cerevisiae* (Sc, cyan, PDB: 4U3N) and *G. intestinalis* (Gi, pink) ribosome (right).

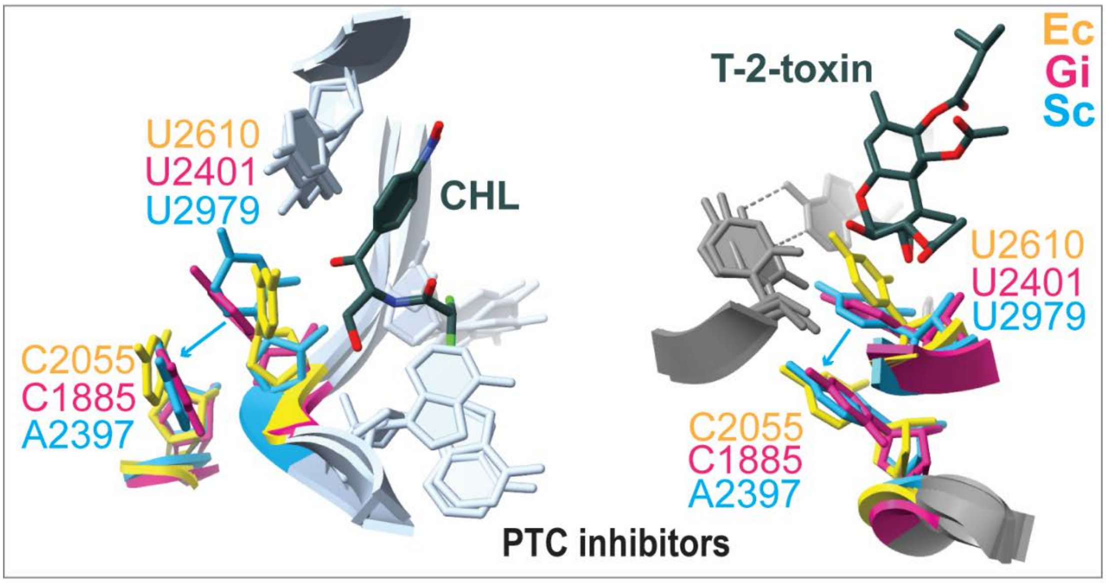

Supplementary Figure 13b. Close view of the peptidyl transferase center (PTC) with or without PTC inhibitors.

Superimposition of *E. coli* ribosome structure (yellow, PDB: 4V7T) in complex with chloramphenicol (CHL) on the *S. cerevisiae* (Sc, cyan, PDB: 4U3N) and *G. intestinalis* (Gi, pink) ribosome (left). Superimposition of *S. cerevisiae* ribosome structure in complex with T2-toxin (Sc, cyan, PDB: 4U6F) on the *E. coli* (yellow, PDB: 7K00) and *G. intestinalis* (Gi, pink) ribosome (right).

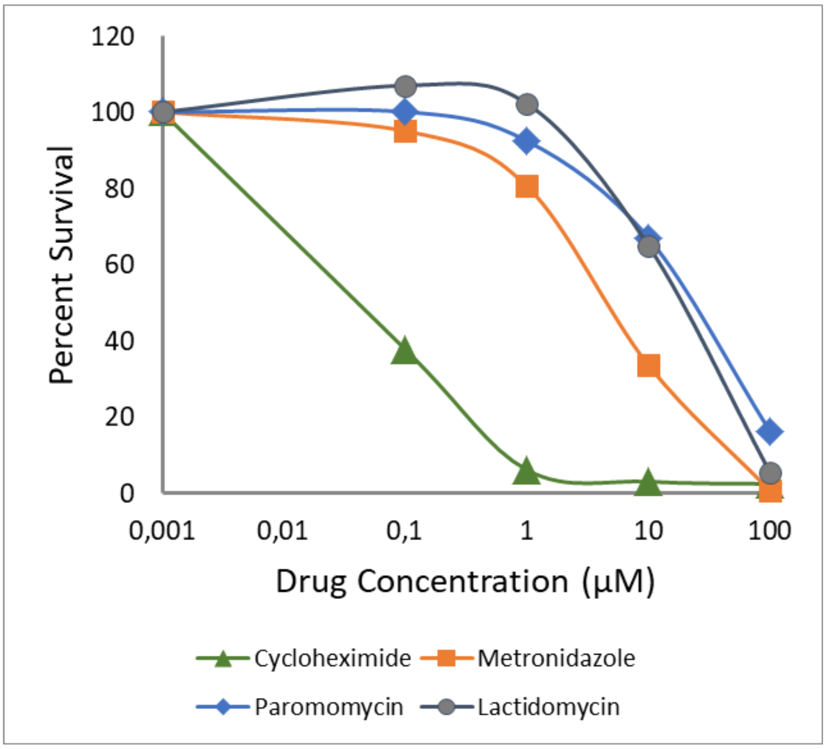

Supplementary Figure 14. Survival test for *Giardia intestinalis* in the presence of various antibiotics.

To test the effect of various antibiotics on survival of *Giardia intestinalis*, an exponentially growing culture (diluted to 5 x 10^3 cells/ml) in TYDK media was divided into smaller batches to which either only the solvent (DMSO) or the testable antibiotics at various concentrations were added after pre-incubation of 2 hours for allowing surface adherence. The cultures were incubated for 72 hours under anaerobic conditions. Population survival was tracked by measuring the amount of ATP present in a test culture (with added antibiotics) compared to the amount of ATP present in the control culture (DMSO) using Cell Titer-Glo reagent (Promega). The percent survival was calculated by dividing the signal ratio from the test samples with that from the solvent control. The experiments were done in duplicates and the plots show one representative data set. The solid lines are polynomial fits of the data.

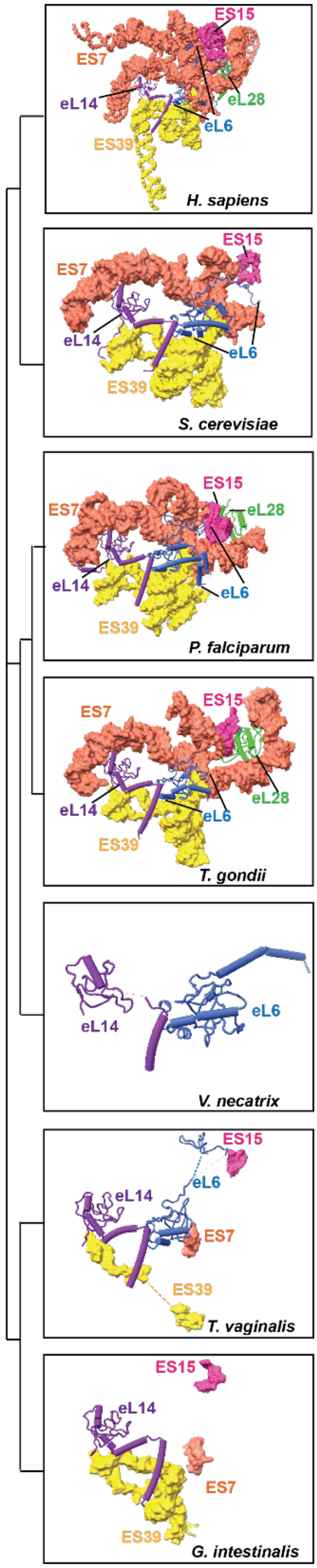

Supplementary Figure 15. Co-evolution of ribosomal proteins eL28, eL6 and eL14 with rRNA expansion segments, ES7, ES15 and ES39.

The illustrations present comparison of the above-mentioned elements across species. *Homo sapiens* (PDB: 4V6X), *S. cerevisiae* (PDB: 4U3N), *P. falciparum* (PDB: 3J79), *T. gondii* (PDB: 5XXB), *V. necatrix* (PDB: 6RM3), *T. vaginalis* (5XY3) and *G. intestinalis* (PDB: 8BRM). While *G. intestinalis* lacks eL6 and eL14, all other ribosomes possess those ribosomal proteins. Interesting to note the gradual co-evolution of complexity in the r-proteins as well as in ES7, ES15 and ES39 (bottom to top).

Supplementary Table 1. Particle distribution amongst states.

Total Particles (after Class2D)	383193	Proportion
State A	221813	0.579
State B	75500	0.197
State C	29156	0.076
State D	35599	0.093
Excluded	21125	0.055

Supplementary Table 2. Cryo-EM Data Table.

	A-I	A-VI	B	C	D-I	D-II
**Model Composition**						
Chains	76	75	79	79	78	78
Non-hydrogen atoms	175377	173960	180812	178793	178904	177590
Protein residues	10156	10206	10867	10190	10403	10228
RNA bases	4363	4306	4386	4538	4466	4467
Ligands	0	0	1 (PO4)	0	0	0
**Refinement**						
Resolution (Å) 0.143	3.35	3.33	3.40	6.49	4.90	3.25
Overall Biso (masked)	54.6	55.9	54.5	57.13	70.0	56.6
**RMS deviations**						
Bonds (A)	0.011	0.010	0.012	0.010	0.011	0.010
Angles (deg)	1.322	1.527	1.616	1.565	1.577	1.540
**Validation (proteins)**						
Molprobity score	2.37	2.90	2.86	3.05	3.15	2.87
Clashscore, all atoms	3.64	5.4	5.48	6.55	7.47	5.57
Good rotamers (%)	86.52	60.40	65.47	56.60	54.00	65.58
Rotamer Outliers (%)	13.66	39.64	34.52	43.39	45.99	34.36
**Ramachandran plot**						
Favored (%)	94.86	94.05	94.04	93.07	92.18	94.03
Outliers (%)	0.69	0.89	0.93	1.05	1.19	0.93
**RNA**						
Correct Sugar Puckers (%)	95.83	96.45	96.37	95,97	95.52	96.35
Angle Outliers (%)	0.08	0.13	0.20	0.13	0.15	0.15
Bond Outliers (%)	0.00	0.00	0.00	0.00	0.00	0.00
Good Backbone Conformations (%)	78.28	56.25	58.05	51.3	46.98	58.05
**Accession Codes**						
PDB	8BR8	8BRM	8BSI	8BSJ	8BTD	8BTR
Cryo-EM Maps	EMD-16211	EMD-16222	EMD-16225	EMD-16226	EMD-16228	EMD-16235

Supplementary Table 3. (A-C) Pairwise rotation angle characteristics of the SSU body and SSU head for each state to another. (D) L1 stalk angles for all A states relative to state A-I. Angles given in degrees to three decimal places.

**A. Rotation of the SSU body (All LSUs Aligned)**			
From/To	D	B	A
C	9.600	1.049	-4.559
D		-8.943	-13.57
B			-4.902
**B. Rotation of the SSU head (All LSUs Aligned)**			
From/To	D	B	A
C	11.147	-17.213	-2.473
D		13.322	-13.128
B			19.596
**C. Rotation of the SSU head (SSU bodies Aligned)**			
From/To	D	B	A
C	2.044	17.474	-2.599
D		15.378	-2.149
B			-16.65

**D. L1 stalk angles, relative to A-I**	
State	Angle (degrees)
A-II	2.445
A-III	2.514
A-IV	1.614
A-V	22.19
A-VI	38.963

Supplementary Table 4. Comparison of the *Giardia* ribosome with bacteria, archaebacteria and eukaryotic ribosomes for structural elements and ribosomal dynamics.

	**Bacteria (*E. coli*)**	**Archaebacteria (*P. furiosus*)**	***Giardia intestinalis***	**Eukaryote (*S. cerevisiae*)**
**Ribosomal RNA (rRNA)**	3	3	4	4
**Ribosomal proteins (rps)**	51	64	78	79-80
**rRNA Expansion segments (ES)**	missing	2 SSU ES and 3 variable regions. 10 LSU ES and 5 variable regions.	4 SSU ES in which 3 are highly reduced, 14 LSU ES in which 10 are highly reduced.	10 SSU and 18 LSU rRNA ES
**DC - Nucleotides corresponding to position 1408 and 1491 in *E. coli***	A and G	A and G	G and G	G and A
**PTC- Nucleotides interacting with U corresponding to position 2504 in *E. coli***	C and A	A and A	C and U	A and A
**E-site tRNA interactions**	uS7 and 23S rRNA	uS7, eS2, eL42 and 23S rRNA	uS7, eS2, eL42 and 25S rRNA	uS7, eS2, eL42 and 25S rRNA
**Inter-subunit bridges**	11	11	18 (two are reduced eB11 and eB12)	19
**Peptide-exit tunnel**	One constriction	One constriction	One constriction	Two constriction (second constriction) formed by an extension in uL4)
**Subunit-rolling**	Absent	Not reported	Intermediate with A-site narrowing by ~6 Å and E-site widening by ~1 Å.	Pronounced with A-site narrowing by~6 Å and E-site widening by ~5 Å.
