# Supplementary material for: Insights into translocation mechanism and ribosome evolution from cryo-EM structures of translocation intermediates of Giardia intestinalis
Source: Nucleic Acids Res. 2023 Mar 13;51(7):3436–51. doi: 10.1093/nar/gkad176 (PMC10123126; doi:10.1093/nar/gkad176)
Supplement: gkad176_Supplemental_Files [file gkad176_supplemental_files.zip › Movie legends.docx]

**MOVIE LEGENDS**

**Movie A**. The dynamics of ‘subunit rotation’ and ‘subunit rolling’ during translocation in *Giardia*  ribosome. The movie presents the axes and the vectors of rotation and rolling movements of the SSU body and head during transitions between different translocation states (D 🡪B, B🡪A, A🡪C and C🡪D). The offset between the rolling and rotation axis in *Giardia* ribosome is 32° unlike 90° in higher eukaryotes (43).

**Movie B**. The dynamics of tRNA, eEF2 and L1 stalk during ribosomal translocation on *Giardia*  ribosome. The movie presents the sequential movement of the tRNAs, binding and release of eEF2 and the movement of the L1 stalk while transition between different translocation states. Noteworthy is the demonstration of tRNA elbow displacement from D-I to D-II state and 39° L1 stalk movement in transition between the A states.
